# Supplementary material for: Systematic functional analysis of Leishmania protein kinases identifies regulators of differentiation or survival
Source: Nat Commun. 2021 Feb 23;12:1244. doi: 10.1038/s41467-021-21360-8 (PMC7902614; doi:10.1038/s41467-021-21360-8)
Supplement: Supplementary file 1 — Supplementary Information [file 41467_2021_21360_MOESM1_ESM.pdf]

Supplementary Information

Systematic functional analysis of *Leishmania* protein kinases identifies regulators of differentiation or survival

Baker N.[1,2#], Catta-Preta C.M.C.[1,2#], Neish R.[1,2], Sadlova, J.[3], Powell B.[4], Alves-Ferreira E.V.C.[1,2], Geoghegan V.[1,2], Carnielli, J.B.T.[1,2], Newling K.[2], Hughes, C[1,2]., Vojtkova, [3]. Anand, J.[1,2], Mihut, A.[2], Walrad, P.B.[1,2], Wilson, L.G.[1,4], Pitchford, J.W.[2,3], Volf, P.[3] and Mottram J.C.

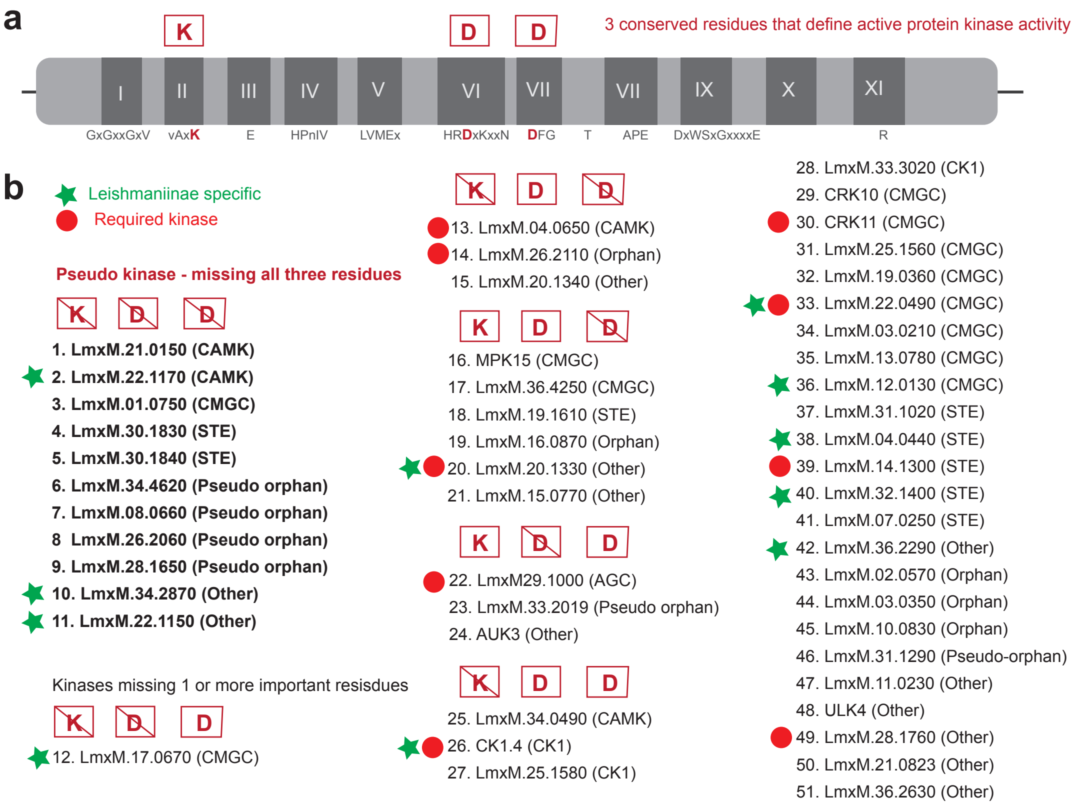

Supplementary Figure 1. **Putative pseudokinases of *L. mexicana*.** **a** Schematic of a conserved protein kinase structure. The highly conserved lysine and two aspartic acid residues required for an active kinase are highlighted. **b** List of all protein kinases missing one or more of these residues. Boxed K, D, D letters with diagonal line indicate missing residues. The 11 pseudokinases are indicated in bold text. Green star denotes protein kinases that are specific to the *Leishmaniinae* family. Solid red circle indicates that the protein kinase is required for promastigote survival.

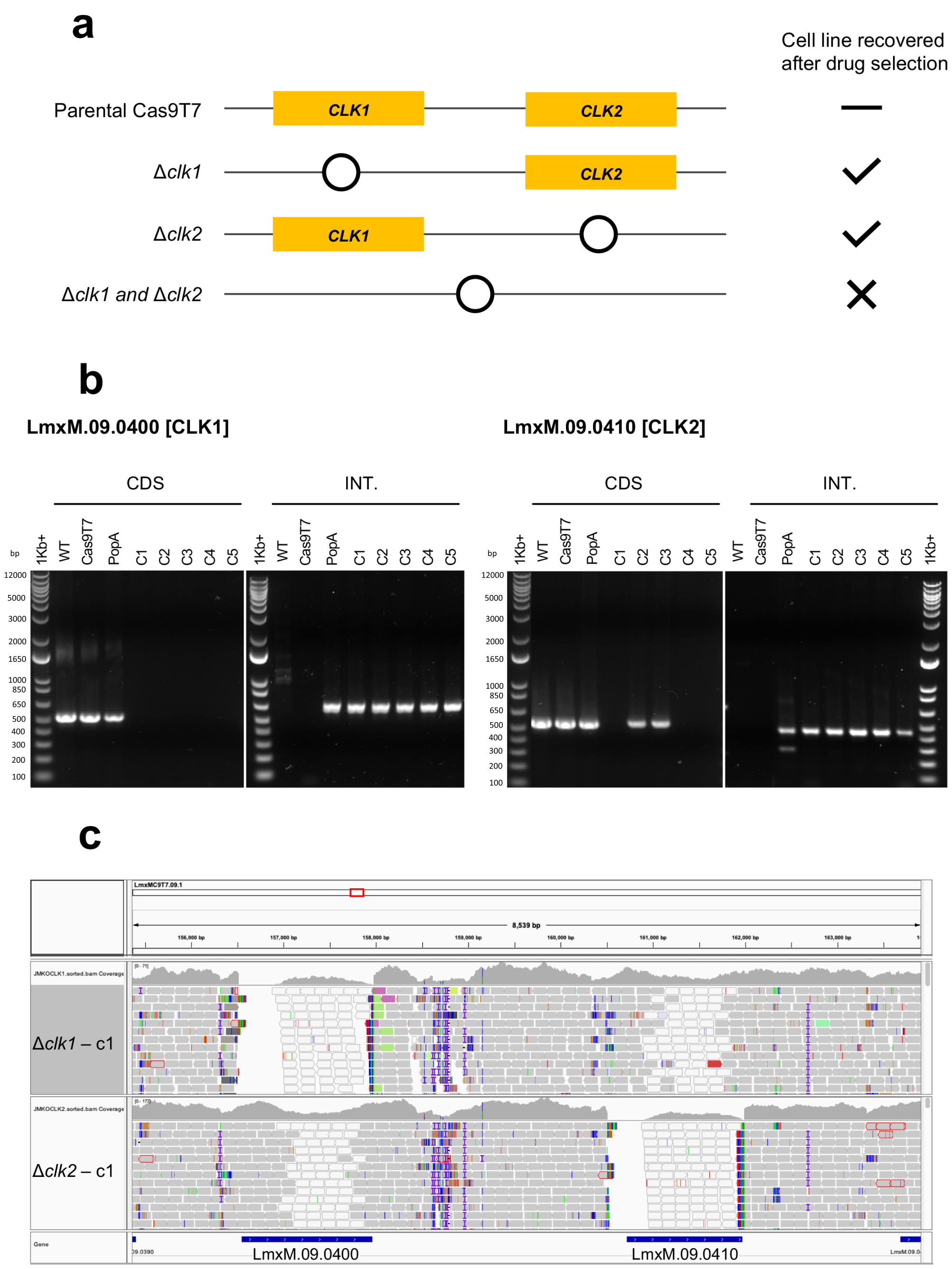

Supplementary Figure 2. **Redundancy of *CLK1* and *CLK2*.** **a** Gene deletion (circle) for *CLK1* (*LmxM.09.0400*) and *CLK2* (*LmxM.09.0410*). Attempts were made to delete *CLK1*, *CLK2* or both *CLK1* and *CLK2*. Successful deletions are indicated after antibiotic selection. **b** Diagnostic PCRs are shown for gene deletion mutants. WT; *L. mexicana* M379, T7 Cas9; *L. mexicana* cell line expressing T7-cas9 in populations (Pop) and clones (C). **c** Integrative genomics viewer of the illumina whole genome sequencing reads from Δ*clk1* and Δ*clk2* mutants aligned against the reference genome of the Cas9 T7 progenitor cell line generated by Nanopore sequence. Reads absent at the N-terminal end of the *CLK1* and *CLK2* indicate a gene deletion mutant as the remainder of the coding sequences are identical between both genes. The multiple alignments are indicated by the coloured white reads (mapping quality zero).

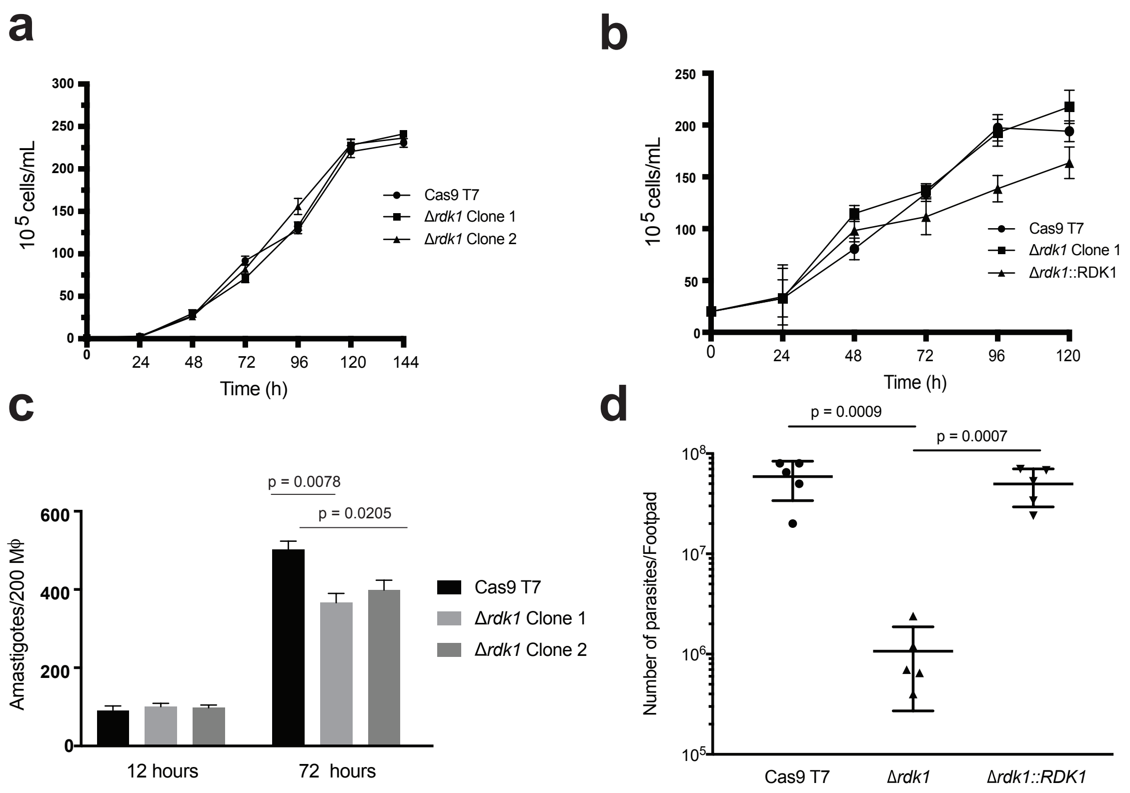

Supplementary Figure 3. **Characterisation of *RDK1*** **a** Proliferation of promastigotes comparing *L. mexicana* Cas9 T7 progenitor line and Δ*rdk1* clones 1 and 2. Values are mean +/- S.E., n=3 biologically independent samples. **b** Proliferation of axenic amastigotes comparing *L. mexicana* Cas9 T7 progenitor line, Δ*rdk1* and Δ*rdk1::RDK1*. Values are mean +/- S.E., n=3 biologically independent samples. **c** Bone-marrow macrophage infections using purified metacyclic promastigotes of *L. mexicana* Cas9 T7 progenitor line and Δ*rdk1* clones 1 and 2 analysed for total number of amastigotes infecting 200 macrophages (MΦ) at early (12 h) and late (72 h) time points in n=2 biologically independent samples, each with n=2 technical replicates. **d** Parasite burden determined by limiting dilution of 5 BALB/c footpad lesions infected with *L. mexicana* Cas9 T7 progenitor line, Δ*rdk1* mutant and an add-back Δ*rdk1::RDK1* for six weeks. Statistical analysis performed with two-tailed unpaired student *t*-test.

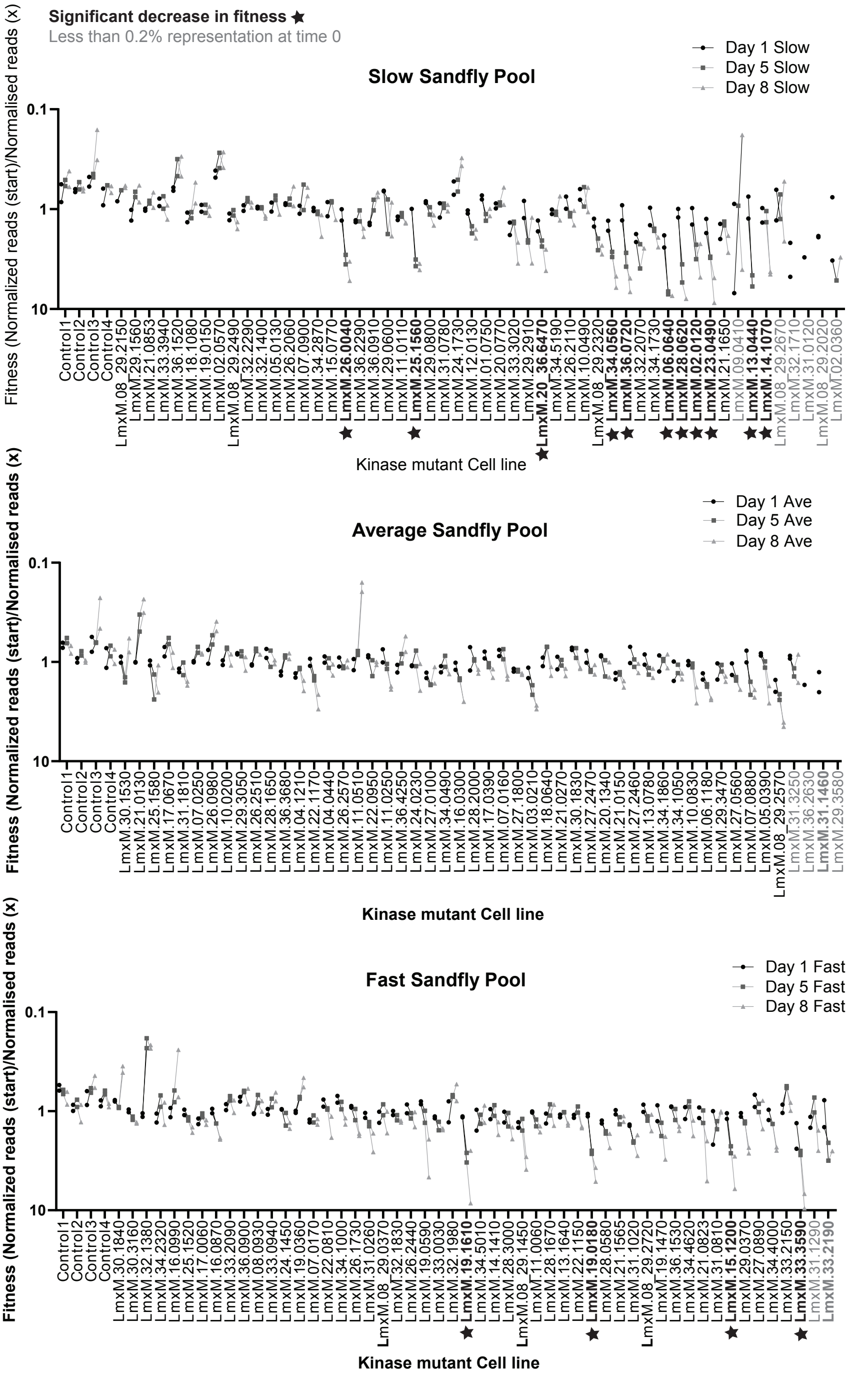

Supplementary Figure 4. **Fitness plots for protein kinase mutants during sand fly colonisation**. Data are shown for day 1 (Black), day 5 (Dark grey) and day 8 (light grey). Top plot shows mutants included in the slow pool, middle plot indicates average growth pool and the bottom plot indicates fast growth pool. Fitness was calculated using normalised reads at each time point against the normalised reads at time point 0. Mutants contributing to less than 0.2% of the reads on the day of the infected blood meal are highlighted in grey and were excluded from the loss of fitness analyses. Mutants showing a significant loss of fitness are indicated with a *. Values are mean +/- S.E., n=2 biologically independent samples. Corresponding raw data can be found in Supplementary Data 7.

a

Accession	Gene Name	Mean Flagella: Body ratio (n=50)	S.D. of Flagella: Body ratio	Mean cell body width (µm)(n = 50)	S.D. of cell body width	Phenotype	Exp. identified	Significant LOF in sand fly?
Cas9T7		1.023	0.302	1.978	0.329			No
LmxM.28.0620	*ULK4*	0.218	0.109	1.875	0.427	Short flagellum	Pool 1 and 2	Yes
LmxM.13.0440	*STK36*	0.061	0.027	2.287	0.347	Short flagellum	Pool 1 and 2	Yes
LmxM.29.0600		0.327	0.126	2.36	0.337	Short flagellum	Pool 1 and 2	No
LmxM.07.0900		1.182	0.380	3.276	0.746	Paralysed	Pool 1 and 2	No
LmxM.02.0570		0.316	0.296	3.472	0.428	Short flagellum	Pool 1 and 2	No
LmxM.36.1520		0.914	0.378	4.838	0.779	Uncoordinated	Pool 1	No
LmxM.29.1000		1.901	0.490	2.249	0.421	Long flagellum, paralysed	Pool1	Not in Pool 2

b

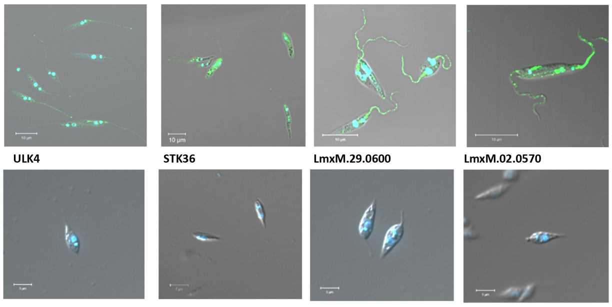

c

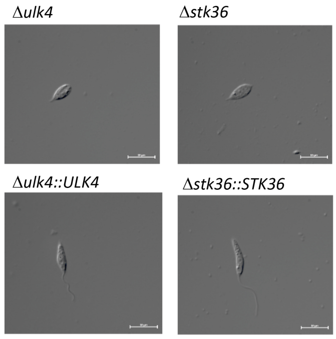

Supplementary Figure 5. **Protein kinases involved in flagellum function**. **a** Phenotypic analysis measurements for protein kinase mutants important for motility. **b** Additional images supporting the exempla protein kinase localisations in Fig. 6d and short flagellum null mutants in Fig. 6b. **c** Phase images of Δ*ulk4* and Δ*stk36* mutants (top) and Δ*ulk4::ULK4 and* Δ*stk36:STK36* add-back mutants (bottom). Images were taken from at least 3 field-of-views/20 cells.

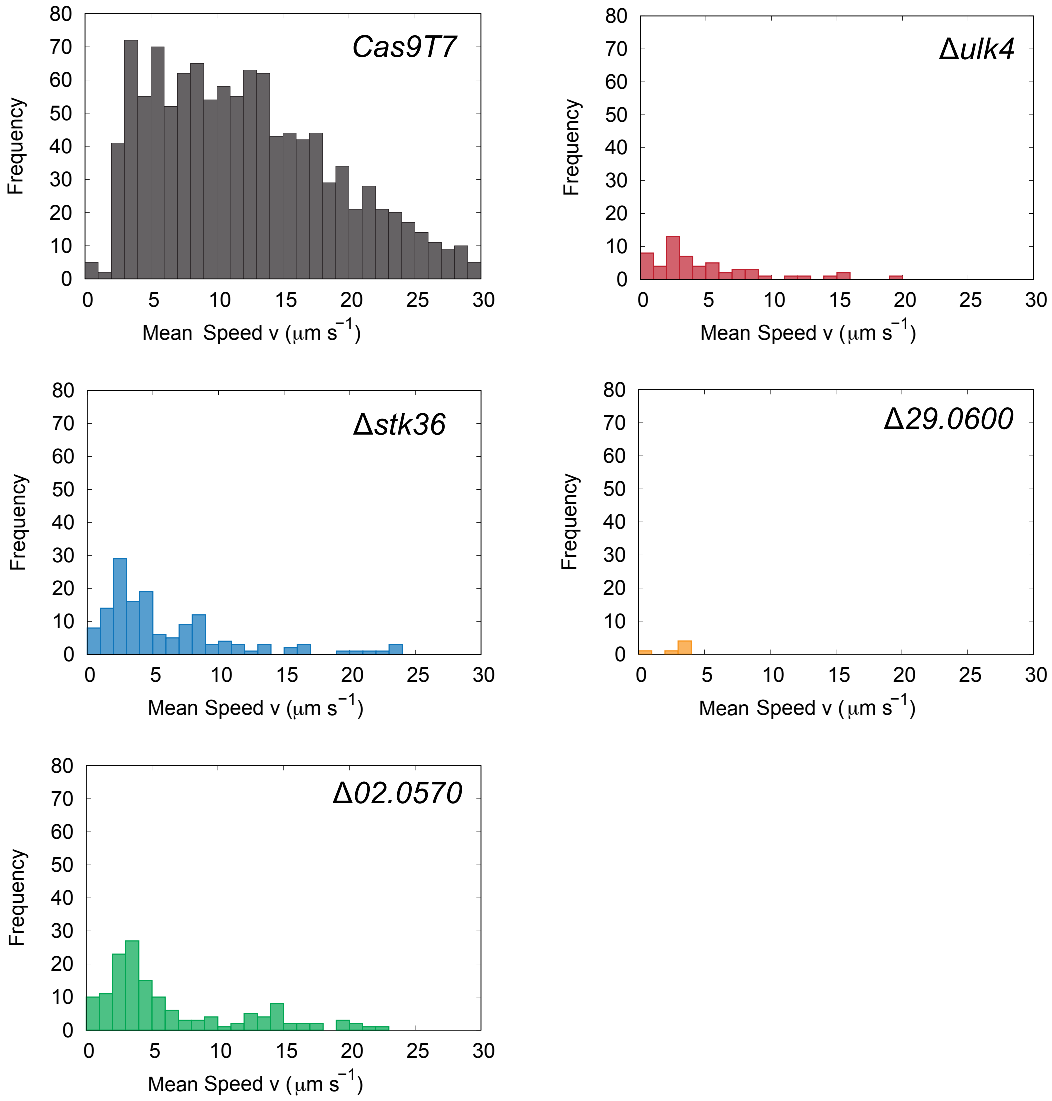

Supplementary Figure 6. **Speed histograms for protein kinase mutants**. These panels show the distribution of swimming speeds in the parental line (Cas9T7, n=1149 cells) and four protein kinase mutants: Δ*ulk4* (n=57 cells), Δ*stk36* (n=144 cells), Δ*29.0600* (n=6 cells), Δ*02.0570* (n=145 cells). Data points represent results from individual technical replicates. The instantaneous swimming speed of each cell was averaged across the duration of its swimming track to produce one count in the histograms.

Supplementary Table 1 **Overview of data on required/dispensable and primary localisation.**

Group/Family	Name	Gene ID	Required/ Dispensable	Primary Localisation
AGC	RAC1	LmxM.15.1550	Dispensable	mitochondrion
AGC	AEK1	LmxM.25.2340	required	cytoplasm
AGC	ZFK	LmxM.28.1670	Dispensable	lysosome
AGC	RAC2	LmxM.29.0800	Dispensable	cytoplasm
AGC		LmxM.29.1000	required	cytoplasm
AGC		LmxM.30.1530	Dispensable	cytoplasm
AGC/NDR		LmxM.06.1180	Dispensable	cytoplasm
AGC/PKA	PKAC3	LmxM.18.1080	Dispensable	pellicular membrane
AGC/PKA	PKAC2	LmxM.34.3960	Dispensable	pellicular membrane
AGC/PKA	PKAC1	LmxM.34.4010	required	nucleus
AGC/RSK		LmxM.03.0780	required	cytoplasm
CAMK		LmxM.27.2460	Dispensable	basal body
CAMK		LmxM.05.0130	Dispensable	nucleus
CAMK	MPKK3	LmxM.17.0060	Dispensable	cytoplasm
CAMK	AKB1	LmxM.19.0140	Dispensable	basal body
CAMK	SOS2	LmxM.22.0810	Dispensable	basal body
CAMK		LmxM.26.2510	Dispensable	cytoplasm
CAMK		LmxM.27.2470	Dispensable	basal body
CAMK	LDK	LmxM.28.2000	Dispensable	lipid droplet
CAMK		LmxM.34.1050	Dispensable	cytoplasm
CAMK/CAMKL		LmxM.07.0900	Dispensable	basal body
CAMK/CAMKL		LmxM.18.0640	Dispensable	basal body
CAMK/CAMKL		LmxM.24.0230	Dispensable	nucleus
CAMK/CAMKL	AMPKα	LmxM.08_29.2020	Dispensable	cytoplasm
CAMK/CAMKL		LmxM.32.1710	Dispensable	cytoplasm
CAMK/CAMKL		LmxM.34.0490	Dispensable	cytoplasm
CAMK/CAMKL	SNF1	LmxM.36.0900	Dispensable	not attempted
CAMK/CAMKL		LmxM.21.0150	Dispensable	flagellar pocket
CAMK/CAMKL		LmxM.04.0650	required	cytoplasm
CAMK/CAMKL		LmxM.19.1470	Dispensable	cytoplasm
CAMK/CAMKL		LmxM.22.1170	Dispensable	basal body
CAMK	AMPKβ	LmxM.23.0490	Dispensable	basal body
CAMK	AMPKγ	LmxM.34.0760	required	cytoplasm
CK1/CK1	CK1.4	LmxM.27.1780	required	flagellar pocket
CK1/CK1	CK1.3	LmxM.04.1210	Dispensable	cytoplasm
CK1/CK1		LmxM.25.1580	Dispensable	flagellum
CK1/CK1		LmxM.29.3470	Dispensable	cytoplasm

CK1/CK1	CK1.1	LmxM.34.1000	Dispensable	cytoplasm
CK1/CK1	CK1.2	LmxM.34.1010	required	cytoplasm
CK1/TTBK		LmxM.33.3020	Dispensable	flagellar pocket
CMGC		LmxM.24.0670	required	nucleus
CMGC	MPK15	LmxM.32.2070	Dispensable	cytoplasm
CMGC/CDK	CRK7	LmxM.26.0040	Dispensable	cytoplasm
CMGC/CDK	CRK2	LmxM.05.0550	required	cytoplasm
CMGC/CDK	CRK12	LmxM.09.0310	required	nucleus
CMGC/CDK	CRK8	LmxM.11.0110	Dispensable	nucleus
CMGC/CDK	CRK4	LmxM.16.0990	Dispensable	cytoplasm
CMGC/CDK	CRK1	LmxM.21.1080	required	mitochondrion
CMGC/CDK	CRK6	LmxM.27.0560	Dispensable	cytoplasm
CMGC/CDK	CRK9	LmxM.27.1940	required	nucleus
CMGC/CDK	CRK10	LmxM.08_29.2150	Dispensable	nucleus
CMGC/CDK	CRK11	LmxM.29.1780	required	nucleus
CMGC/CDK	CRK3	LmxM.36.0550	required	cytoplasm
CMGC/CLK	CLK1/KKT10	LmxM.09.0400	Dispensable	nucleus
CMGC/CLK	CLK2/KKT19	LmxM.09.0410	Dispensable	nucleus
CMGC		LmxM.25.1560	Dispensable	cytoplasm
CMGC/DYRK		LmxM.27.1800	Dispensable	endoplasmic reticulum
CMGC/DYRK		LmxM.14.0830	required	basal body
CMGC/DYRK		LmxM.14.1070	Dispensable	basal body
CMGC/DYRK	DYRK1	LmxM.15.0180	required	lysosome
CMGC/DYRK		LmxM.19.0360	Dispensable	cytoplasm
CMGC	PK4	LmxM.21.1650	Dispensable	cytoplasm
CMGC/DYRK		LmxM.34.1860	Dispensable	flagellar pocket
CMGC/DYRK	DYRK2	LmxM.32.1830	Dispensable	cytoplasm
CMGC/GSK		LmxM.36.4250	Dispensable	no signal
CMGC/GSK	GSK3	LmxM.18.0270	required	pellicular membrane
CMGC/MAPK	GSKA	LmxM.22.0490	required	no signal
CMGC/MAPK		LmxM.03.0210	Dispensable	cytoplasm
CMGC/MAPK	MPK10	LmxM.10.0200	Dispensable	cytoplasm
CMGC/MAPK	MPK3	LmxM.10.0490	Dispensable	cytoplasm
CMGC/MAPK		LmxM.13.0780	Dispensable	cytoplasm
CMGC/MAPK	MPK7	LmxM.13.1640	Dispensable	cytoplasm
CMGC/MAPK	MPK4	LmxM.19.1440	required	lysosome
CMGC/MAPK	MPK12	LmxM.29.0370	Dispensable	cytoplasm
CMGC/MAPK	MPK5	LmxM.29.2910	Dispensable	pellicular membrane
CMGC/MAPK	MPK11	LmxM.32.1380	Dispensable	cytoplasm
CMGC/MAPK	MPK2	LmxM.36.0720	Dispensable	cytoplasm
CMGC/CDKL	MPK1	LmxM.20_36.6470	Dispensable	flagellum
CMGC/CDKL	MPK8	LmxM.28.0580	Dispensable	flagellum

CMGC/RCK	MPK6	LmxM.31.3250	Dispensable	flagellum
CMGC/RCK	MPK9	LmxM.19.0180	Dispensable	cytoplasm
CMGC/RCK-MAPK	MPK14	LmxM.27.0100	Dispensable	flagellum
CMGC/SRPK	MPK13	LmxM.34.5010	Dispensable	basal body
CMGC/SRPK		LmxM.26.0980	Dispensable	cytoplasm
CMGC/DYRK		LmxM.29.3580	Dispensable	cytoplasm
CMGC/SRPK		LmxM.17.0670	Dispensable	basal body
CMGC/CDK		LmxM.01.0750	Dispensable	cytoplasm
CMGC		LmxM.12.0130	Dispensable	cytoplasm
STE	MKK4	LmxM.24.2320	required	endomembrane
STE	MKK1	LmxM.08_29.2320	Dispensable	cytoplasm
STE		LmxM.08.1228	Dispensable	cytoplasm
STE		LmxM.15.1200	Dispensable	cytoplasm
STE		LmxM.17.0390	Dispensable	basal body
STE		LmxM.20.0770	Dispensable	pellicular membrane
STE		LmxM.21.0270	Dispensable	cytoplasm
STE		LmxM.25.1990	required	endomembrane
STE		LmxM.31.1020	Dispensable	cytoplasm
STE		LmxM.34.3170	required	cytoplasm
STE		LmxM.04.0440	Dispensable	flagellar pocket
STE		LmxM.05.0390	Dispensable	basal body
STE		LmxM.06.0640	Dispensable	lysosome
STE		LmxM.07.0690	required	cytoplasm
STE		LmxM.07.0880	Dispensable	cytoplasm
STE		LmxM.14.1300	required	flagellum
STE		LmxM.17.0490	required	flagellum
STE		LmxM.19.0150	Dispensable	cytoplasm
STE		LmxM.21.0130	Dispensable	endomembrane
STE		LmxM.24.1450	Dispensable	cytoplasm
STE		LmxM.26.1730	Dispensable	lysosome
STE		LmxM.27.1370	required	endomembrane
STE		LmxM.29.0600	Dispensable	flagellum
STE		LmxM.29.3050	Dispensable	cytoplasm
STE	MRK1	LmxM.31.0120	Dispensable	cytoplasm
STE/STE11		LmxM.31.0780	Dispensable	basal body
STE	RDK1	LmxM.31.0810	Dispensable	cytoplasm
STE		LmxM.32.1400	Dispensable	nucleus
STE		LmxM.32.2290	Dispensable	lysosome
STE		LmxM.36.0910	Dispensable	lysosome
STE		LmxM.16.0300	Dispensable	basal body
STE		LmxM.07.0250	Dispensable	cytoplasm
STE		LmxM.36.0860	required	cytoplasm

STE		LmxM.19.1610	Dispensable	cytoplasm
STE		LmxM.33.2090	Dispensable	cytoplasm
STE/STE11		LmxM.34.4000	Dispensable	cytoplasm
STE/STE11		LmxM.36.3680	Dispensable	endomembrane
STE/STE11		LmxM.30.1830	Dispensable	endomembrane
STE		LmxM.30.1840	Dispensable	cytoplasm
Other/NEK		LmxM.02.0290	required	cytoplasm
Other/NEK		LmxM.07.0160	Dispensable	endomembrane
Other/NEK		LmxM.07.0170	Dispensable	basal body
Other/NEK		LmxM.08.0930	Dispensable	flagellum
Other/NEK		LmxM.14.1410	Dispensable	flagellum
Other/NEK		LmxM.21.0853	Dispensable	cytoplasm
Other/NEK		LmxM.21.1565	Dispensable	basal body
Other/NEK		LmxM.22.0950	Dispensable	cytoplasm
Other/NEK		LmxM.26.2570	Dispensable	basal body
Other/NEK		LmxM.28.3000	Dispensable	cytoplasm
Other/NEK		LmxM.08_29.2570	Dispensable	cytoplasm
Other/NEK		LmxM.08_29.2670	Dispensable	cytoplasm
Other/NEK		LmxM.29.2130	Dispensable	basal body
Other/NEK	RDK2	LmxM.30.2960	required	cytoplasm
Other/NEK		LmxM.30.3160	Dispensable	cytoplasm
Other/NEK		LmxM.31.0260	Dispensable	flagellum
Other/NEK		LmxM.31.1810	Dispensable	cytoplasm
Other/NEK		LmxM.32.1980	Dispensable	endomembrane
Other/NEK		LmxM.34.5190	Dispensable	cytoplasm
Other/NEK		LmxM.36.1520	Dispensable	lysosome
Other/NEK		LmxM.36.1530	Dispensable	cytoplasm
Other/NEK		LmxM.36.2290	Dispensable	lysosome
Orphan		LmxM.02.0570	Dispensable	flagellum
Orphan		LmxM.03.0350	Dispensable	cytoplasm
Orphan		LmxM.10.0830	Dispensable	nucleus
Orphan		LmxM.11.0510	Dispensable	lysosome
Orphan		LmxM.16.0870	Dispensable	cytoplasm
Orphan		LmxM.25.1520	Dispensable	endomembrane
Orphan		LmxM.26.2110	required	lysosome
Orphan		LmxM.08_29.2490	Dispensable	cytoplasmic organelles
Orphan	KKT3	LmxM.34.4050	required	nucleus
Orphan	KKT2	LmxM.36.5350	required	nucleus
Pseudo-Orphans		LmxM.34.4620	Dispensable	-
Pseudo-Orphans		LmxM.08.0660	Dispensable	-
Pseudo-Orphans		LmxM.26.2060	Dispensable	-
Pseudo-Orphans		LmxM.28.1650	Dispensable	-

Pseudo-Orphans		LmxM.31.1290	Dispensable	-
Pseudo-Orphans		LmxM.33.2190	Dispensable	-
Other/unique		LmxM.20.1330	required	endomembrane
Other/unique		LmxM.20.1340	Dispensable	cytoplasm
Other/unique		LmxM.08_29.0370	Dispensable	cytoplasm
Other/unique		LmxM.34.2870	Dispensable	cytoplasm
Other/AUR	AUK3	LmxM.26.2440	Dispensable	nucleus
Other/AUR	AUK1/AIRK	LmxM.28.0520	required	nucleus
Other/AUR	AUK2	LmxM.08_29.1330	required	cytoplasm
Other/CAMKK		LmxM.19.0590	Dispensable	cytoplasm
Other/CAMKK		LmxM.11.0250	Dispensable	cytoplasm
Other/CAMKK		LmxM.24.1730	Dispensable	cytoplasm
Other/CAMKK		LmxM.34.2320	Dispensable	flagellum
Other/CK2	CK2A1	LmxM.34.1730	Dispensable	cytoplasm
Other/CK2	CK2A2	LmxM.02.0360	Dispensable	nucleus
Other/NAK		LmxM.15.0770	Dispensable	cytoplasm
Other/NAK		LmxM.33.0030	Dispensable	cytoplasm
Other/PEK	EIF2AK1	LmxM.11.0060	Dispensable	cytoplasm
Other/PEK	EIF2AK3	LmxM.29.1560	Dispensable	cytoplasm
Other/PEK	EIF2AK2	LmxM.33.2150	Dispensable	cytoplasmic organelles
Other/PLK	PLK	LmxM.17.0790	required	basal body
Other/TLK	TLK	LmxM.30.2860	required	nucleus
Other/ULK	ULK4	LmxM.28.0620	Dispensable	basal body
Other/ULK	STK36	LmxM.13.0440	Dispensable	basal body
Other/VPS15		LmxM.28.1760	required	endomembrane
Other/WEE		LmxM.08_29.2720	Dispensable	cytoplasm
Other/WEE	WEE1	LmxM.33.0940	Dispensable	cytoplasm
Other/unique		LmxM.08.0530	required	endomembrane
Other/unique		LmxM.20.0960	required	endomembrane
Other/unique		LmxM.21.0823	Dispensable	lysosome
Other/unique		LmxM.36.2630	Dispensable	endomembrane
Other/unique		LmxM.22.1150	Dispensable	cytoplasm
Atypical	PIK	LmxM.08_29.1450	Dispensable	lysosome
Atypical	PI3K	LmxM.24.2010	required	cytoplasm
Atypical	PI4K	LmxM.33.3590	Dispensable	cytoplasm
Atypical	PI3,5K	LmxM.27.0890	Dispensable	cytoplasm
Atypical	PIPKalpha	LmxM.33.3090	Dispensable	cytoplasm
Atypical	PI4P5K	LmxM.34.0560	Dispensable	flagellar pocket
Atypical	TOR1	LmxM.36.6320	required	cytoplasm
Atypical	TOR2	LmxM.33.4530	required	cytoplasmic organelles
Atypical	TOR3	LmxM.33.3940	Dispensable	cytoplasm

Atypical	ATM	LmxM.02.0120	Dispensable	nucleus
Atypical	ATR	LmxM.31.1460	Dispensable	cytoplasm
