# Supplementary material for: Systematic functional analysis of Leishmania protein kinases identifies regulators of differentiation or survival
Source: Nat Commun. 2021 Feb 23;12:1244. doi: 10.1038/s41467-021-21360-8 (PMC7902614; doi:10.1038/s41467-021-21360-8)
Supplement: Supplementary file 3 — Description of Additional Supplementary Files [file 41467_2021_21360_MOESM3_ESM.pdf]

Description of Additional Supplementary Files

File Name: Supplementary Data 1

Description: Orthology of protein kinases in trypanosomatids, Crithidia fasciculata strain Cf-Cl, Endotrypanum monterogeii strain LV88, Leptomonas seymouri ATCC 30220, Blechomonas ayalai B08-376, Trypanosoma grayi ANR4, Trypanosoma cruzi, Trypanosoma vivax, Trypanosoma brucei, Paratrypanosoma confusum CUL13. Data from TritrypDB.

File Name: Supplementary Data 2

Description: Diagnostic PCRs to test for gene deletion mutants. The DNA prepared from populations (Pop) recovered after transfection was used to test for the presence/ or absence of the protein kinase coding sequence (CDS) and integration (INT) of the resistance cassette at the correct locus. Exemplar gel explained in Fig 2b. All primers used listed in Supplementary Data 3.

File Name: Supplementary Data 3.

Description: Primers used for gene deletion and predicted size of PCR products for CDS and integration.

File Name: Supplementary Data 4

Description: Primers used for endogenous tagging of protein kinases.

File Name: Supplementary Data 5

Description: Localisation of mNeonGreen tagged protein kinases. An example image is shown for each protein kinase in the G1 phase of the cell cycle (1K 1N). Additional images are included for protein kinases that are stage specific or for which Nand C- terminal tagging differ. Images are ordered by the protein kinase family. Scale bar: 2 µm. Descriptors are based on those described in Halliday, C. et al. Cellular landmarks of Trypanosoma brucei and Leishmania mexicana. Mol. Biochem. Parasitol. 230, 24– 36 (2019).

File Name: Supplementary Data 6.

Description: Localisation data overview. Summary of localisation data.

File Name: Supplementary Data 7

Description: Kinome wide library outputs. Tab 1: Axa_InMac_Mouse data Pool 1; bar-seq data for protein kinases required for differentiation and survival in culture and in the mammalian host. Tab 2: Sand fly data Pool 2; bar-seq data for protein kinases required for colonisation of the sand fly. Tab 3: Motility data Pool 1; bar-seq data for protein kinases required for motility. Tab 4: Motility data Pool 2; bar-seq data for protein kinases required for motility. Tab 5: Axa_InMac_Mouse data analysis; cluster analysis for data in Pool 1. Tab 6: Sandfly analysis; analysis of data from Pool 2. Tab 7: Motility analysis; protein kinases with significant loss of motility from Pool 1 and Pool 2 and cell phenotype measurements.

File Name: Supplementary Data 8

Description: Heat maps and cluster analysis for amastigotes. Data for the three experimental arms (EA1, 2 and 3 (see Fig 4a)) sorted via cluster analysis. Heat maps show % abundance of barcodes. Cluster trajectories plotted using the logged % of barcodes, normalised to time 0 (relative abundance (Log10)). a Axenic amastigote data clustered by 1 difference b Individual plots for axenic amastigote data. c Macrophage infection data clustered by 1 difference. d Individual plots for macrophage infection data (coloured according to 1 difference clustering). e Mouse footpad infection data clustered by 1 difference. f Individual plots for mouse footpad infection data (coloured according to 1 difference clustering). Procyclic promastigotes (PRO), axenic amastigotes (AXA), metacyclic promastigotes (META), infected macrophages (inMAC) and mouse footpads (FP).

File Name: Supplementary Movie 1

Description: Motility of protein kinase mutants. These movies show the effect of mutations on the flagellar beat of L. mexicana. The movies were acquired at a frame rate of 500 Hz using a phase-contrast imaging and a 60x magnification microscope objective. The scale is the same for all panels, and the movie is played back at 1/5 th of the true speed so the high-speed flagellar motion can be more easily seen.

File Name: Supplementary Software

Description: This includes Projection pursuit cluster analysis methodology and script and the Python script to count the occurrence of barcode sequences in FASTA files generated in the barseq experiments.
