# Supplementary material for: Systematic functional analysis of Leishmania protein kinases identifies regulators of differentiation or survival
Source: Nat Commun. 2021 Feb 23;12:1244. doi: 10.1038/s41467-021-21360-8 (PMC7902614; doi:10.1038/s41467-021-21360-8)

Supplementary Data 2. **Diagnostic PCRs to test for gene deletion mutants.**

AGC FAMILY

LmxM.15.1550

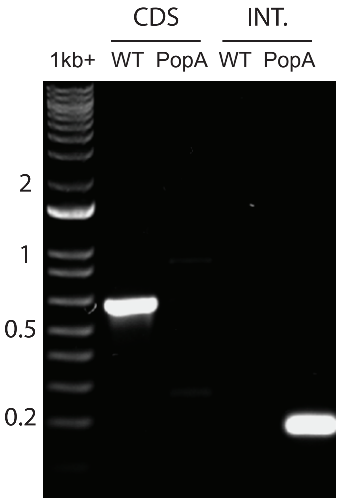

LmxM.25.2340

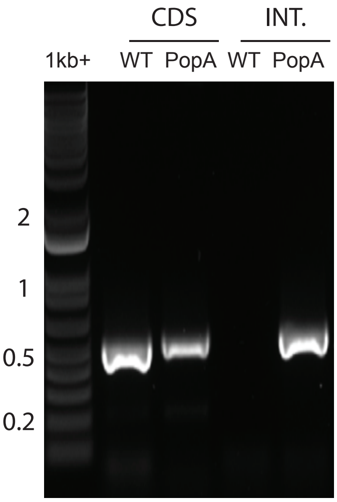

LmxM.28.1670

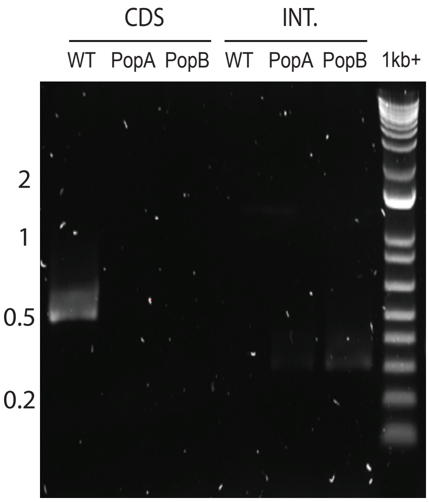

LmxM.29.0800

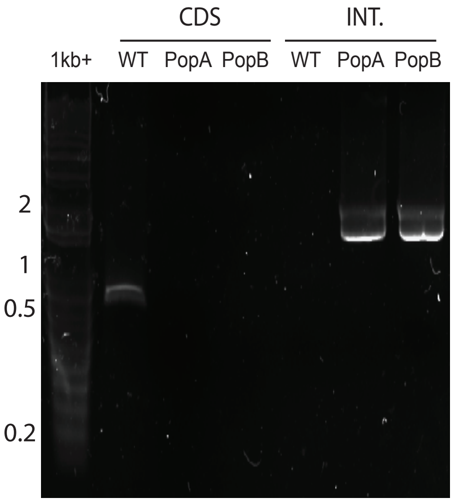

LmxM.29.1000

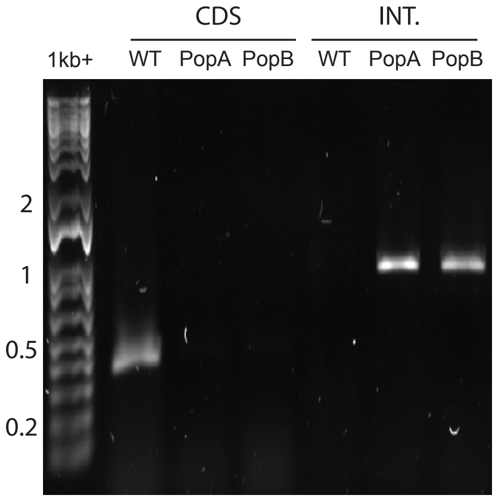

LmxM.30.1530

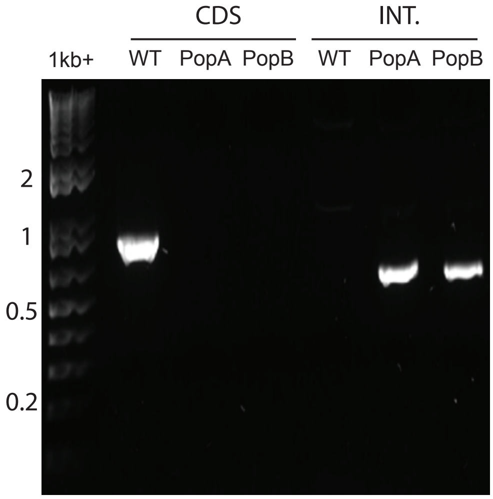

LmxM.06.1180

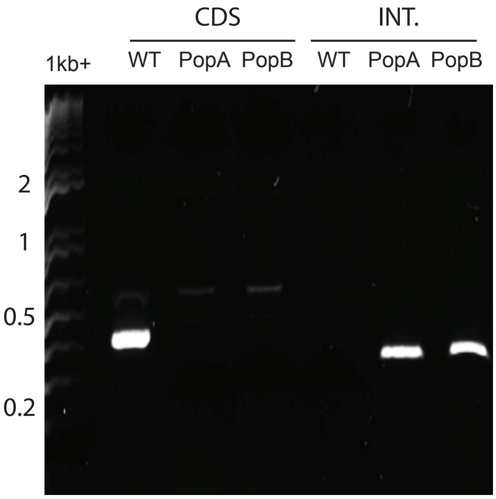

LmxM.18.1080

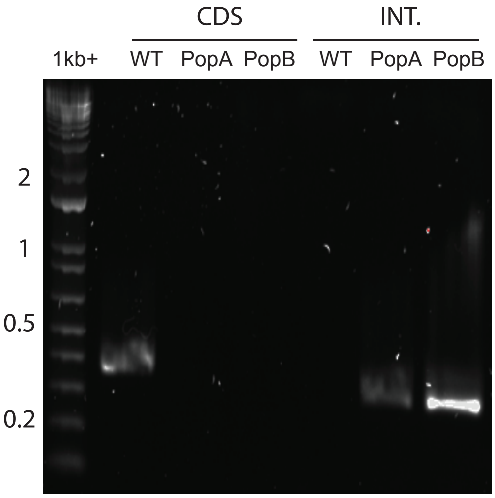

LmxM.34.3960

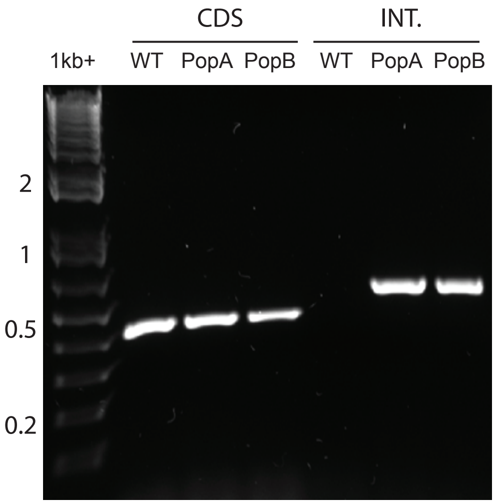

LmxM.34.4010

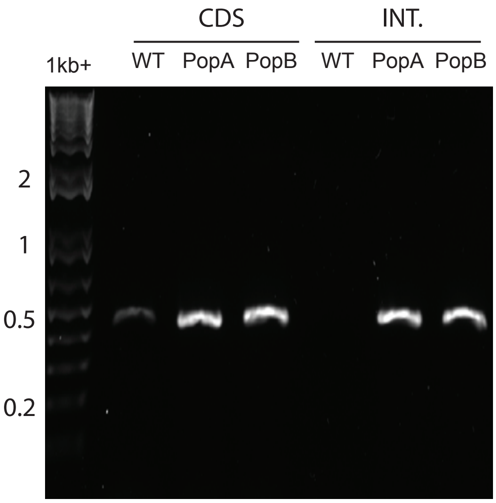

CAMK FAMILY

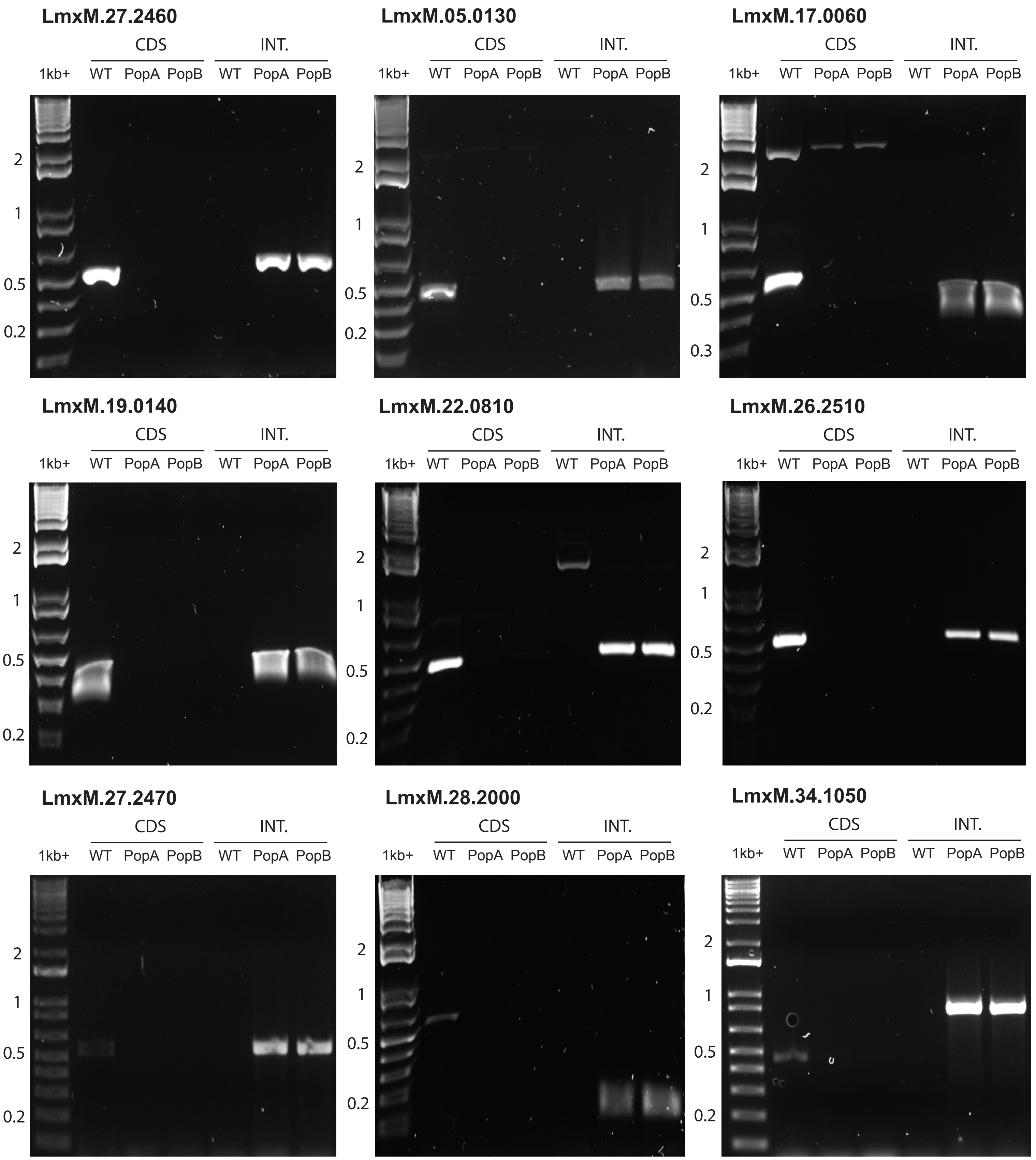
LmxM.27.2460
CDS
INT.
1kb+ WT PopA PopB WT PopA PopB
2
1
0.5
0.2
LmxM.05.0130
CDS
INT.
1kb+ WT PopA PopB WT PopA PopB
2
1
0.5
0.2
LmxM.17.0060
CDS
INT.
1kb+ WT PopA PopB WT PopA PopB
2
1
0.5
0.3
LmxM.19.0140
CDS
INT.
1kb+ WT PopA PopB WT PopA PopB
2
1
0.5
0.2
LmxM.22.0810
CDS
INT.
1kb+ WT PopA PopB WT PopA PopB
2
1
0.5
0.2
LmxM.26.2510
CDS
INT.
1kb+ WT PopA PopB WT PopA PopB
2
1
0.5
0.2
LmxM.27.2470
CDS
INT.
1kb+ WT PopA PopB WT PopA PopB
2
1
0.5
0.2
LmxM.28.2000
CDS
INT.
1kb+ WT PopA PopB WT PopA PopB
2
1
0.5
0.2
LmxM.34.1050
CDS
INT.
1kb+ WT PopA PopB WT PopA PopB
2
1
0.5
0.2

CAMK FAMILY

LmxM.07.0900

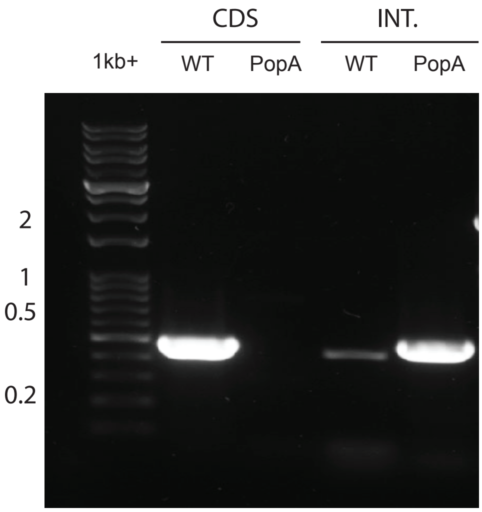

LmxM.18.0640

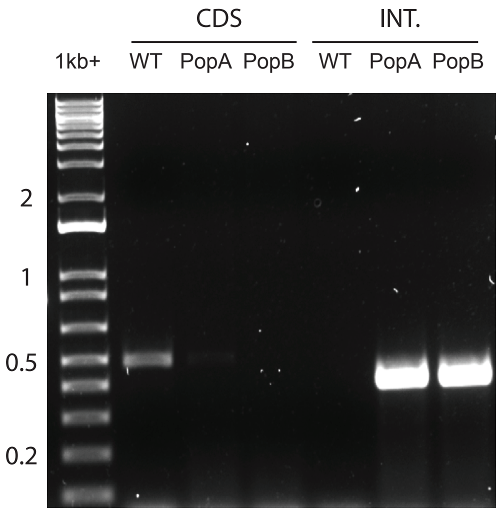

LmxM.24.0230

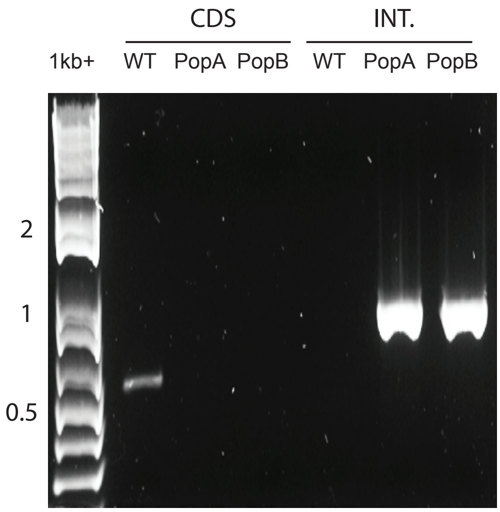

LmxM.08_29.2020

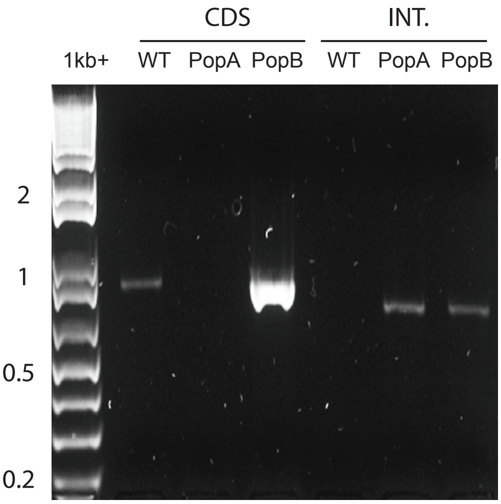

LmxM.32.1710

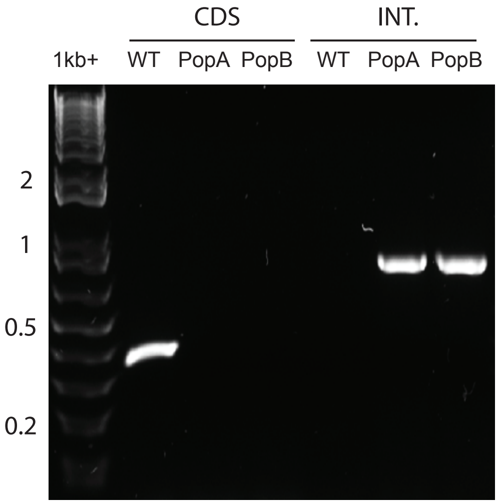

LmxM.34.0490

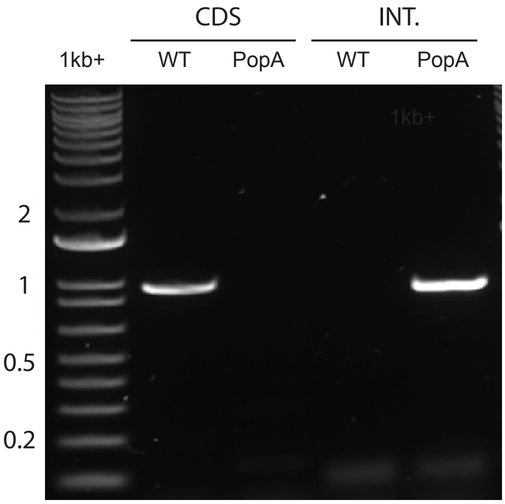

LmxM.36.0900

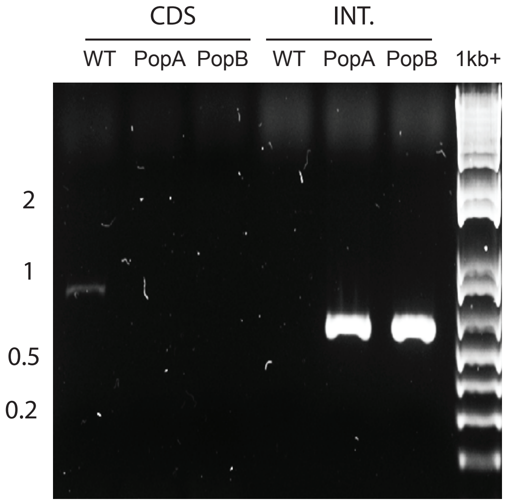

LmxM.21.0150

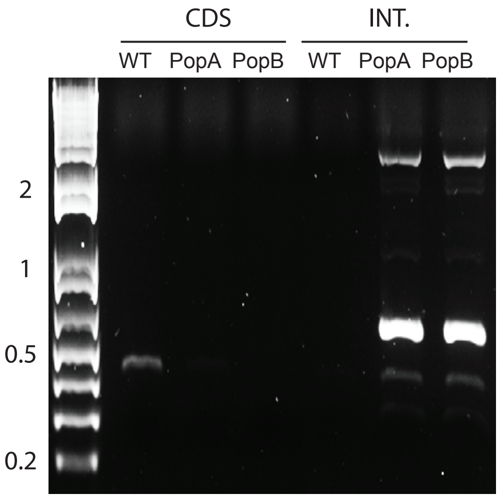

LmxM.22.1170

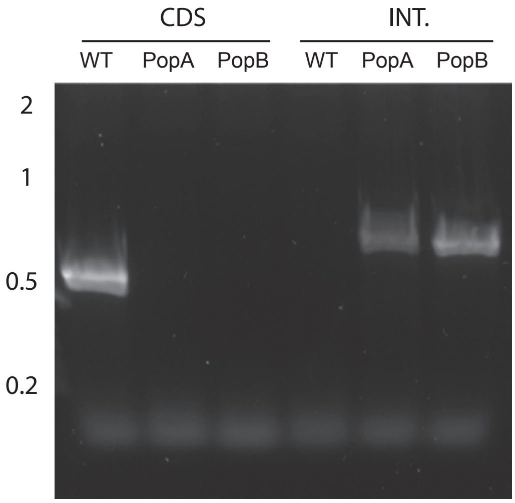

CAMK FAMILY

LmxM.19.1470

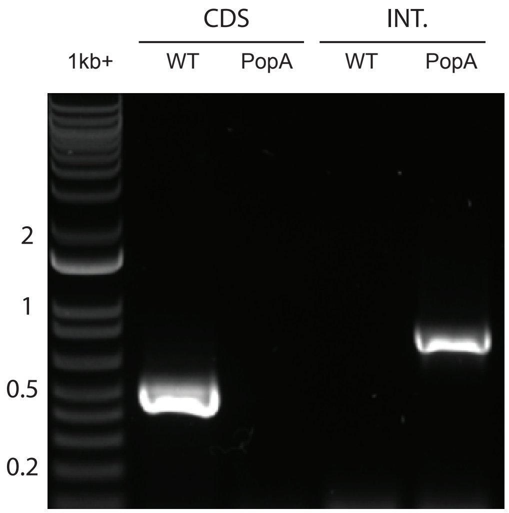

LmxM.23.0490

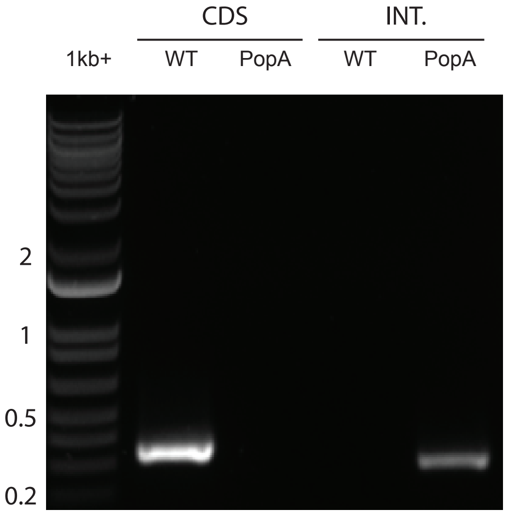

CK1 FAMILY

LmxM.04.1210

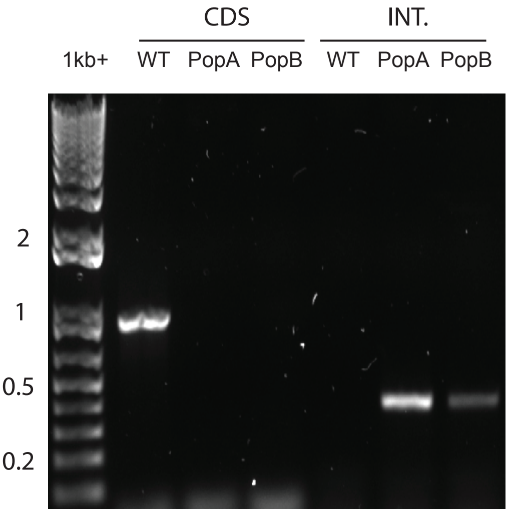

LmxM.25.1580

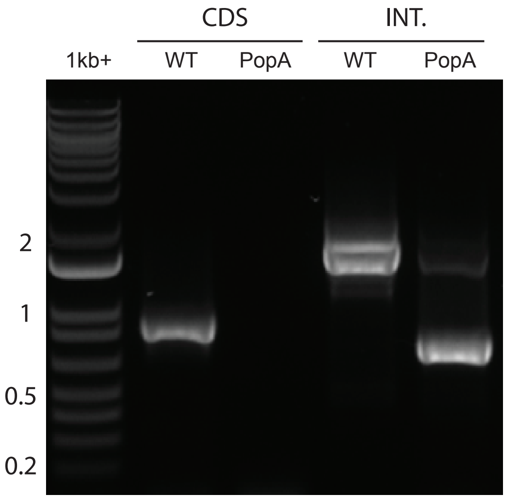

LmxM.29.3470

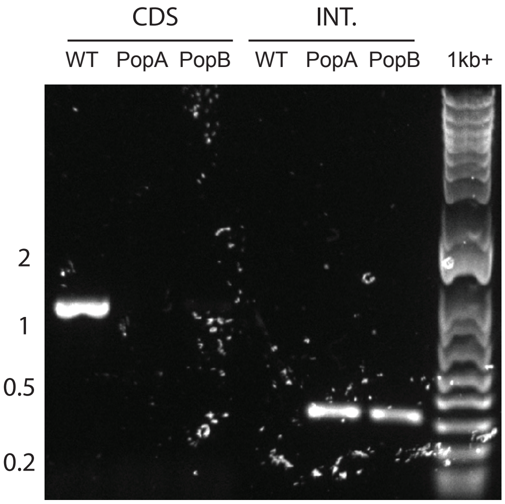

LmxM.34.1000

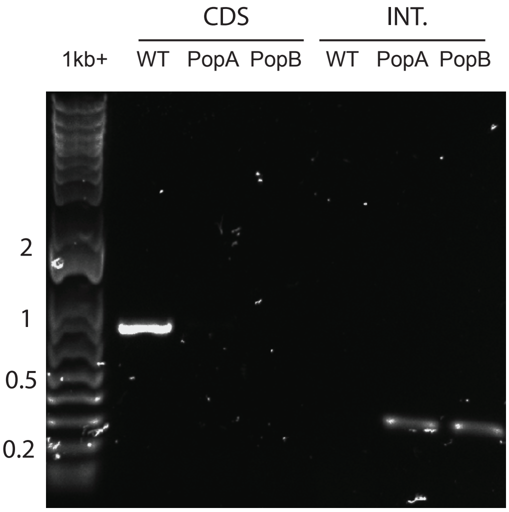

LmxM.33.3020

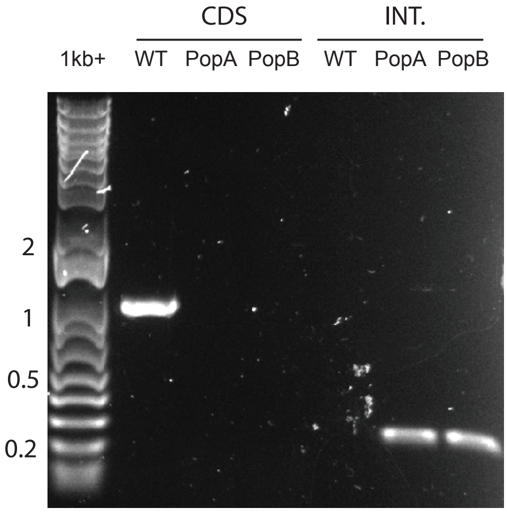

CMGC FAMILY

LmxM.32.2070

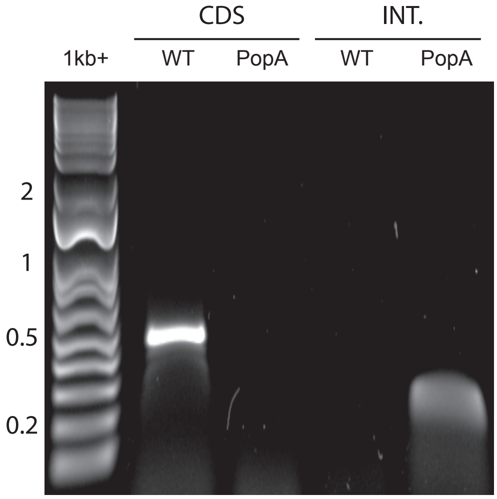

LmxM.26.0040

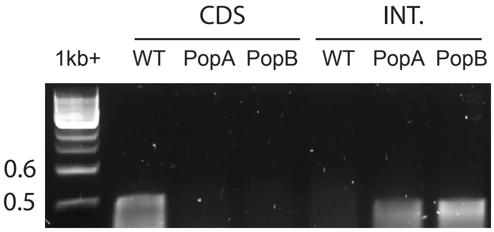

LmxM.16.0990

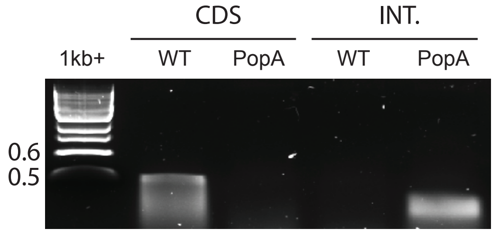

LmxM.11.0110

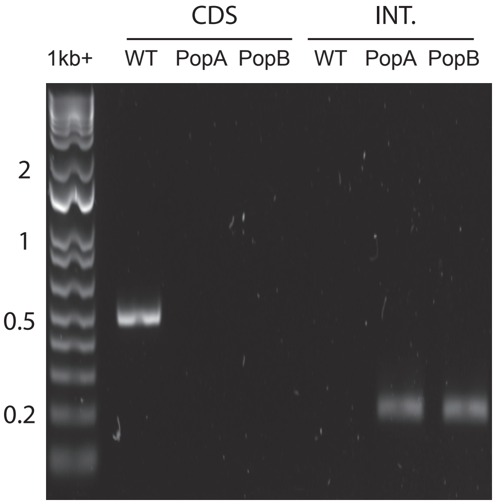

LmxM.27.0560

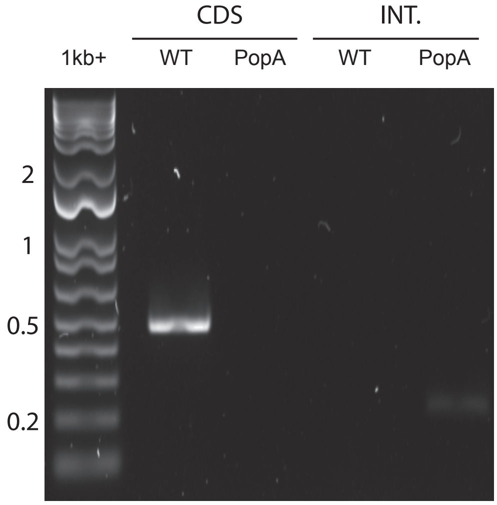

LmxM.08_29.2150

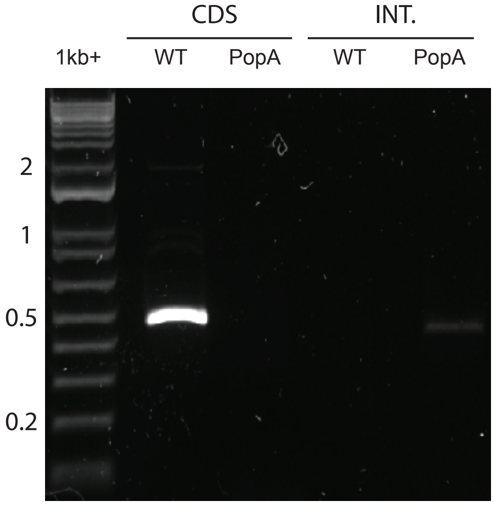

LmxM.25.1560

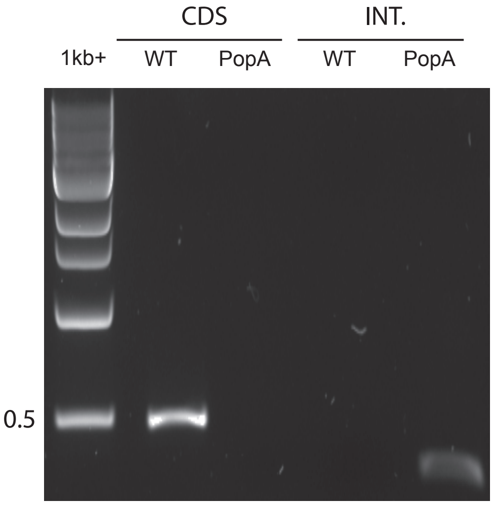

LmxM.27.1800

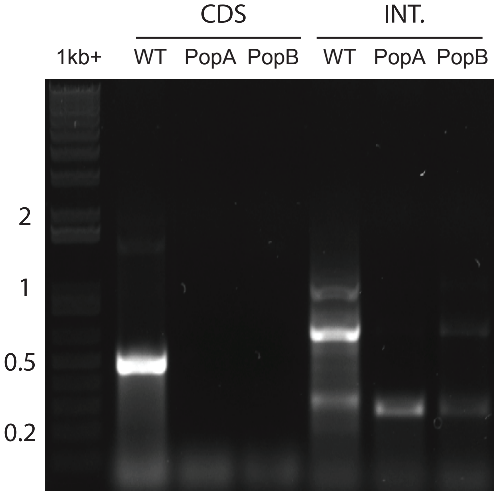

LmxM.14.1070

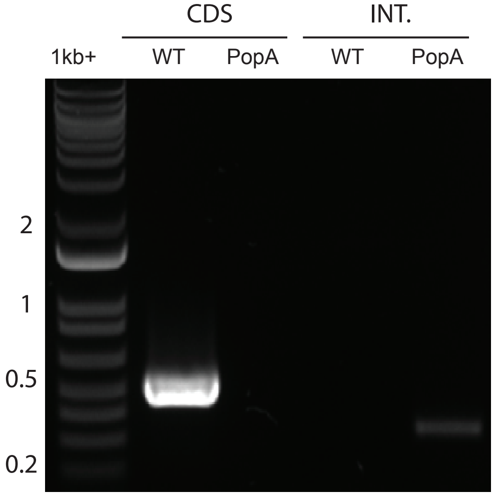

LmxM.19.0360

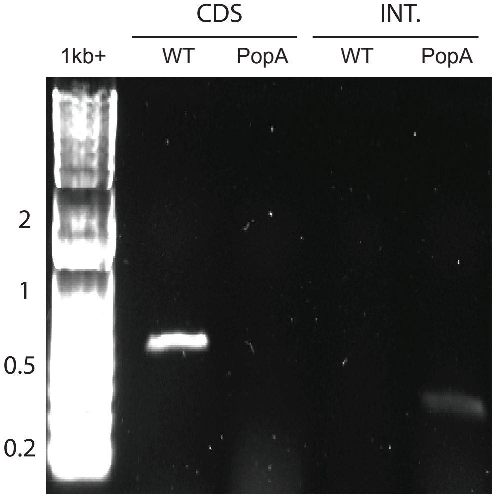

CMGC FAMILY

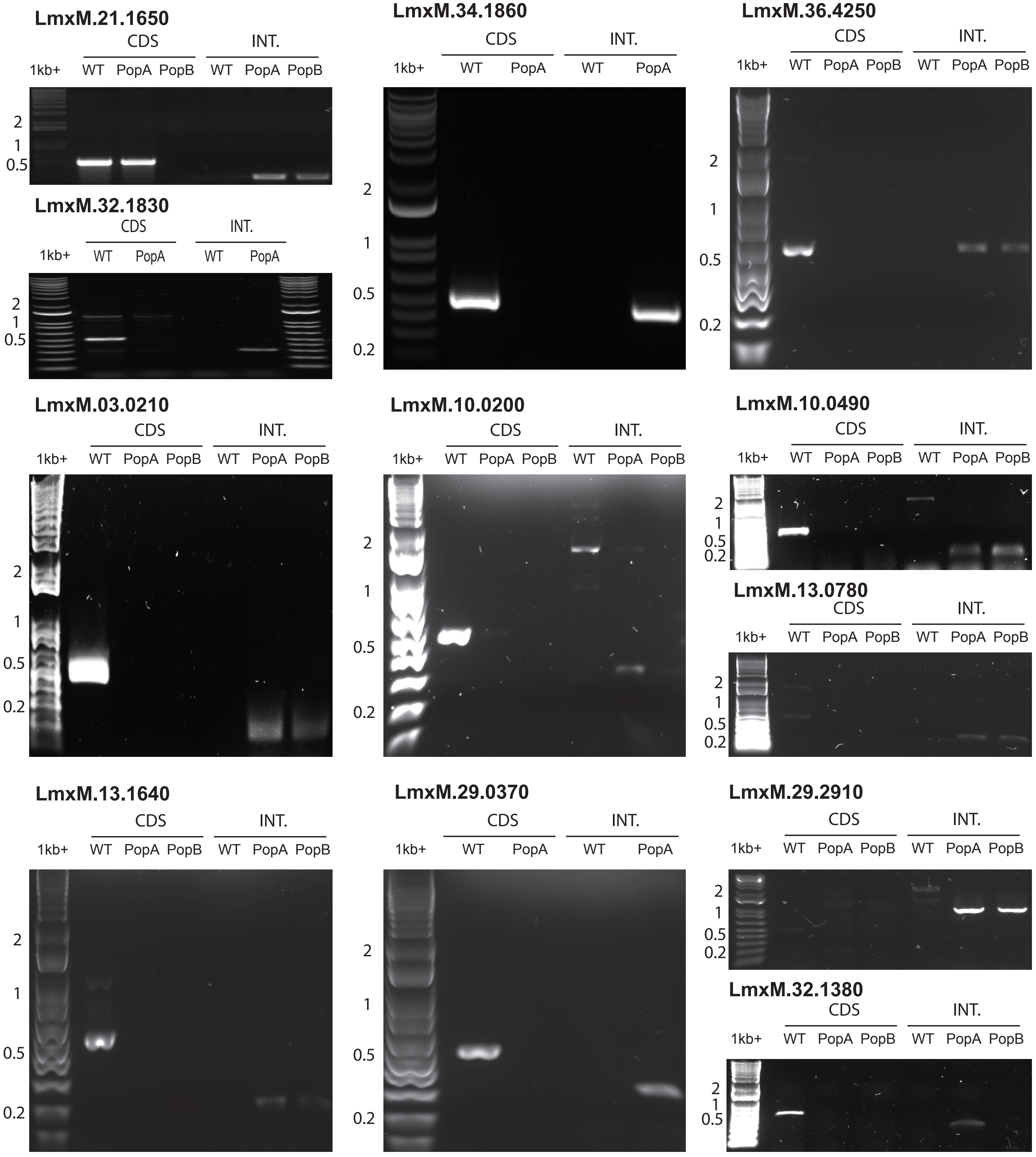
LmxM.21.1650
CDS
INT.
1kb+ WT PopA PopB WT PopA PopB
2
1
0.5
LmxM.34.1860
CDS
INT.
1kb+ WT PopA WT PopA
2
1
0.5
0.2
LmxM.36.4250
CDS
INT.
1kb+ WT PopA PopB WT PopA PopB
2
1
0.5
0.2
LmxM.32.1830
CDS
INT.
1kb+ WT PopA WT PopA
2
1
0.5
LmxM.03.0210
CDS
INT.
1kb+ WT PopA PopB WT PopA PopB
2
1
0.5
0.2
LmxM.10.0200
CDS
INT.
1kb+ WT PopA PopB WT PopA PopB
2
1
0.5
0.2
LmxM.10.0490
CDS
INT.
1kb+ WT PopA PopB WT PopA PopB
2
1
0.5
0.2
LmxM.13.0780
CDS
INT.
1kb+ WT PopA PopB WT PopA PopB
2
1
0.5
0.2
LmxM.13.1640
CDS
INT.
1kb+ WT PopA PopB WT PopA PopB
2
1
0.5
0.2
LmxM.29.0370
CDS
INT.
1kb+ WT PopA WT PopA
2
1
0.5
0.2
LmxM.29.2910
CDS
INT.
1kb+ WT PopA PopB WT PopA PopB
2
1
0.5
0.2
LmxM.32.1380
CDS
INT.
1kb+ WT PopA PopB WT PopA PopB
2
1
0.5

CMGC FAMILY

LmxM.20_36.6470

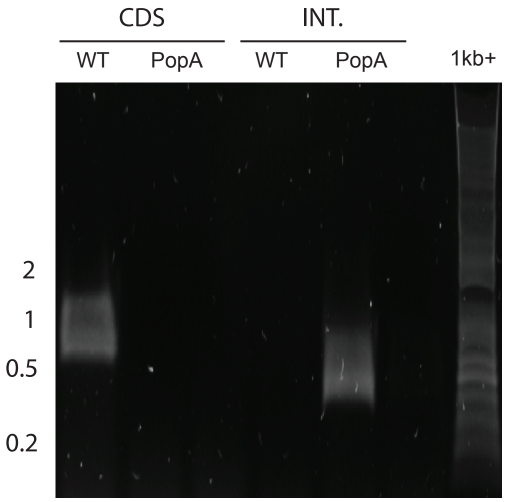

LmxM.28.0580

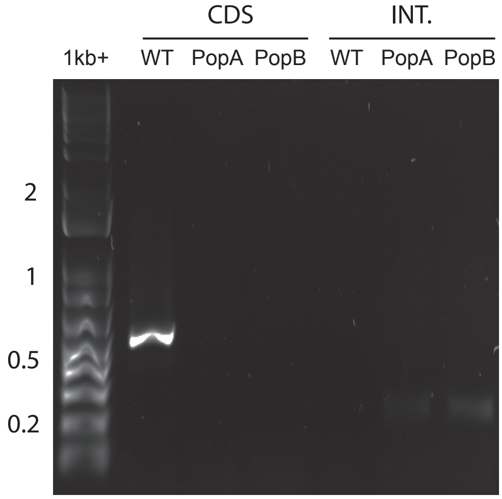

LmxM.31.3250

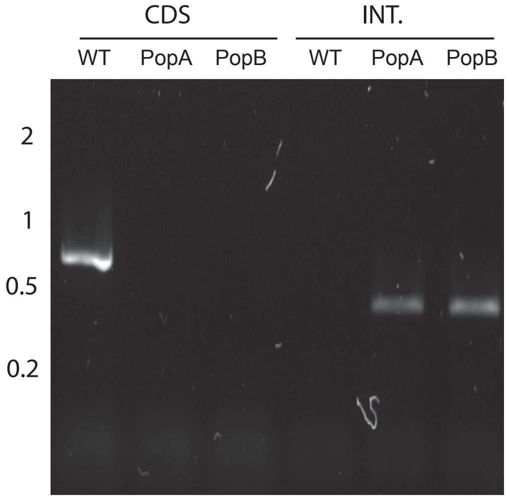

LmxM.19.0180

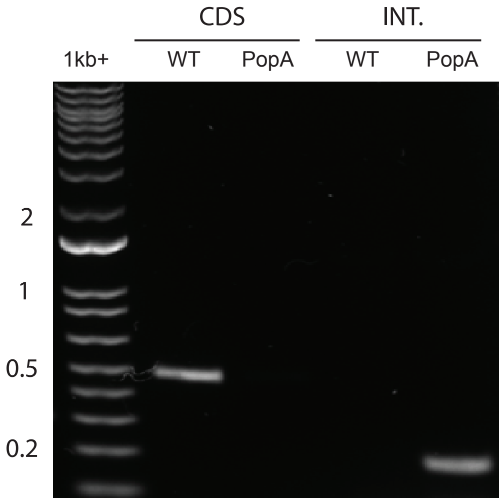

LmxM.27.0100

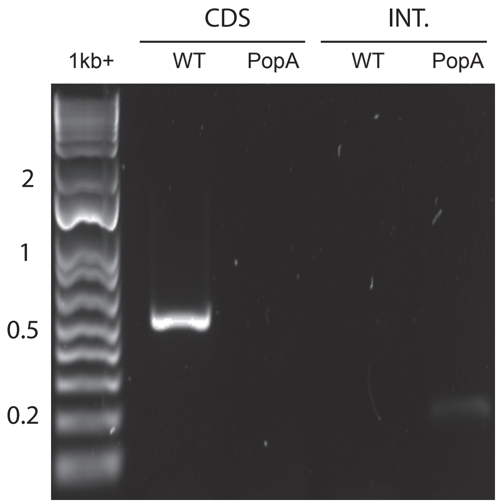

LmxM.34.5010

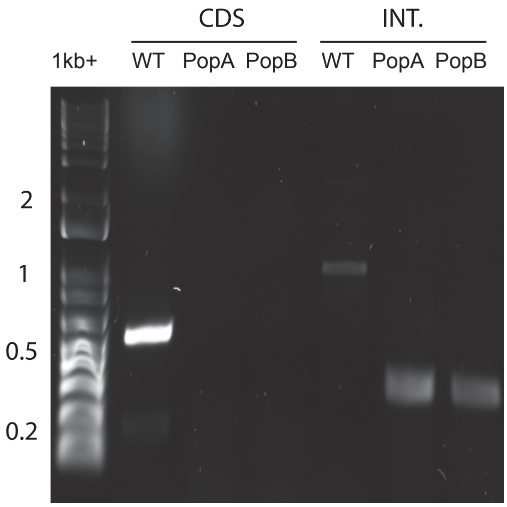

LmxM.26.0980

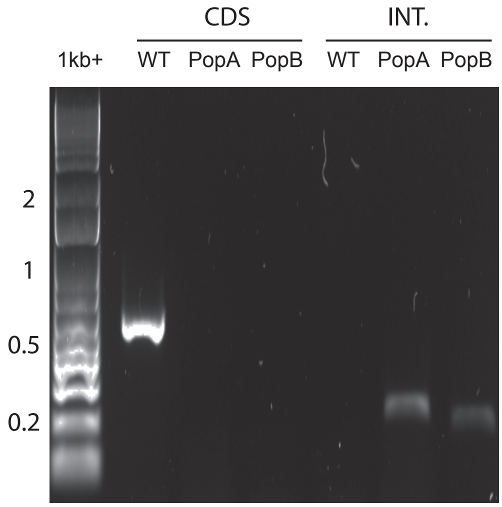

LmxM.36.0720

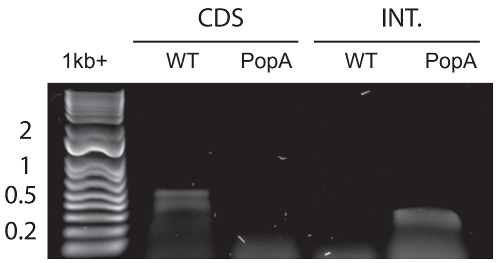

LmxM.17.0670

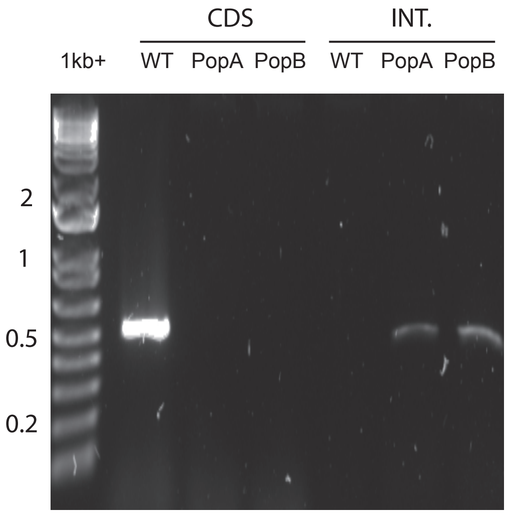

CMGC FAMILY

LmxM.01.0750

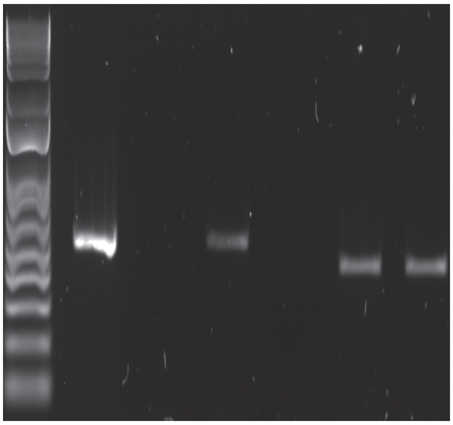

LmxM.12.0130

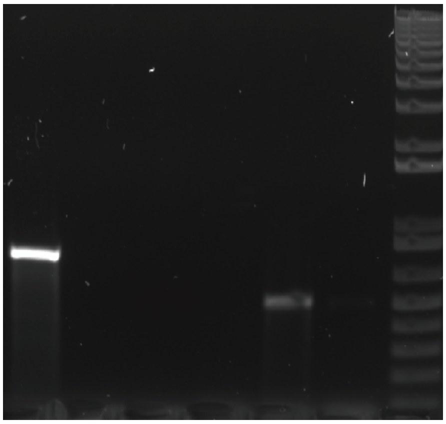

STE FAMILY

LmxM.08_29.2320

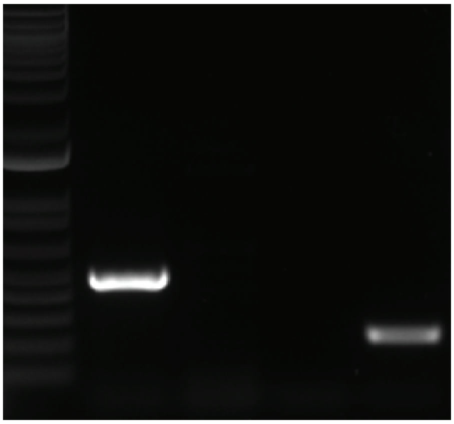

LmxM.08.1228

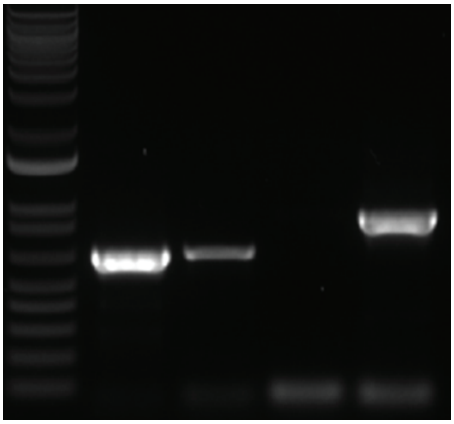

LmxM.15.1200

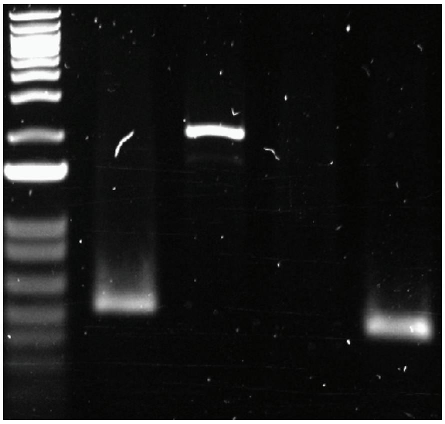

LmxM.17.0390

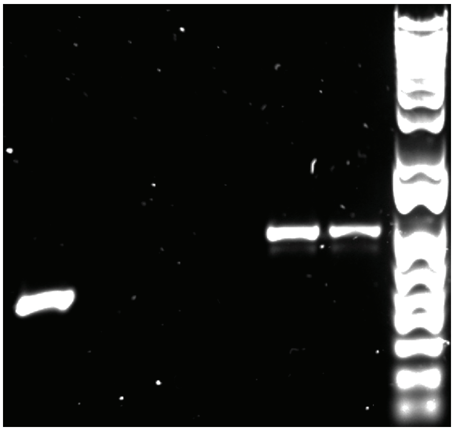

LmxM.20.0770

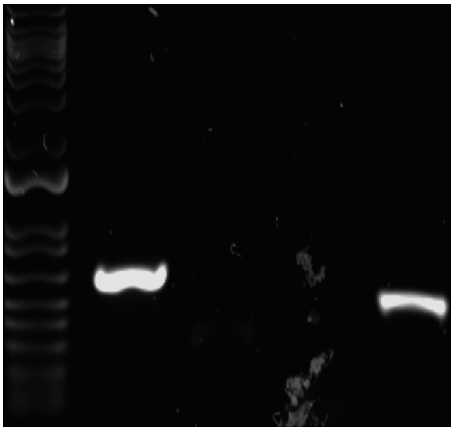

LmxM.21.0270

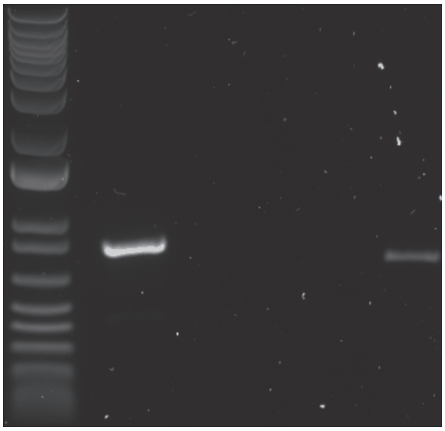

STE FAMILY

LmxM.25.1990

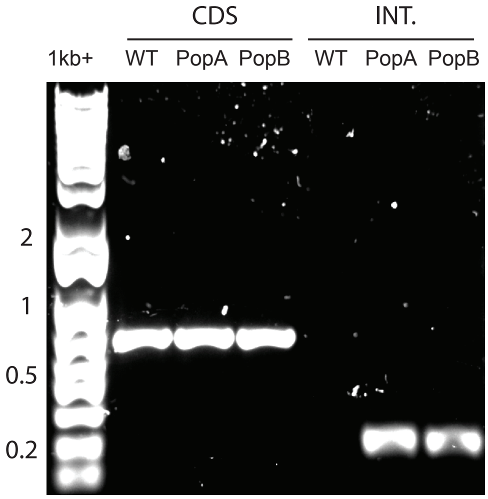

LmxM.31.1020

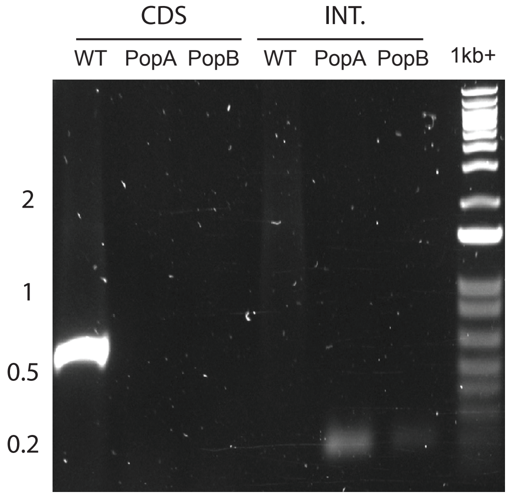

LmxM.04.0440

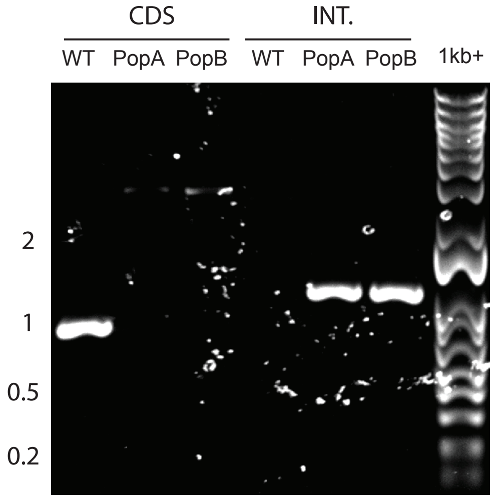

LmxM.05.0390

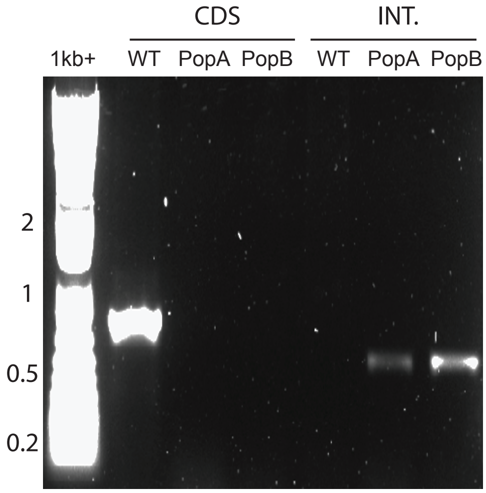

LmxM.06.0640

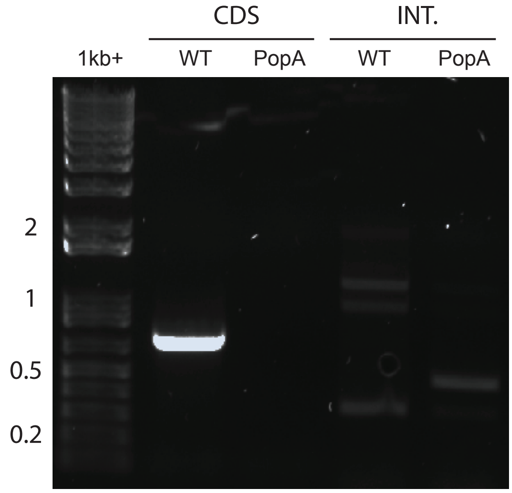

LmxM.07.0880

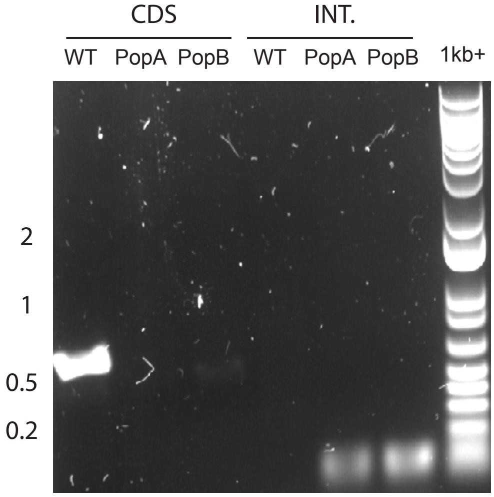

LmxM.19.0150

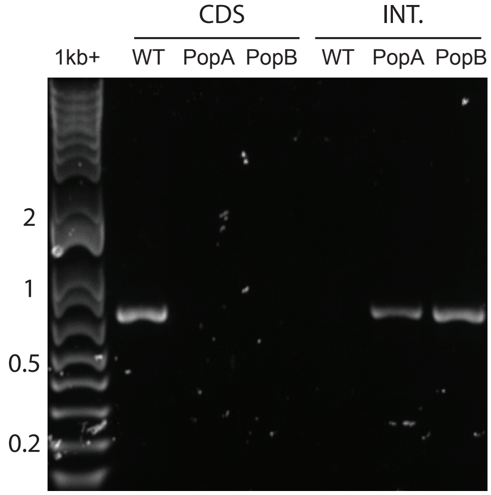

LmxM.21.0130

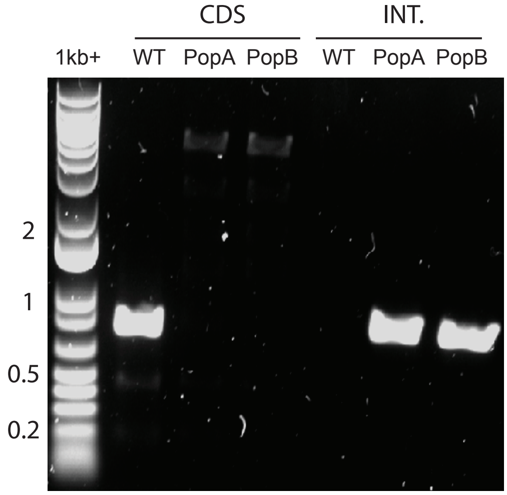

LmxM.24.1450

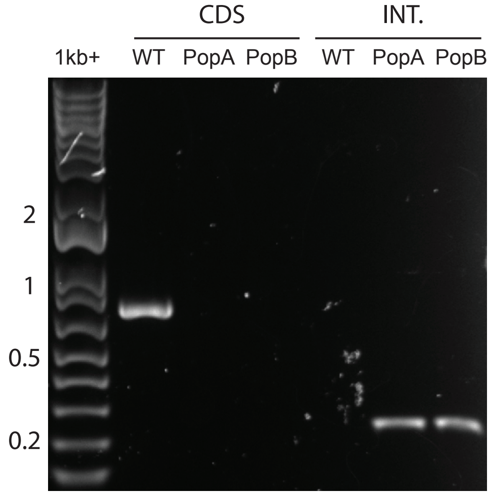

STE FAMILY

LmxM.26.1730

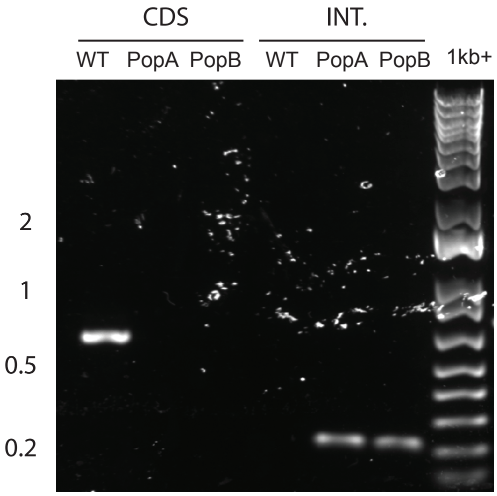

LmxM.29.0600

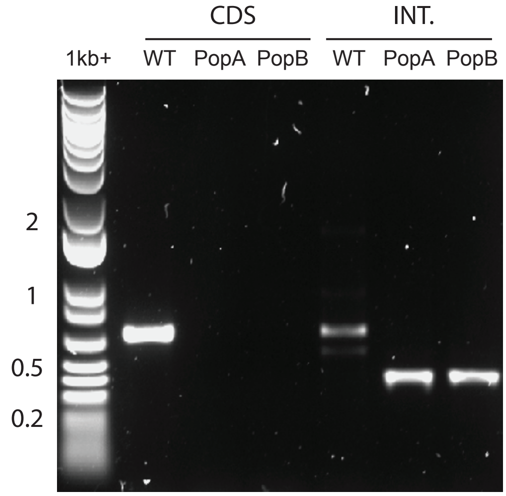

LmxM.29.3050

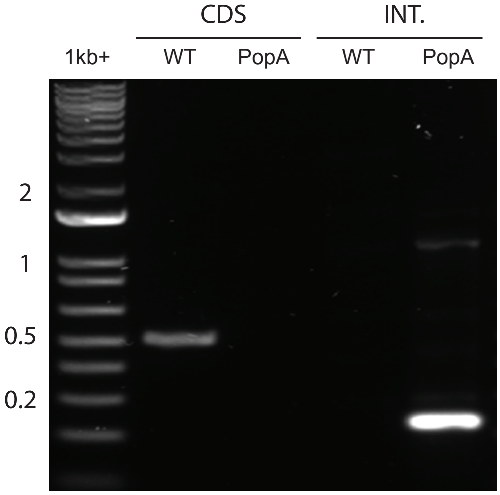

LmxM.31.0120

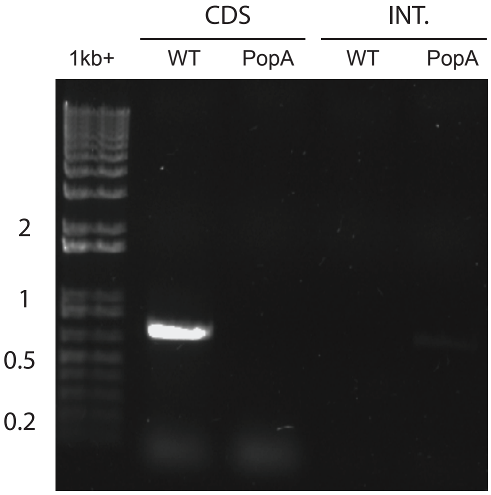

LmxM.31.0780

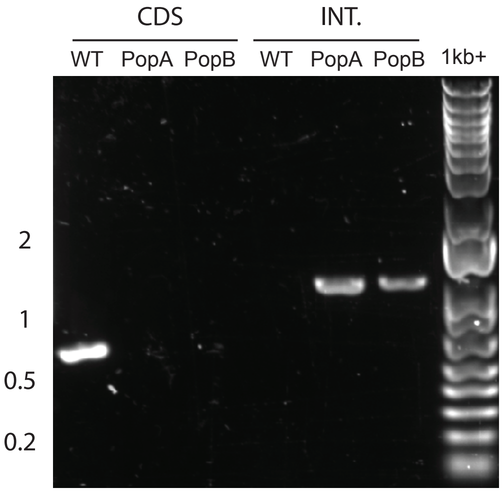

LmxM.31.0810

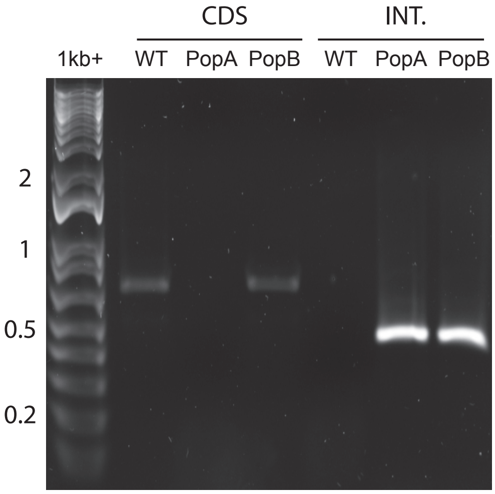

LmxM.32.1400

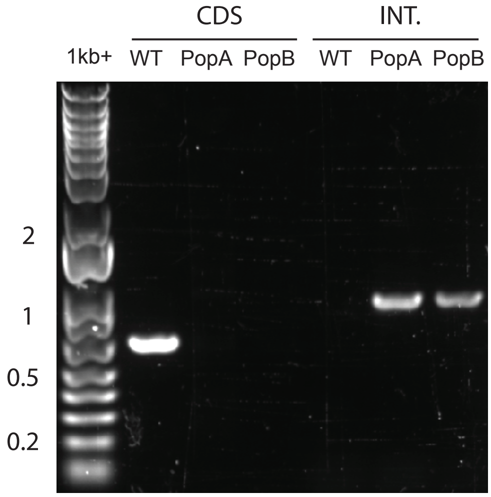

LmxM.32.2290

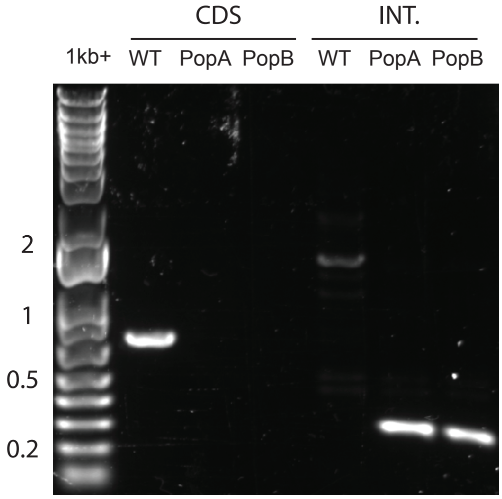

LmxM.36.0910

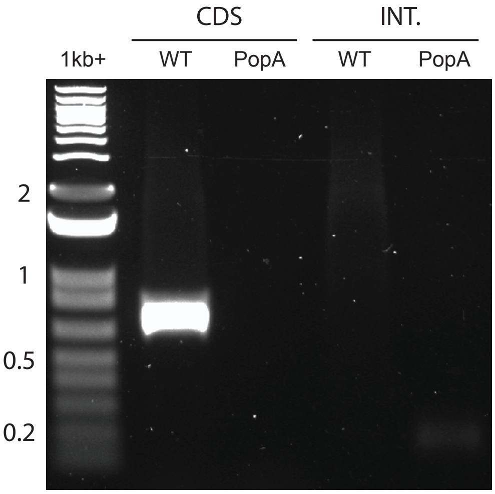

STE FAMILY

LmxM.16.0300

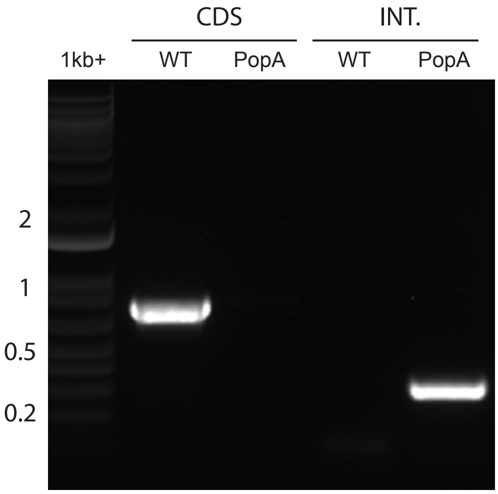

LmxM.07.0250

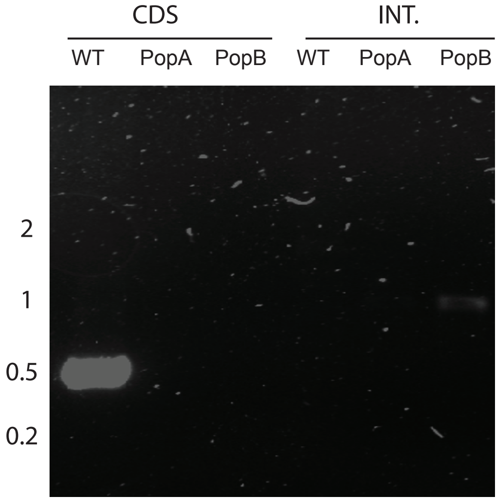

LmxM.19.1610

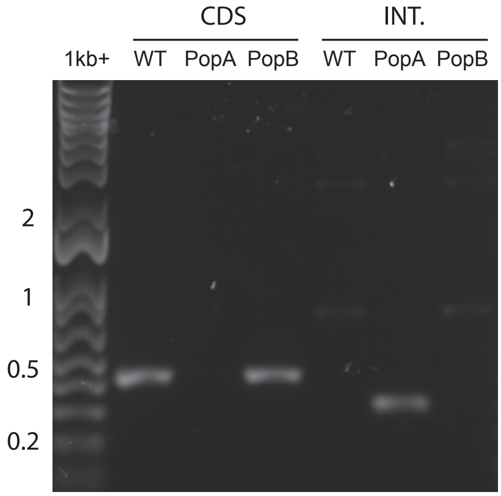

LmxM.33.2090

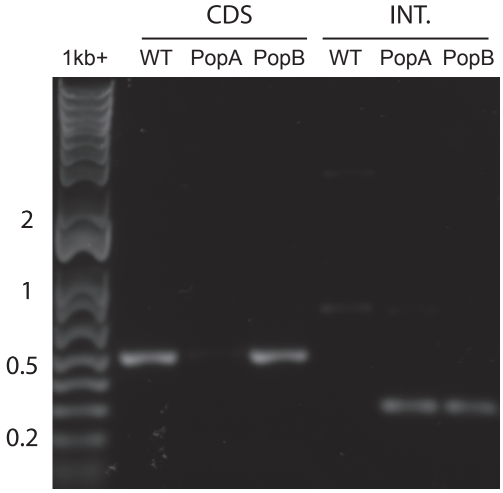

LmxM.34.4000

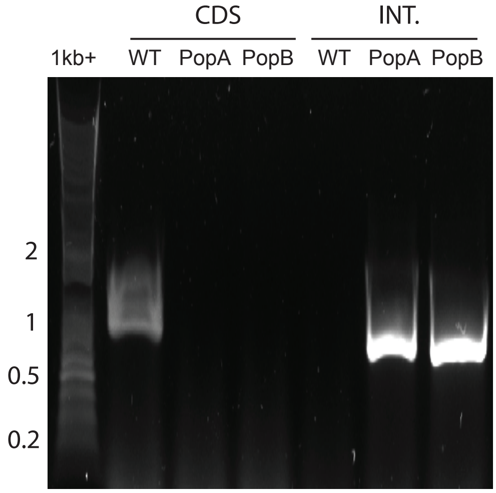

LmxM.36.3680

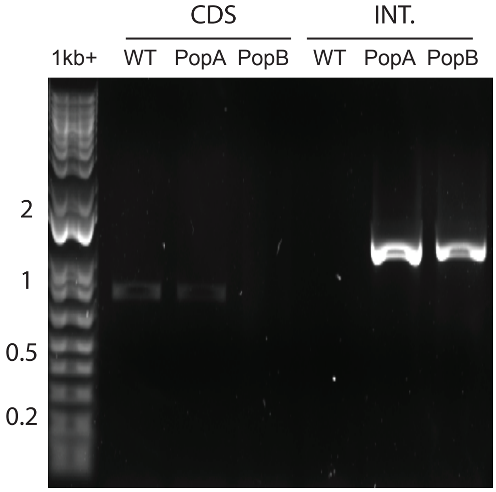

LmxM.30.1830

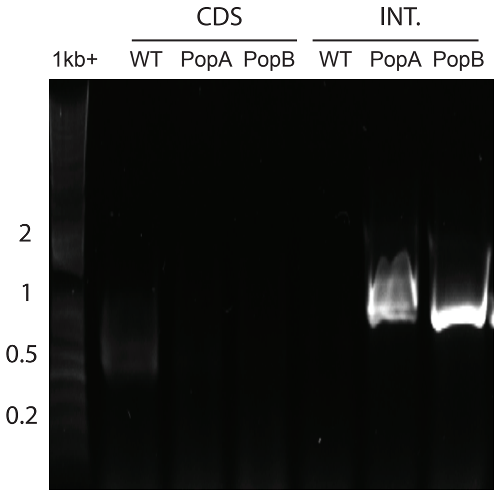

LmxM.30.1840

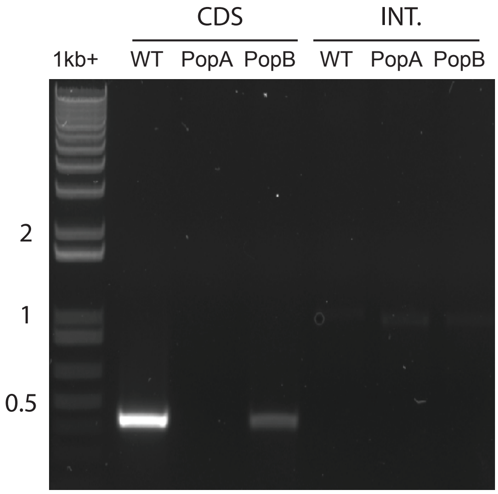

NEK FAMILY

LmxM.02.0290

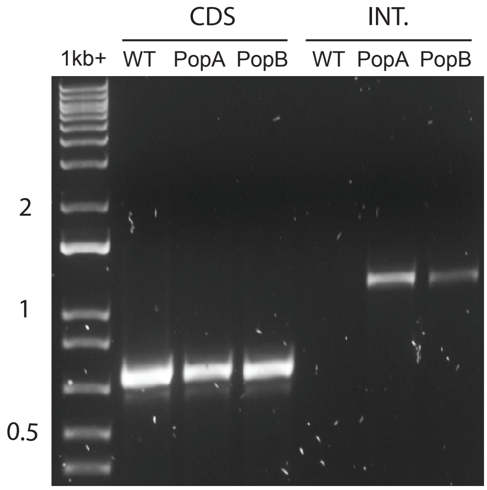

LmxM.07.0160

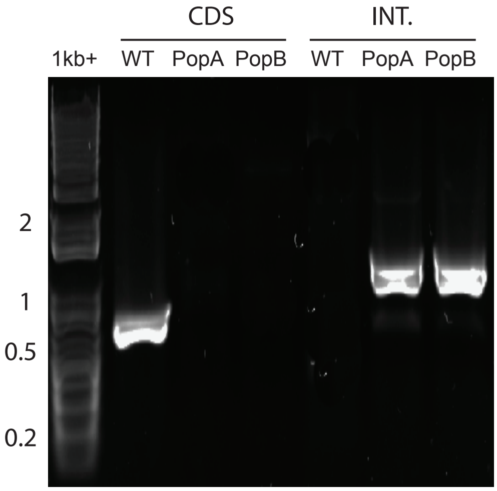

LmxM.07.0170

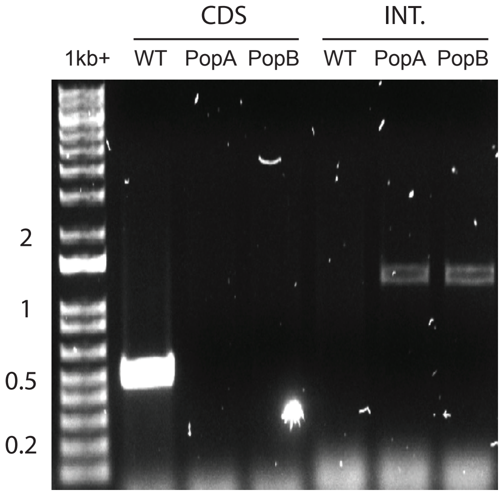

LmxM.08.0930

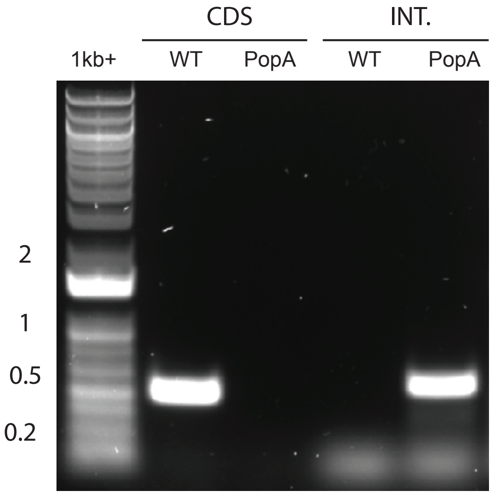

LmxM.14.1410

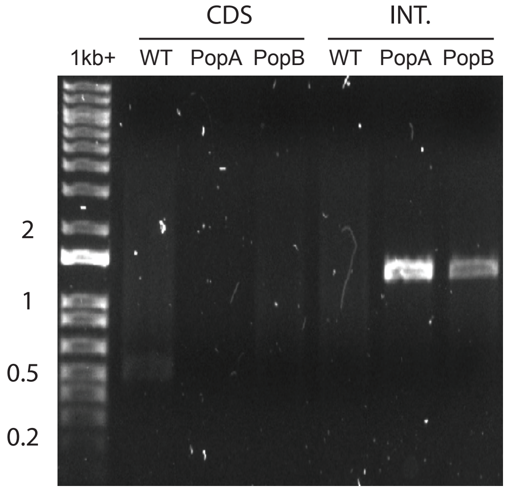

LmxM.21.0853

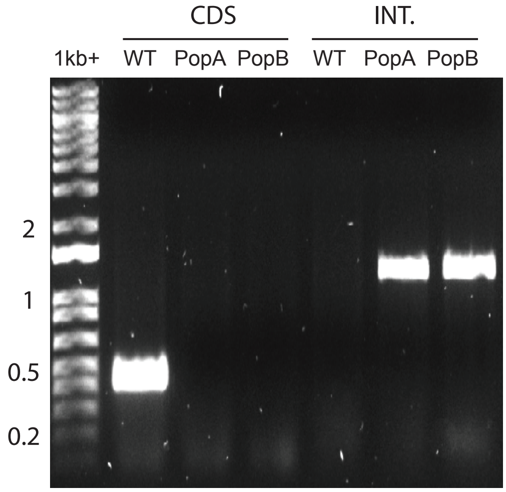

LmxM.21.1565

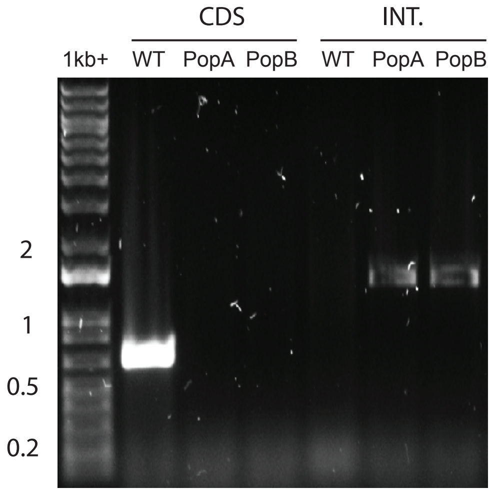

LmxM.22.0950

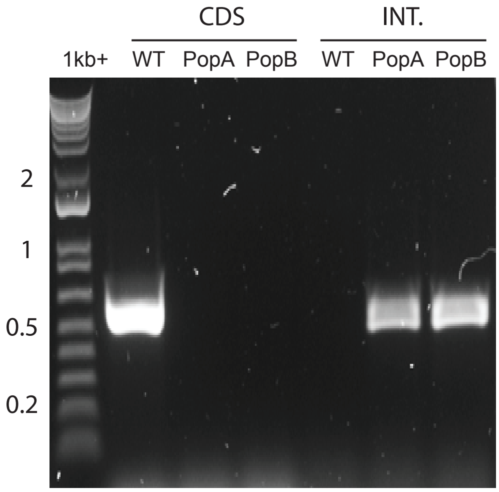

LmxM.26.2570

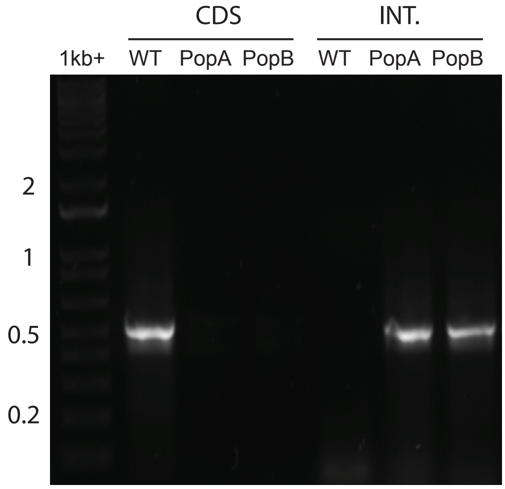

NEK FAMILY

LmxM.28.3000

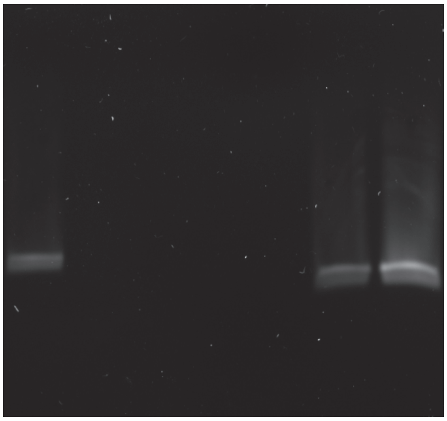

LmxM.08_29.2570

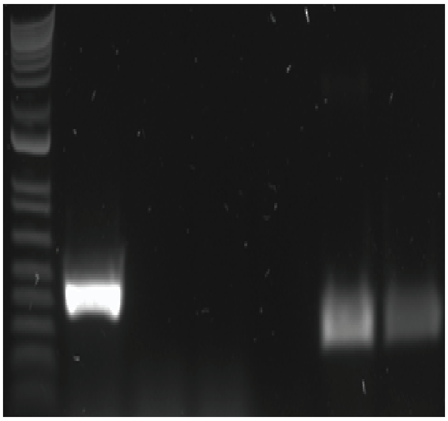

LmxM.08_29.2670

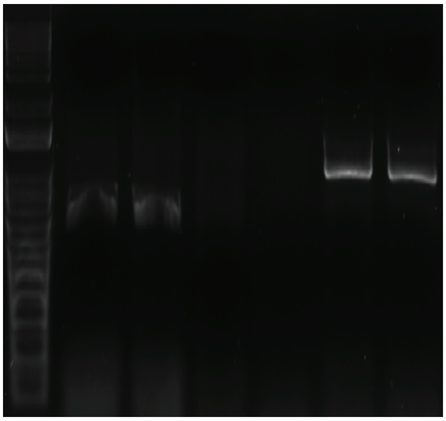

LmxM.29.2130

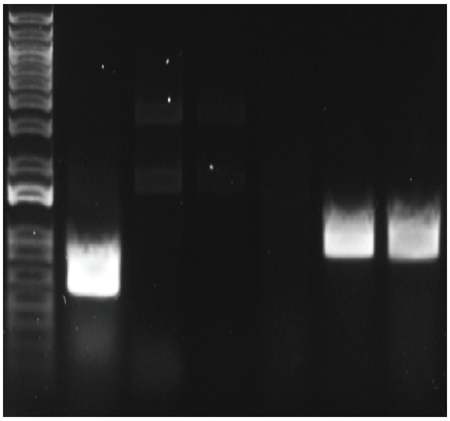

LmxM.30.2960

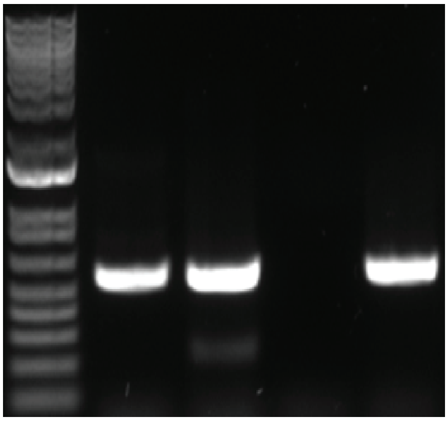

LmxM.30.3160

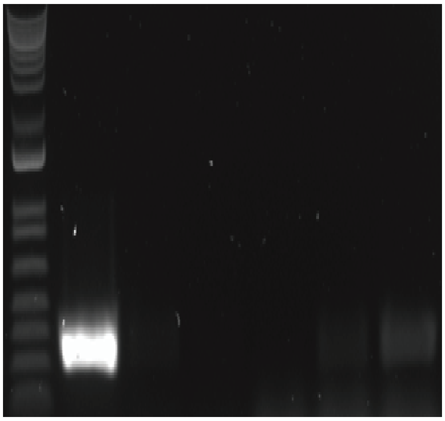

LmxM.31.0260

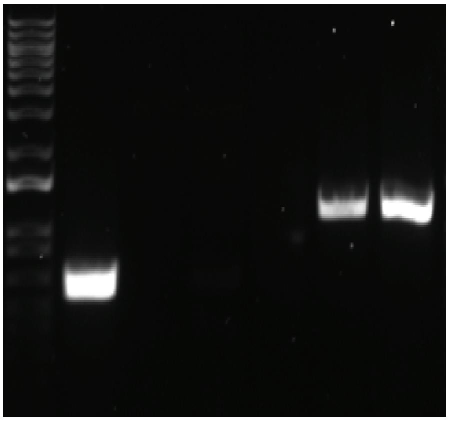

LmxM.31.1810

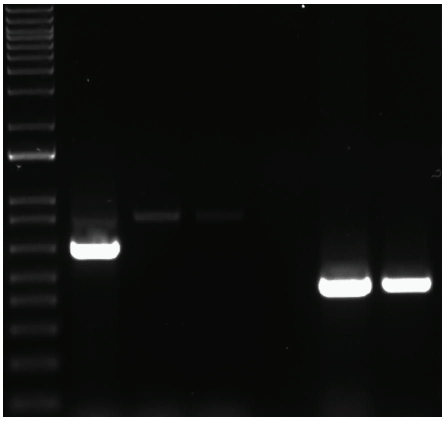

LmxM.32.1980

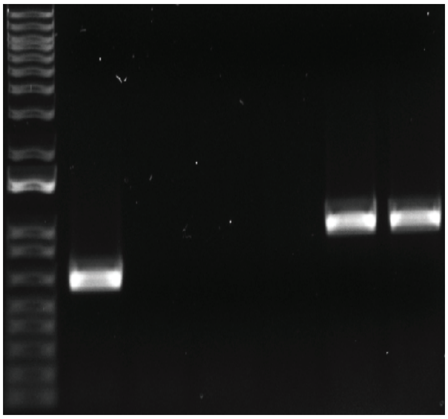

NEK FAMILY

LmxM.34.5190

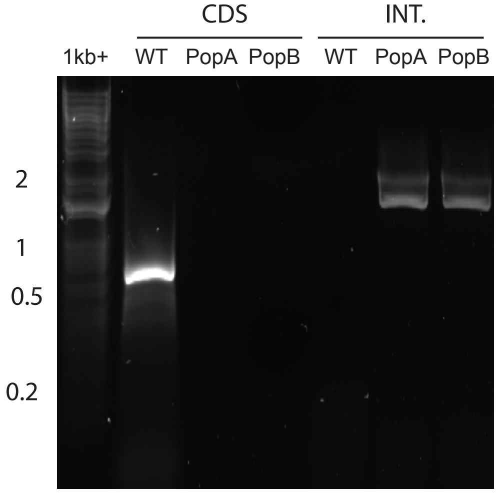

LmxM.36.1520

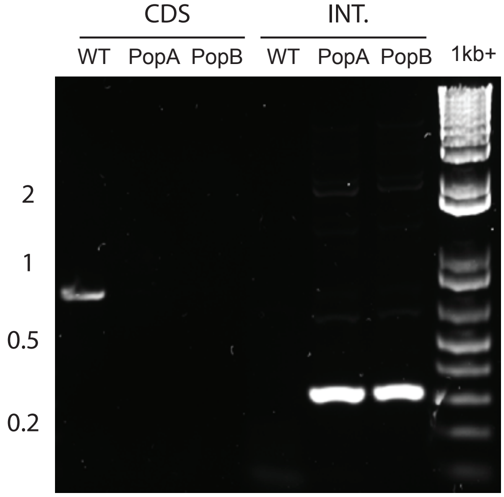

LmxM.36.1530

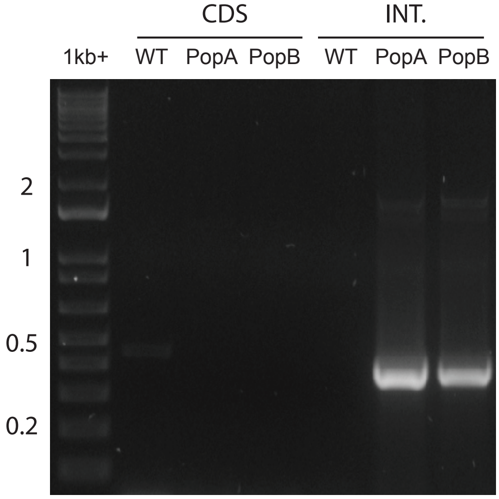

LmxM.36.2290

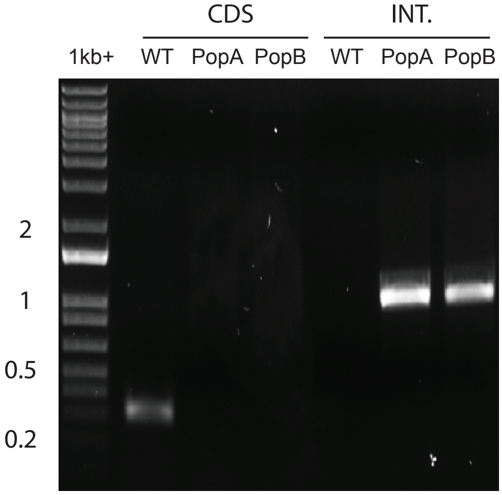

ORPHAN KINASES

LmxM.03.0350

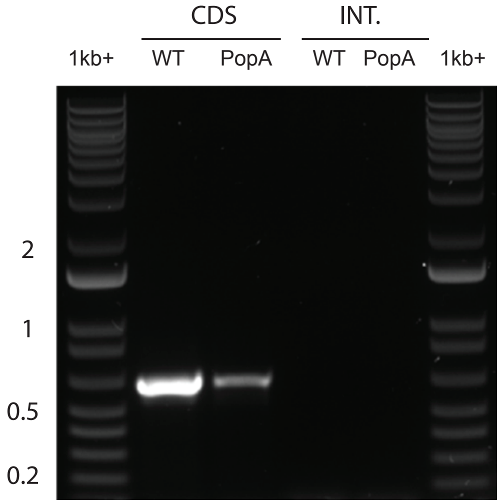

LmxM.08_29.2490

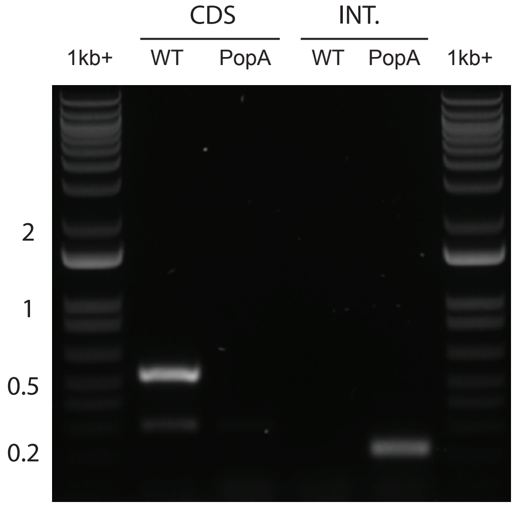

LmxM.26.2110

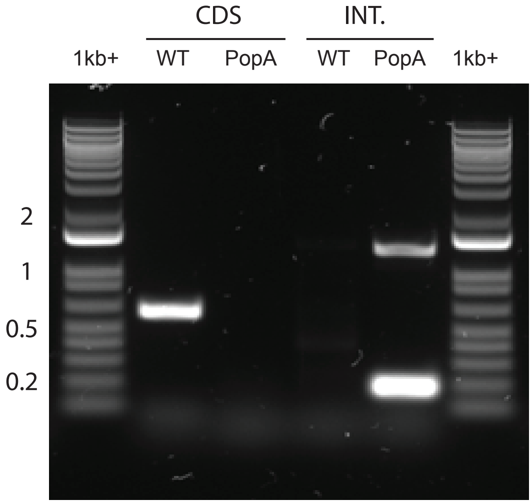

ORPHAN KINASES

LmxM.16.0870

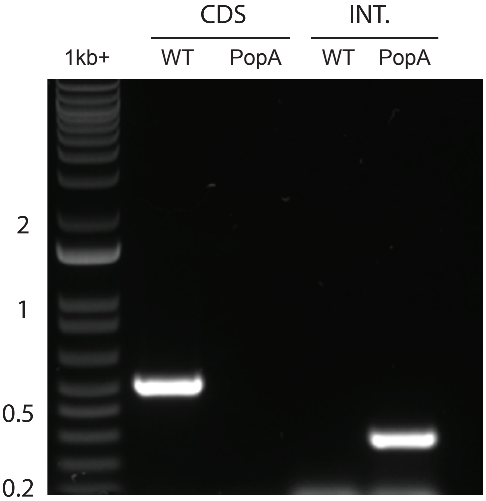

LmxM.11.0510

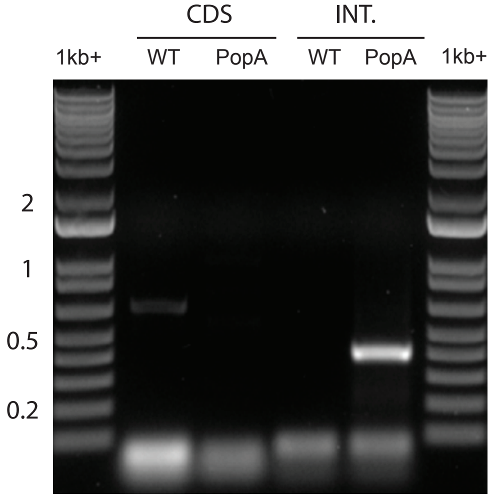

LmxM.25.1520

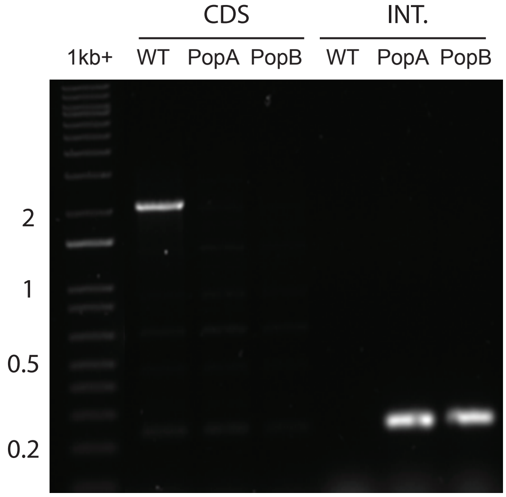

LmxM.02.0570

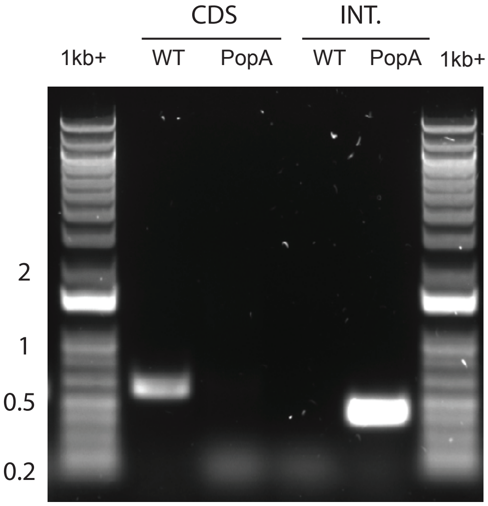

LmxM.10.0830

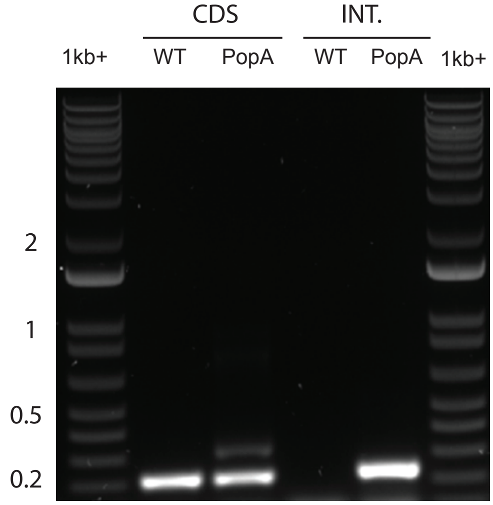

LmxM.34.4620

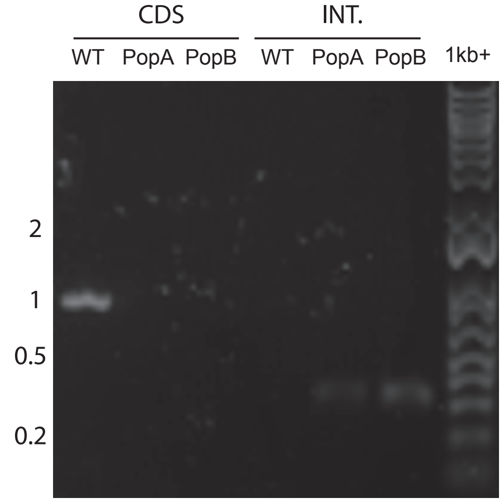

LmxM.08.0660

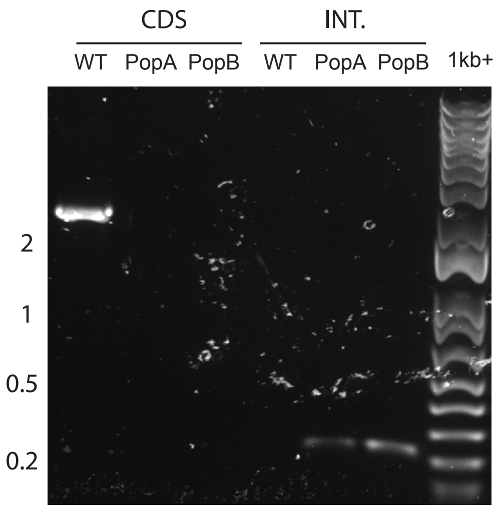

LmxM.26.2060

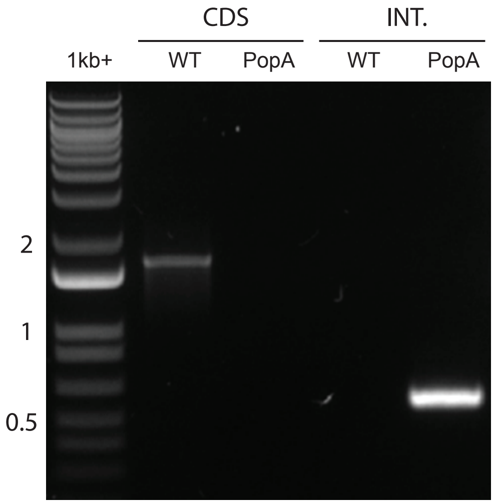

LmxM.28.1650

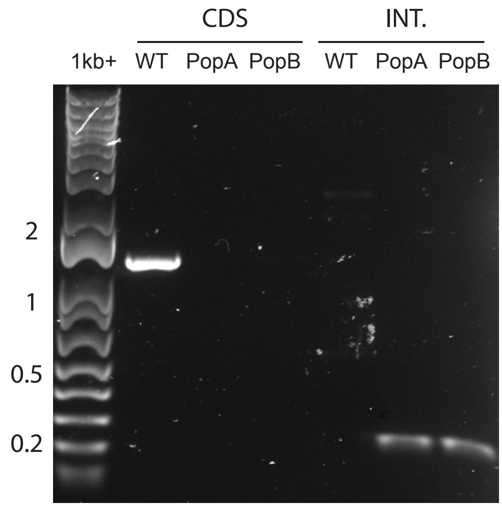

ORPHAN KINASES

LmxM.31.1290

CDS | INT.

1kb+ WT PopA PopB WT PopA PopB

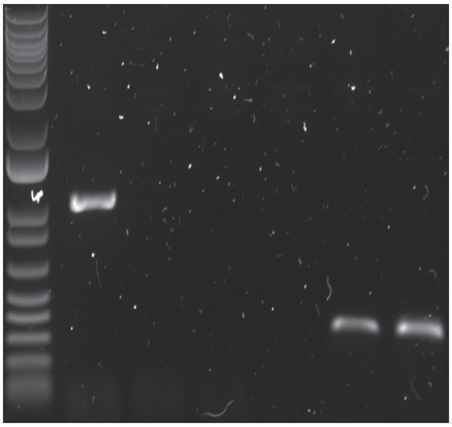

2
1
0.5
0.2

LmxM.33.2190

CDS | INT.

1kb+ WT PopA WT PopA

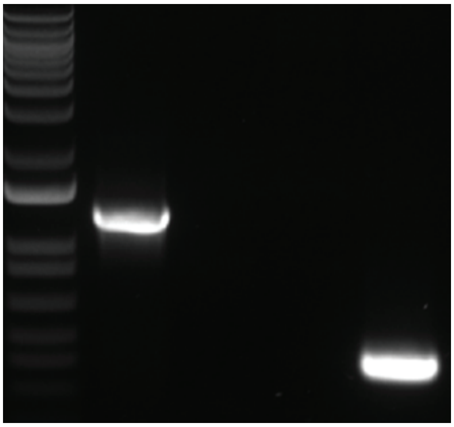

2
1
0.5

OTHER KINASES

LmxM.20.1330

CDS | INT.

1kb+ WT PopA WT PopA 1kb+

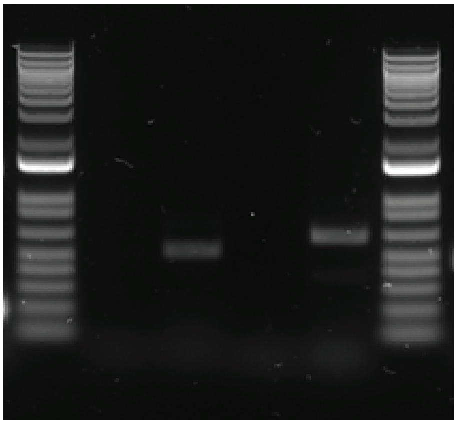

2
1
0.5
0.2

LmxM.20.1340

CDS | INT.

1kb+ WT PopA WT PopA

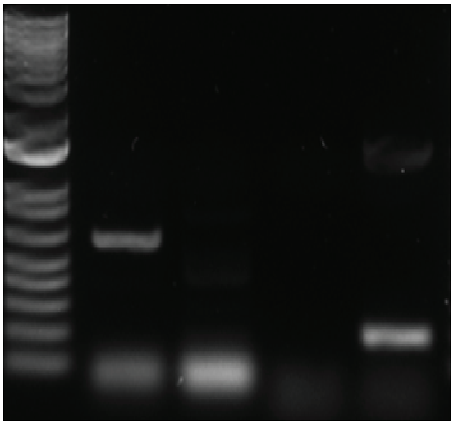

2
1
0.5
0.2

LmxM.08_29.0370

CDS | INT.

1kb+ WT PopA WT PopA

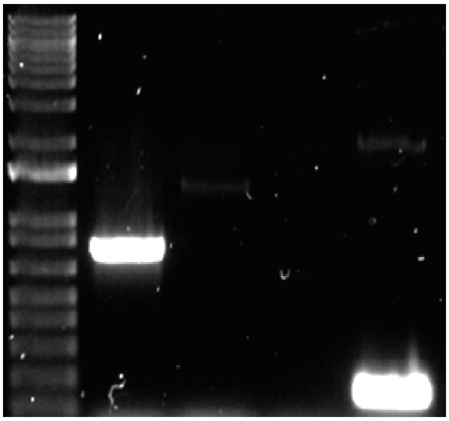

2
1
0.5
0.2

LmxM.34.2870

CDS | INT.

1kb+ WT PopA WT PopA

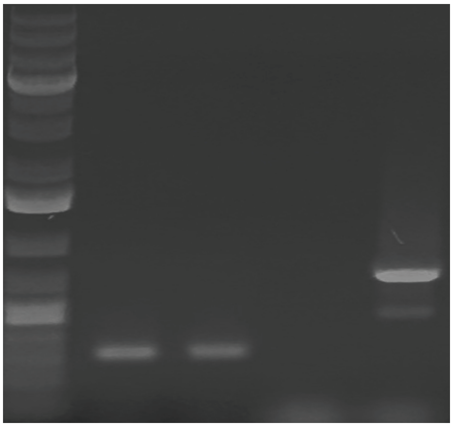

2
1
0.5
0.2

LmxM.26.2440

CDS | INT.

1kb+ WT PopA PopB WT PopA PopB

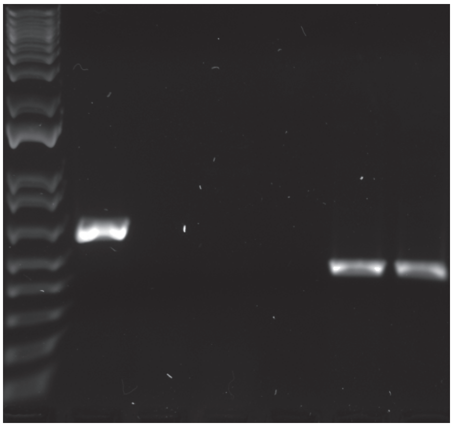

2
1
0.5
0.2

LmxM.28.0520

CDS | INT.

1kb+ WT PopA WT PopA

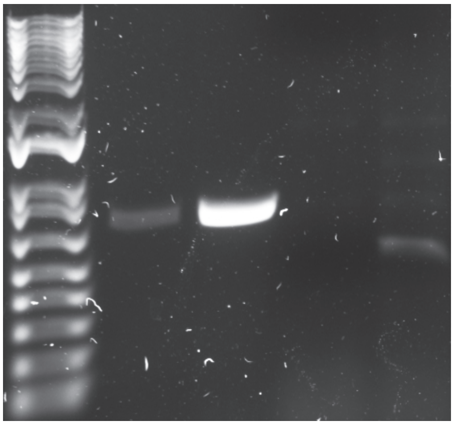

2
1
0.5
0.2

OTHER KINASES

LmxM.08_29.1330

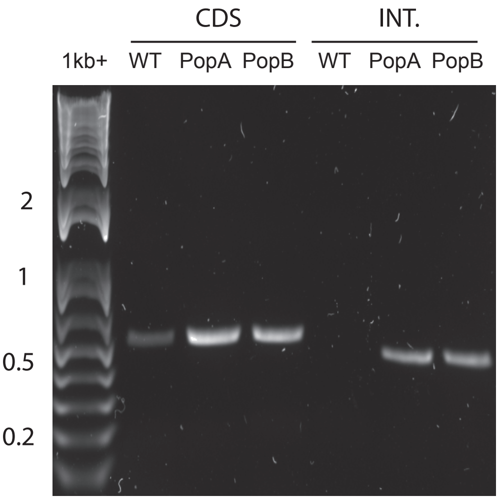

LmxM.19.0590

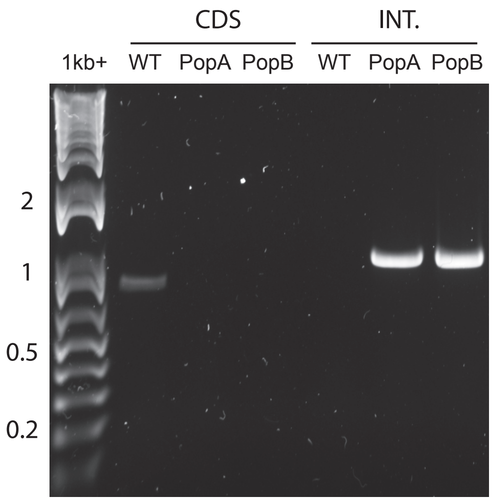

LmxM.11.0250

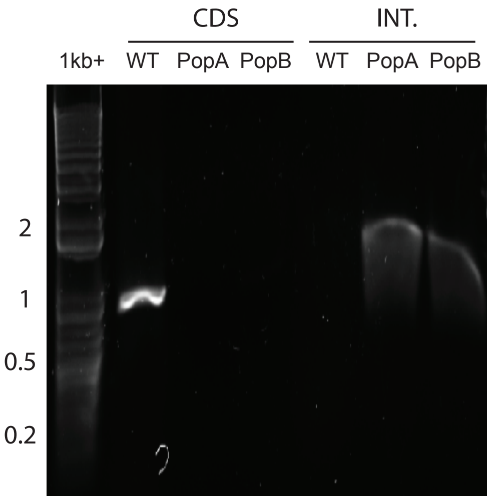

LmxM.24.1730

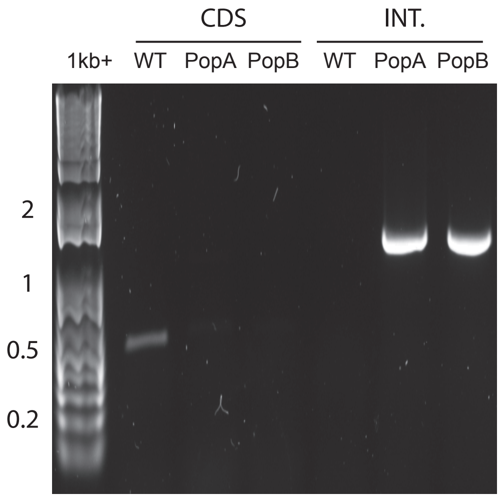

LmxM.34.2320

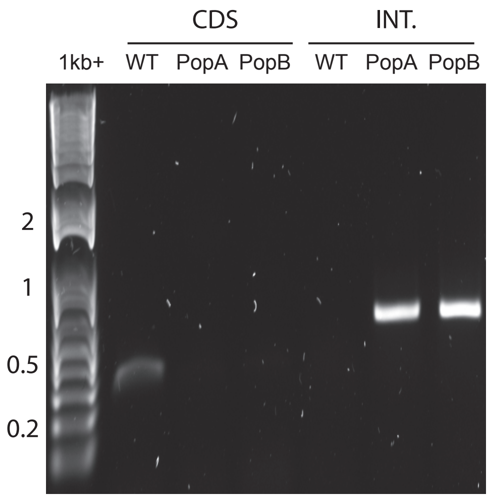

LmxM.34.1730

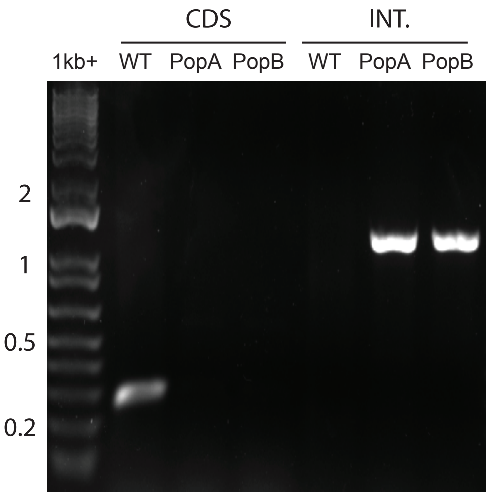

LmxM.02.0360

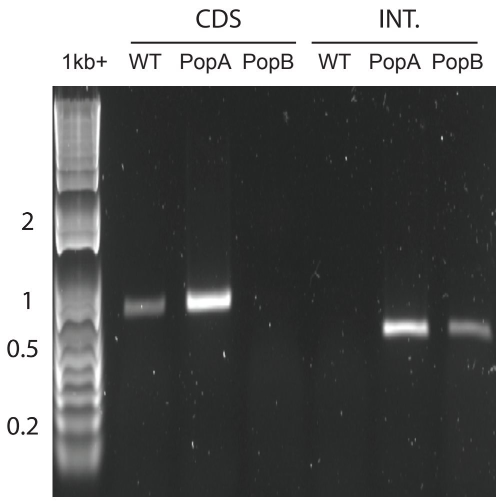

LmxM.15.0770

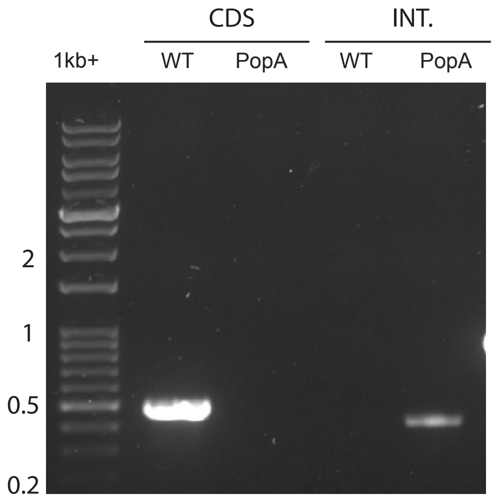

LmxM.33.0030

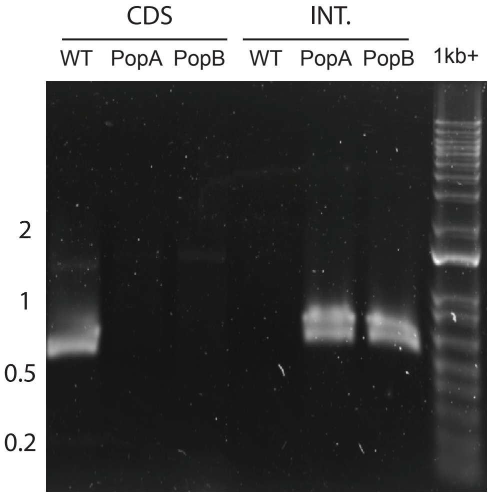

OTHER KINASES

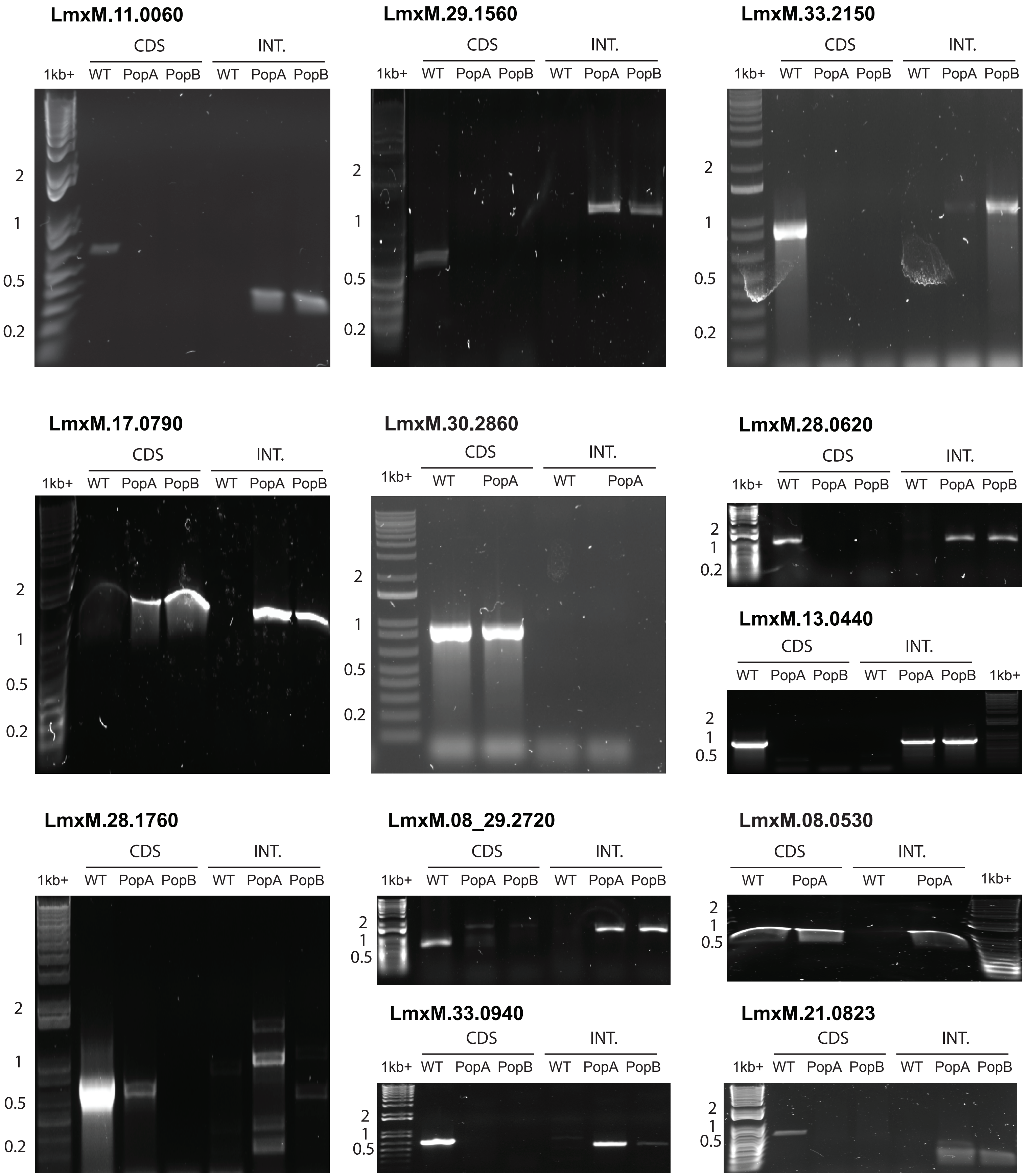

OTHER KINASES

LmxM.36.2630

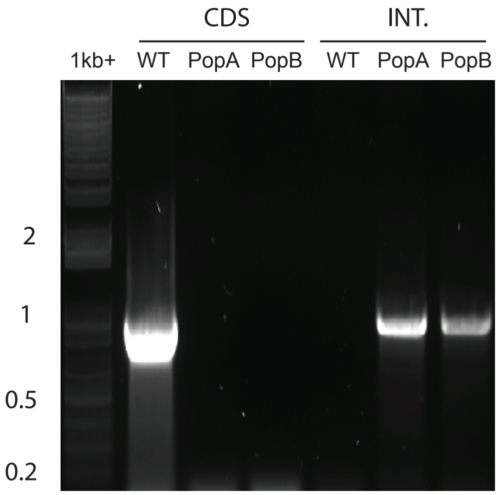

LmxM.22.1150

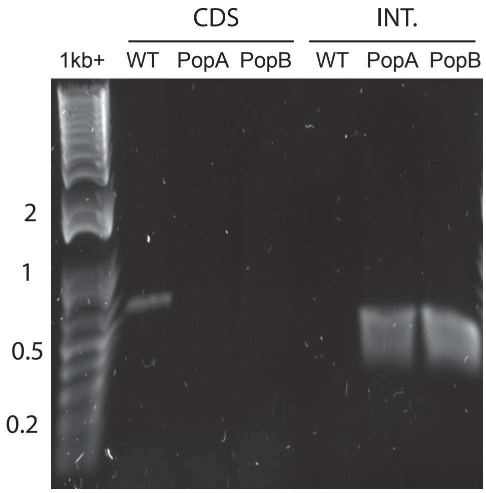

PIKK (aPK)

LmxM.08_29.1450

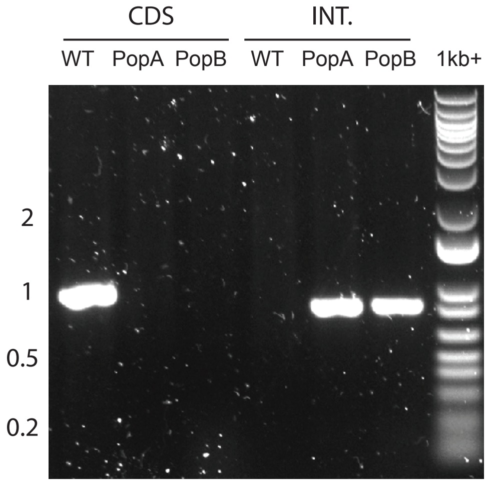

LmxM.24.2010

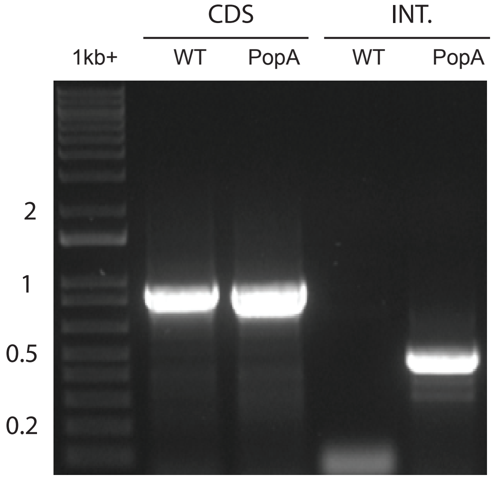

LmxM.33.3590

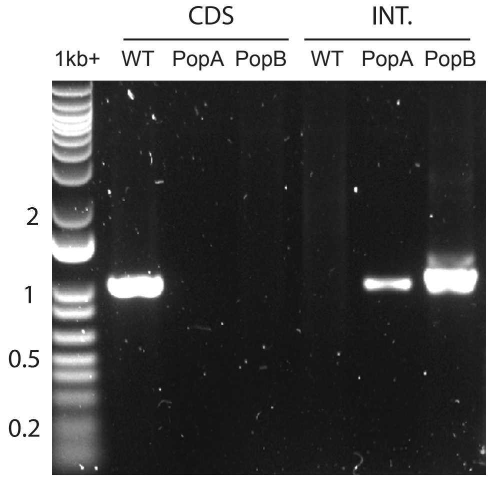

LmxM.27.0890

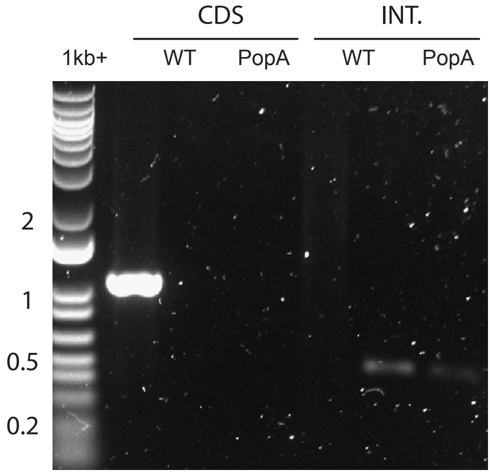

LmxM.33.3090

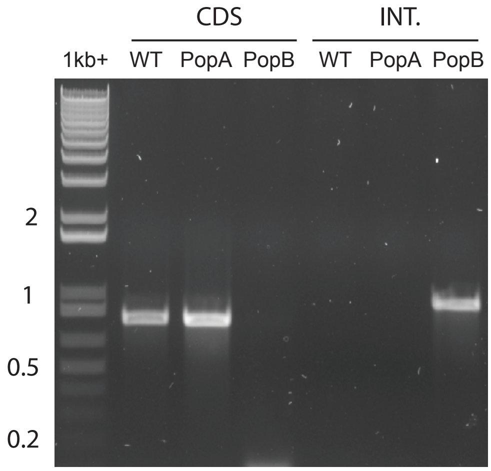

LmxM.34.0560

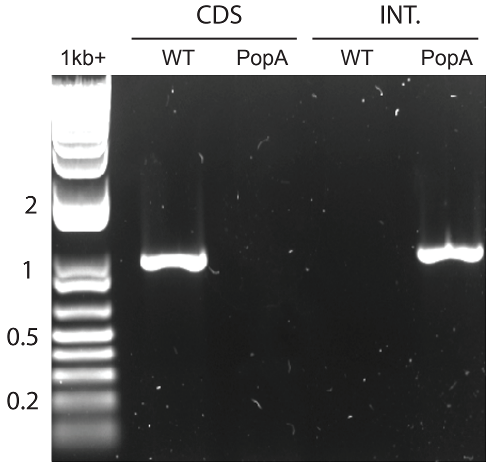

OTHER KINASES

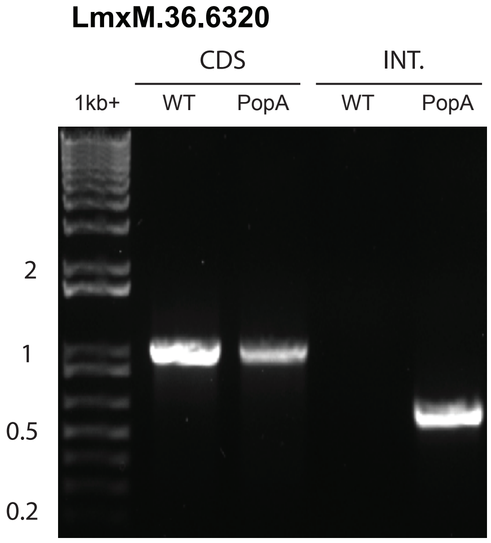

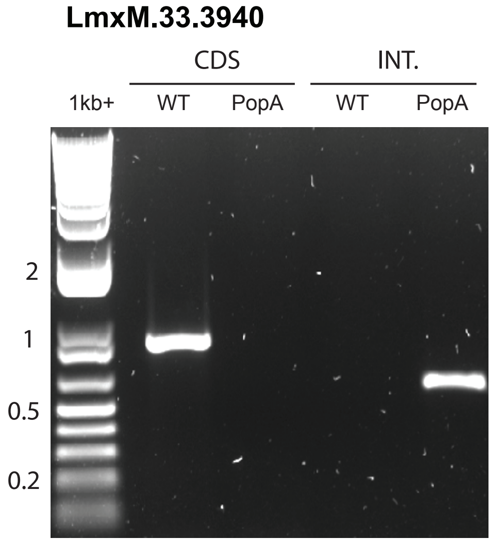

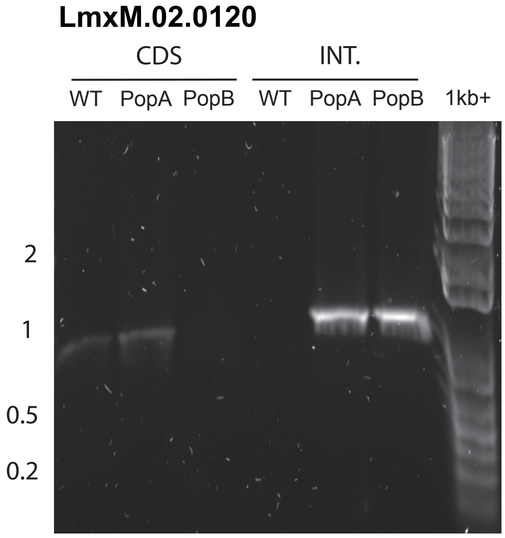

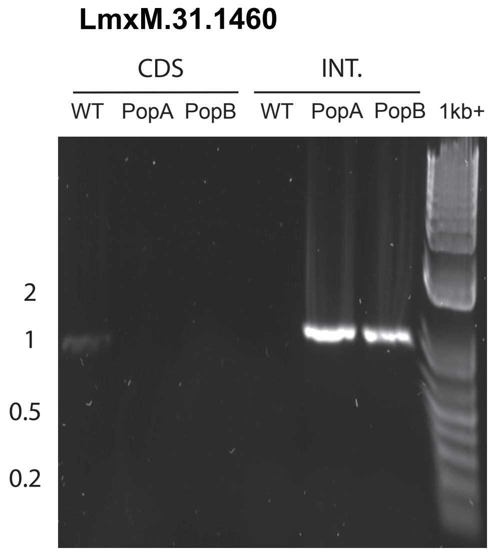

Supplement: Supplementary file 5 — Supplementary Data 2 [file 41467_2021_21360_MOESM5_ESM.pdf]
