# Supplementary material for: Systematic functional analysis of Leishmania protein kinases identifies regulators of differentiation or survival
Source: Nat Commun. 2021 Feb 23;12:1244. doi: 10.1038/s41467-021-21360-8 (PMC7902614; doi:10.1038/s41467-021-21360-8)

Supplementary Data 5. **Localisation of mNeonGreen tagged protein kinases**.

AGC FAMILY

LmxM.15.1550 (RAC1 – beta Serine/threonine kinase)

N-terminus

Primary localisation: mitochondrion

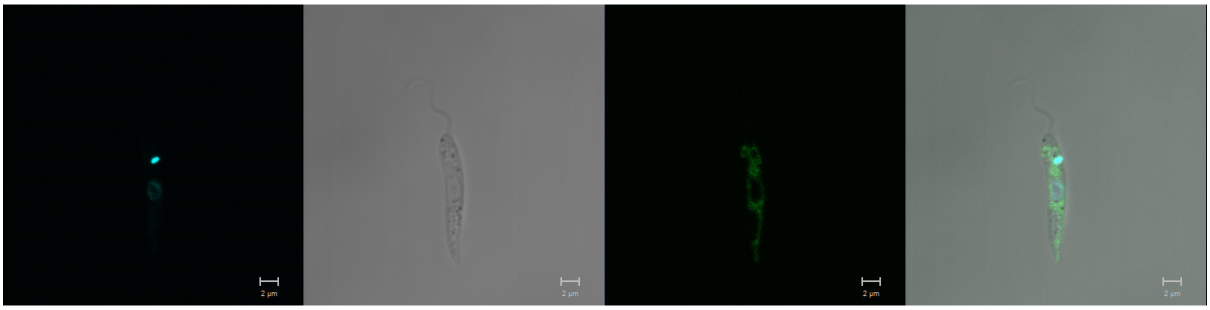

C-terminus

Primary localisation: mitochondrion

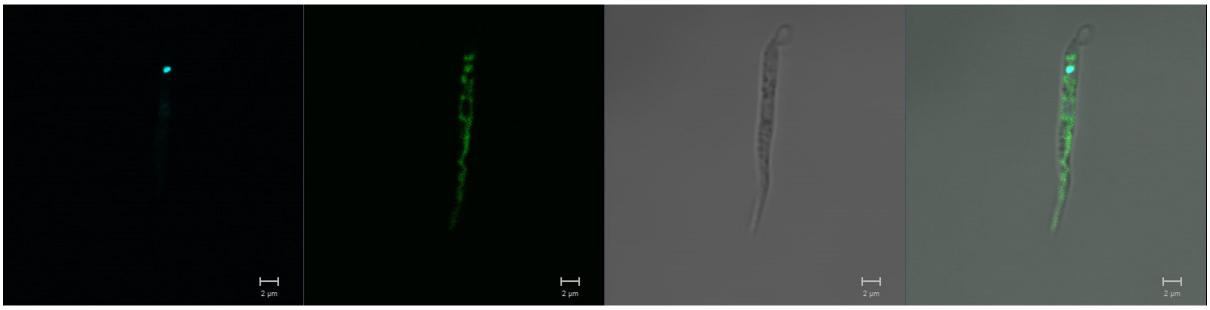

LmxM.25.2340 (AEK1, AGC essential kinase 1)

N-terminus

Primary localisation: cytoplasm

Secondary localisation: flagellum, basal body

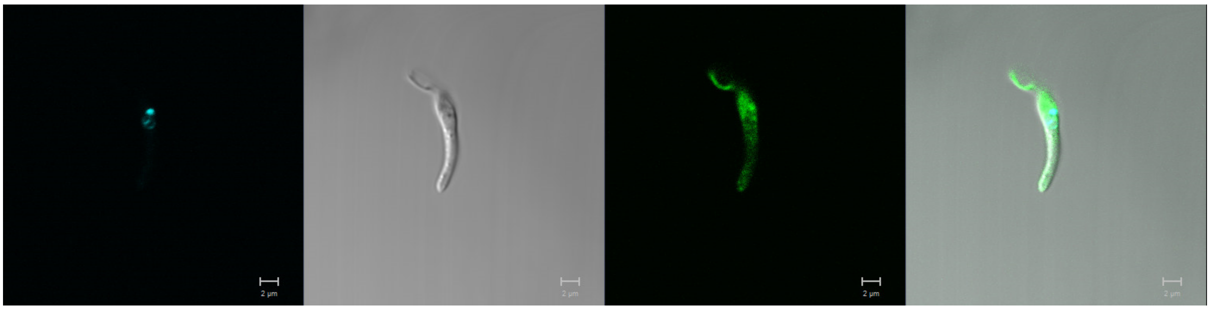

LmxM.28.1670 (ZFK, differentiation inhibitory kinase)
N-terminus

Primary localisation: lysosome

Secondary localisation: cytoplasm

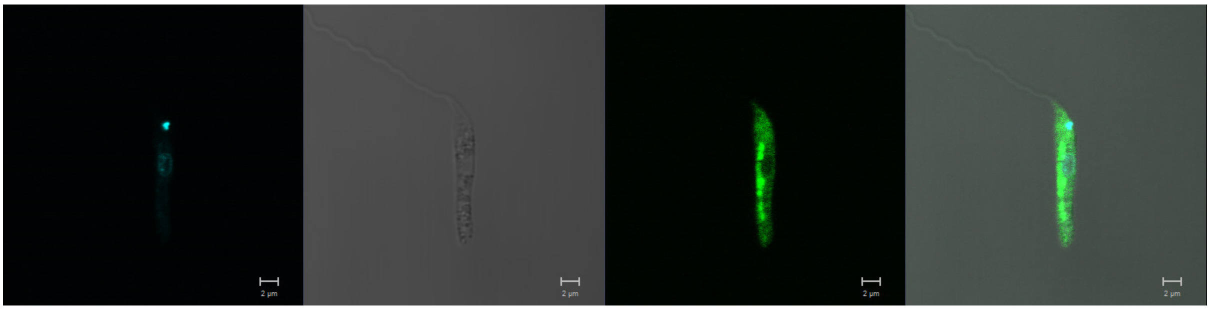

C-terminus

Primary localisation: endomembrane

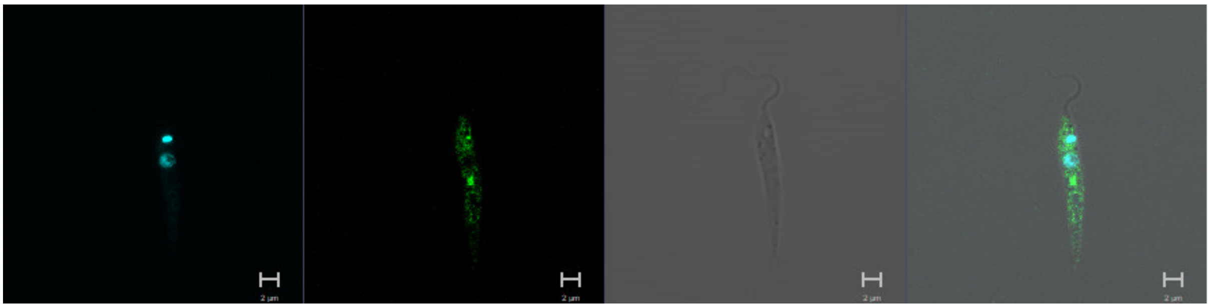

LmxM.29.0800 (RAC2 serine-threonine kinase)
N-terminus

Primary localisation: cytoplasm

Secondary localisation: flagellum, kinetoplast

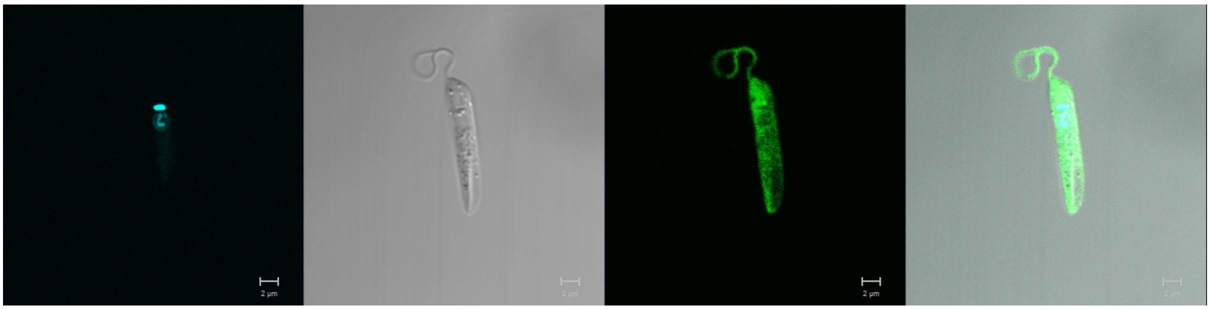

LmxM.29.1000
N-terminus

Primary localisation: cytoplasm

Secondary localisation: flagellum, nucleus, kinetoplast

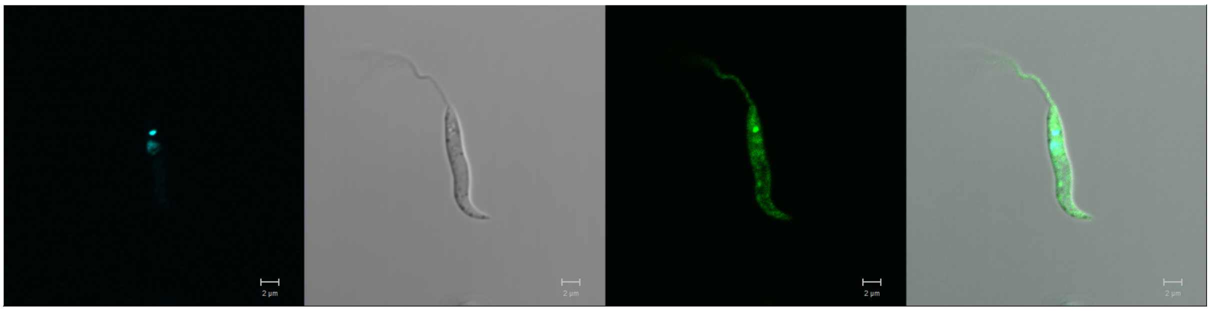

LmxM.30.1530
N-terminus

Primary localisation: cytoplasm

Secondary localisation: nucleus

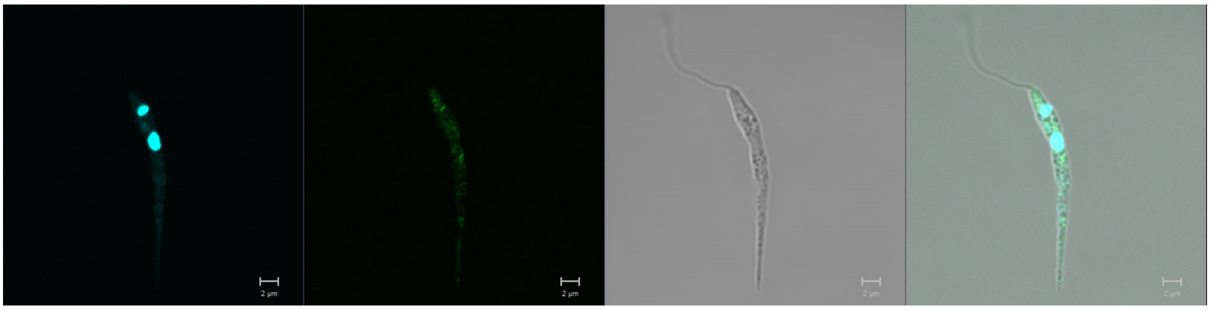

LmxM.06.1180
N-terminus

Primary localisation: cytoplasm

Secondary localisation: flagellum, nucleus

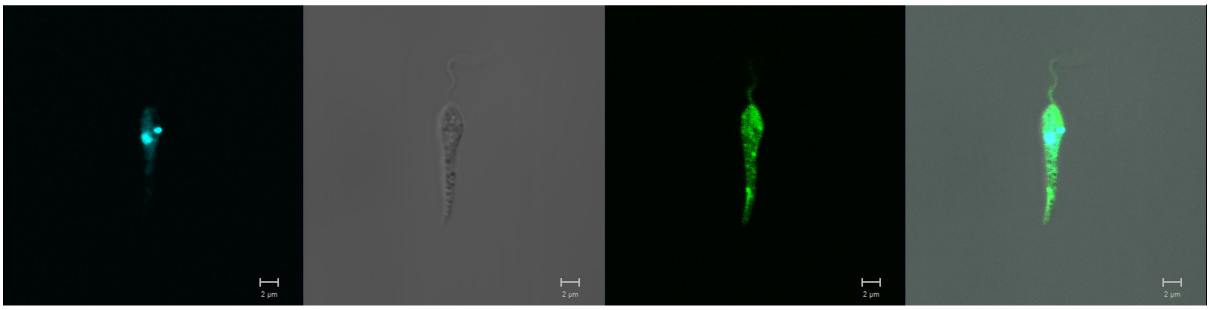

LmxM.18.1080 (PKAC1, protein kinase A catalytic subunit 1)
N-terminus

Primary localisation: pellicular membrane

Secondary localisation: cytoplasm, flagellum, nucleus

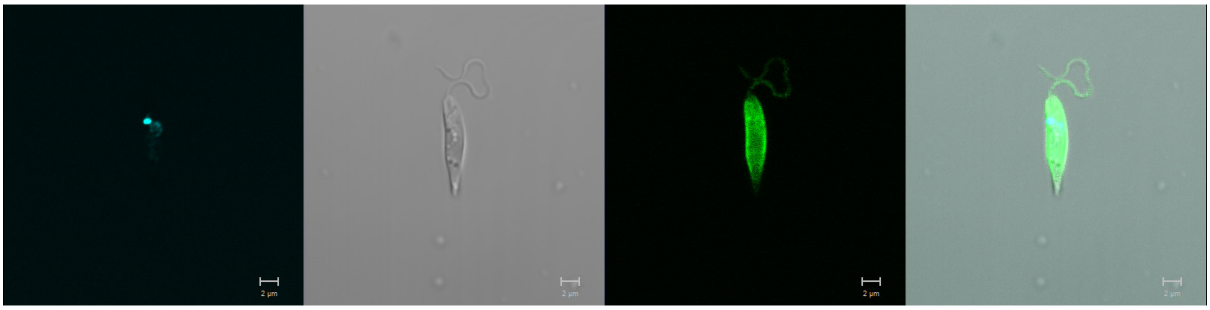

LmxM.34.3960 (PKAC2a, protein kinase A catalytic subunit isoform 2)
N-terminus

Primary localisation: pellicular membrane

Secondary localisation: kinetoplast, cytoplasm, flagellum, nucleus

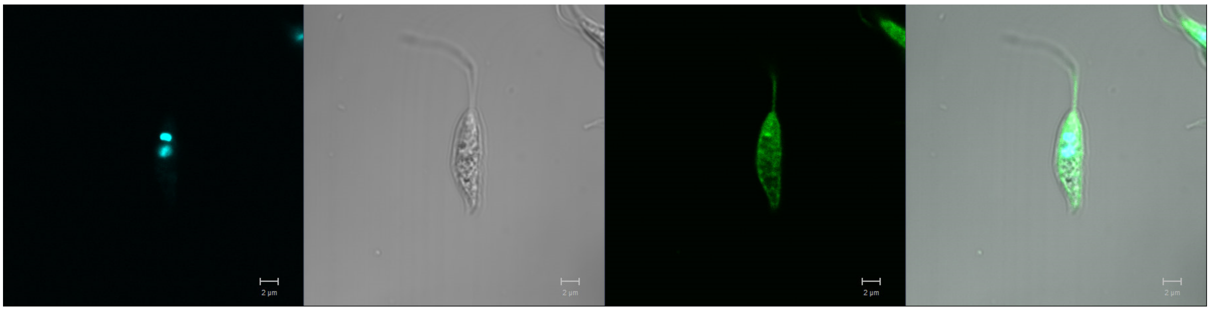

LmxM.34.4010 (PKAC1a, protein kinase A catalytic subunit isoform 1)
N-terminus

Primary localisation: nucleus

Secondary localisation: cytoplasm (only during division)

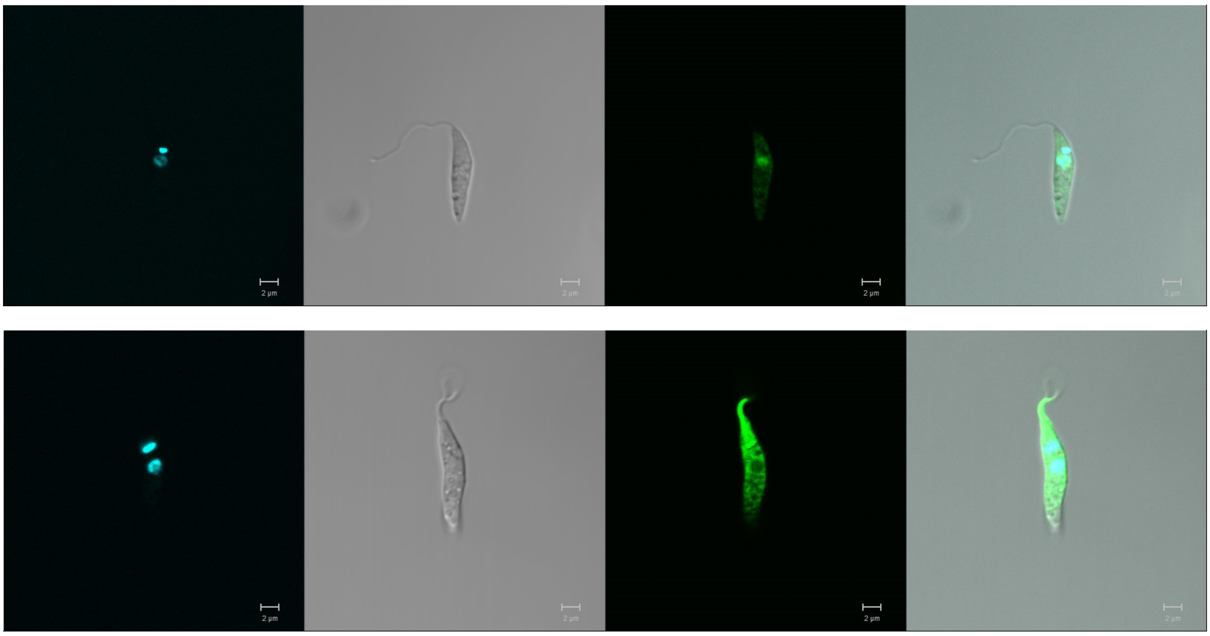

LmxM.03.0780
N-terminus

Primary localisation: cytoplasm

Secondary localisation: flagellum

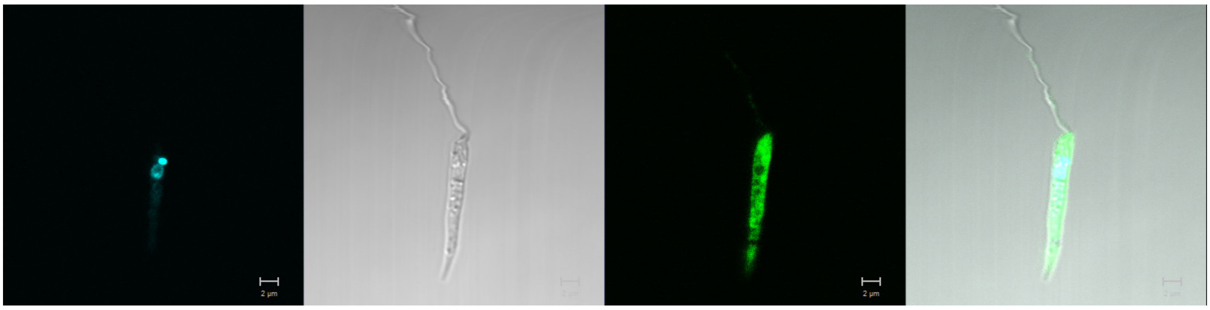

<u>CAMK FAMILY</u>

LmxM.27.2460
N-terminus

Primary localisation: basal body

Secondary localisation: cytoplasm, flagellum, nucleus

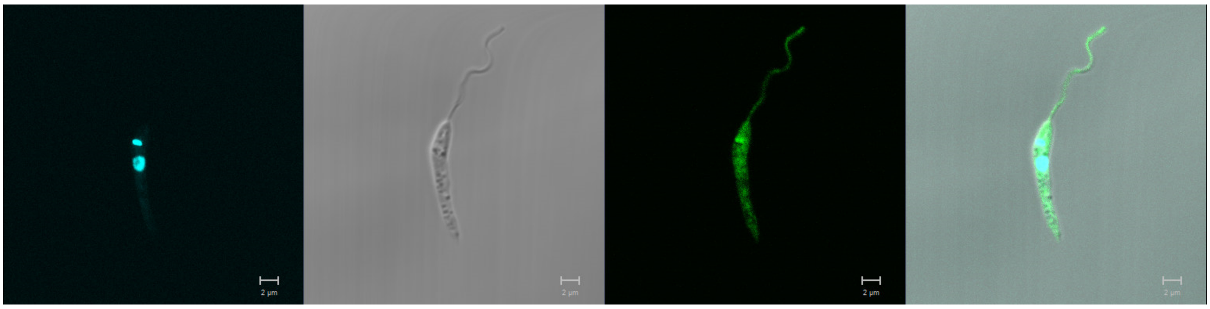

LmxM.05.0130
N-terminus

Primary localisation: nucleus

Secondary localisation: nucleus, cytoplasm, flagellum

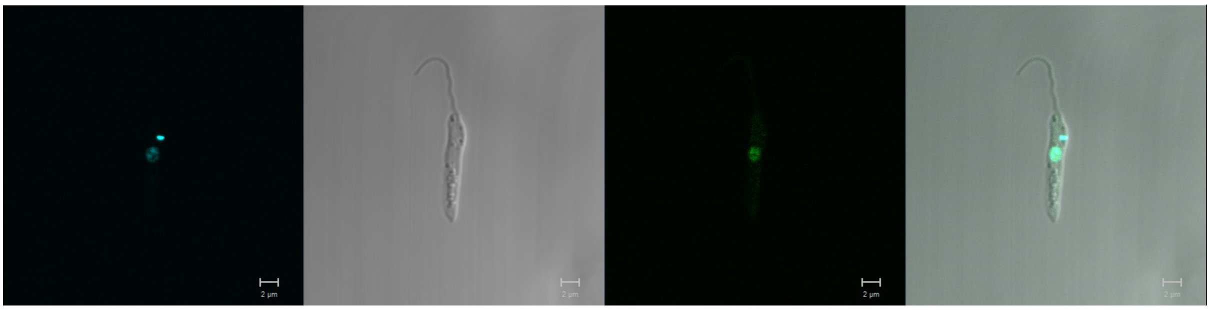

LmxM.17.0060 (mitogen-activated protein kinase kinase 3 - MAPKK3)
N-terminus

Primary localisation: cytoplasm

Secondary localisation: cytoplasm, flagellum, nucleus

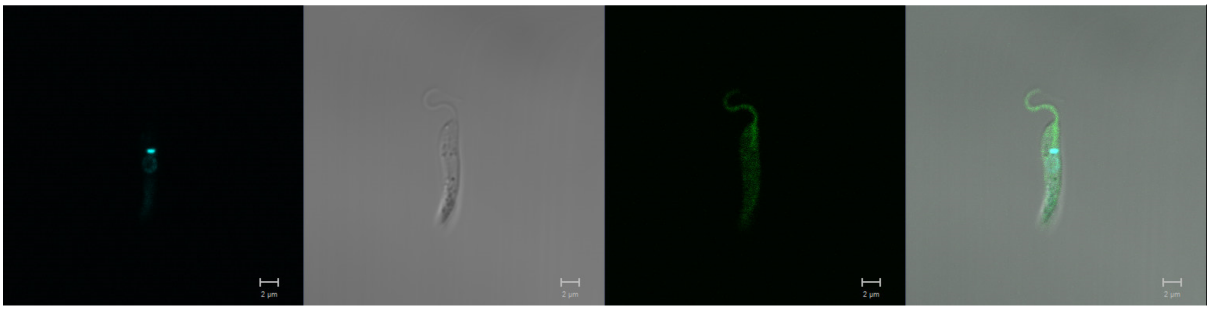

LmxM.19.0140 (AKB1)
N-terminus

Primary localisation: basal body

Secondary localisation: cytoplasm

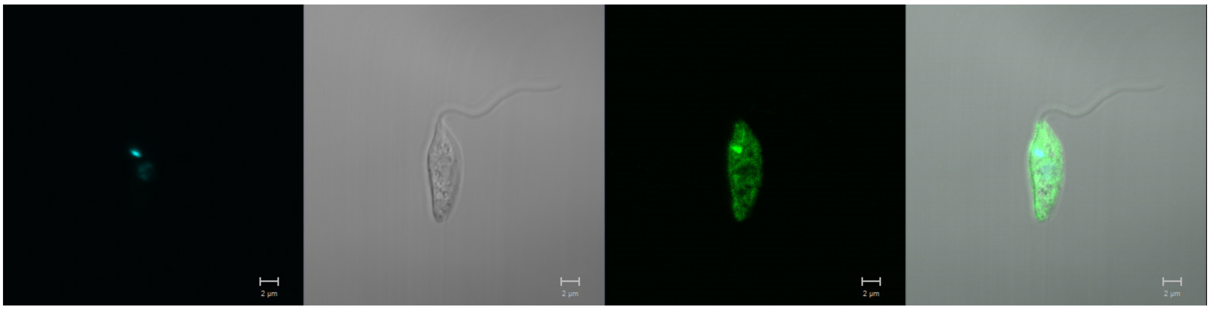

LmxM.22.0810 (SOS2)
N-terminus

Primary localisation: basal body

Secondary localisation: cytoplasm, flagellum, nucleus

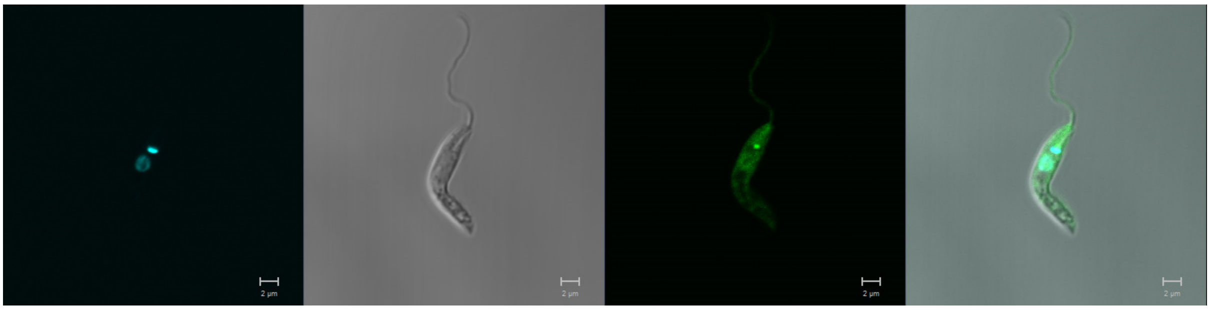

LmxM.26.2510
N-terminus

Primary localisation: cytoplasm

Secondary localisation: flagellum

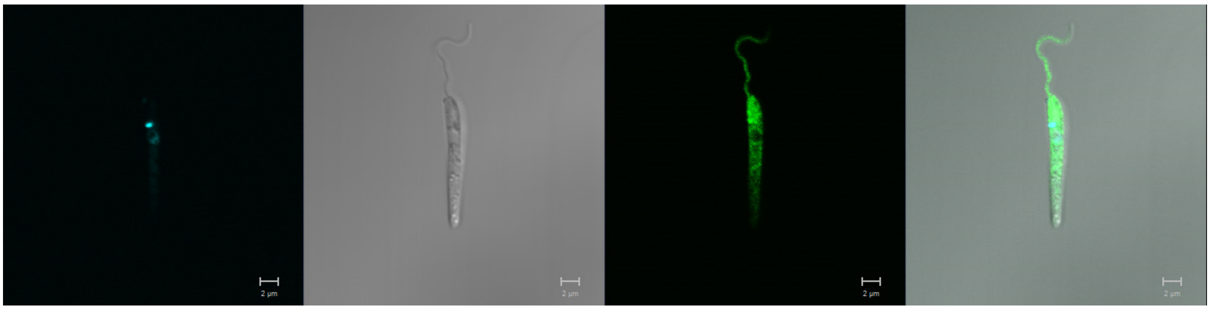

LmxM.27.2470
N-terminus

Primary localisation: basal body

Secondary localisation: cytoplasm, flagellum, nucleus

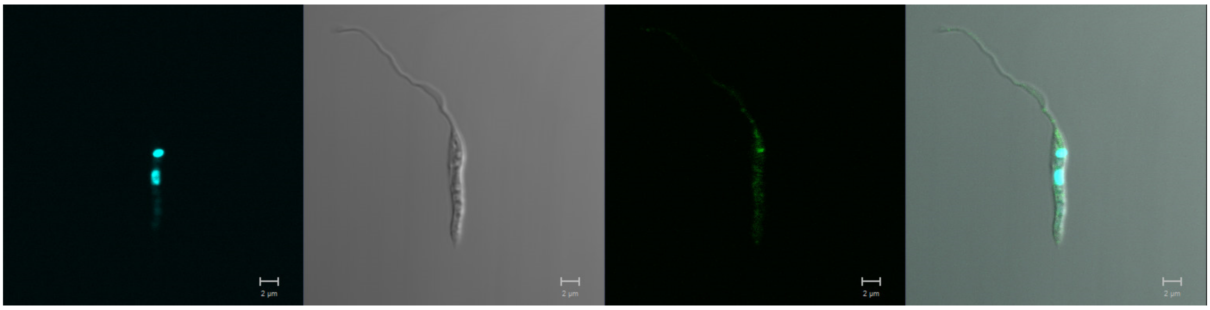

LmxM.28.2000 (LDK)
N-terminus

Primary localisation: cytoplasmic organelles (lipid droplet)

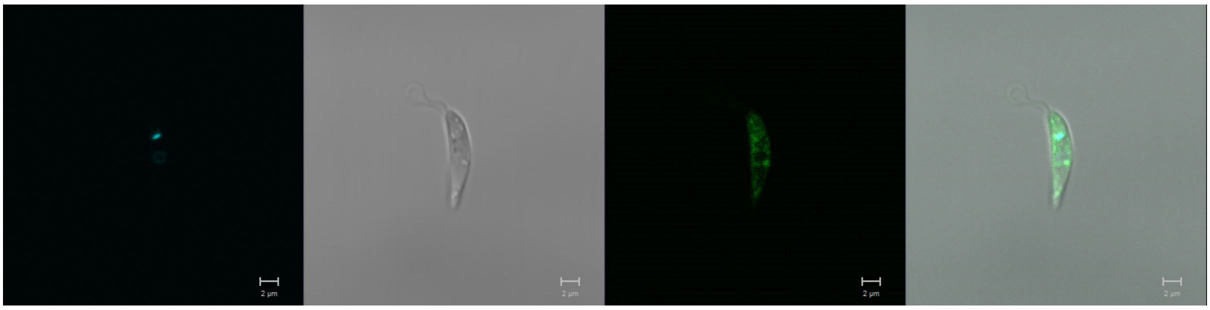

LmxM.34.1050
N-terminus

Primary localisation: cytoplasm

Secondary localisation: flagellum

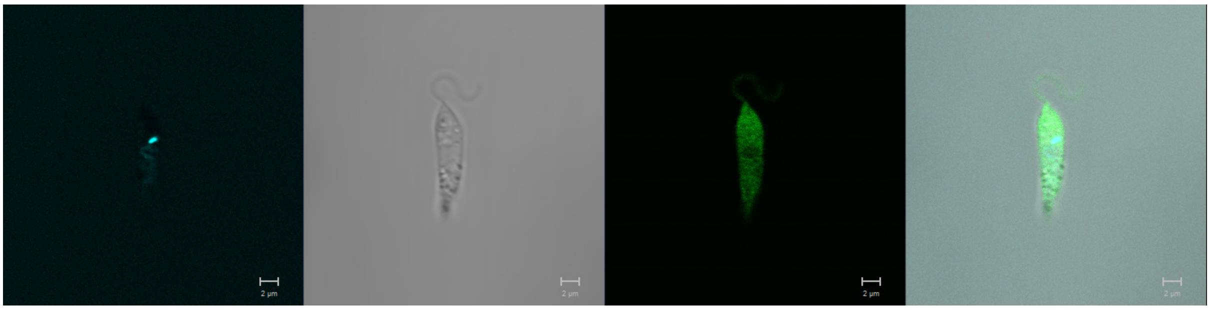

LmxM.07.0900
N-terminus

Primary localisation: basal body

Secondary localisation: cytoplasm, flagellum, basal body

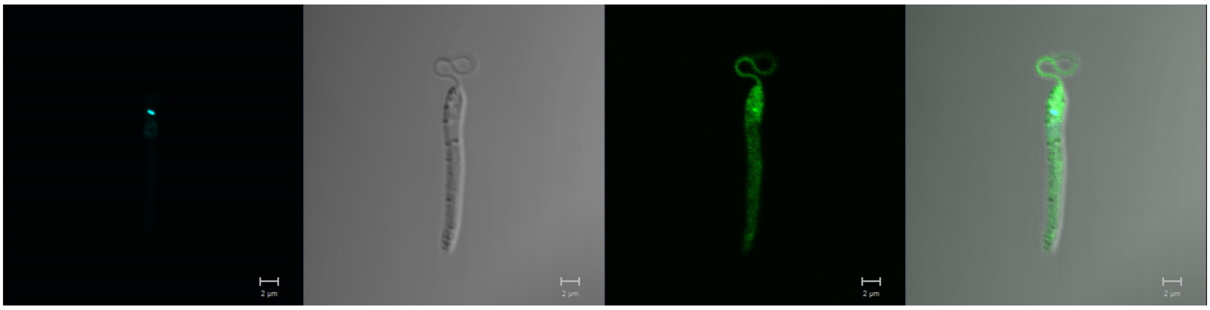

LmxM.18.0640
N-terminus

Primary localisation: basal body

Secondary localisation: cytoplasm, flagellum

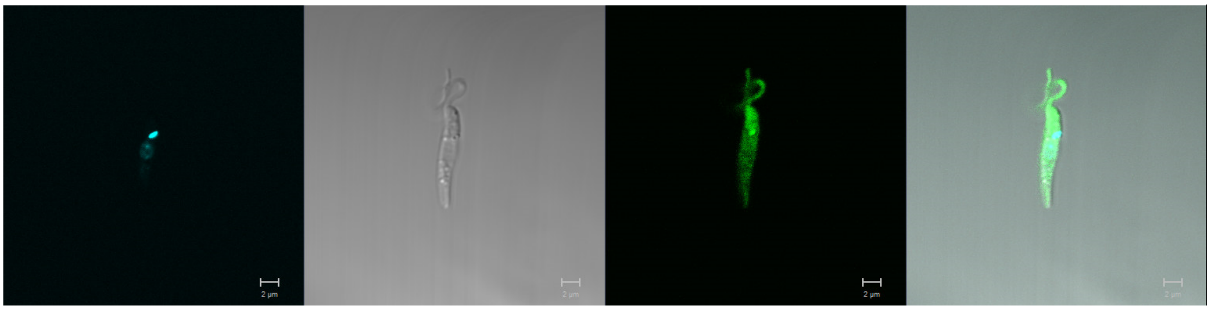

LmxM.24.0230
N-terminus

Primary localisation: nucleus

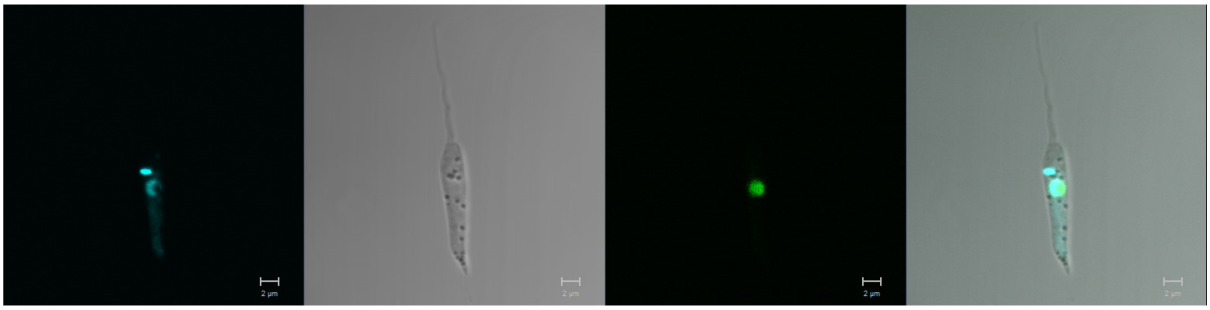

LmxM.32.1710
N-terminus

Primary localisation: cytoplasm

Secondary localisation: flagellum, nucleus, basal body

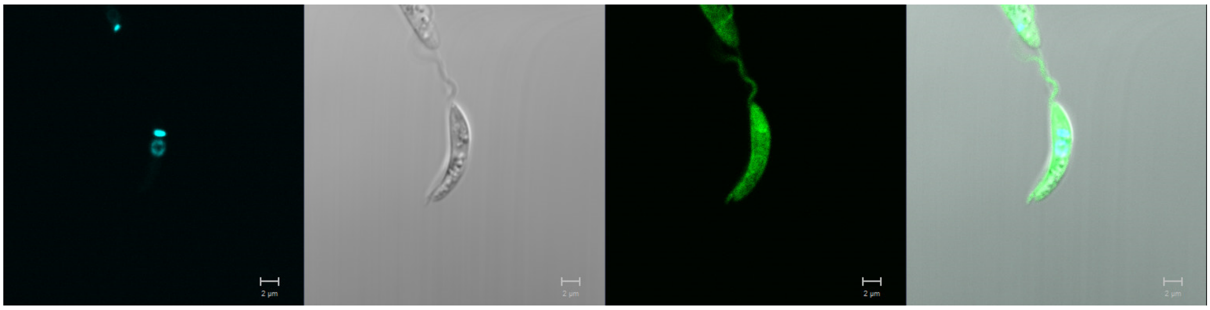

LmxM.08_29.2020 (5'-AMP-activated protein kinase catalytic subunit alpha)
N-terminus

Primary localisation: cytoplasm

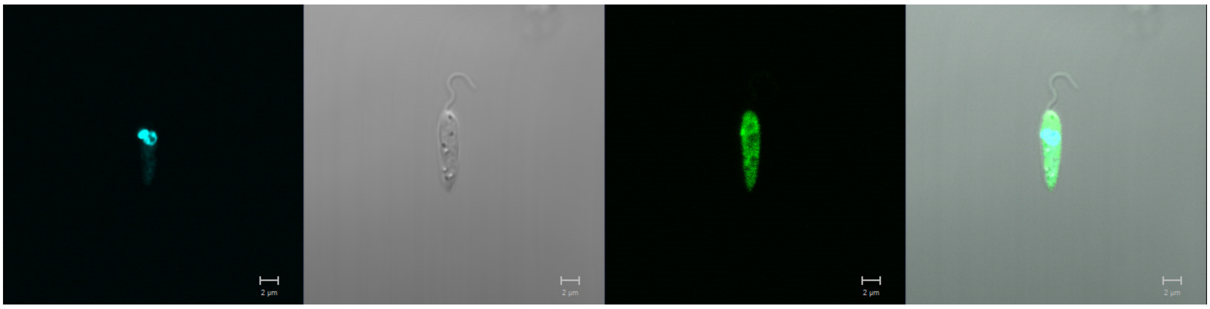

LmxM.34.0490
N-terminus

Primary localisation: cytoplasm

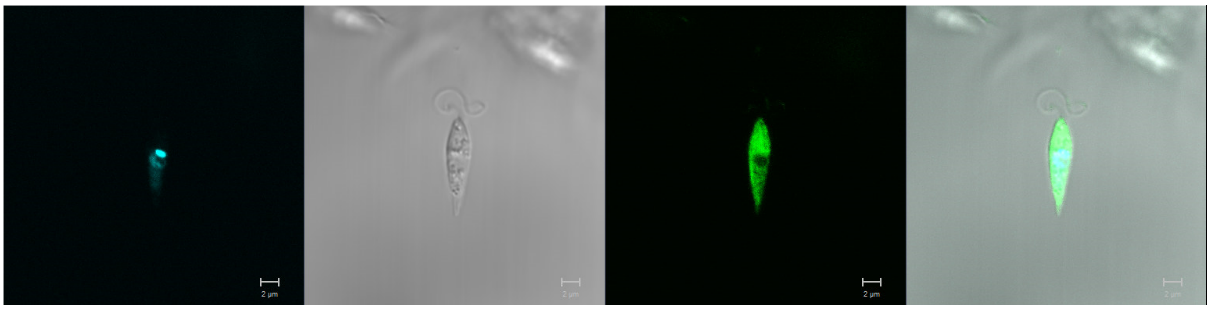

LmxM.36.0900 (SNF1-related protein kinase)

Missing – incomplete sequencing – cannot design primers.

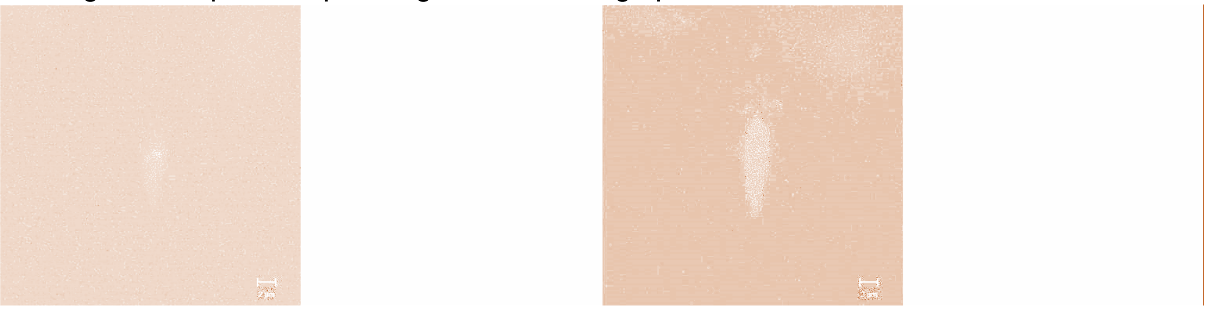

LmxM.21.0150

N-terminus

Primary localisation: flagellar pocket

Secondary localisation: cytoplasm

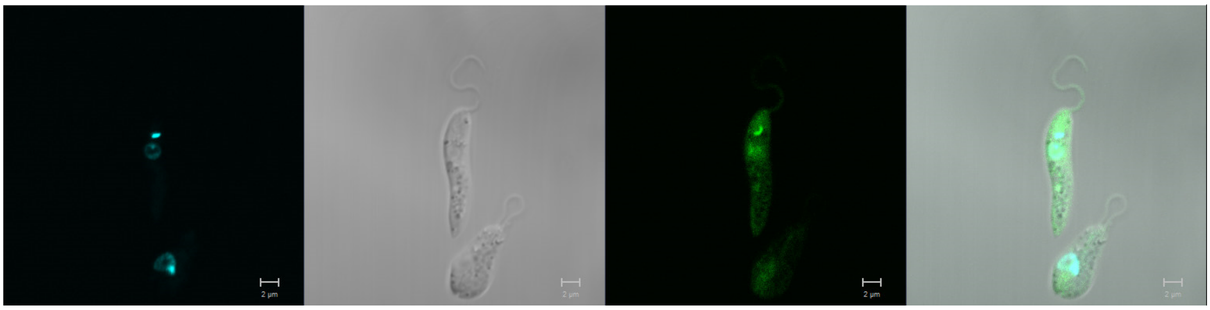

LmxM.04.0650 (LUK1)

N-terminus

Primary localisation: cytoplasm

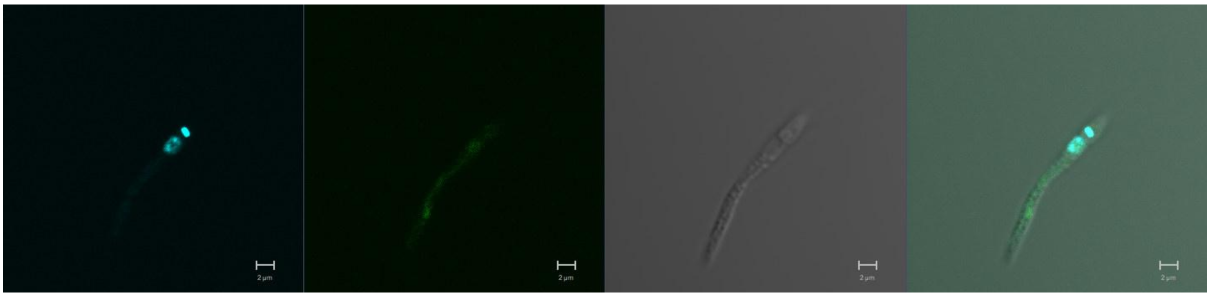

LmxM.19.1470 (LUK3)
N-terminus

Primary localisation: cytoplasm

Secondary localisation: basal body

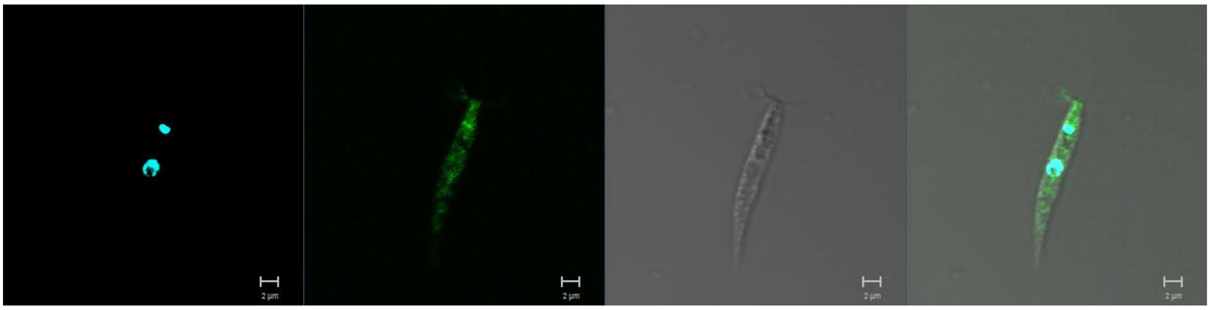

LmxM.22.1170
N-terminus

Primary localisation: basal body

Secondary localisation: flagellum, cytoplasm

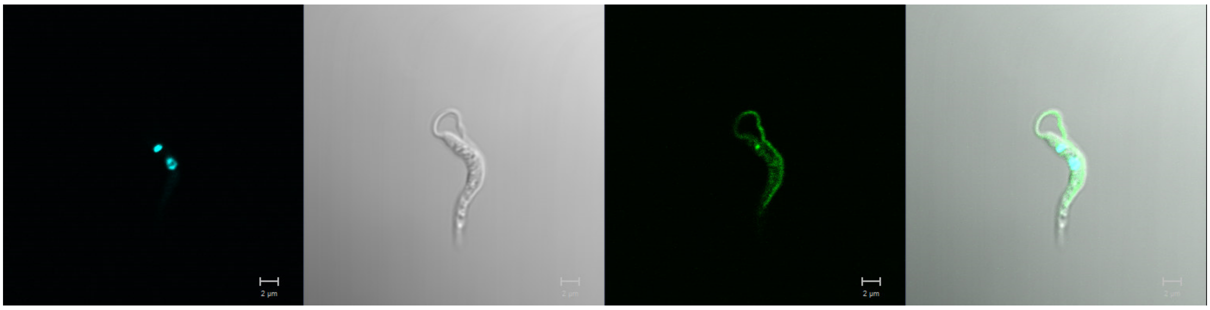

LmxM.23.0490 (AMPKβ)
N-terminus

Primary localisation: basal body

Secondary localisation: cytoplasm, basal body

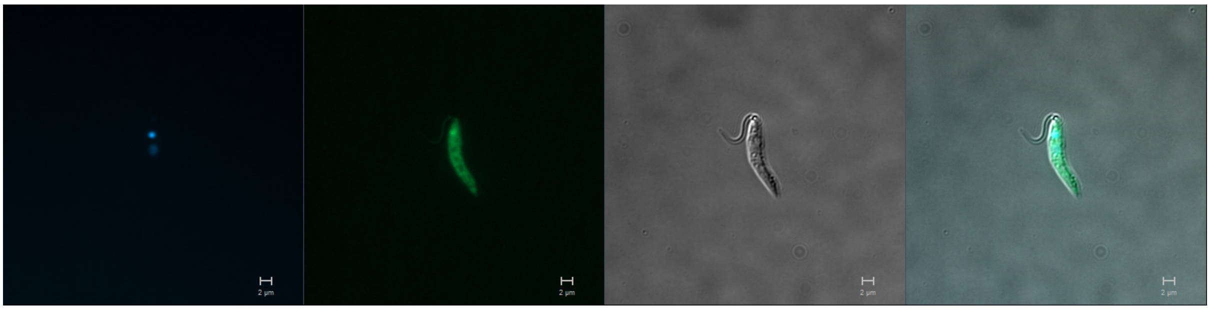

C-terminus

Primary localisation: cytoplasm

Secondary localisation: basal body

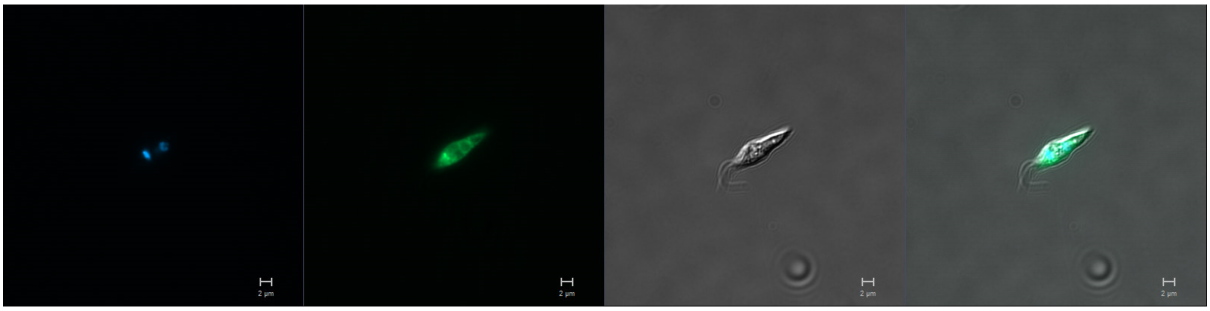

LmxM.34.0760 (AMPKγ)
N-terminus

Primary localisation: cytoplasm

Secondary localisation: basal body, flagellum

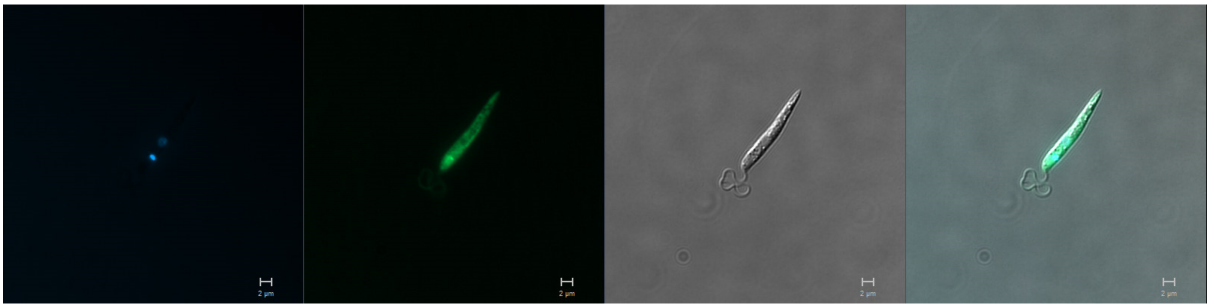

C-terminus

Primary localisation: cytoplasm

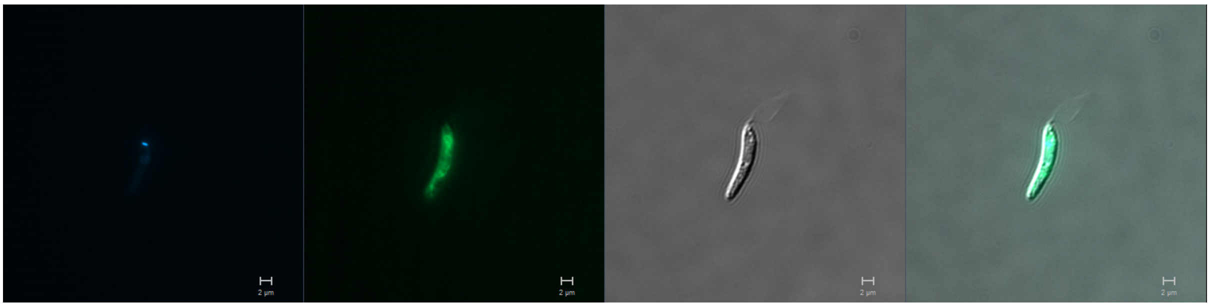

CASEIN KINASE FAMILY

LmxM.27.1780 (CK1.4)
N-terminus

Primary localisation: flagellar pocket

Secondary localisation: cytoplasm

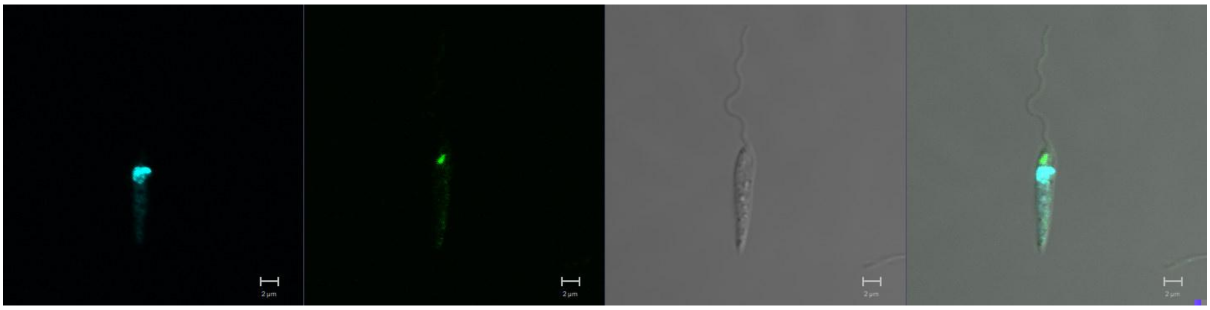

C-terminus

Primary localisation: cytoplasm

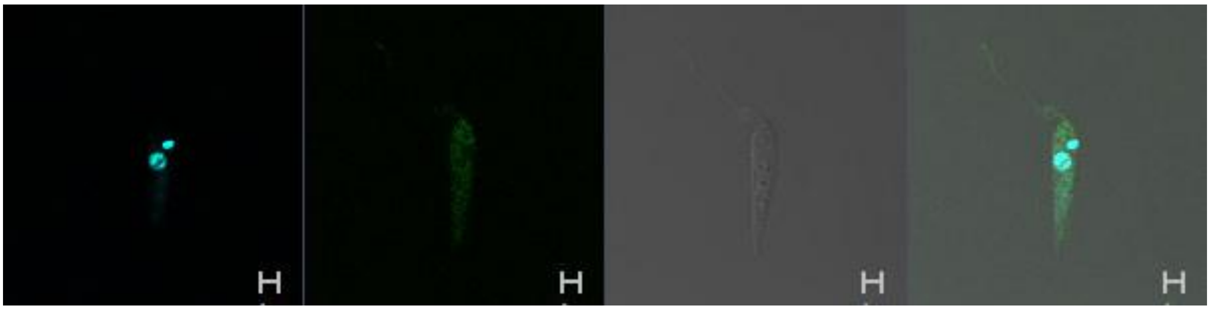

LmxM.04.1210 (CK1.3)
N-terminus

Primary localisation: no signal

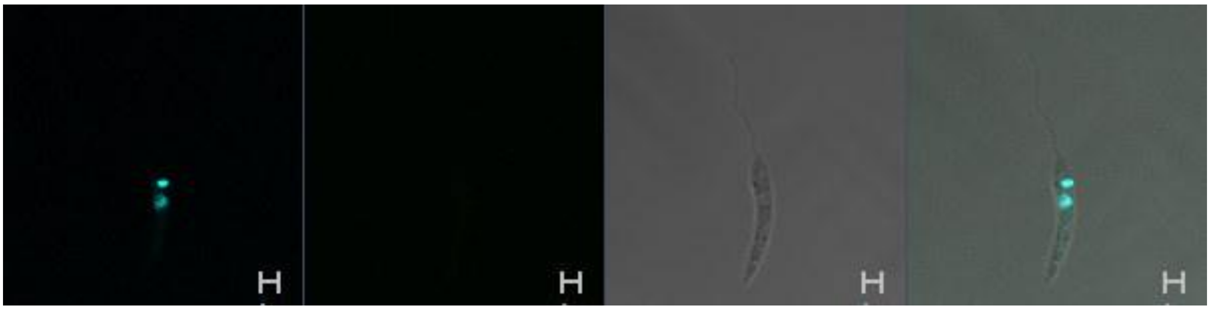

C-terminus

Primary localisation: cytoplasm

Secondary localisation: flagellum

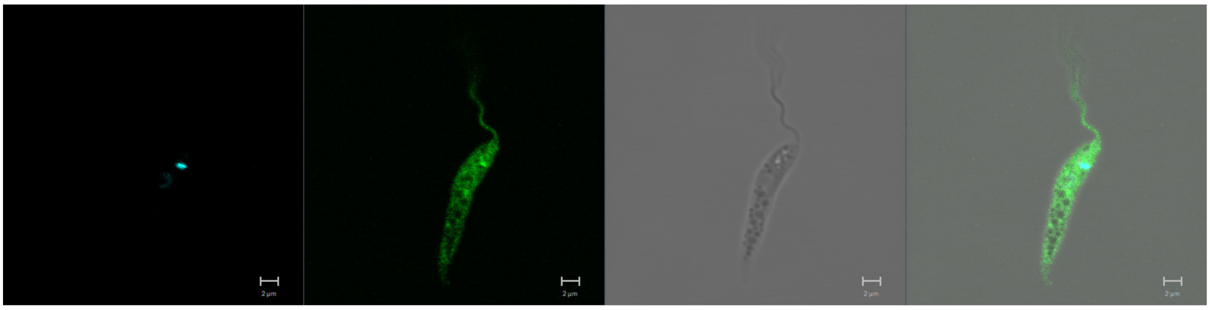

LmxM.25.1580
N-terminus

Primary localisation: flagellum

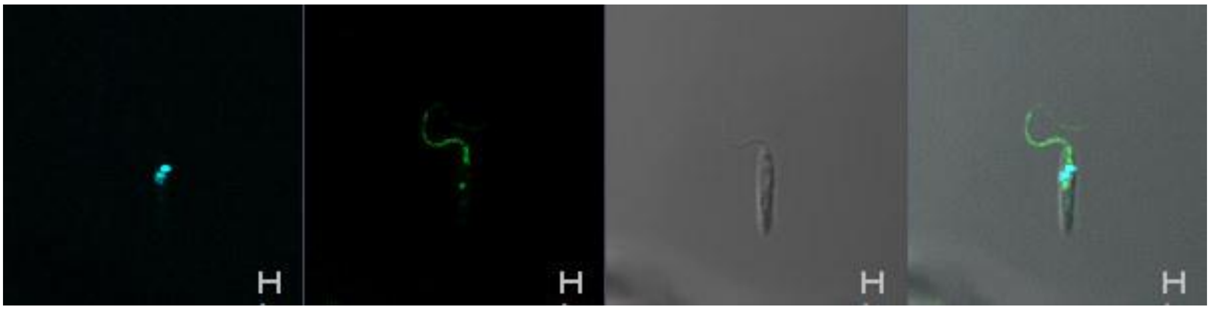

LmxM.29.3470
N-terminus

Primary localisation: cytoplasm

Secondary localisation: flagellum

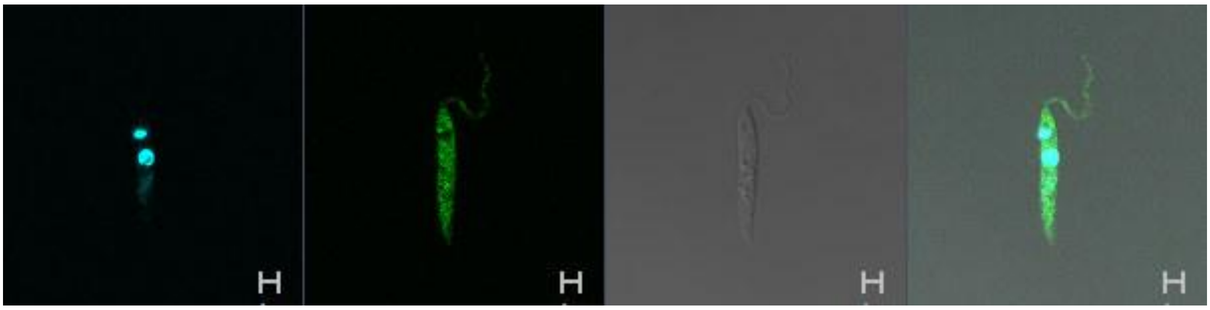

LmxM.34.1000 (CK1.1)
N-terminus

Primary localisation: cytoplasm

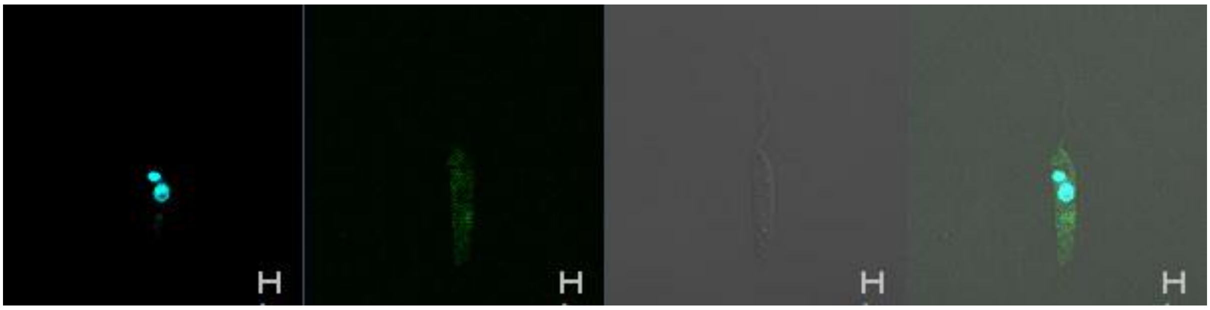

LmxM.34.1010 (CK1.2)
N-terminus

Primary localisation: cytoplasm

Secondary localisation: flagellum, basal body, nucleus

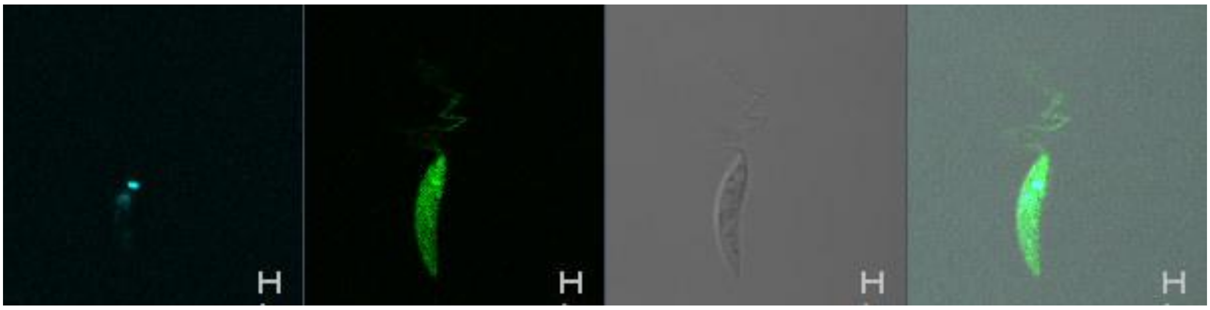

LmxM.33.3020
N-terminus

Primary localisation: flagellar pocket

Secondary localisation: cytoplasm

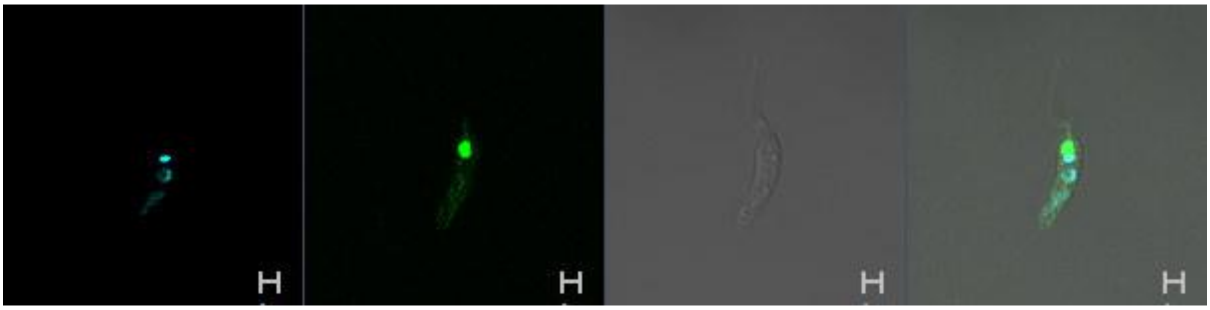

<u>CMGC FAMILY</u>

LmxM.24.0670
N-terminus

Primary localisation: nucleus

Secondary localisation: kinetoplast, cytoplasm

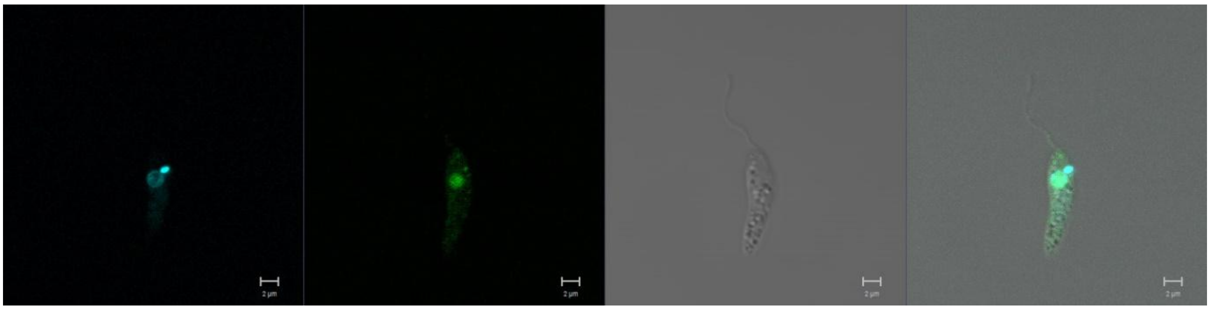

LmxM.32.2070 (MPK15)
N-terminus

Primary localisation: cytoplasm

Secondary localisation: cytoplasmic organelles, flagellum, nucleus

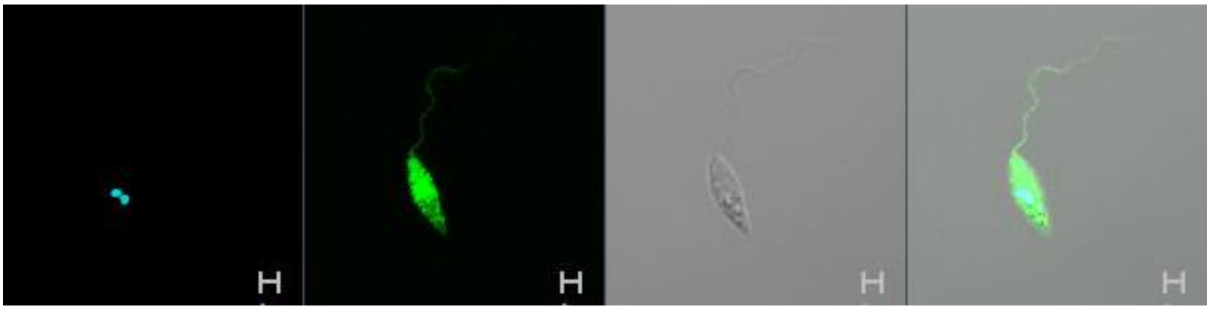

LmxM.26.0040 (CRK7)
N-terminus

Primary localisation: cytoplasm

Secondary localisation: flagellum, nucleus

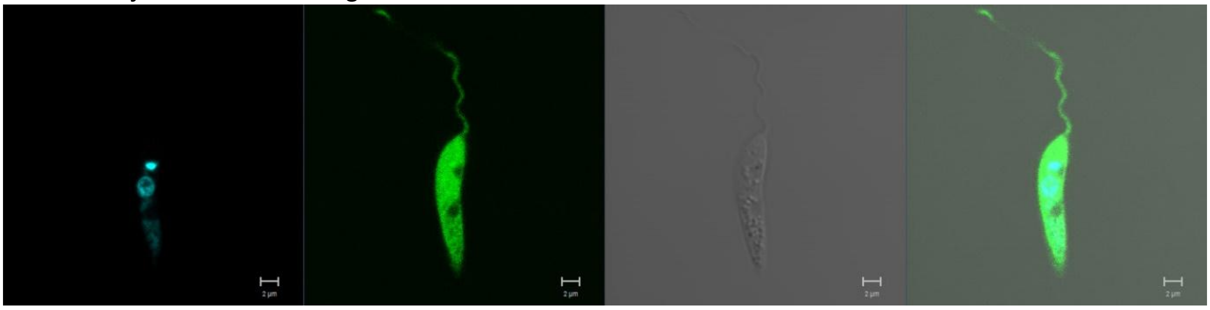

LmxM.05.0550 (CRK2)
N-terminus

Primary localisation: cytoplasm

Secondary localisation: nucleus, flagellum

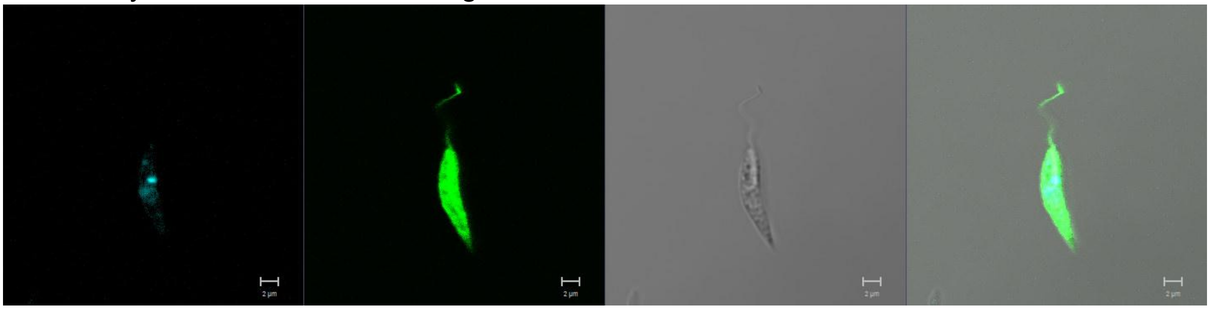

LmxM.09.0310 (CRK12)
N-terminus

Primary localisation: nucleus

Secondary localisation: kinetoplast, cytoplasm

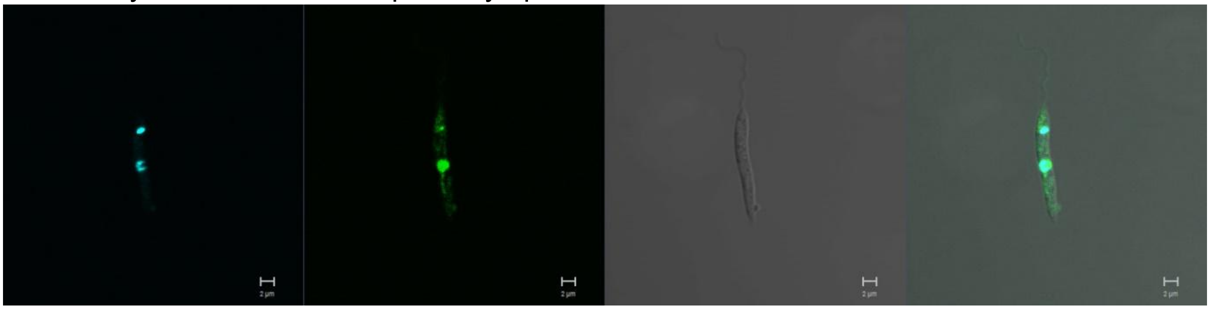

LmxM.11.0110 (CRK8)
N-terminus

Primary localisation: nucleus

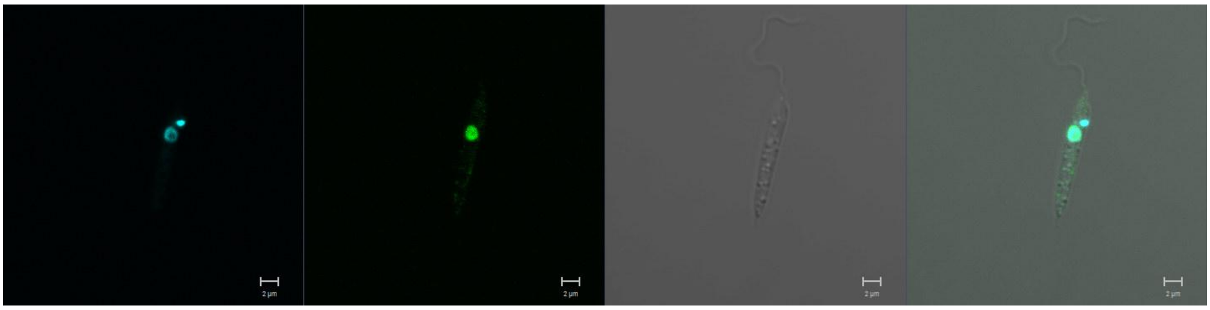

LmxM.16.0990 (CRK4)
N-terminus

Primary localisation: cytoplasm

Secondary localisation: flagellum

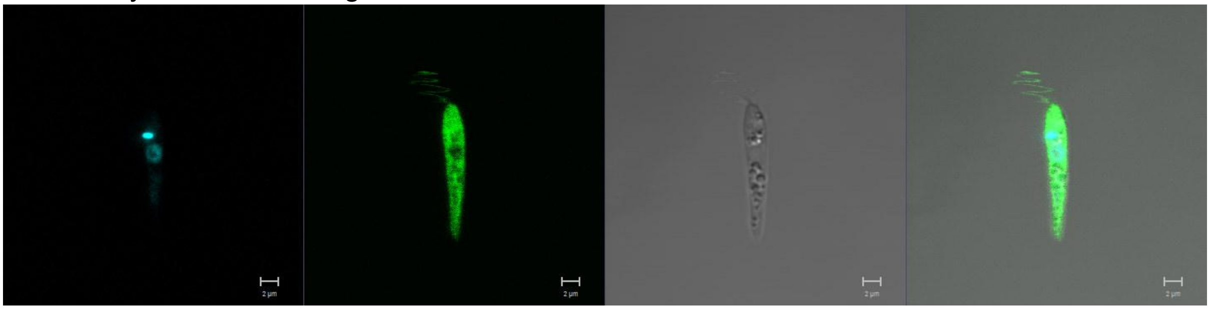

LmxM.21.1080 (CRK1)
N-terminus

Primary localisation: mitochondrion

Secondary localisation: cytoplasm

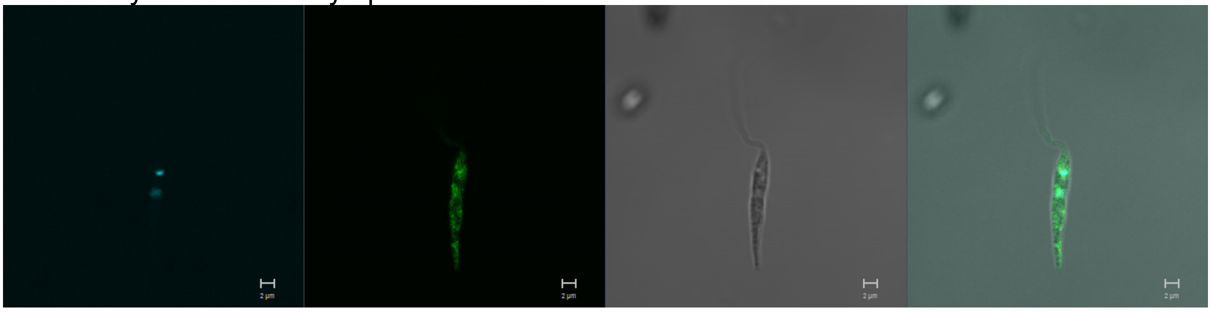

C-terminus

Primary localisation: mitochondrion

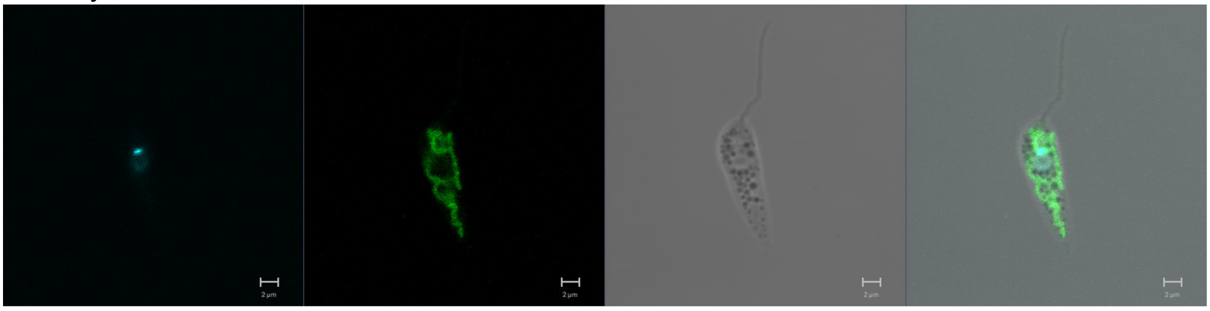

LmxM.27.0560 (CRK6)
N-terminus

Primary localisation: cytoplasm

Secondary localisation: flagellum, basal body

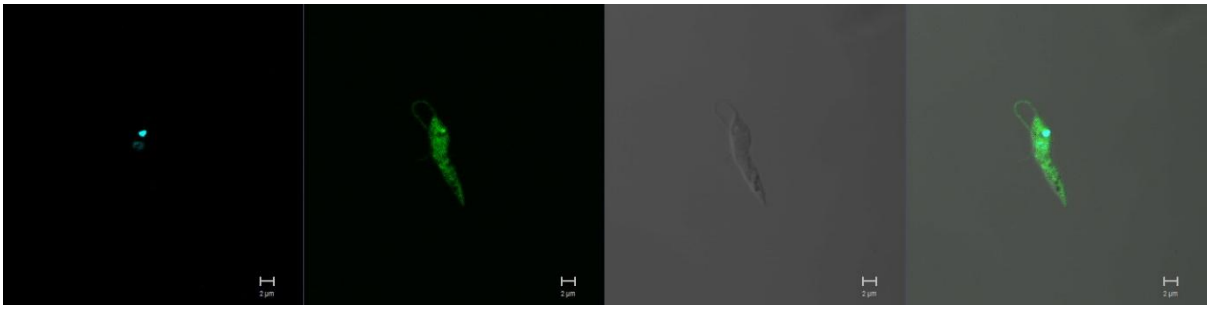

LmxM.27.1940 (CRK9)
C-terminus

Primary localisation: nucleus

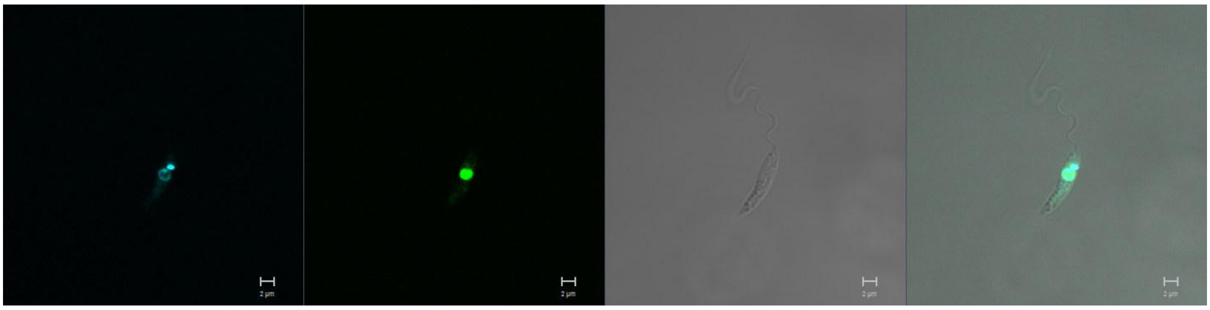

LmxM.08_29.2150 (CRK10)
N-terminus

Primary localisation: nucleus

Secondary localisation: kinetoplast, cytoplasm

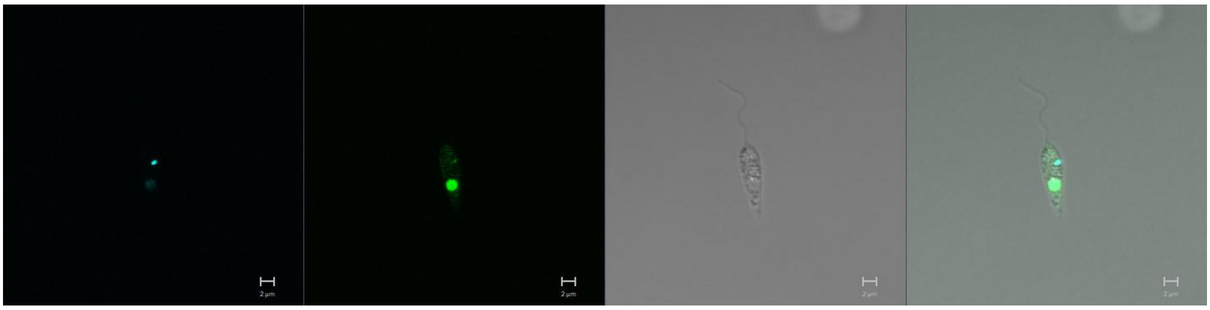

LmxM.29.1780 (CRK11)
C-terminus

Primary localisation: nucleus

Secondary localisation: cytoplasm

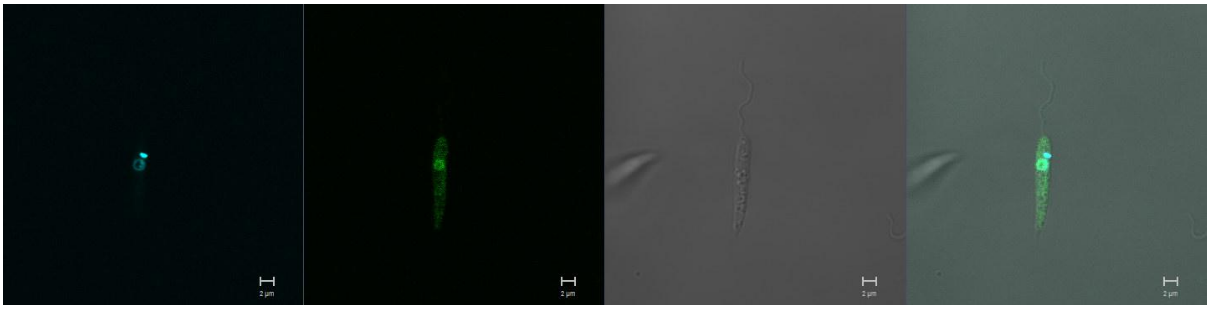

LmxM.36.0550 (CRK3)
N-terminus

Primary localisation: cytoplasm

Secondary localisation: flagellum, nucleus, kinetoplast

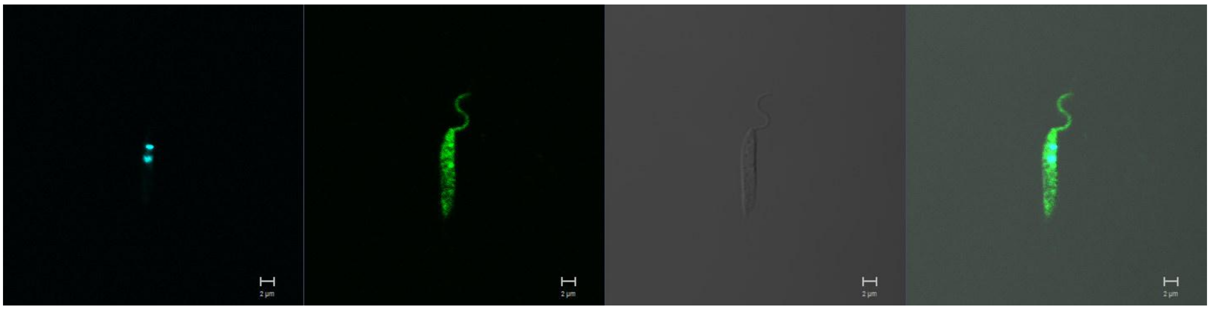

LmxM.09.0400 (CLK1/KKT10)
N-terminus

Primary localisation: nucleus

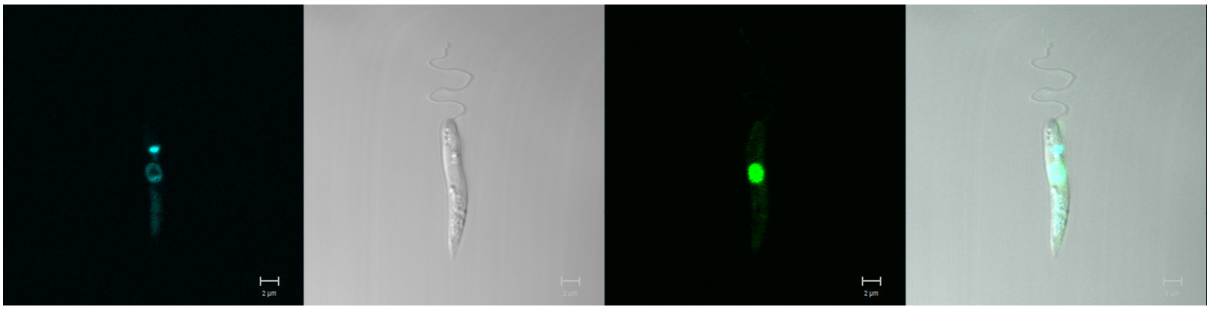

C-terminus

Primary localisation: nucleus

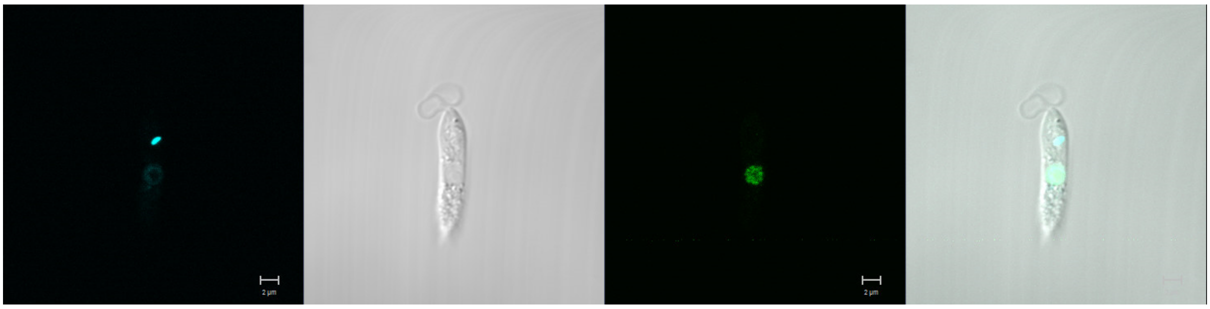

LmxM.09.0410 (CLK2/KKT19)
N-terminus

Primary localisation: nucleus

Secondary localisation: cytoplasm (only during division), kinetoplast

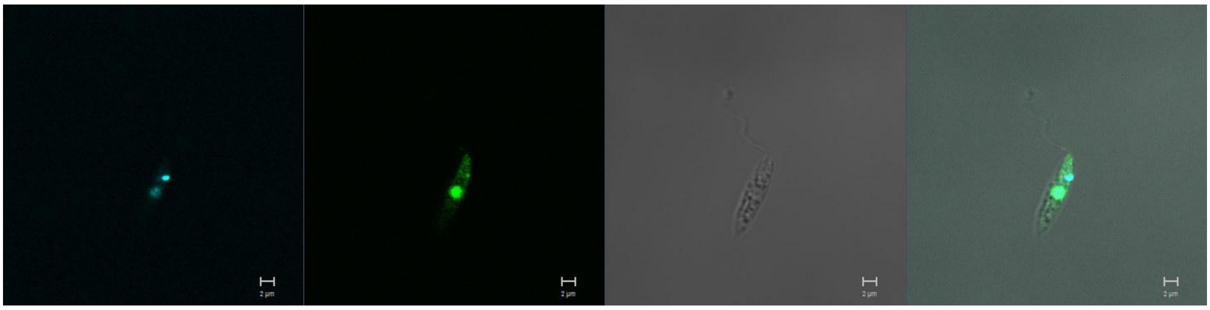

Field of view of dividing cells expressing CLK2/KKT19 in the cytoplasm (white arrows)

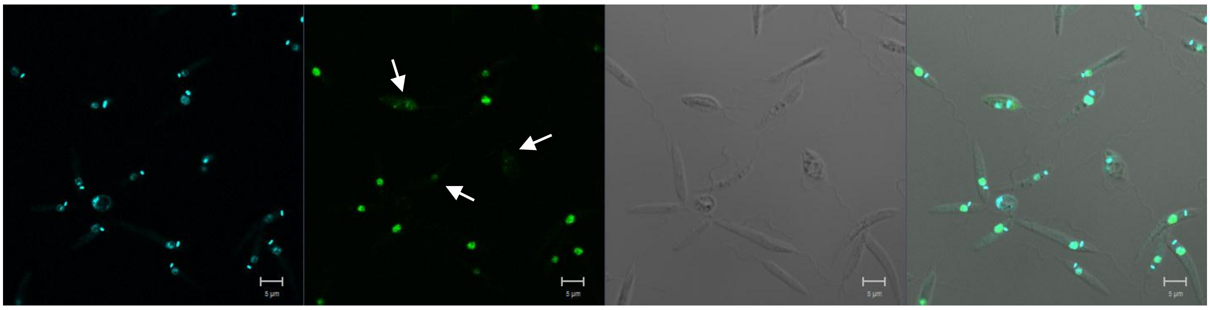

LmxM.25.1560
N-terminus

Primary localisation: cytoplasm

Secondary localisation: nucleus, kinetoplast

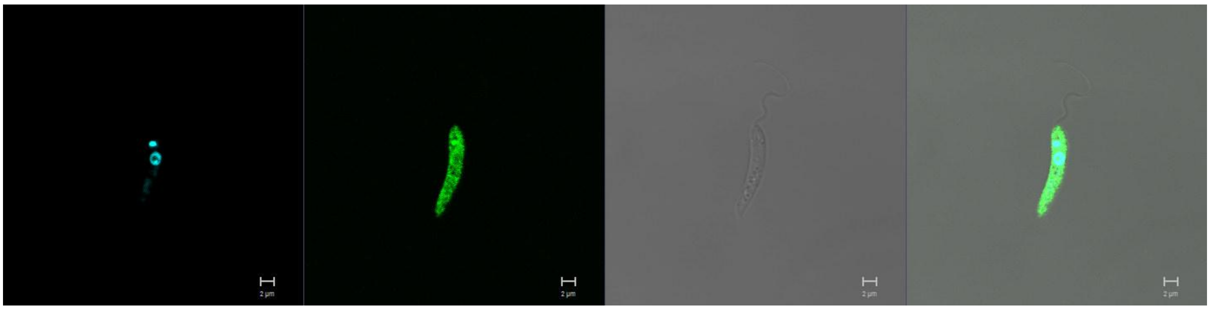

LmxM.27.1800
C-terminus

Primary localisation: endoplasmic reticulum

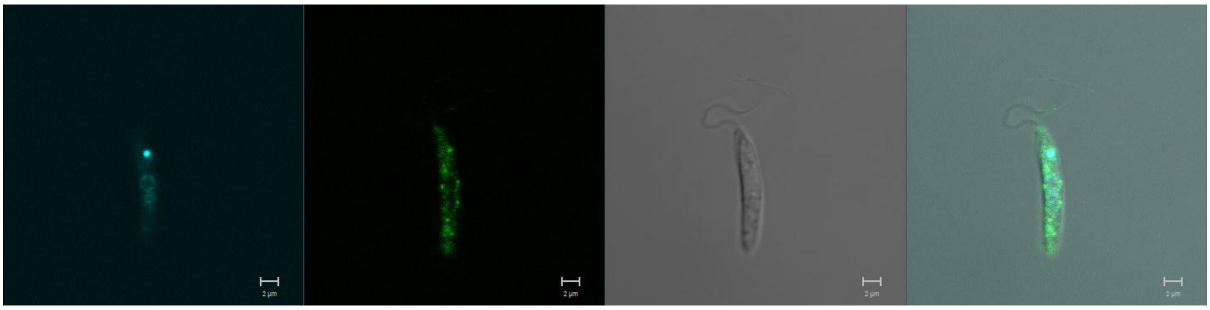

LmxM.14.0830
N-terminus

Primary localisation: basal body

Secondary localisation: cytoplasm

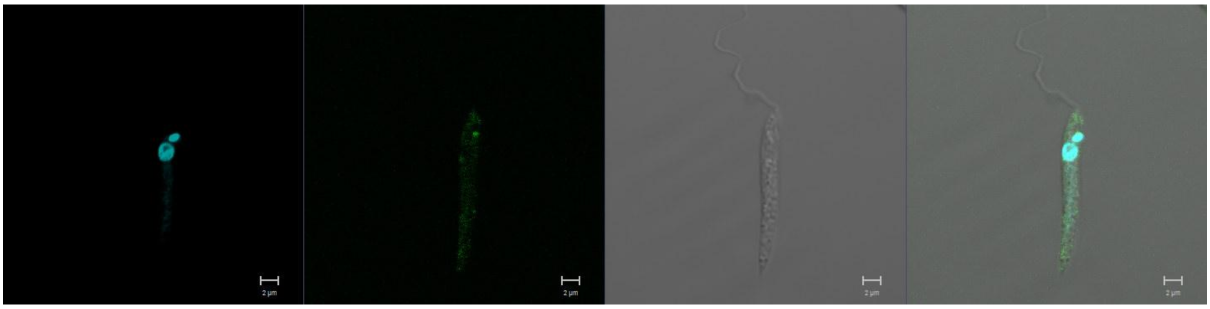

LmxM.14.1070
N-terminus

Primary localisation: basal body

Secondary localisation: flagellar pocket, lysosome, cytoplasm

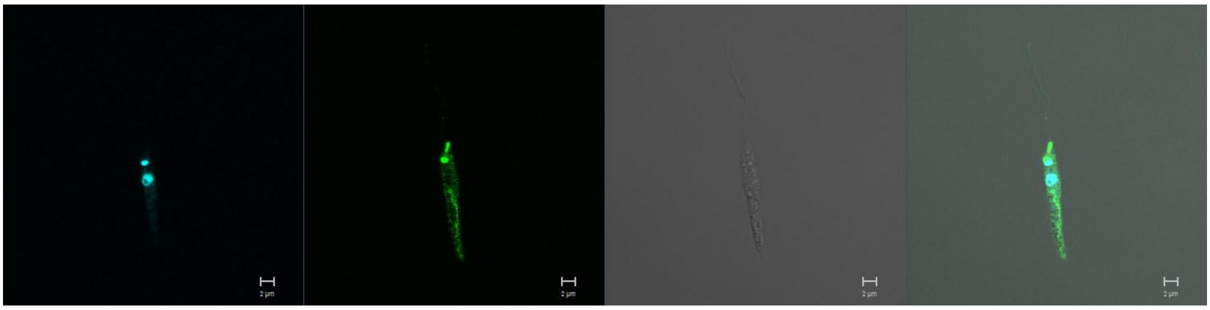

LmxM.15.0180 (DYRK1)
N-terminus

Primary localisation: lysosome

Secondary localisation: cytoplasm, endomembrane

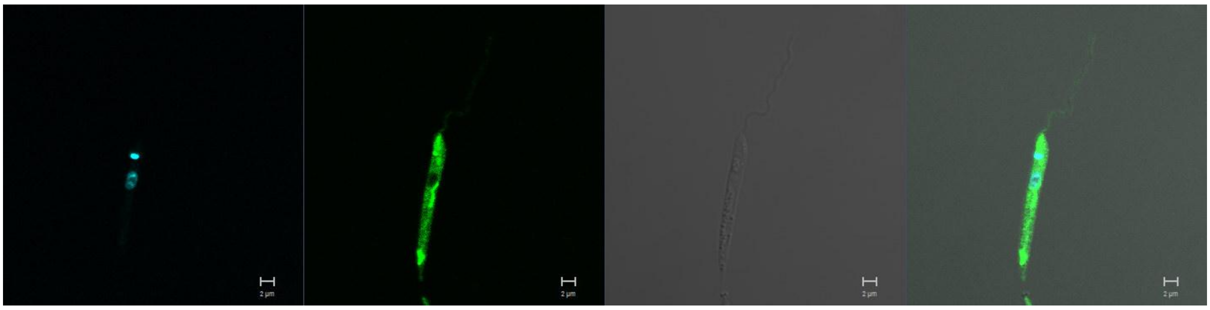

LmxM.19.0360
N-terminus

Primary localisation: cytoplasm

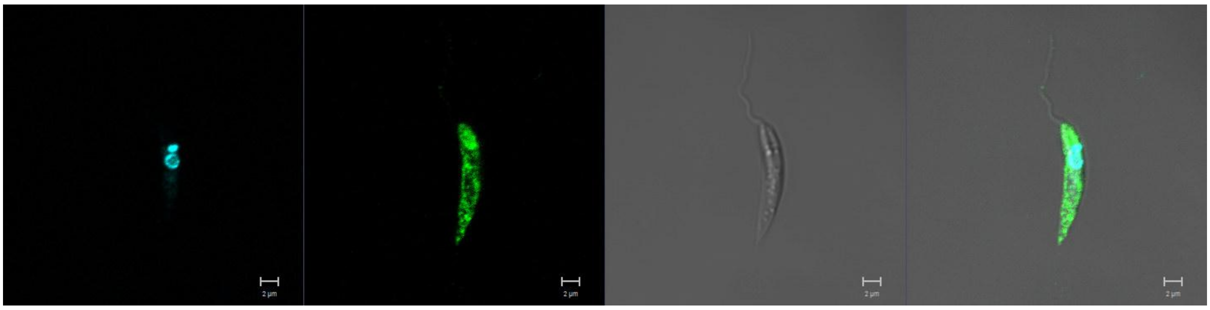

LmxM.21.1650 (PK4)
N-terminus

Primary localisation: cytoplasm (low expression)

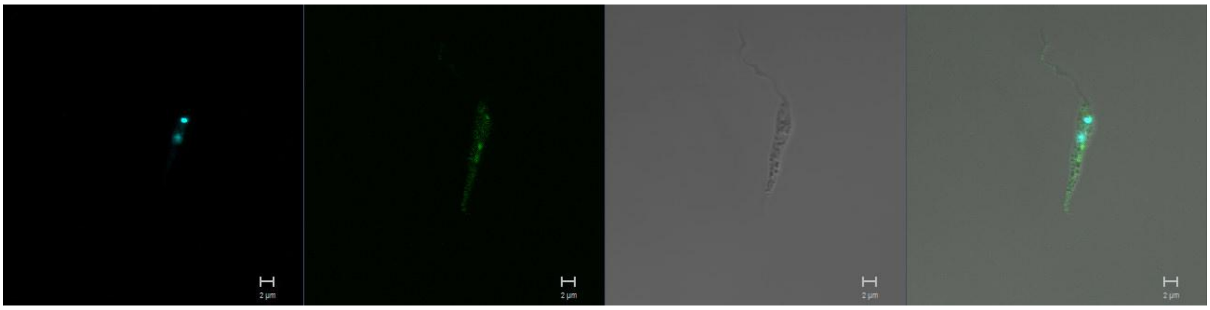

LmxM.34.1860
N-terminus

Primary localisation: flagellar pocket

Secondary localisation: basal body, cytoplasm, flagellum

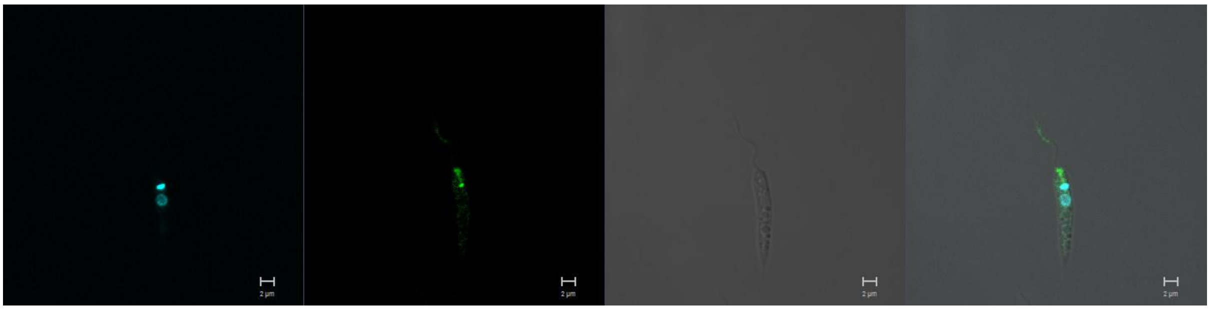

LmxM.32.1830 (DYRK2)
N-terminus

Primary localisation: cytoplasm (low expression)

Secondary localisation: flagellum (low expression)

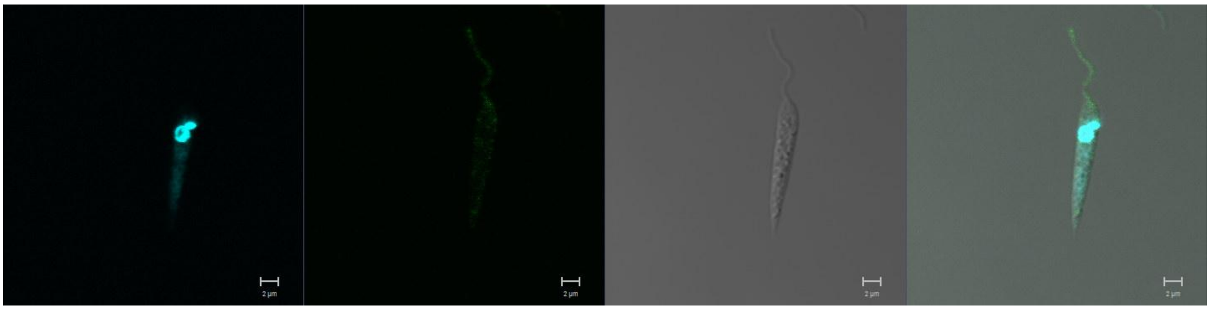

LmxM.36.4250
N-terminus

Primary localisation: no signal

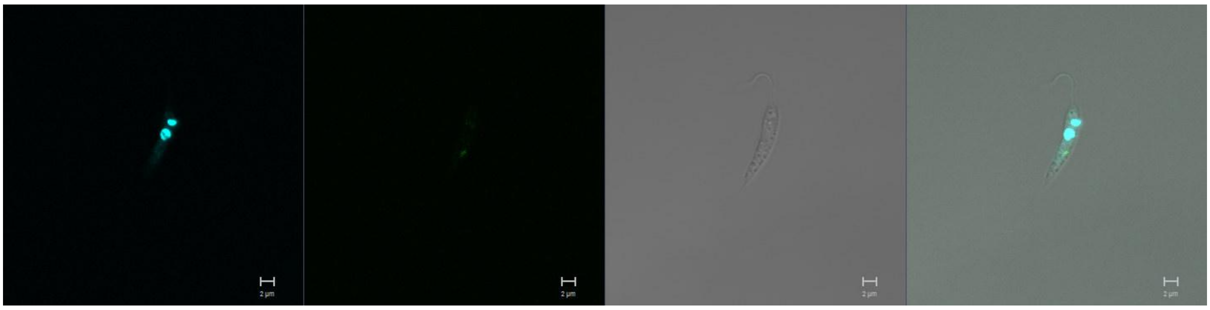

C-terminus

Primary localisation: no signal

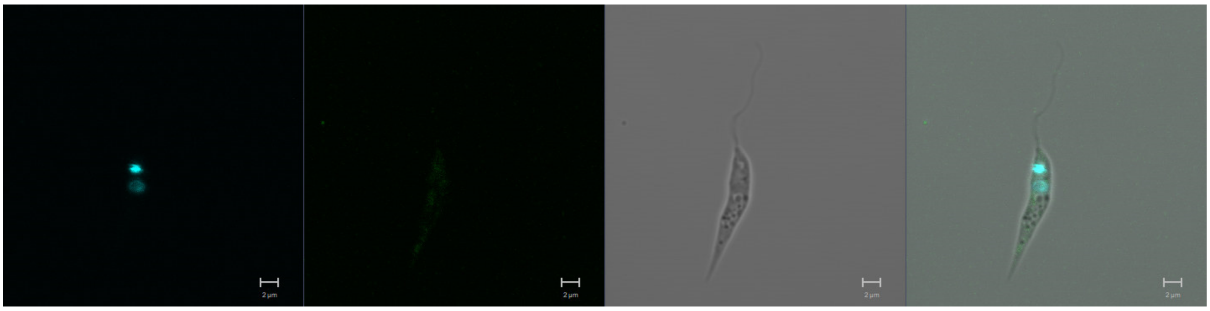

LmxM.18.0270 (GSK3)
N-terminus

Primary localisation: pellicular membrane

Secondary localisation: cytoplasm

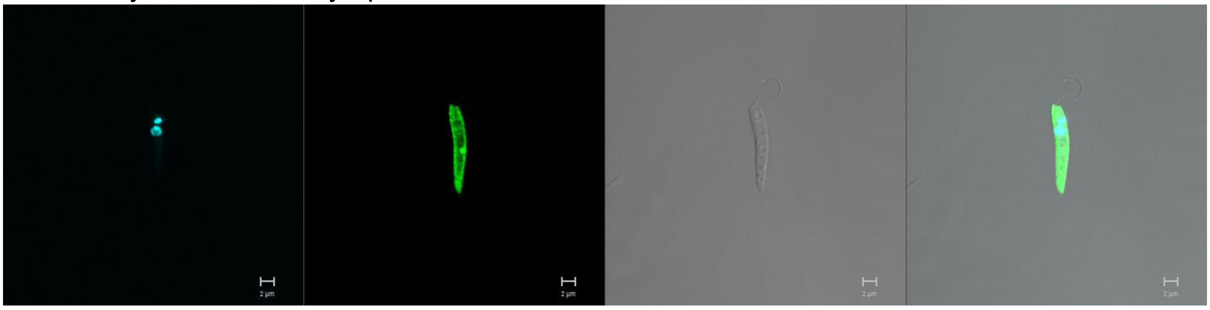

LmxM.22.0490 (GSKA)
N-terminus

Primary localisation: no signal

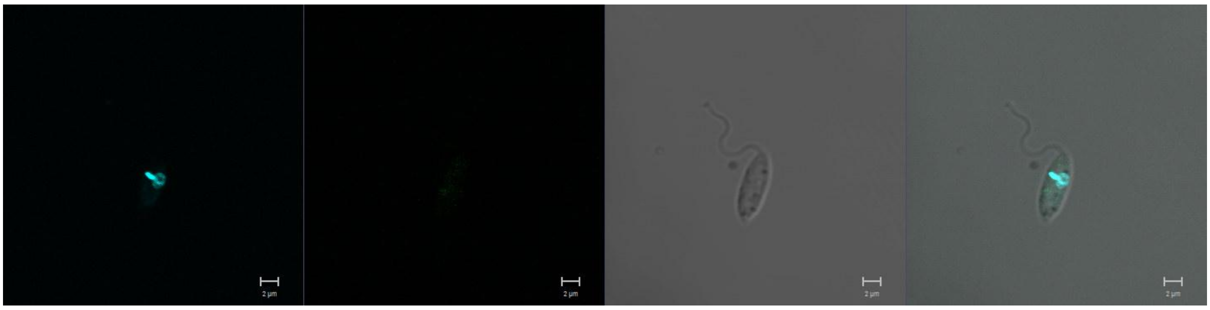

C-terminus

Primary localisation: no signal

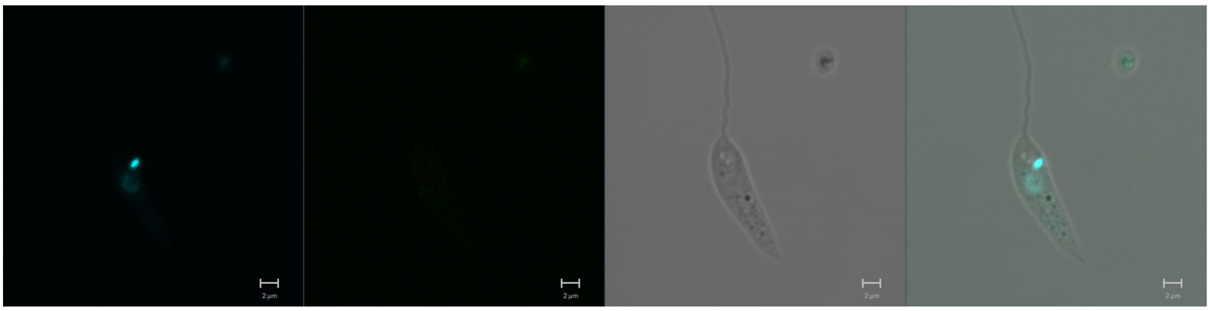

LmxM.03.0210
N-terminus

Primary localisation: cytoplasm

Secondary localisation: nucleus, flagellum

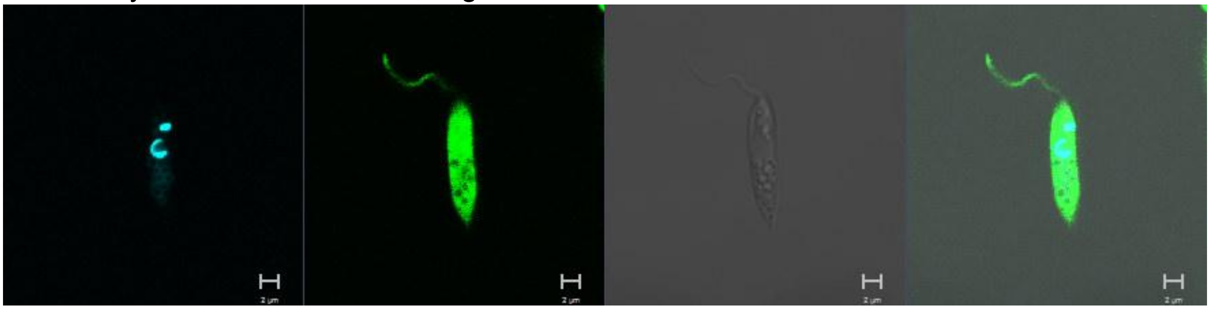

LmxM.10.0200 (MPK10)
N-terminus

Primary localisation: cytoplasm

Secondary localisation: flagellum, nucleus

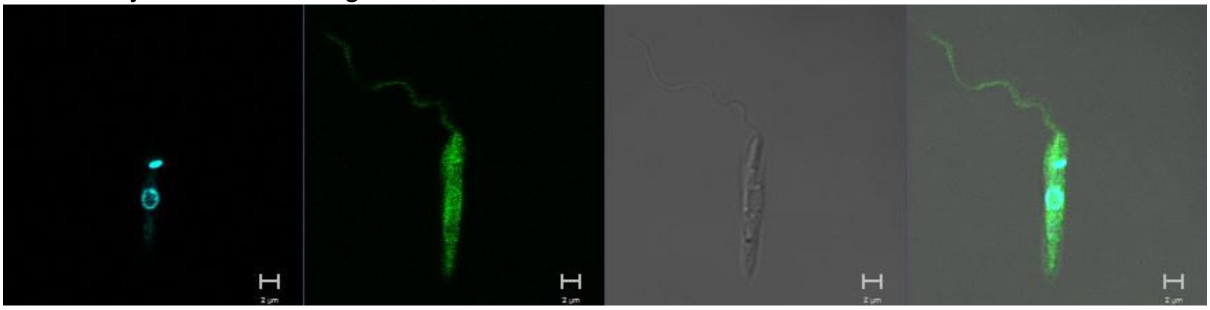

LmxM.10.0490 (MPK3)
N-terminus

Primary localisation: cytoplasm

Secondary localisation: flagellum

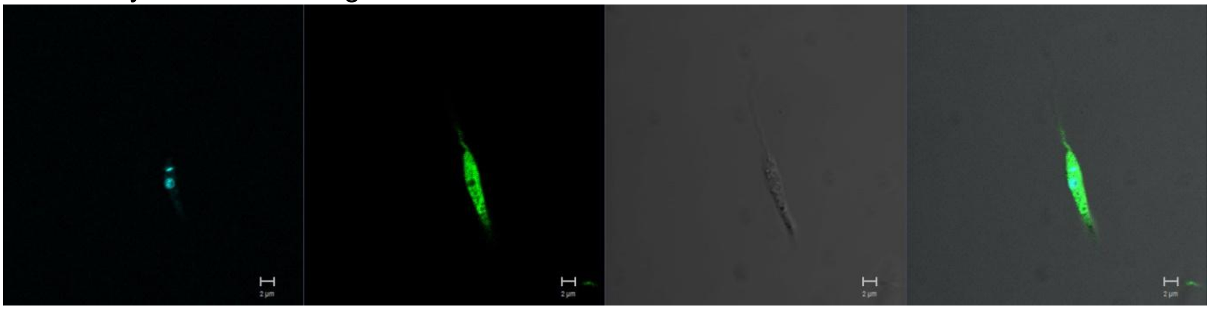

LmxM.13.0780
N-terminus

Primary localisation: cytoplasm

Secondary localisation: flagellum

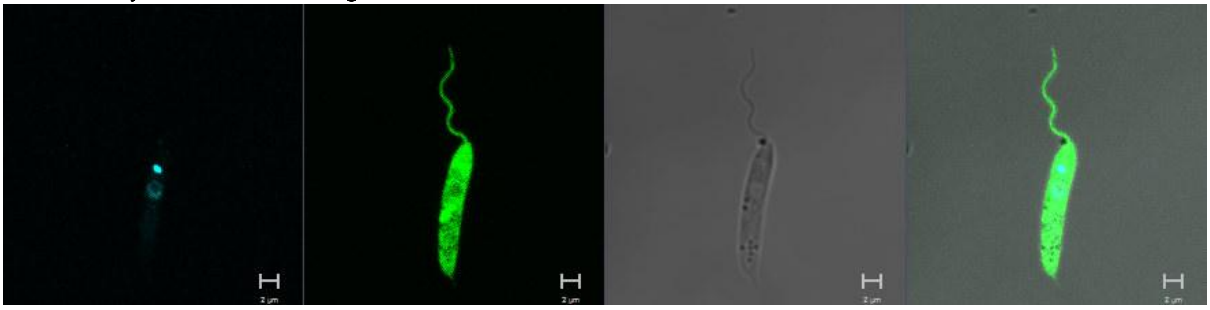

LmxM.13.1640 (MPK7)
N-terminus

Primary localisation: cytoplasm

Secondary localisation: nucleus, flagellum, basal body

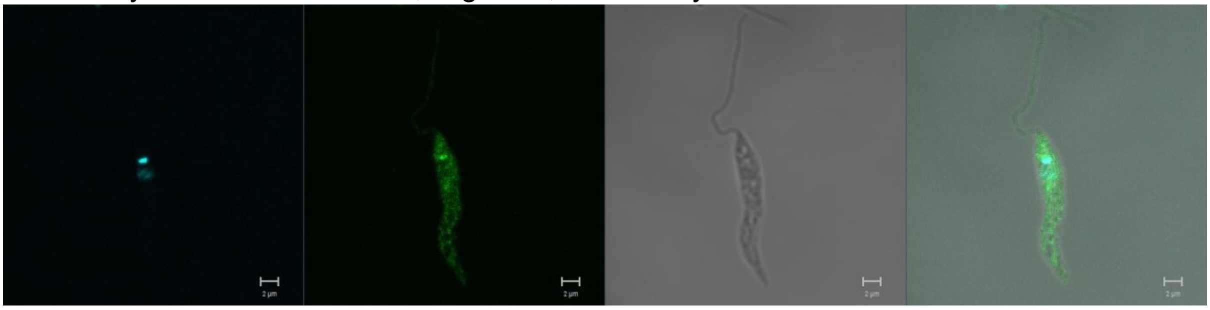

LmxM.19.1440 (MPK4)
N-terminus

Primary localisation: lysosome

Secondary localisation: cytoplasm, flagellum

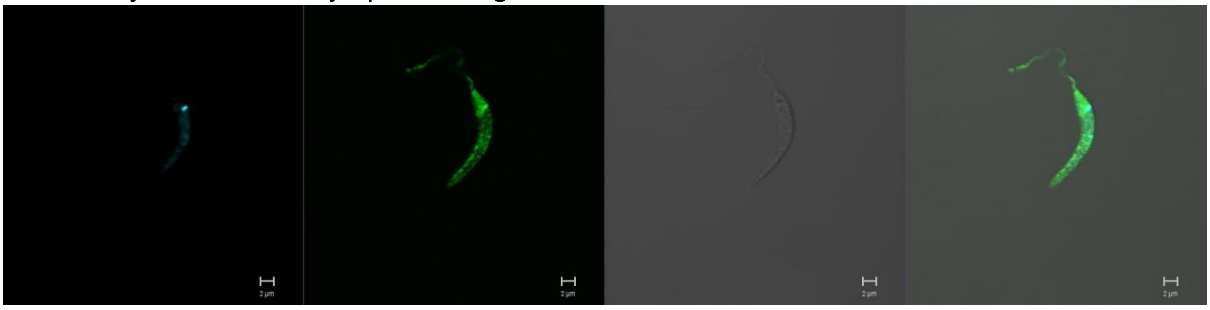

Field of view of MPK4 fluorescent mutants

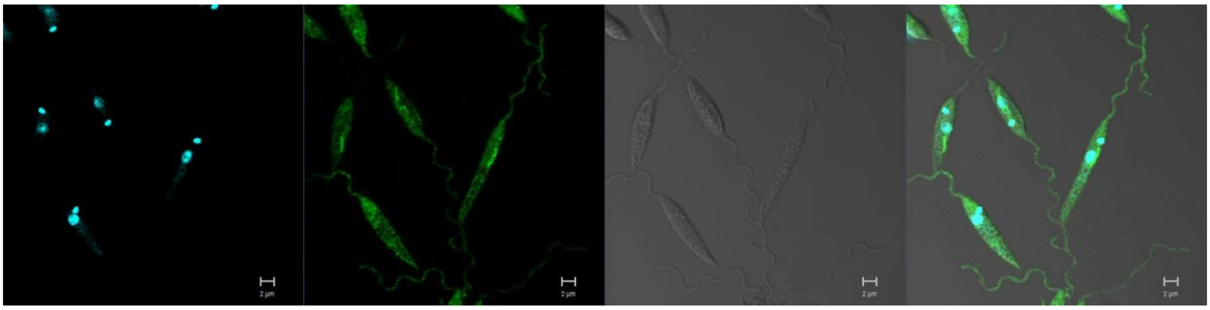

LmxM.29.0370 (MPK12)
N-terminus

Primary localisation: cytoplasm

Secondary localisation: flagellum, nucleus, kinetoplast

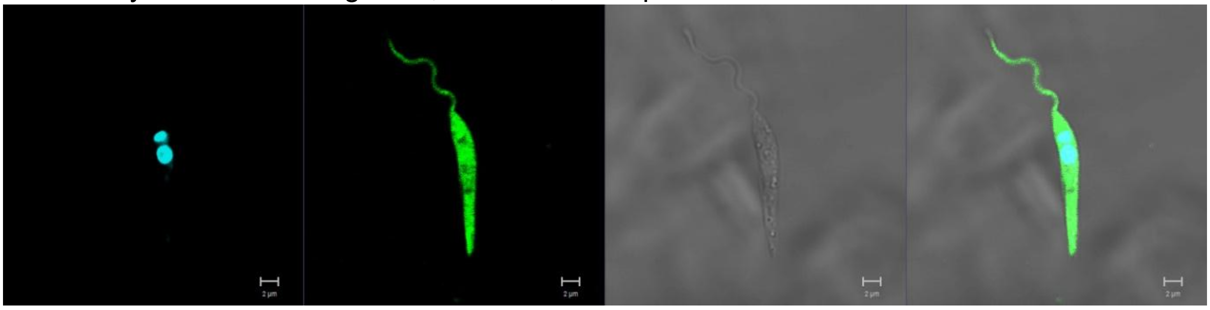

LmxM.29.2910 (MPK5)
N-terminus

Primary localisation: pellicular membrane

Secondary localisation: cytoplasm

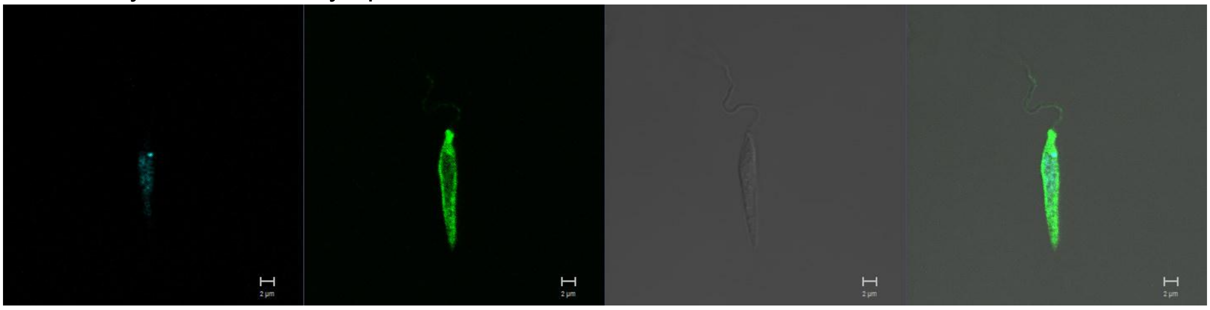

LmxM.32.1380 (MPK11)
N-terminus

Primary localisation: cytoplasm

Secondary localisation: nucleus, flagellum, basal body

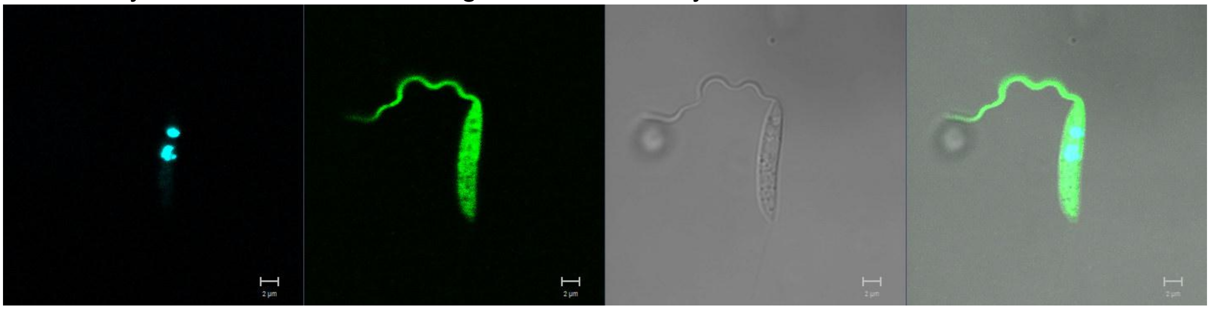

LmxM.36.0720 (MPK2)
N-terminus

Primary localisation: cytoplasm

Secondary localisation: flagellum, basal body

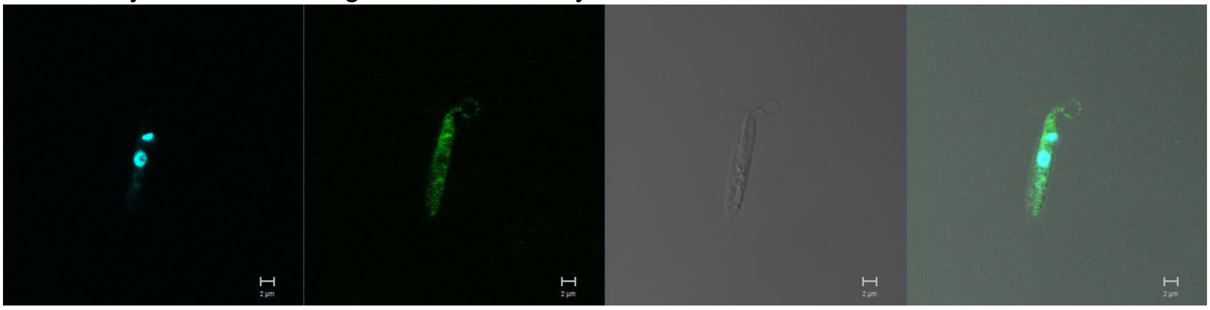

LmxM.20_36.6470 (MPK1)
N-terminus

Primary localisation: flagellum (anterior)

Secondary localisation: cytoplasm

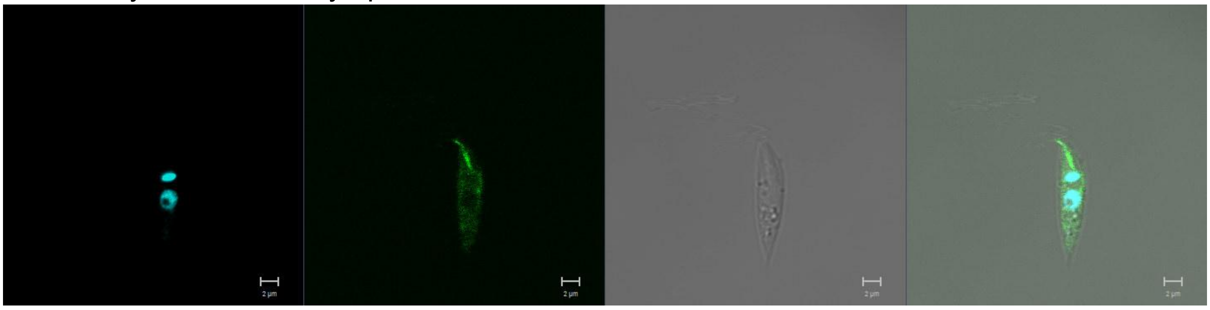

LmxM.28.0580 (MPK8)
N-terminus

Primary localisation: flagellum

Secondary localisation: cytoplasm

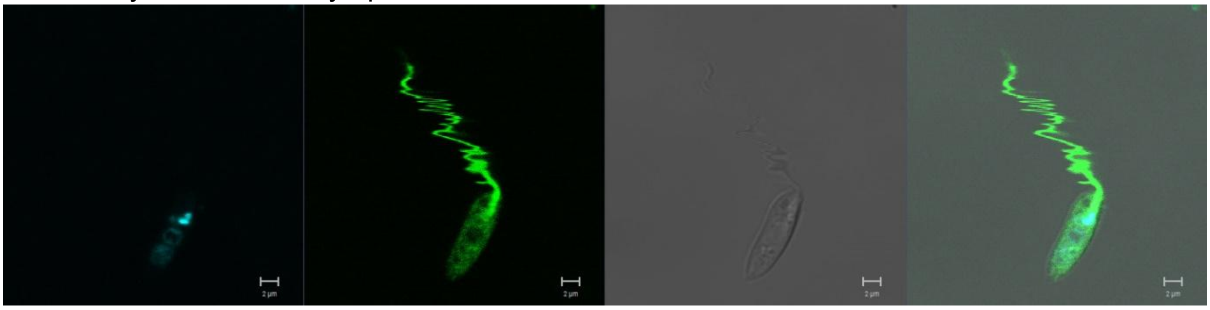

LmxM.31.3250 (MPK6)
N-terminus

Primary localisation in G1 phase: flagellum

Secondary localisation in G1 phase: cytoplasm

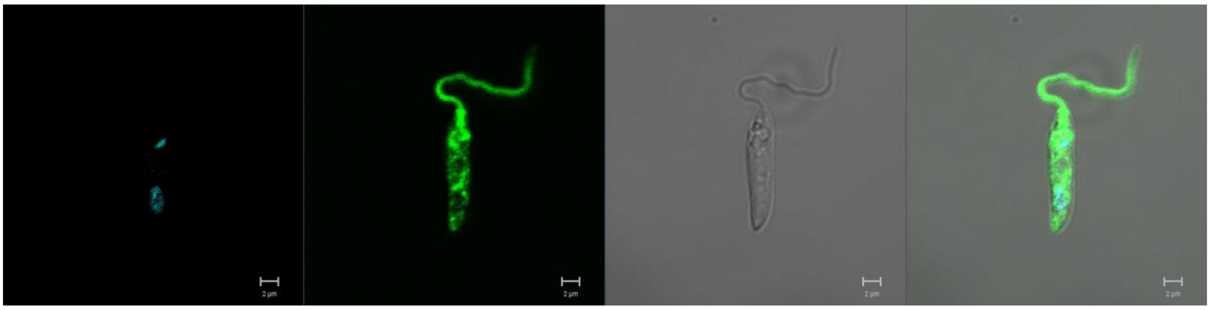

N-terminus

Primary localisation in S phase: pellicular membrane

Secondary localisation in S phase: flagellum, cytoplasm

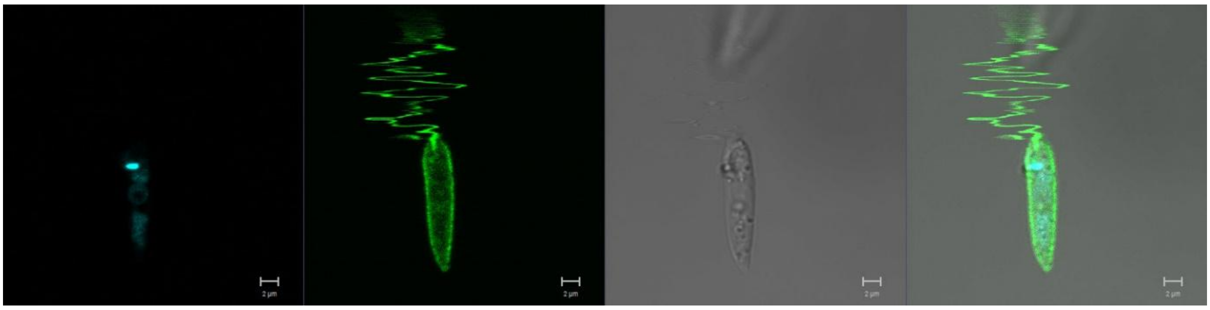

LmxM.19.0180 (MPK9)
N-terminus

Primary localisation: cytoplasm

Secondary localisation: lysosome

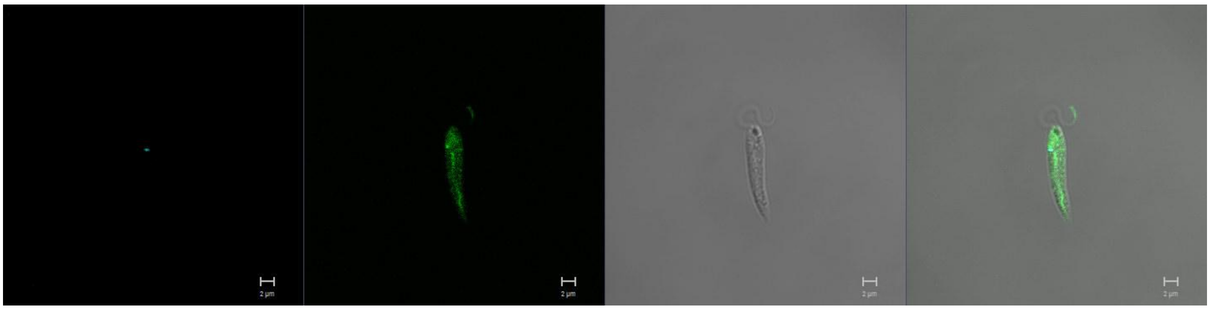

LmxM.27.0100 (MPK14)
N-terminus

Primary localisation: flagellum

Secondary localisation: basal body, cytoplasm

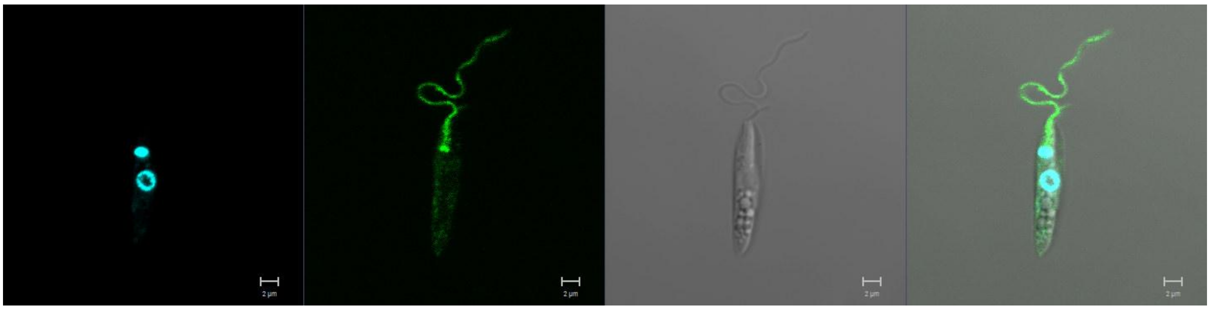

LmxM.34.5010 (MPK13)
N-terminus

Primary localisation: basal body

Secondary localisation: cytoplasm

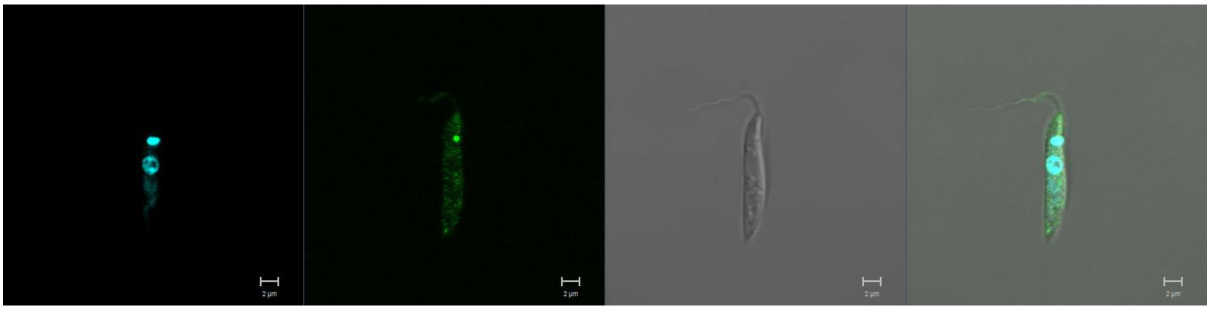

LmxM.26.0980
N-terminus

Primary localisation: cytoplasm

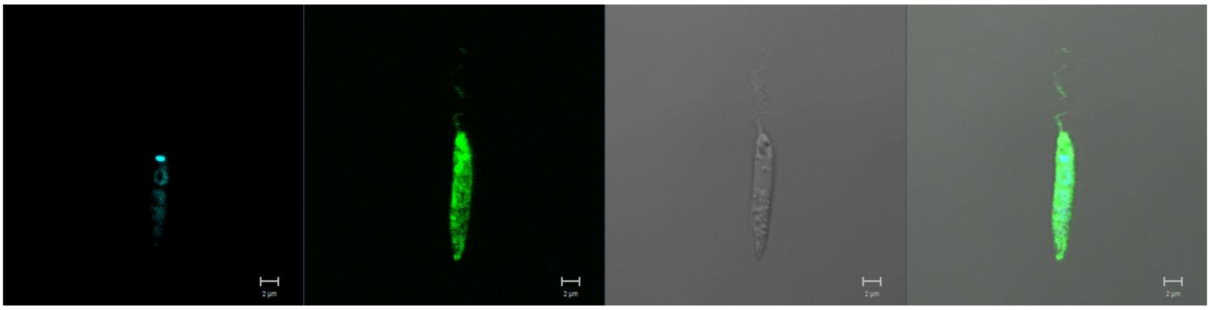

LmxM.29.3580
N-terminus

Primary localisation: cytoplasm

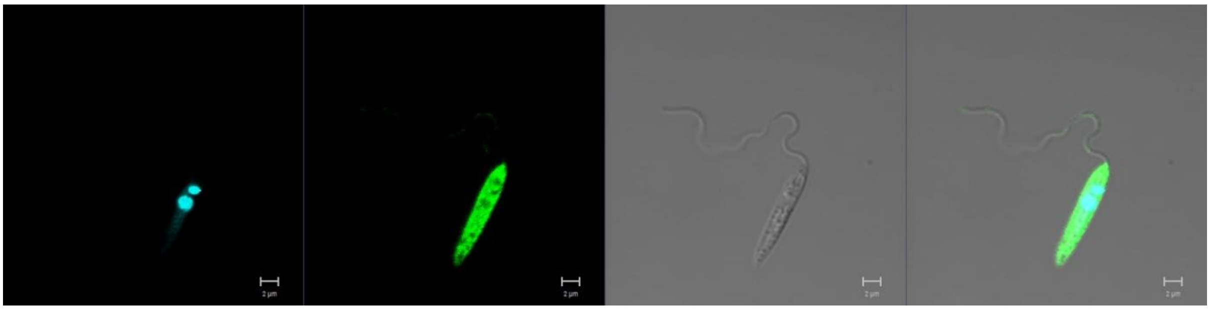

LmxM.17.0670
N-terminus

Primary localisation: basal body

Secondary localisation: cytoplasm

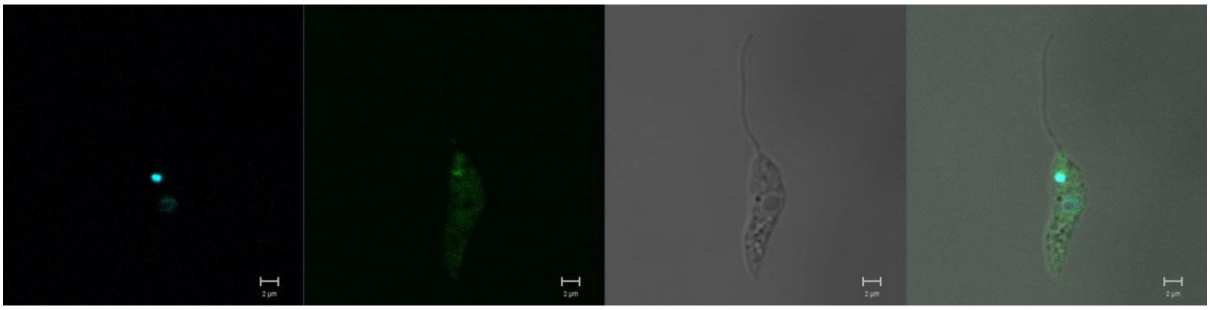

LmxM.01.0750
N-terminus

Primary localisation: cytoplasm

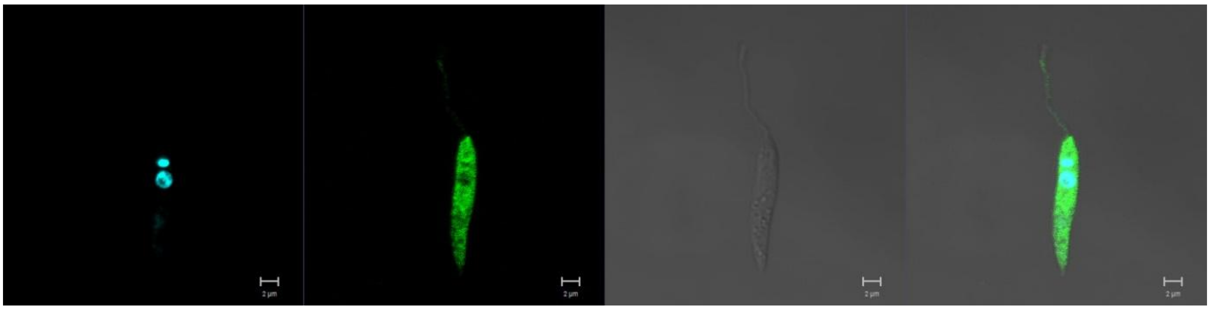

LmxM.12.0130 (LUK2)
N-terminus

Primary localisation: cytoplasm

Secondary localisation: flagellum, nucleus, kinetoplast

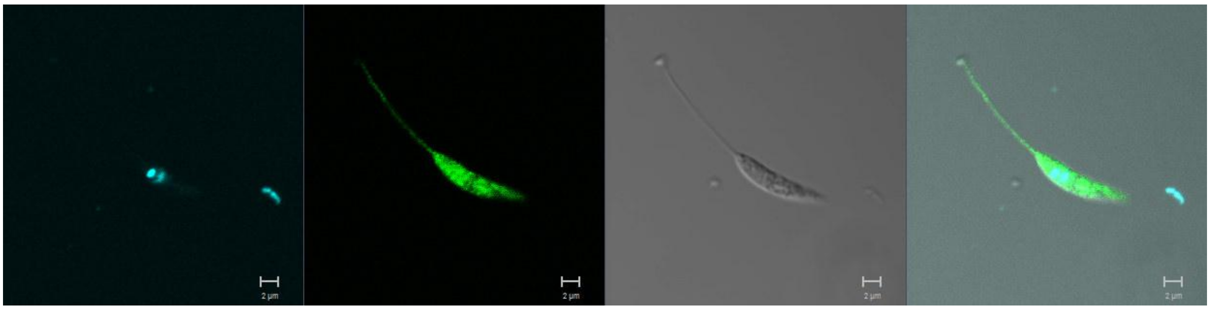

STE FAMILY

LmxM.24.2320 (MKK4)
N-terminus

Primary localisation: endomembrane

Secondary localisation: cytoplasm, flagellum

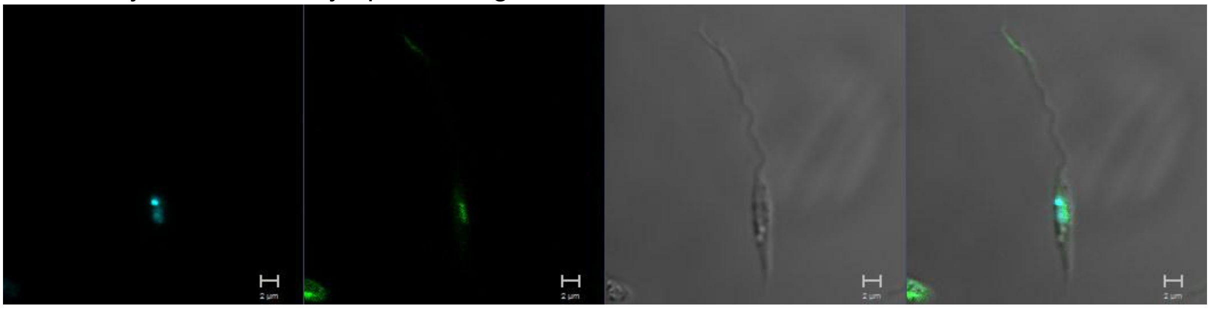

LmxM.08_29.2320 (MKK1)
C-terminus

Primary localisation: cytoplasm

Secondary localisation: flagellum

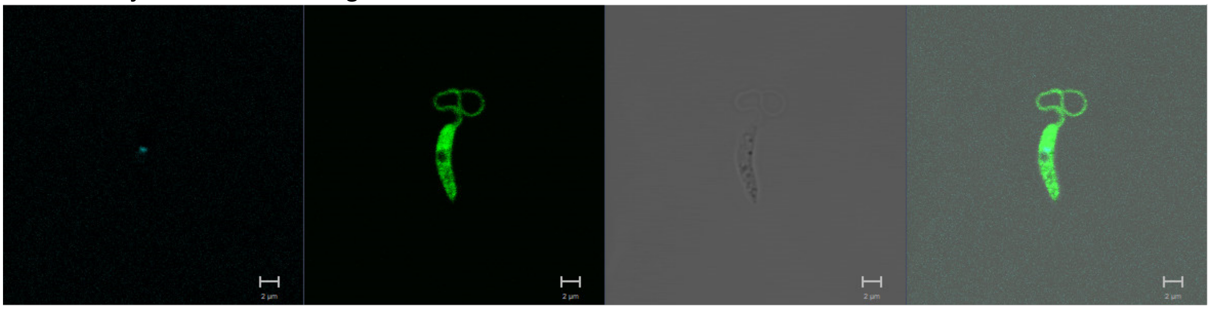

LmxM.08.1228

N-terminus

Primary localisation: cytoplasm

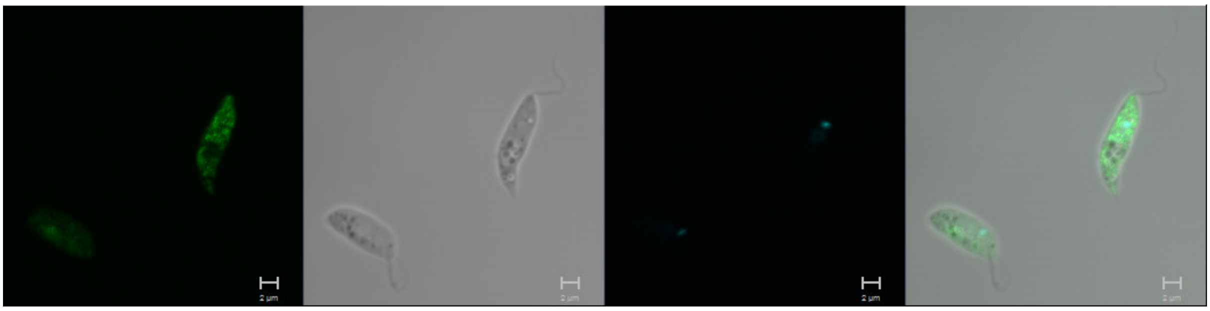

LmxM.15.1200
N-terminus

Primary localisation: cytoplasm

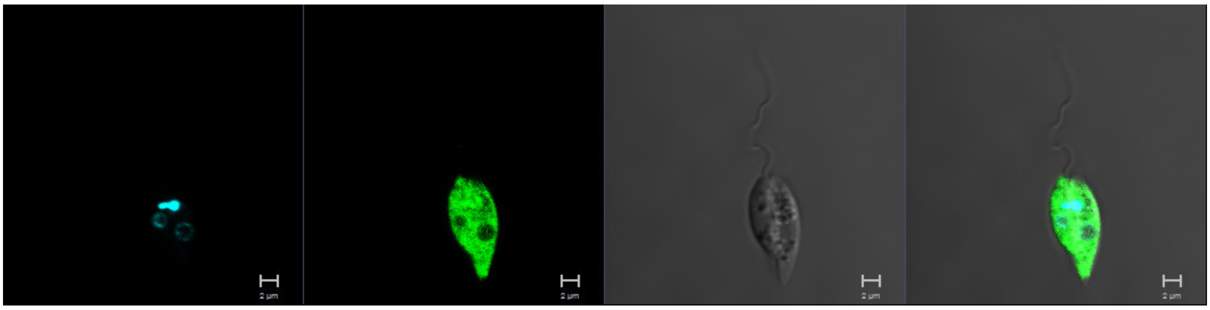

LmxM.17.0390
N-terminus

Primary localisation: basal body

Secondary localisation: cytoplasm

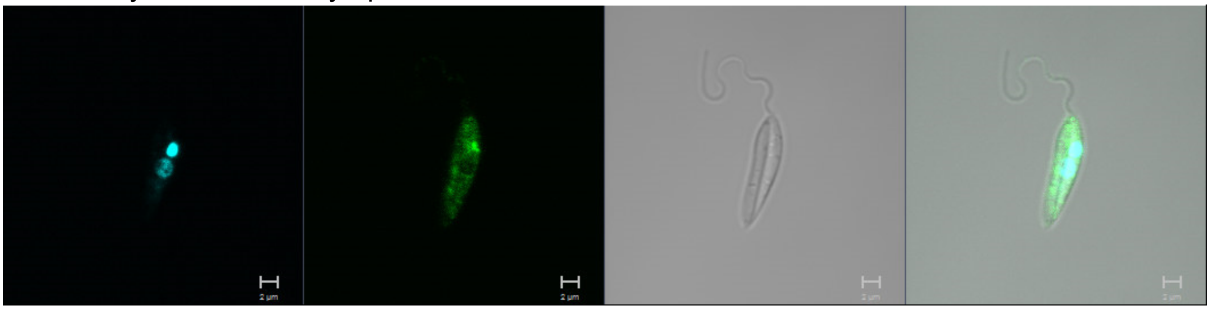

LmxM.20.0770
N-terminus

Primary localisation: pellicular membrane

Secondary localisation: cytoplasm

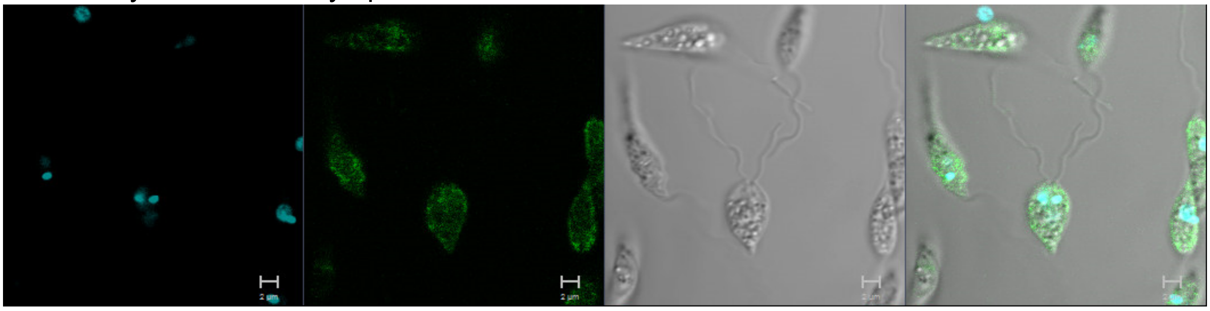

LmxM.21.0270
N-terminus

Primary localisation: cytoplasm

Secondary localisation: basal body, flagellum

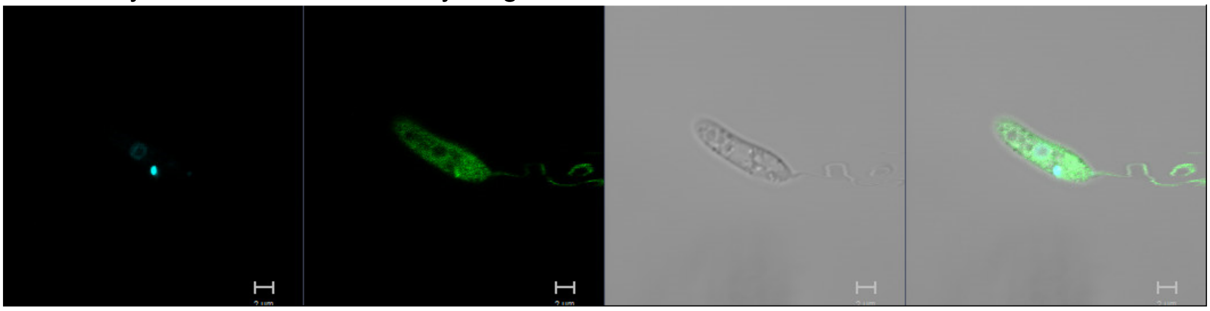

LmxM.25.1990
N-terminus

Primary localisation: endomembrane

Secondary localisation: lysosome, cytoplasm

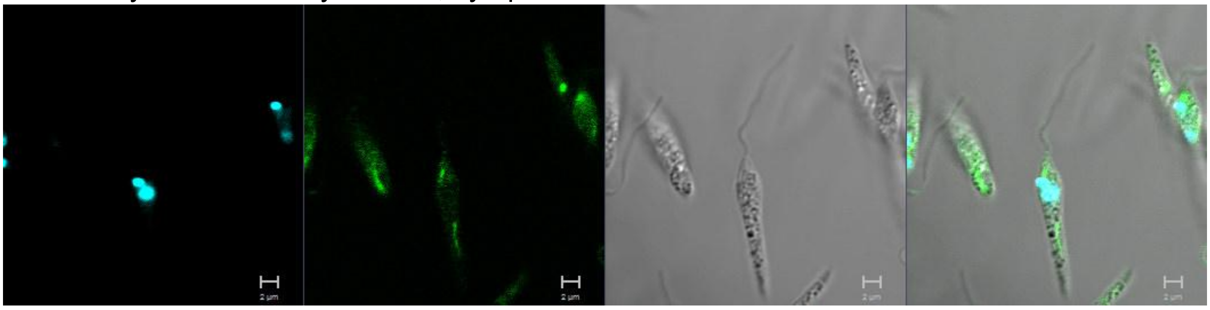

LmxM.31.1020
C-terminus

Primary localisation: cytoplasm

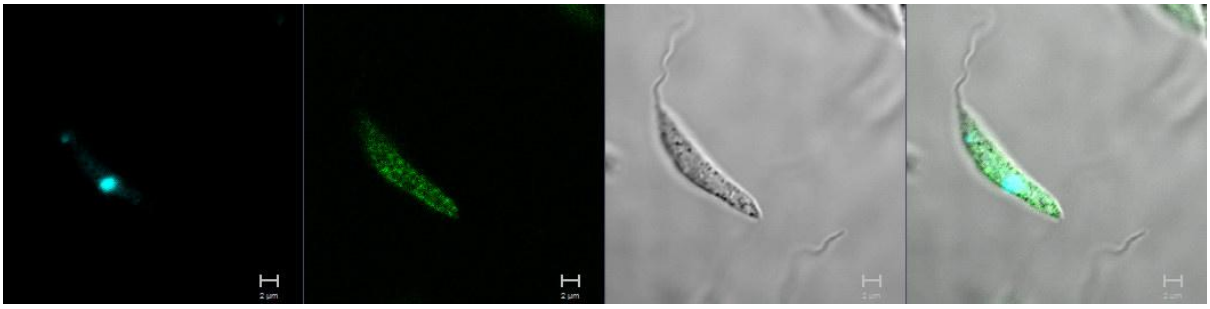

LmxM.34.3170
N-terminus

Primary localisation: cytoplasm

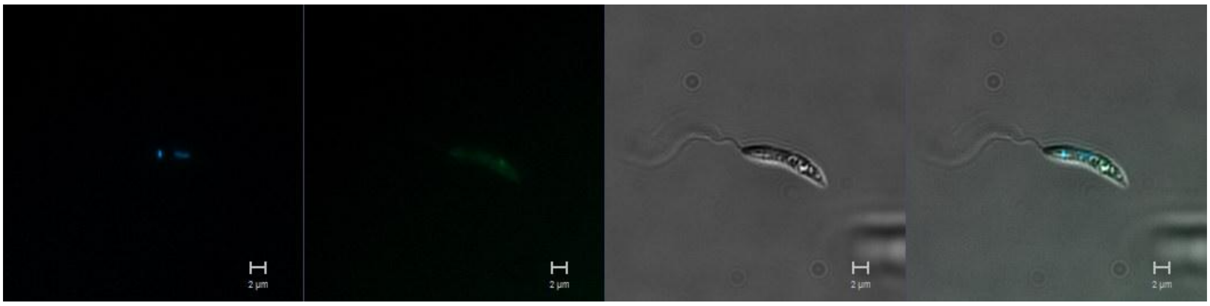

LmxM.04.0440
N-terminus

Primary localisation: flagellar pocket (collar region)

Secondary localisation: lysosomal, pellicular membrane, cytoplasm

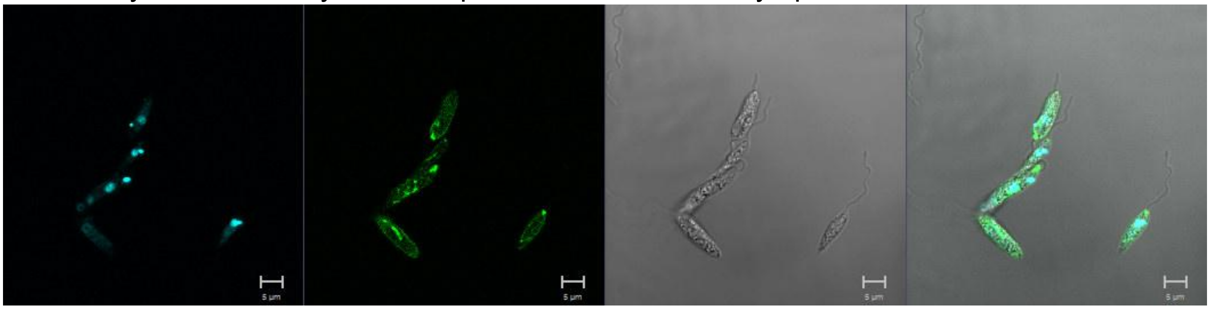

LmxM.05.0390
N-terminus

Primary localisation: basal body

Secondary localisation: flagellar pocket, cytoplasm, flagellum

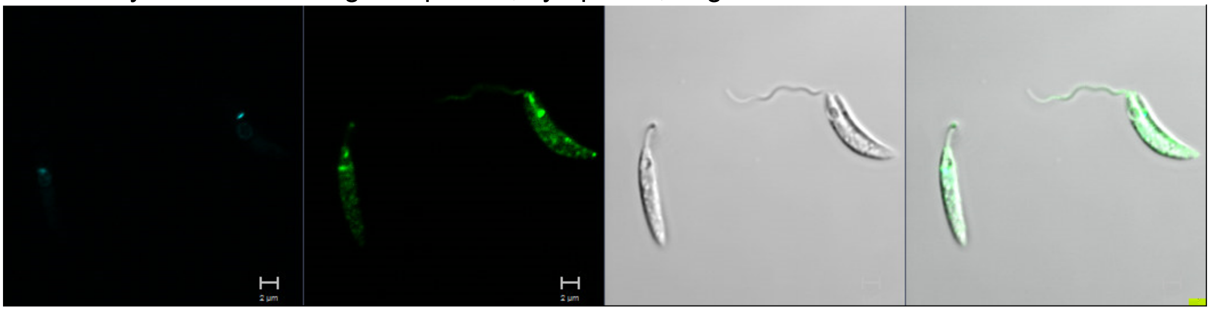

C-terminus

Primary localisation: basal body

Secondary localisation: flagellar pocket, cytoplasm

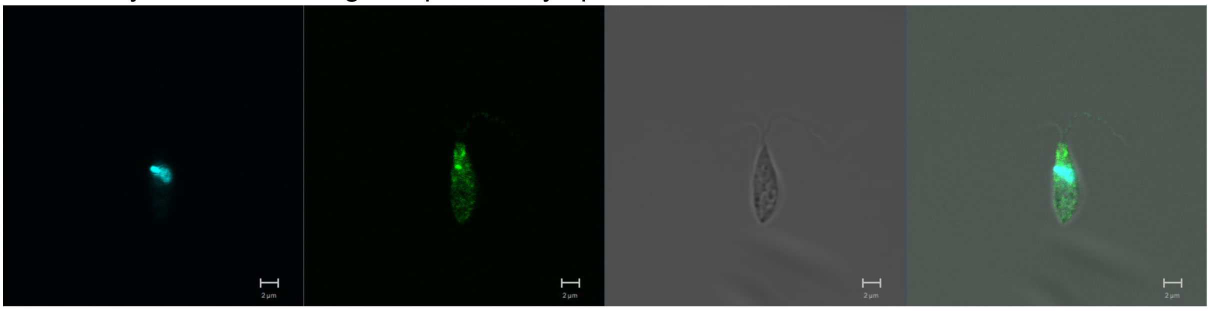

LmxM.06.0640
N-terminus

Primary localisation: lysosome

Secondary localisation: endomembrane

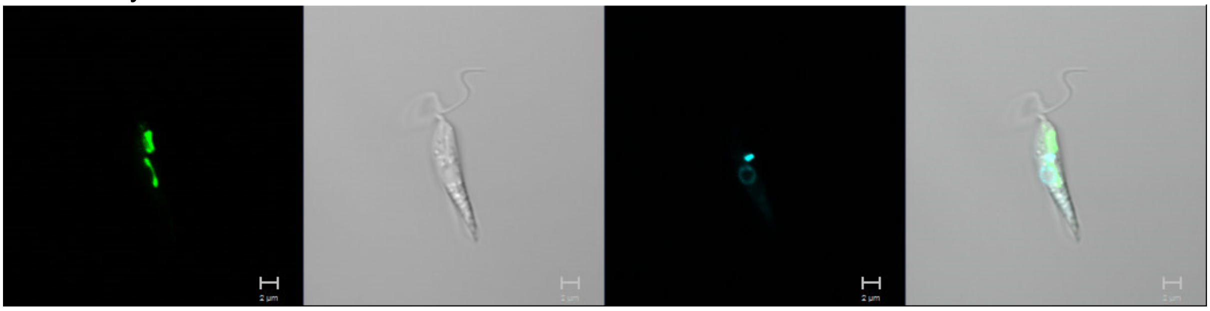

LmxM.07.0690
N-terminus

Primary localisation: cytoplasm

Secondary localisation: nucleus

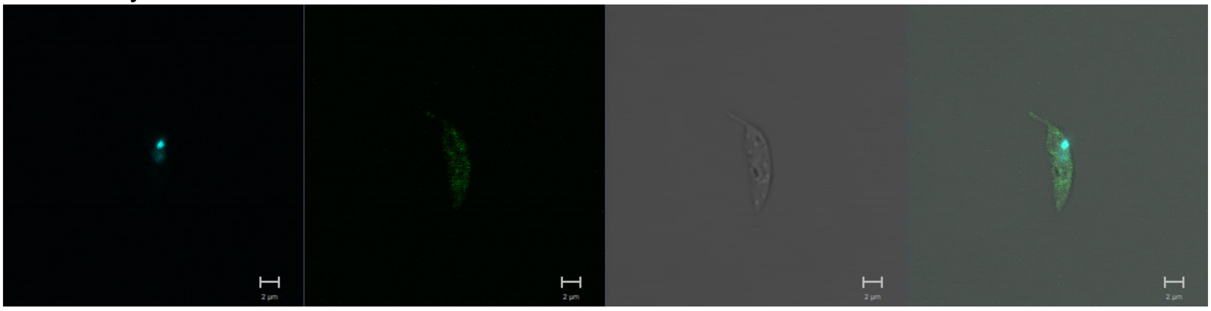

LmxM.07.0880
N-terminus

Primary localisation: cytoplasm

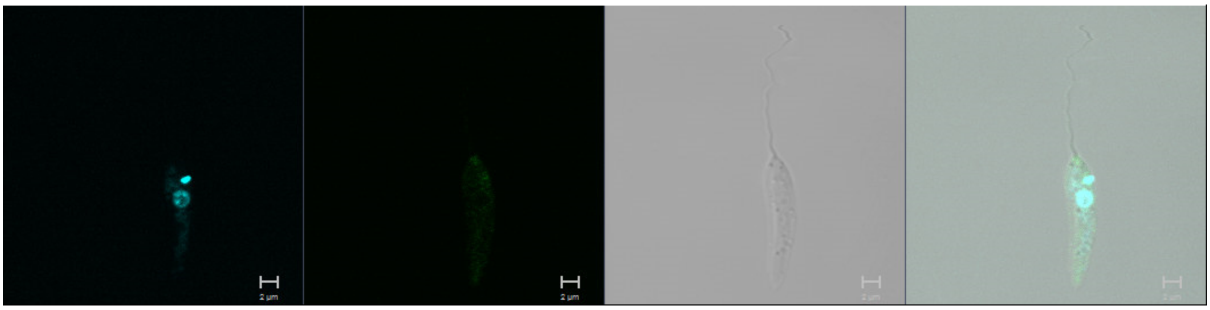

LmxM.14.1300
N-terminus

Primary localisation: lysosome

Secondary localisation: endomembrane

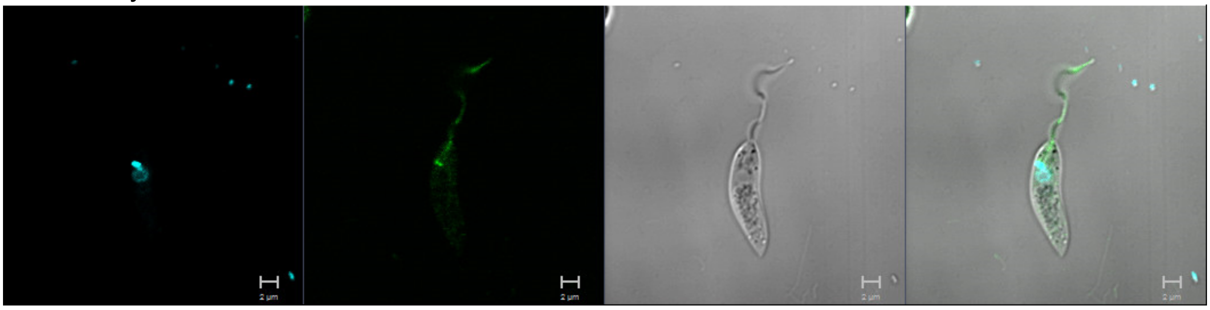

LmxM.17.0490
N-terminus

Primary localisation: flagellum

Secondary localisation: cytoplasm

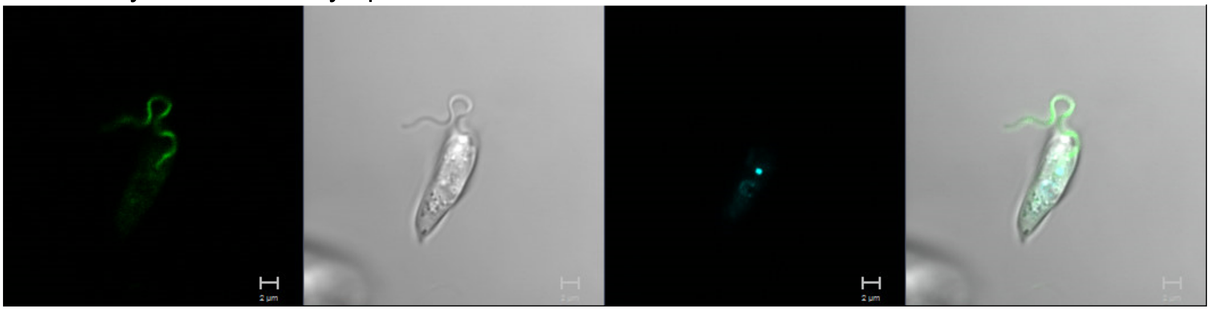

LmxM.19.0150 (CBPK1)
N-terminus

Primary localisation: cytoplasm

Secondary localisation: flagellum

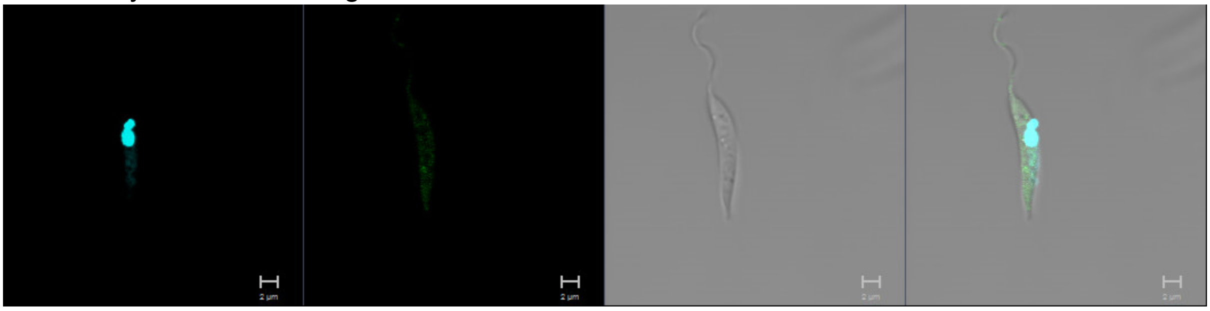

LmxM.21.0130
N-terminus

Primary localisation: endomembrane

Secondary localisation: golgi apparatus, cytoplasm

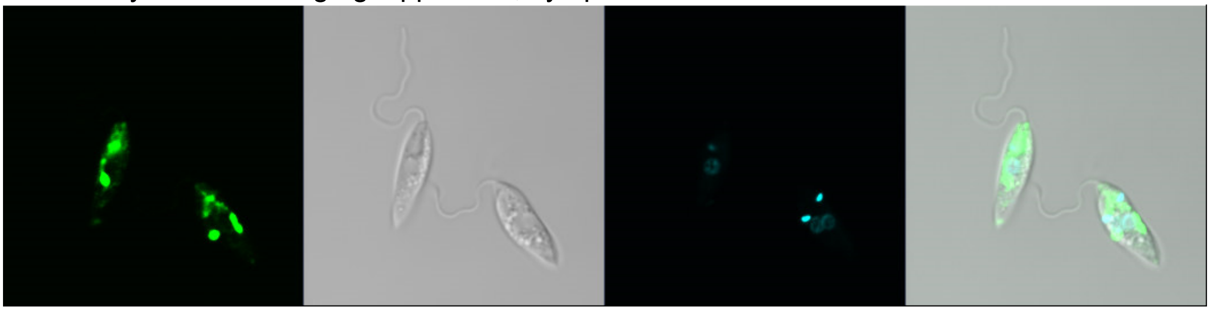

LmxM.24.1450
N-terminus

Primary localisation: cytoplasm

Secondary localisation: flagellum, nucleus, kinetoplast

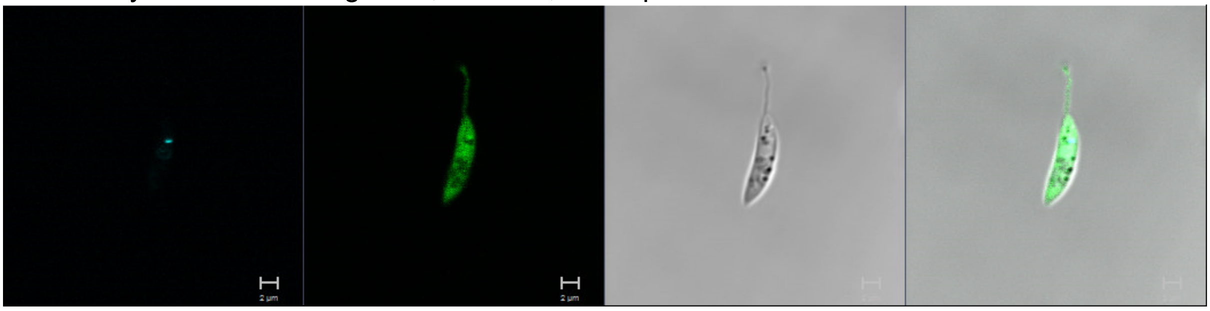

LmxM.26.1730
N-terminus

Primary localisation: lysosome

Secondary localisation: flagellum

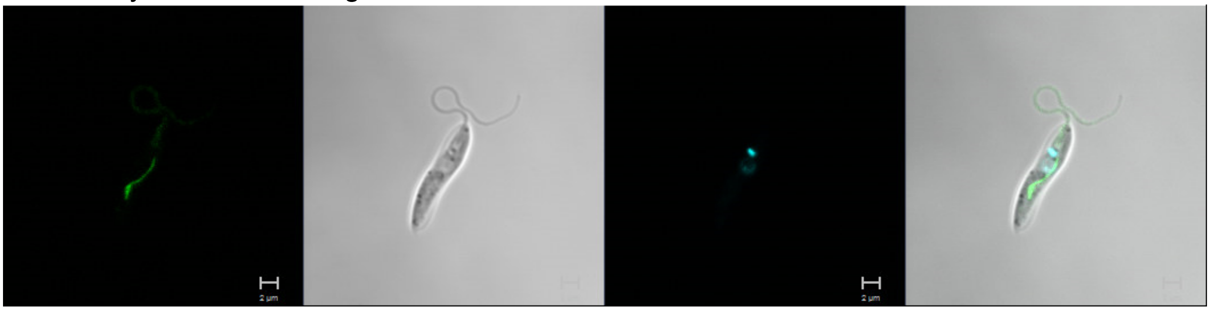

LmxM.27.1370
N-terminus

Primary localisation: endomembrane

Secondary localisation: cytoplasm

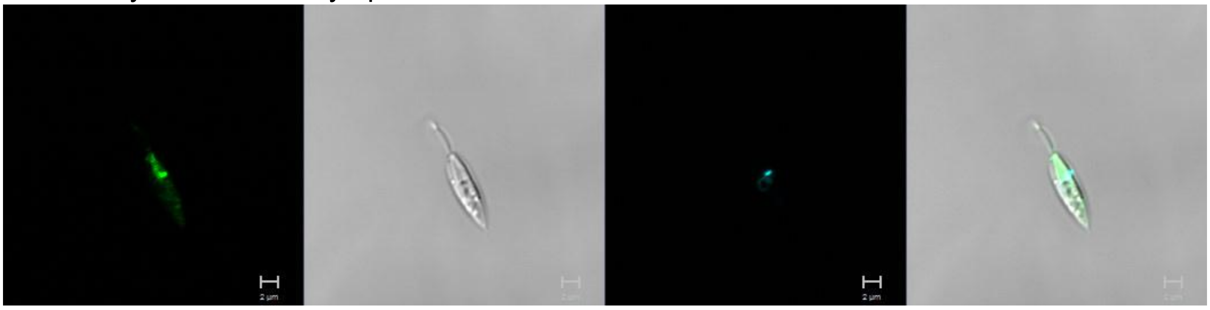

LmxM.29.0600
N-terminus

Primary localisation: flagellum

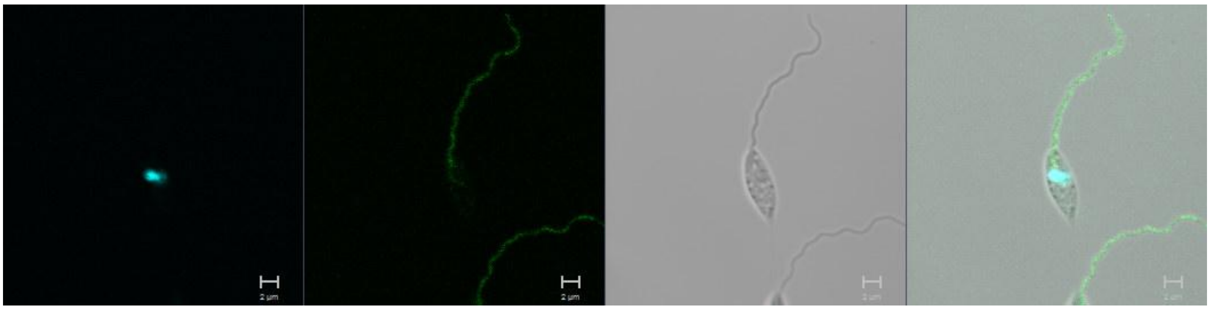

LmxM.29.3050
N-terminus

Primary localisation: cytoplasm (low expression)

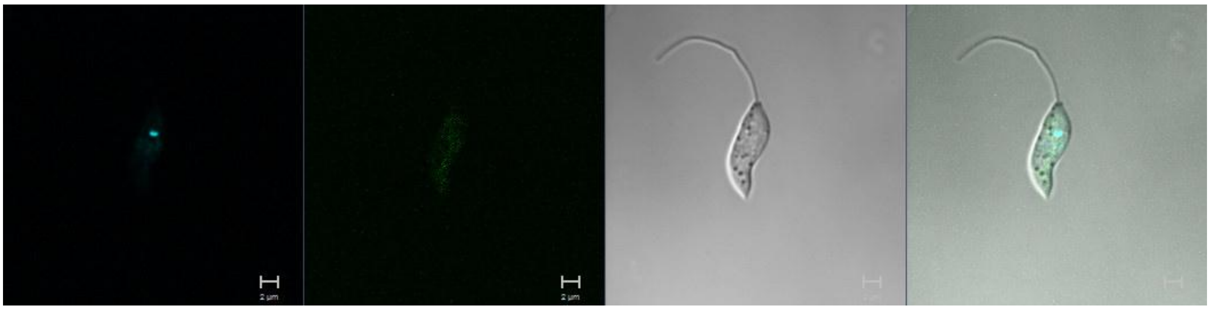

LmxM.31.0120 (MRK1)
N-terminus

Primary localisation: cytoplasm

Secondary localisation: kinetoplast

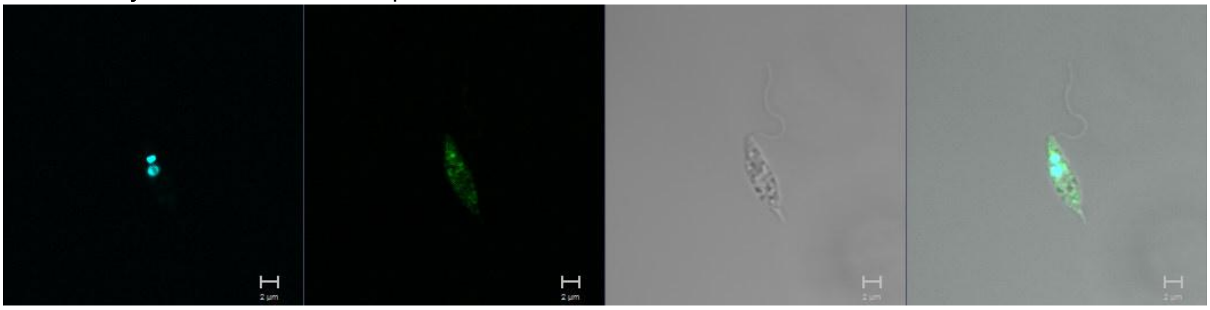

LmxM.31.0780
N-terminus

Primary localisation: basal body

Secondary localisation: cytoplasm

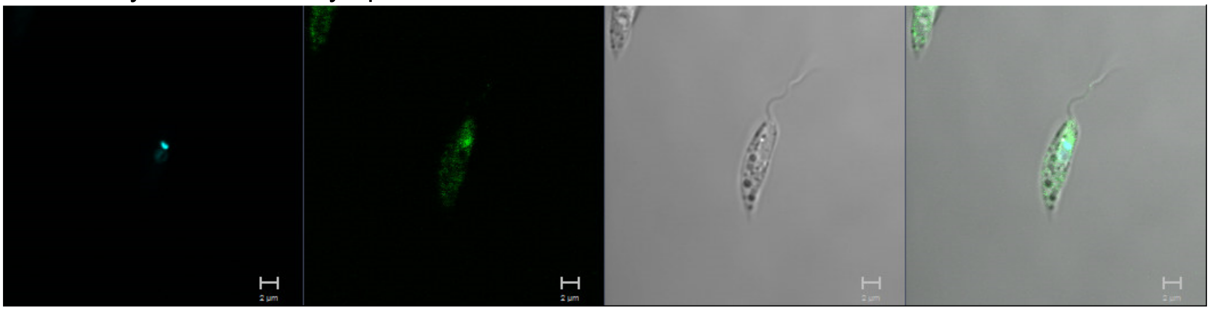

LmxM.31.0810 (RDK1)
N-terminus

Primary localisation: cytoplasm

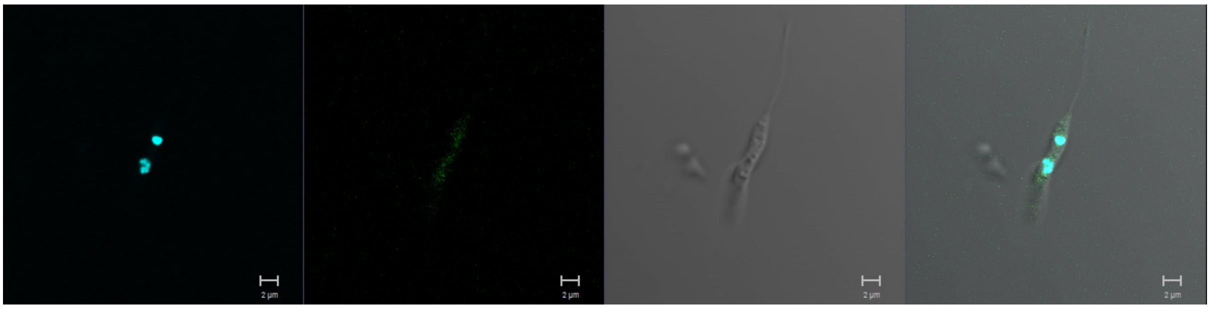

LmxM.32.1400
C-terminus

Primary localisation: nucleus

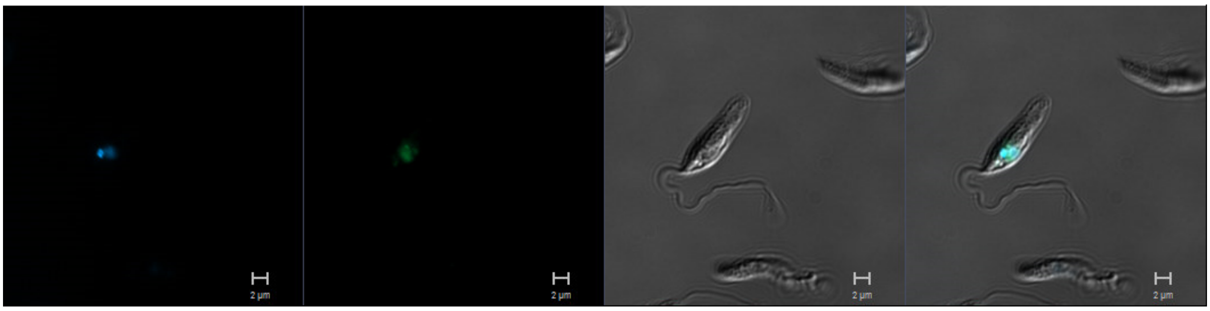

LmxM.32.2290
N-terminus

Primary localisation: lysosome

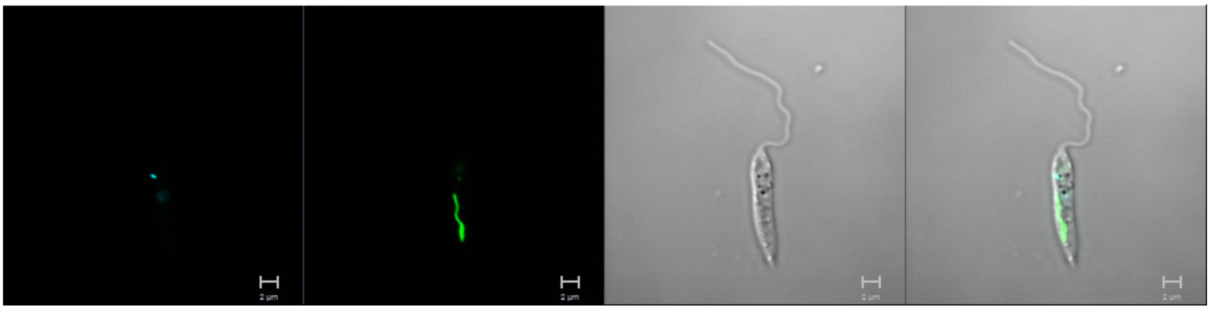

LmxM.36.0910
N-terminus

Primary localisation in log-phase culture: endomembrane

Secondary localisation: flagellum

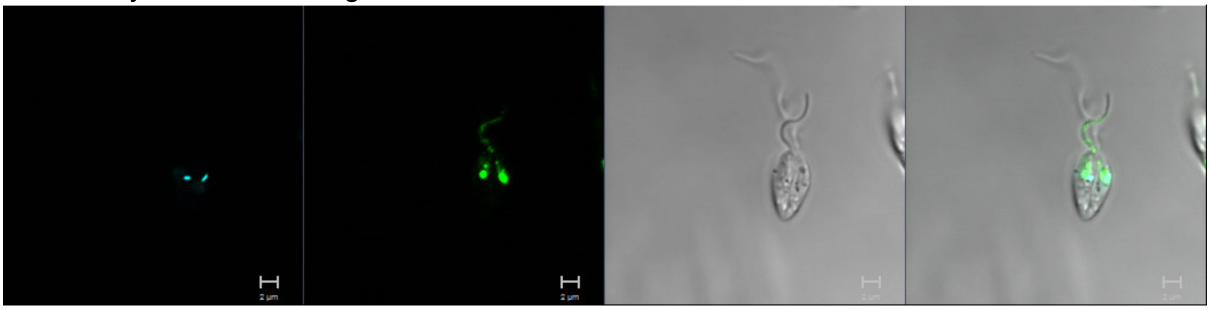

Primary localisation in stationary phase culture: lysosome

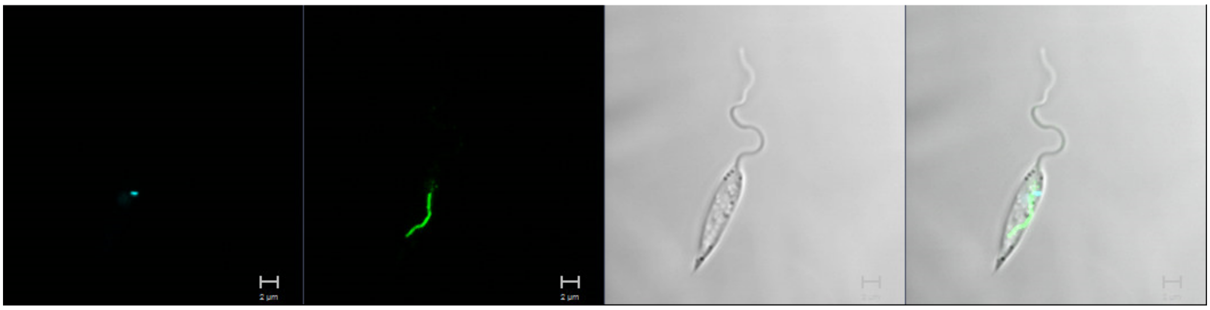

LmxM.16.0300
N-terminus

Primary localisation: basal body

Secondary localisation: cytoplasm

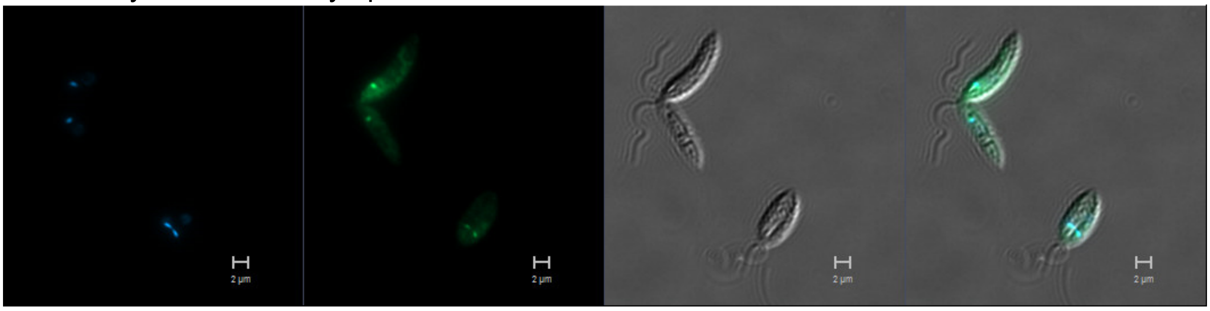

C-terminus

Primary localisation: basal body

Secondary localisation: cytoplasm

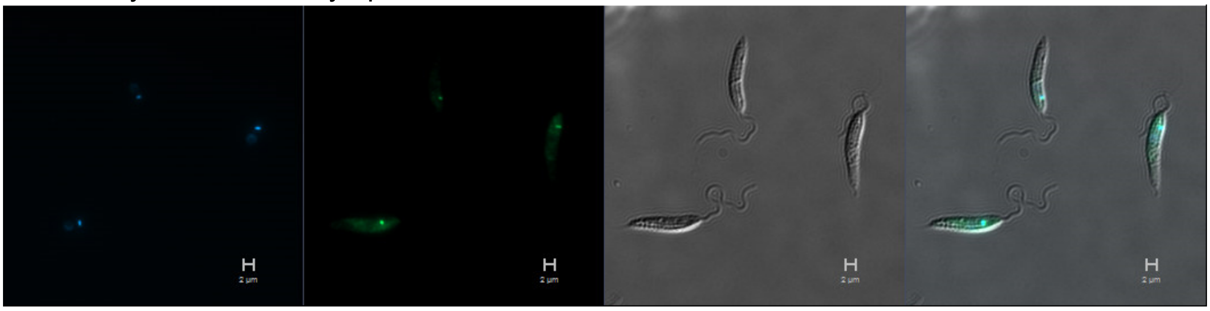

LmxM.07.0250
C-terminus

Primary localisation: cytoplasm

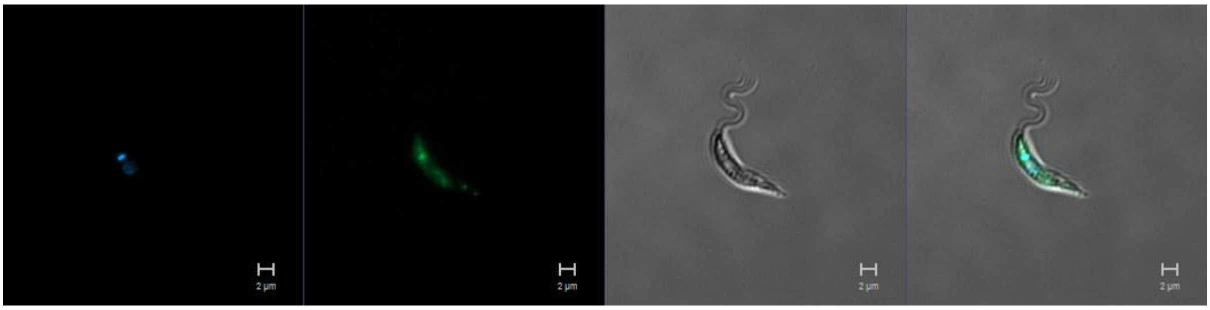

LmxM.36.0860 (MKK5)
C-terminus

Primary localisation: cytoplasm

Secondary localisation: flagellum

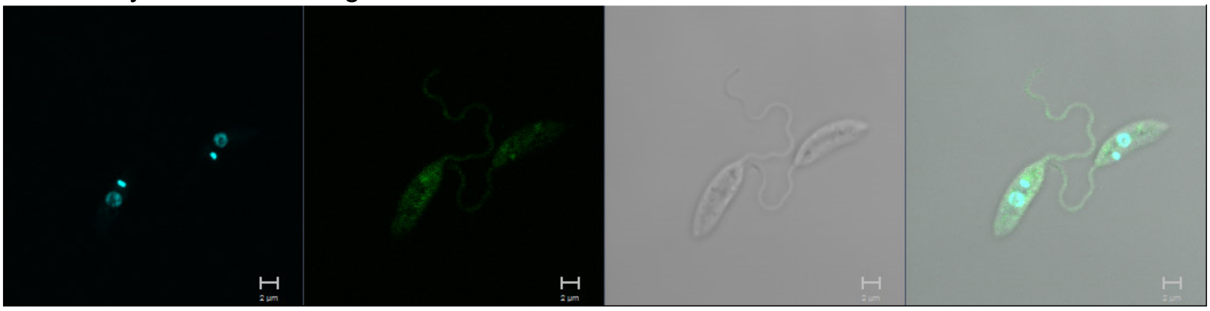

LmxM.19.1610
C-terminus

Primary localisation: cytoplasm

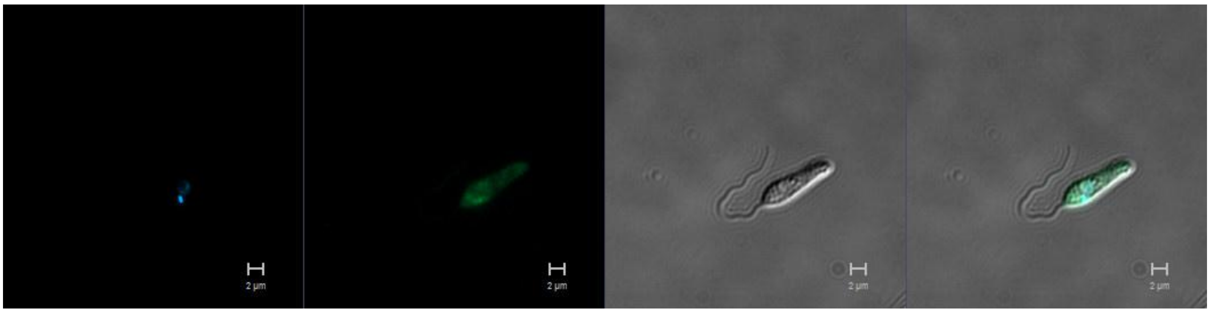

LmxM.33.2090
N-terminus

Primary localisation: cytoplasm

Secondary localisation: flagellum

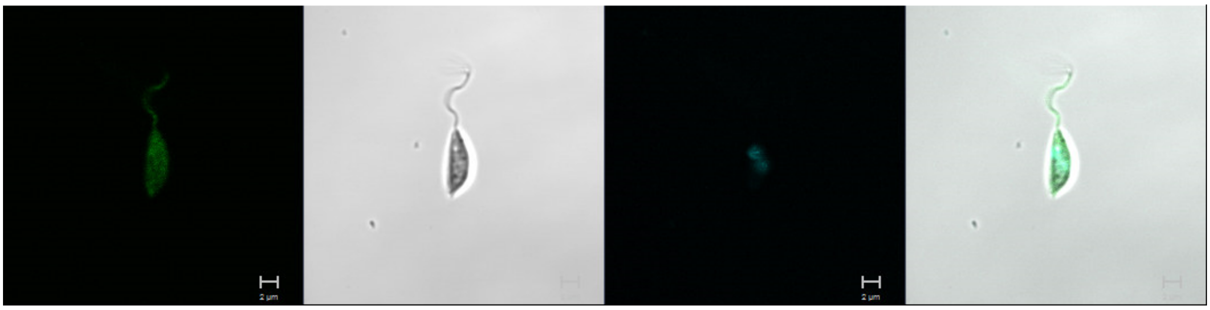

LmxM.34.4000
N-terminus

Primary localisation: cytoplasm

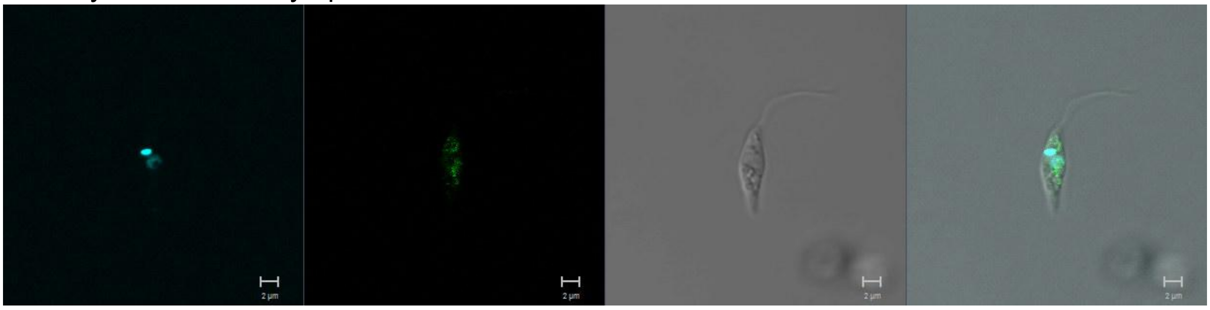

LmxM.36.3680
N-terminus

Primary localisation: endomembrane

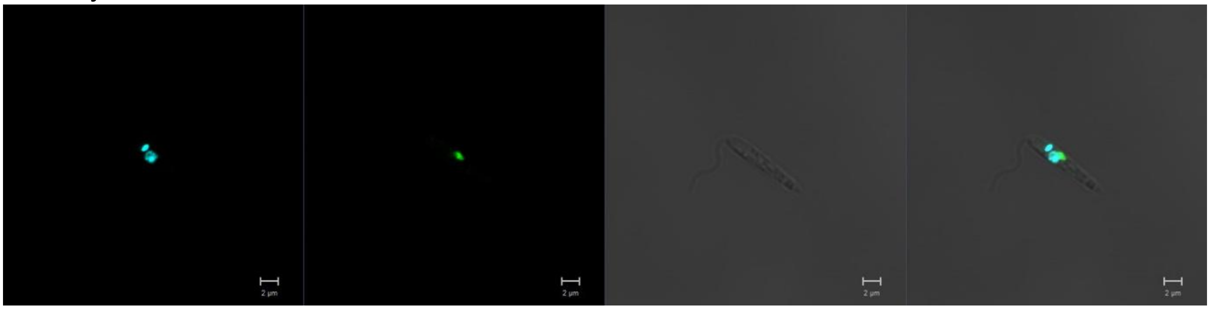

LmxM.30.1830
N-terminus

Primary localisation: endomembrane

Secondary localisation: cytoplasm

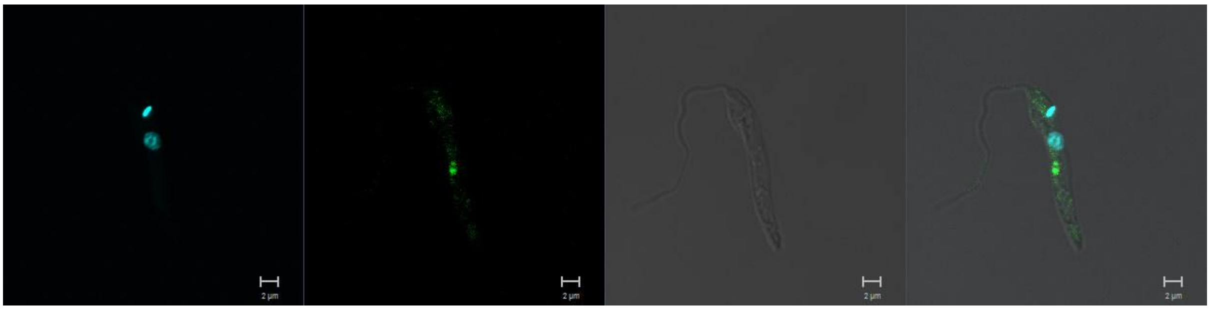

LmxM.30.1840
N-terminus

Primary localisation: cytoplasm

Secondary localisation: nucleus, flagellum, basal body

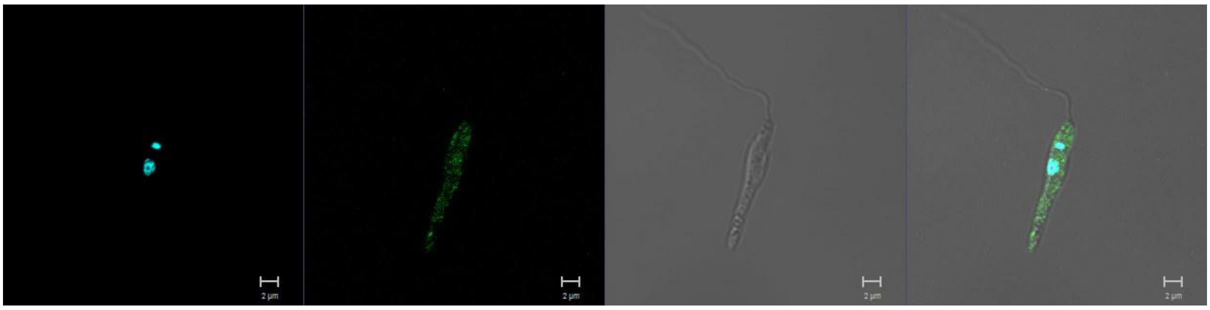

NEK FAMILY

LmxM.02.0290
N-terminus

Primary localisation: cytoplasm

Secondary localisation: nucleus, flagellum

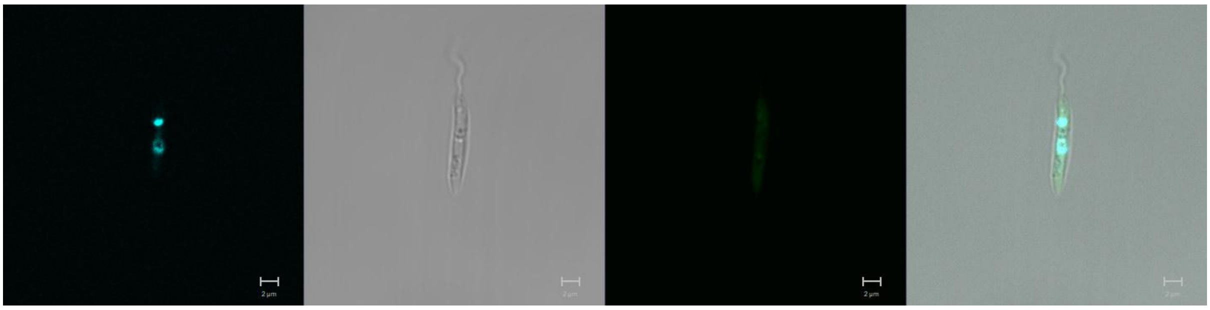

Primary localisation in dividing cells: basal body

Secondary localisation in dividing cells: cytoplasm, flagellum

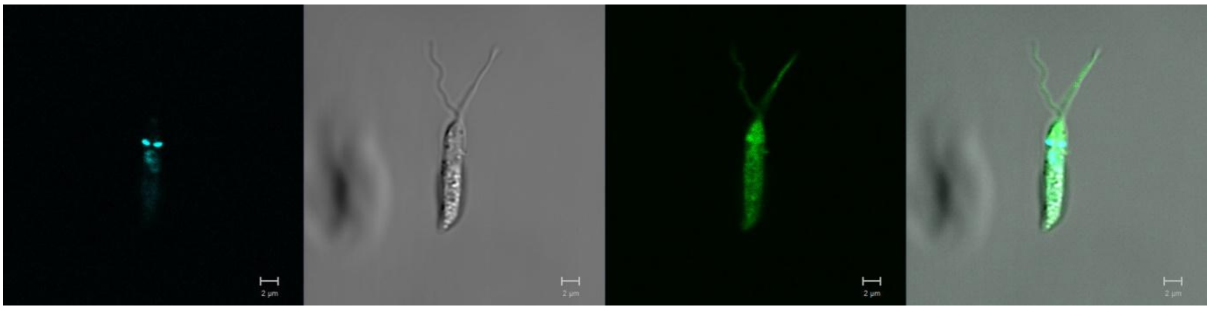

LmxM.07.0160
N-terminus

Primary localisation: endomembrane

Secondary localisation: lysosome (region)

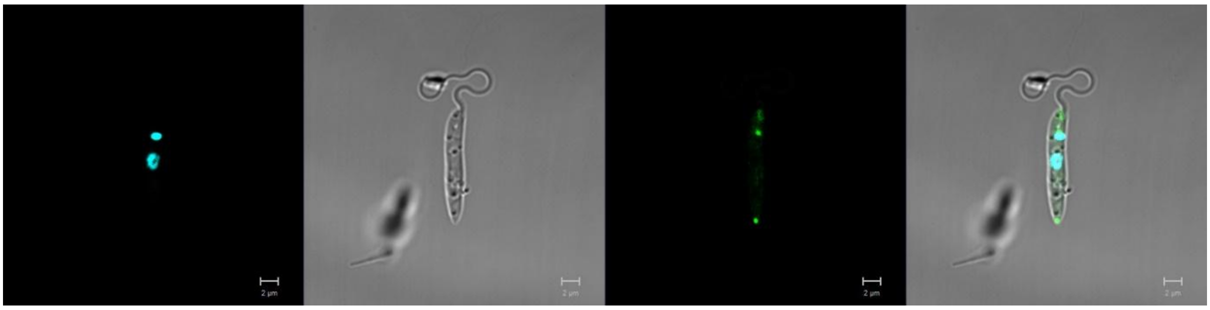

LmxM.07.0170
N-terminus

Primary localisation: basal body

Secondary localisation: cytoplasm, flagellum

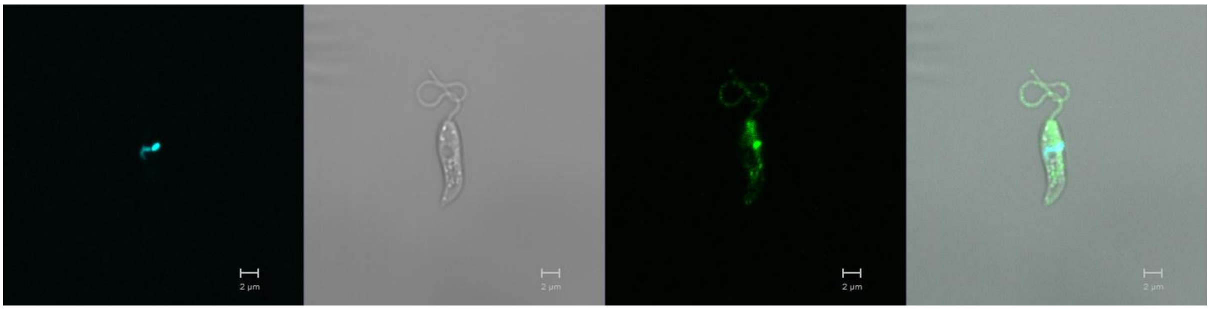

LmxM.08.0930
N-terminus

Primary localisation: flagellum

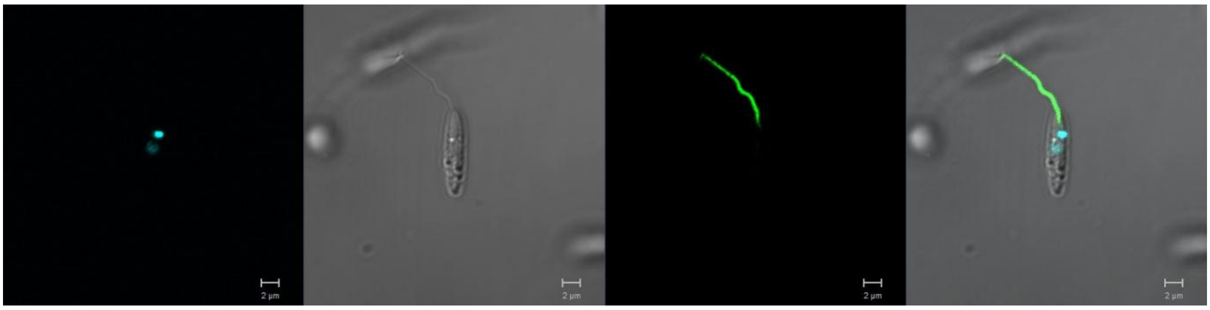

LmxM.14.1410
N-terminus

Primary localisation: flagellum

Secondary localisation: cytoplasm

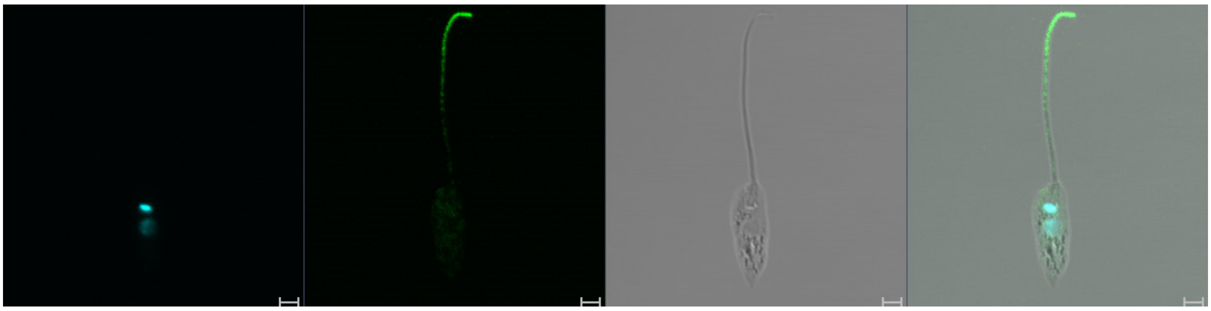

LmxM.21.0853
N-terminus

Primary localisation: cytoplasm

Secondary localisation: flagellum

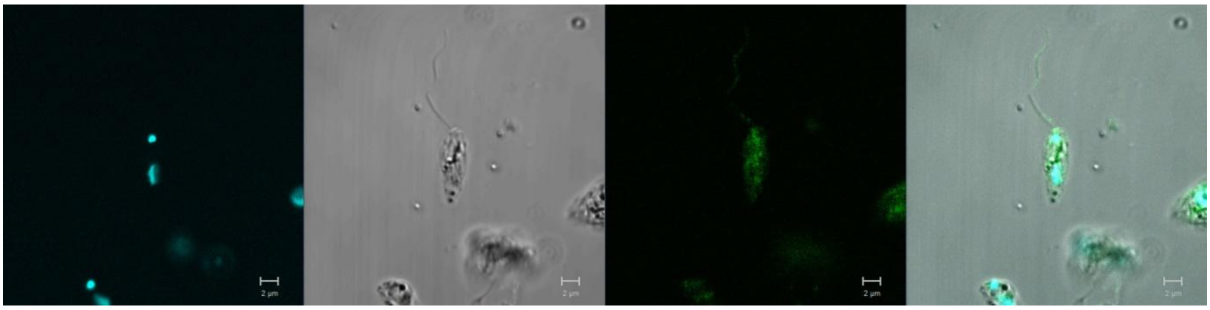

LmxM.21.1565
N-terminus

Primary localisation: basal body

Secondary localisation: cytoplasm, flagellum

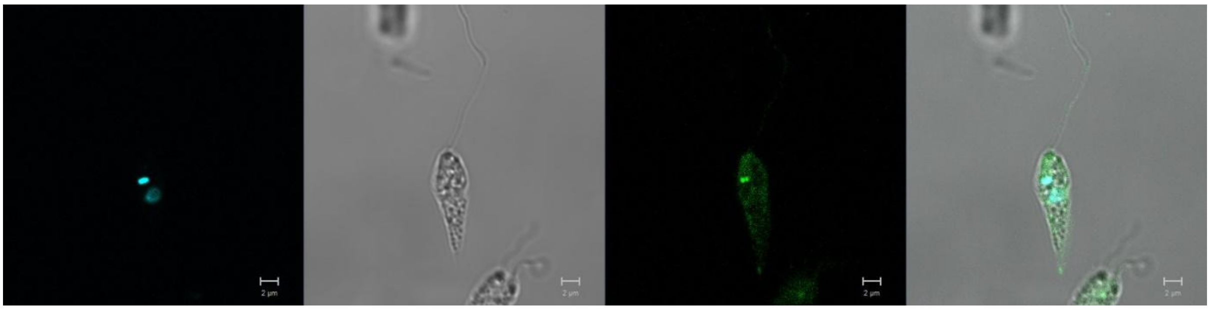

LmxM.22.0950
N-terminus

Primary localisation: cytoplasm

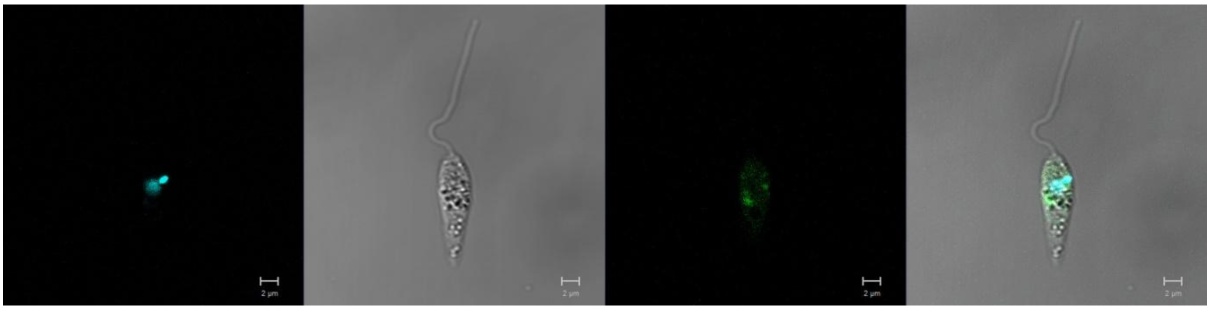

LmxM.26.2570
N-terminus

Primary localisation: basal body

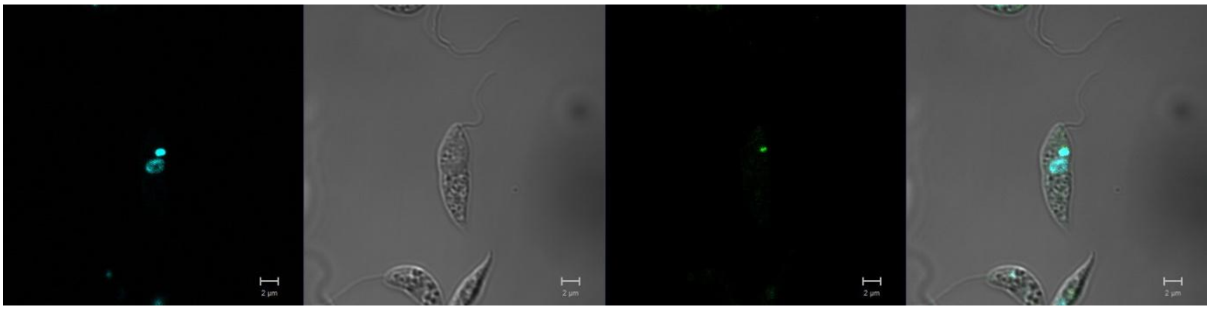

LmxM.28.3000
N-terminus

Primary localisation: cytoplasm

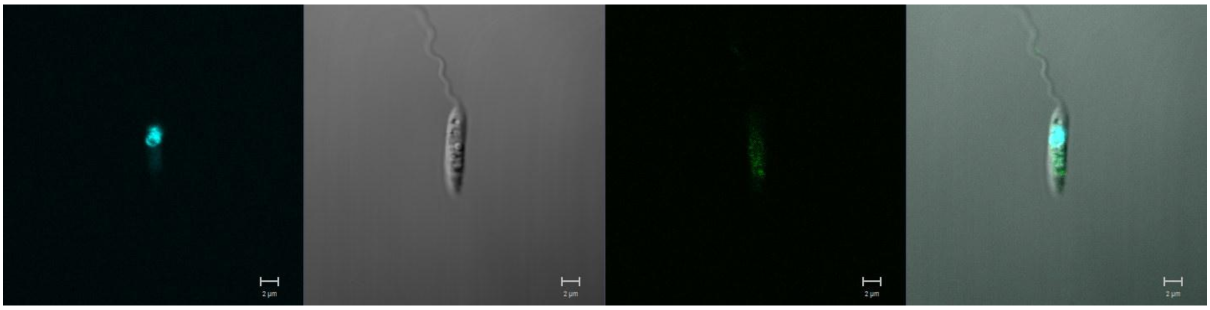

LmxM.08_29.2570
N-terminus

Primary localisation: cytoplasm

Secondary localisation: nucleus, flagellum

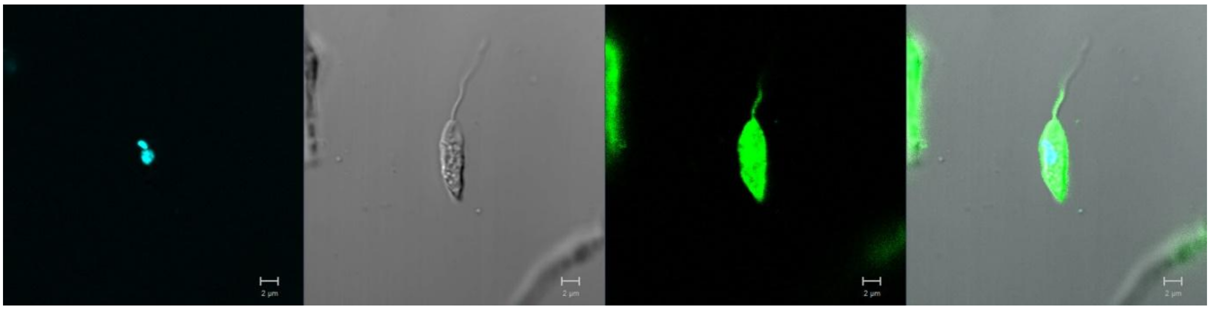

LmxM.08_29.2670
N-terminus

Primary localisation: cytoplasm

Secondary localisation: flagellum

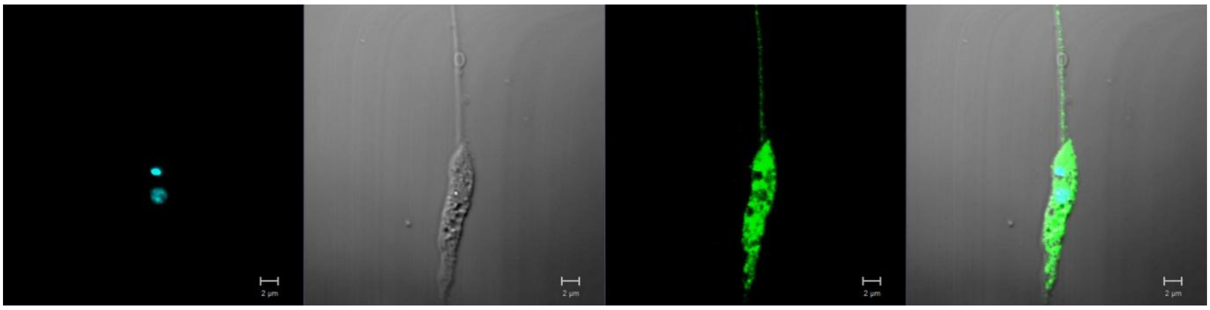

LmxM.29.2130
N-terminus

Primary localisation: basal body

Secondary localisation: cytoplasm, flagellum

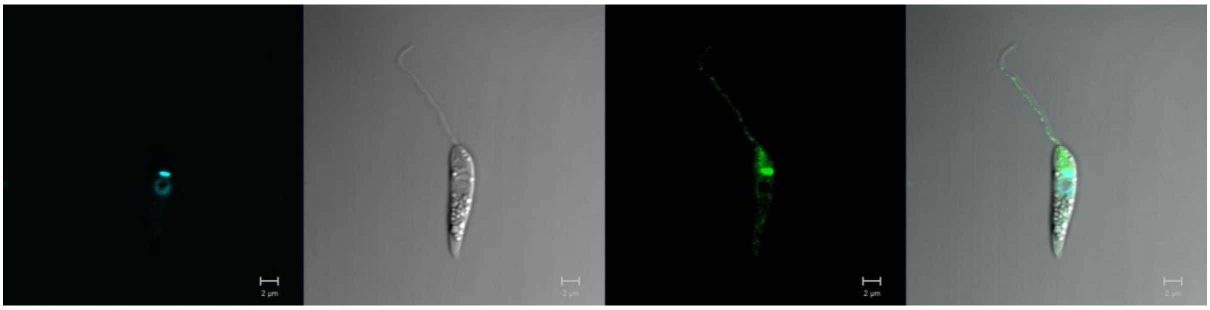

LmxM.30.2960 (RDK2)
N-terminus

Primary localisation: cytoplasm

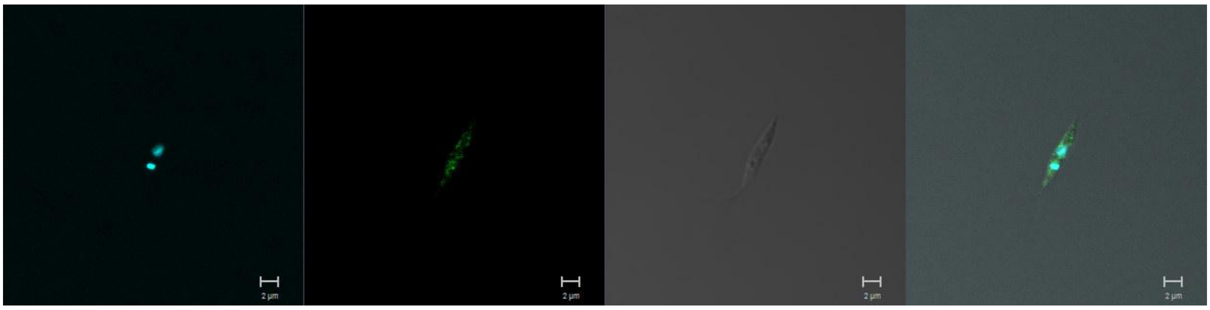

LmxM.30.3160
N-terminus

Primary localisation: cytoplasm

Secondary localisation: nucleus, flagellum

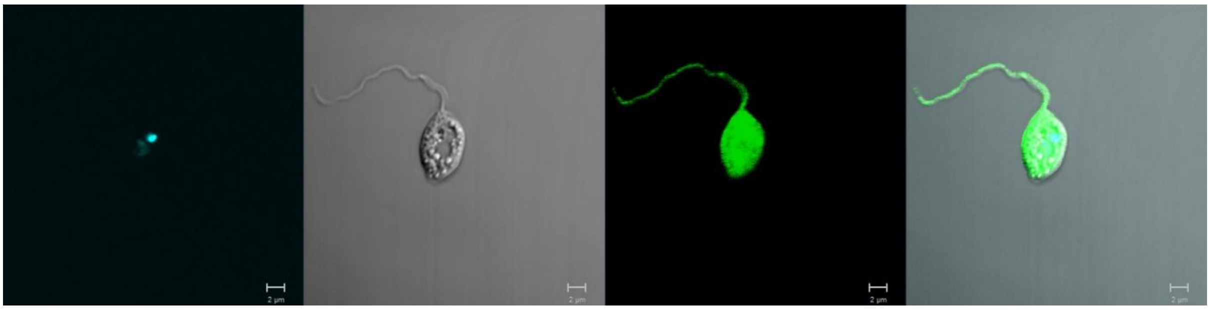

C-terminus

Primary localisation: cytoplasm

Secondary localisation: flagellum, basal body

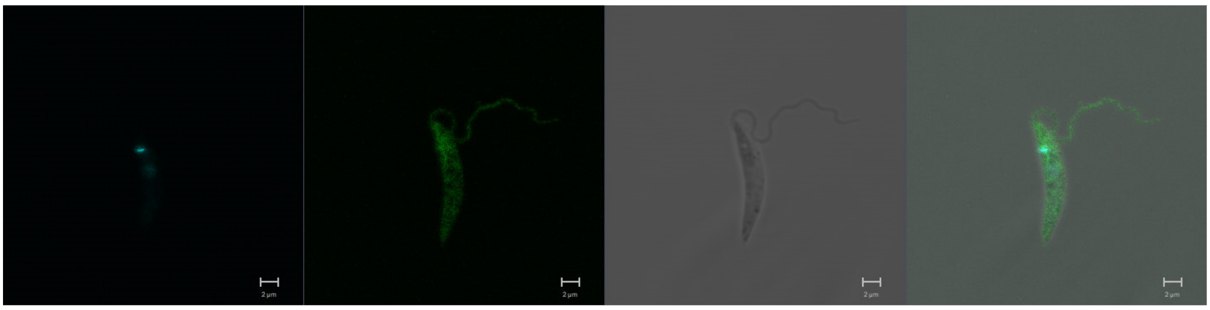

LmxM.31.0260
N-terminus

Primary localisation: flagellum

Secondary localisation: basal body, cytoplasm

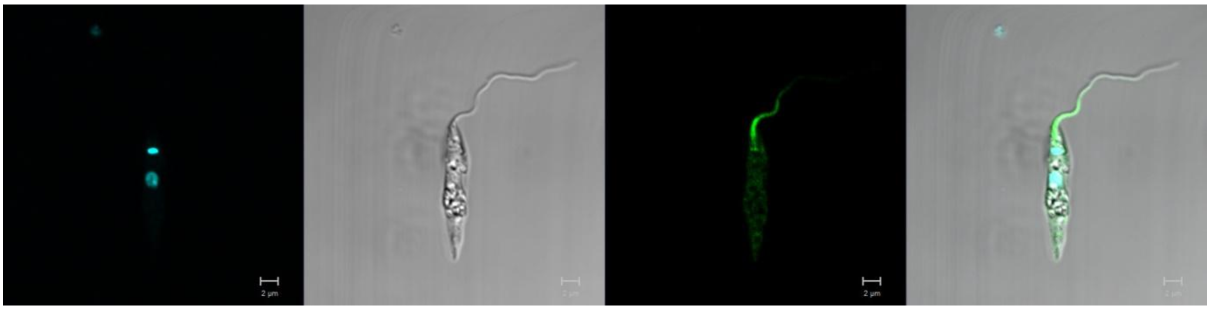

LmxM.31.1810
N-terminus

Primary localisation: cytoplasm

Secondary localisation: nucleus, flagellum

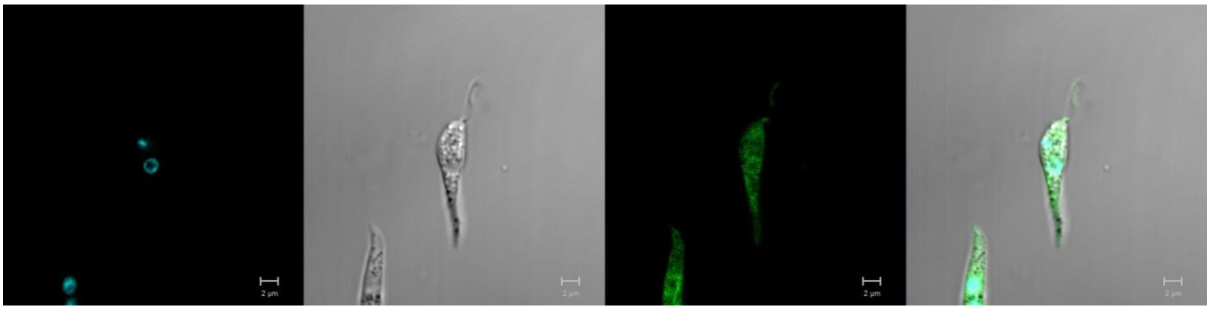

LmxM.32.1980
N-terminus

Primary localisation: endomembrane

Secondary localisation: cytoplasm

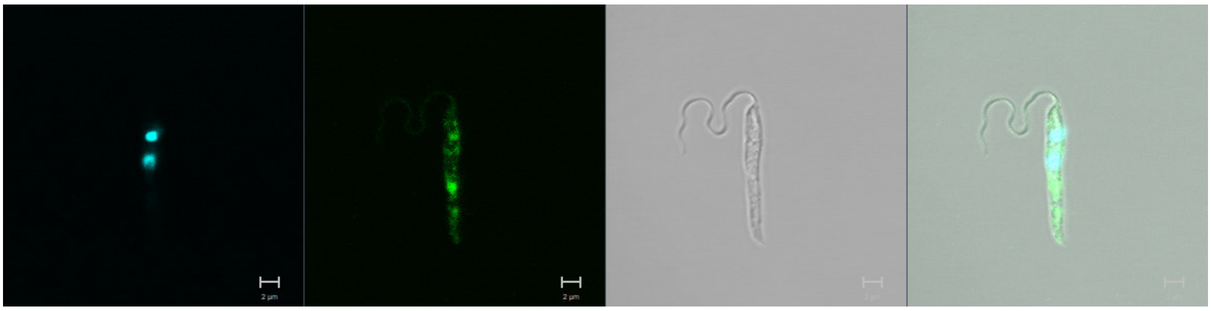

LmxM.34.5190
N-terminus

Primary localisation: cytoplasm

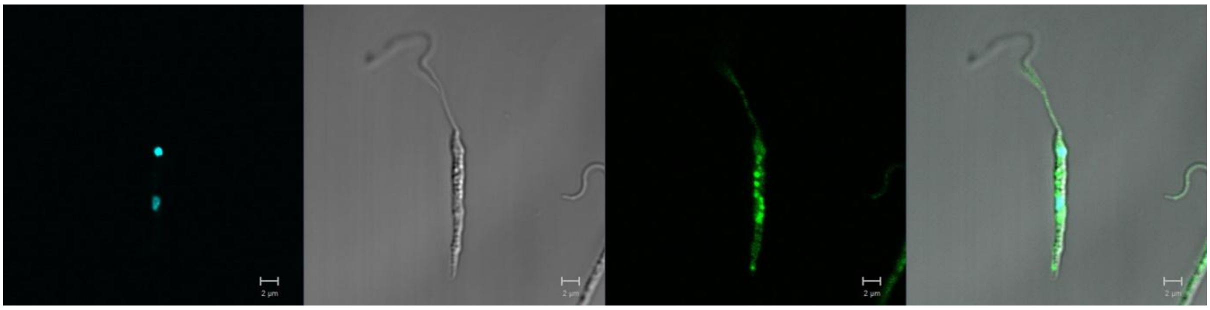

LmxM.36.1520
N-terminus

Primary localisation: lysosome

Secondary localisation: cytoplasm, flagellum

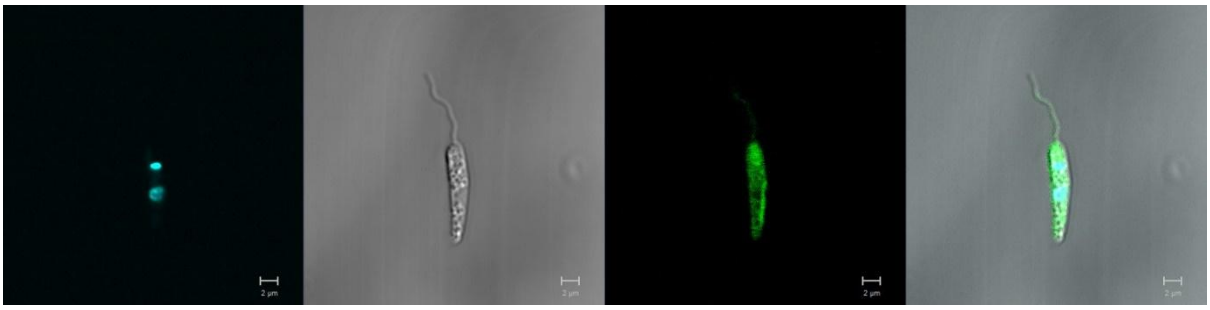

LmxM.36.1530
N-terminus

Primary localisation: cytoplasm

Secondary localisation: flagellum, endomembrane

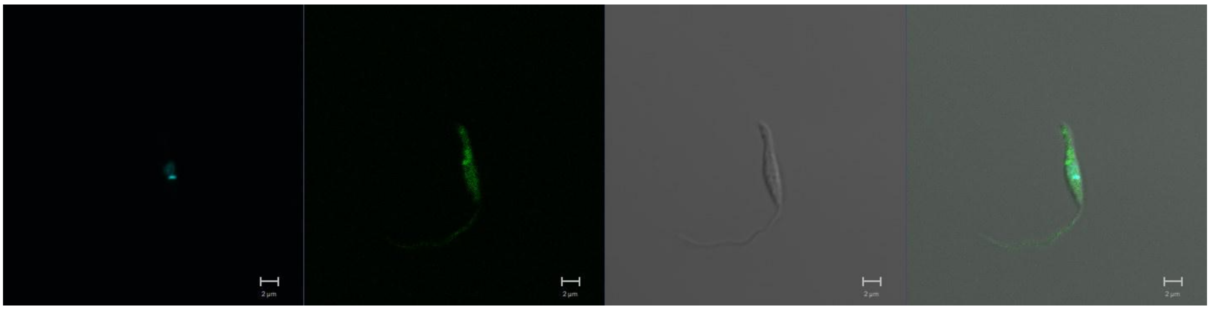

LmxM.36.2290
N-terminus

Primary localisation: lysosome

Secondary localisation: cytoplasm

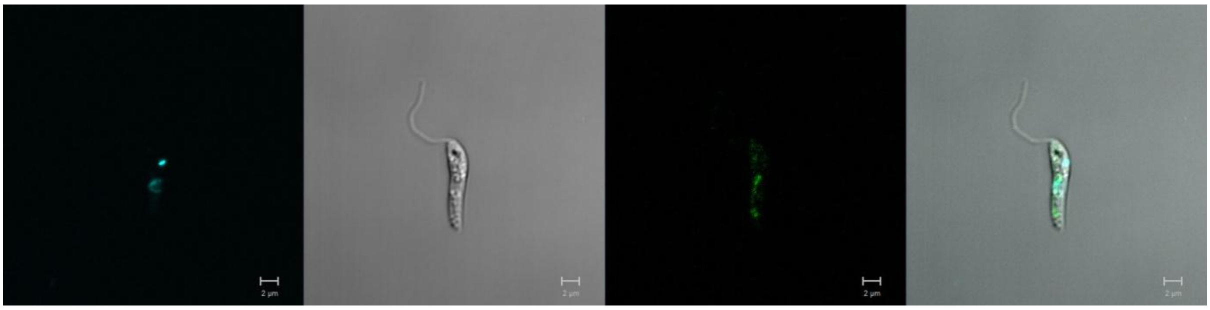

<u>ORPHAN FAMILY</u>

LmxM.02.0570
N-terminus

Primary localisation: flagellum

Secondary localisation: lysosome, cytoplasm

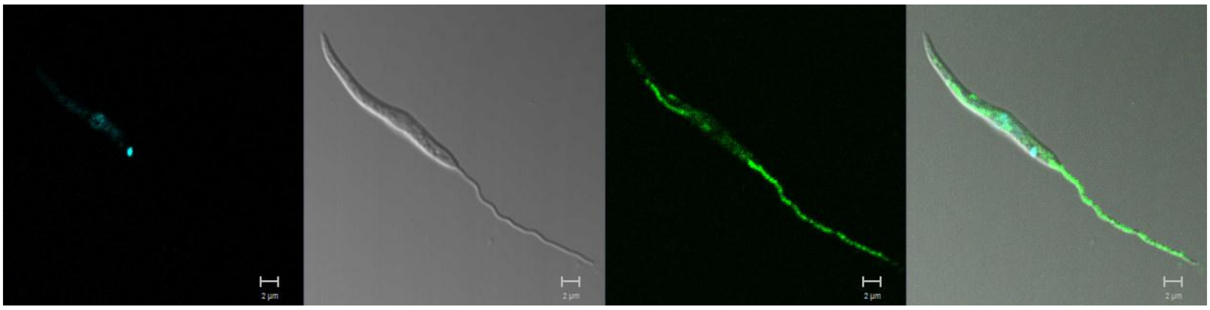

LmxM.03.0350
N-terminus

Primary localisation: cytoplasm

Secondary localisation: endomembrane

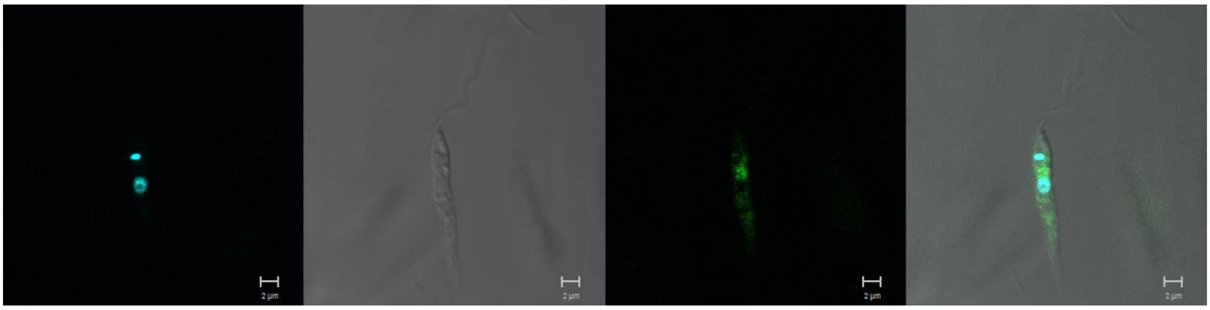

LmxM.10.0830
C-terminus

Primary localisation: nucleus

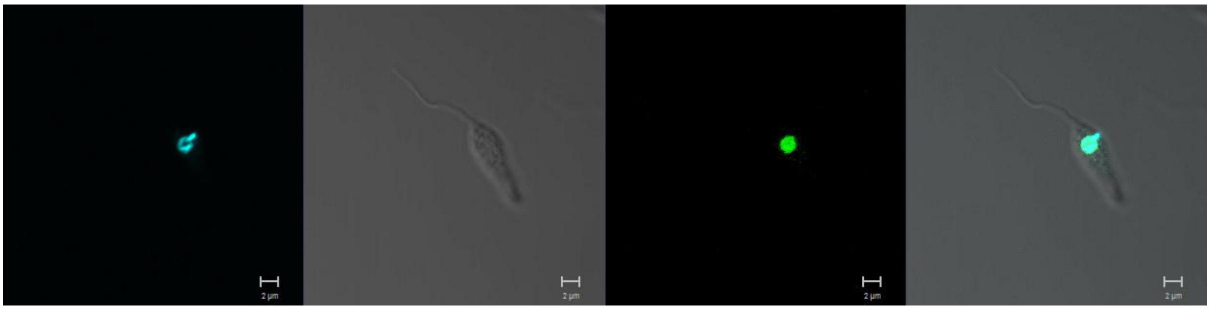

LmxM.11.0510
N-terminus

Primary localisation: lysosome

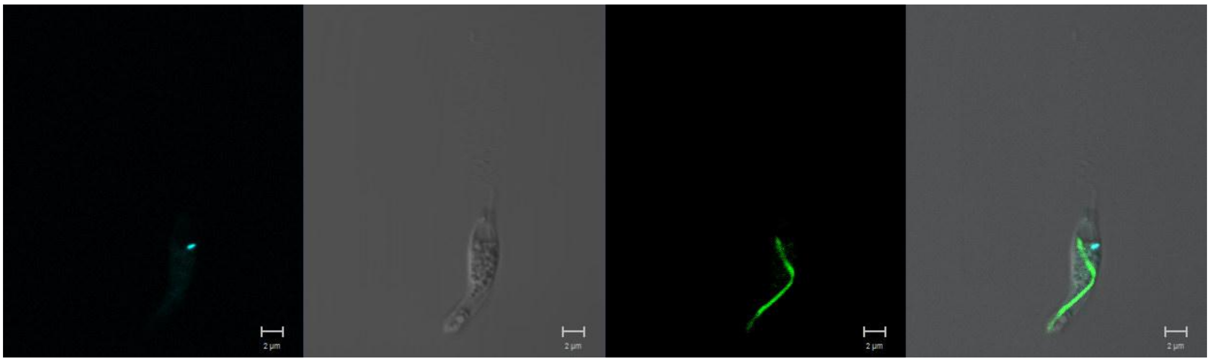

LmxM.16.0870
N-terminus

Primary localisation: cytoplasm

Secondary localisation: flagellum, nucleus, kinetoplast

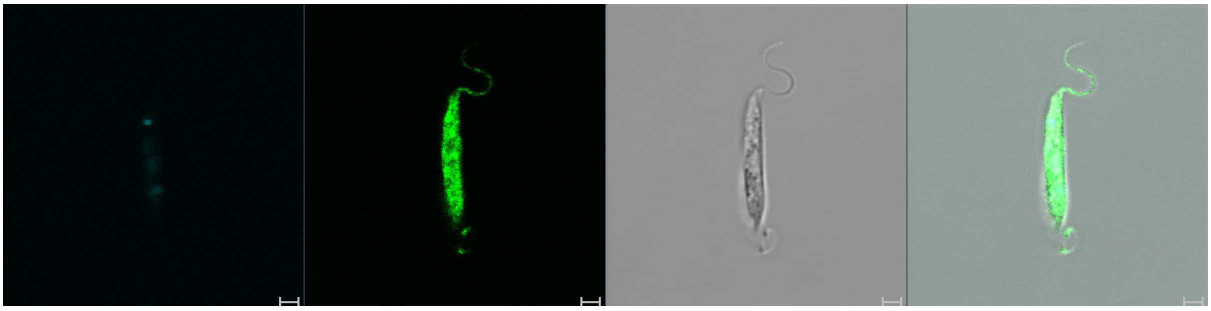

LmxM.25.1520
N-terminus

Primary localisation: endomembrane

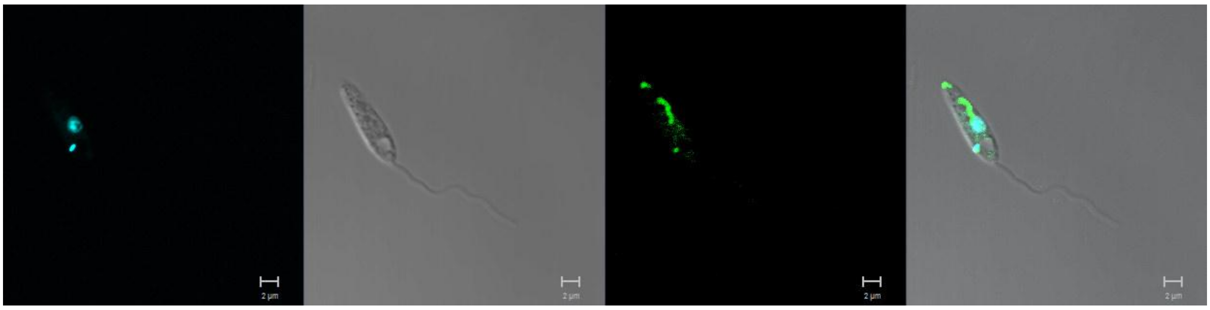

LmxM.26.2110
N-terminus

Primary localisation: lysosome

Secondary localisation: cytoplasm

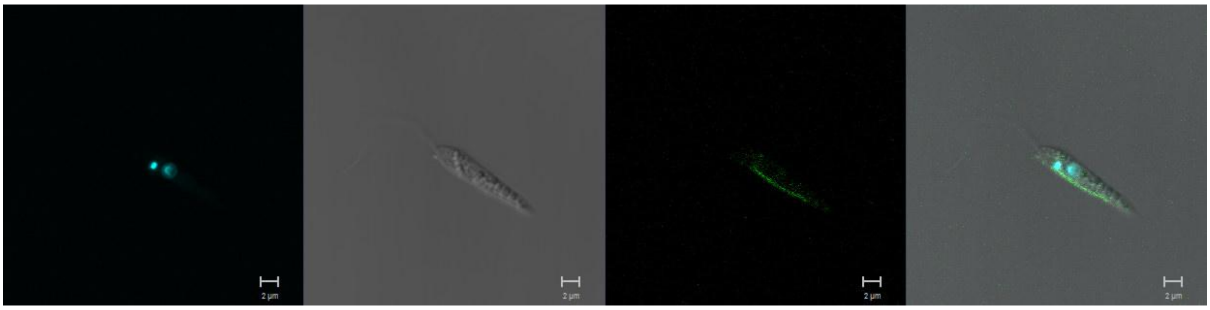

LmxM.08_29.2490
N-terminus

Primary localisation: cytoplasmic organelles

Secondary localisation: cytoplasm

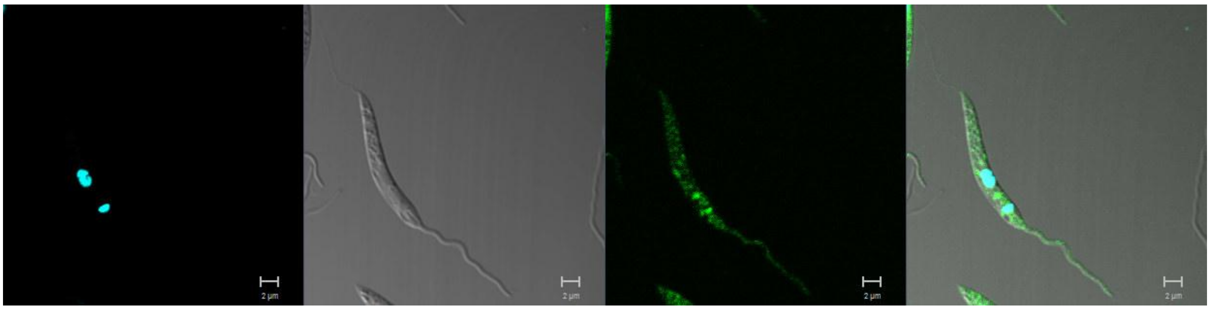

LmxM.34.4050 (KKT3)
C-terminus

Primary localisation: nucleus

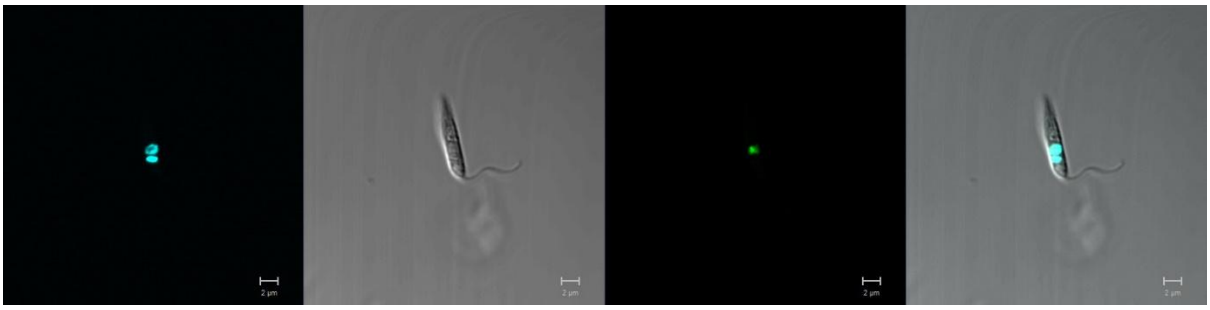

LmxM.36.5350 (KKT2)
C-terminus

Primary localisation: nucleus

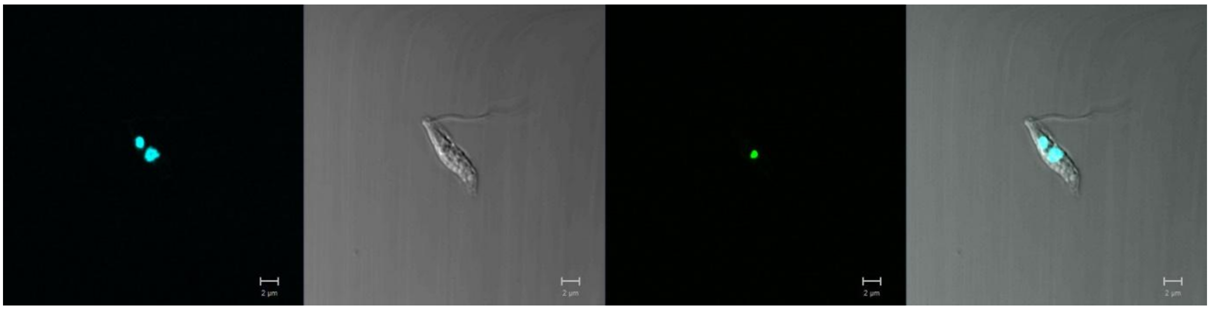

LmxM.34.4620
Not tagged
LmxM.08.0660
Not tagged
LmxM.26.2060
Not tagged
LmxM.28.1650
Not tagged
LmxM.31.1290
Not tagged
LmxM.33.2190
Not tagged

<u>OTHERS FAMILY</u>

LmxM.20.1330
N-terminus

Primary localisation: endomembrane

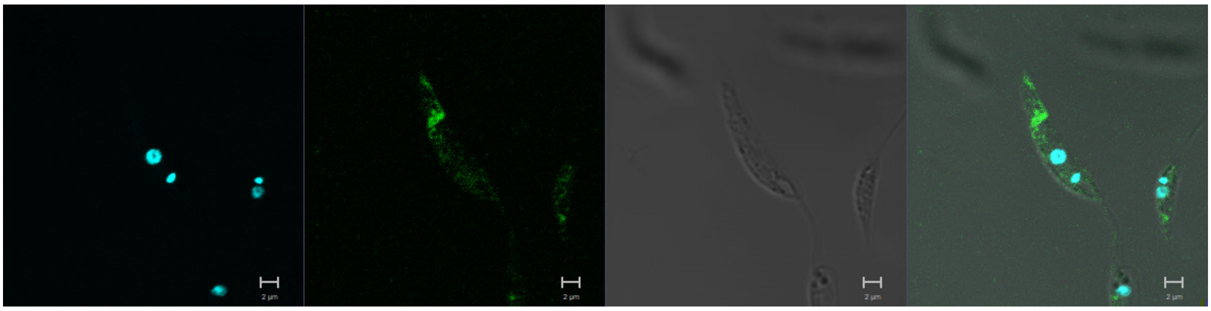

LmxM.20.1340
N-terminus

Primary localisation: cytoplasm

Secondary localisation: endomembrane

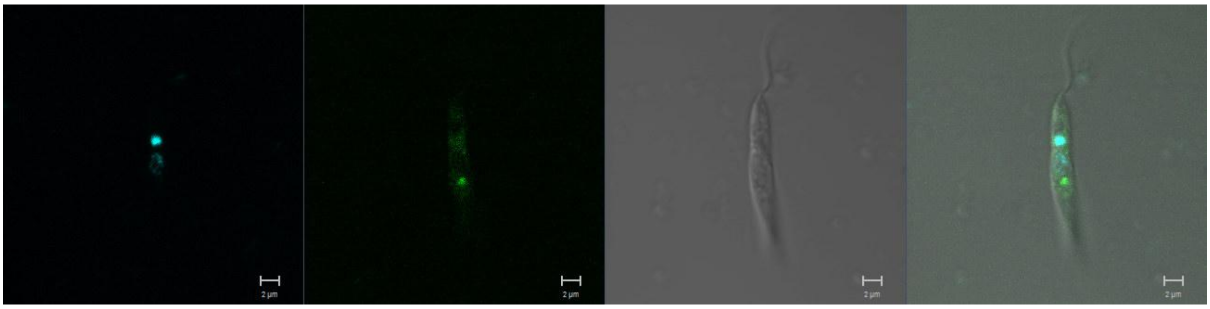

LmxM.08_29.0370
N-terminus

Primary localisation: cytoplasm

Secondary localisation: flagellum, endomembrane

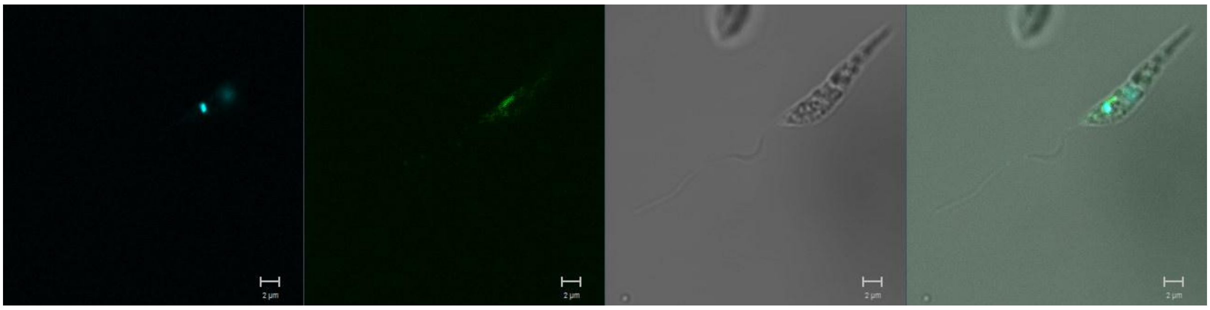

LmxM.34.2870
N-terminus

Primary localisation: cytoplasm

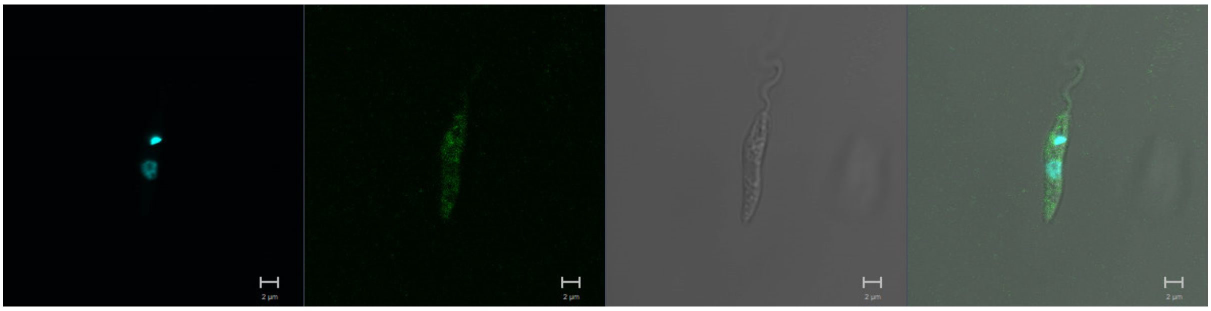

LmxM.26.2440 (AUK3)
N-terminus

Primary localisation: nucleus

Secondary localisation: endomembrane

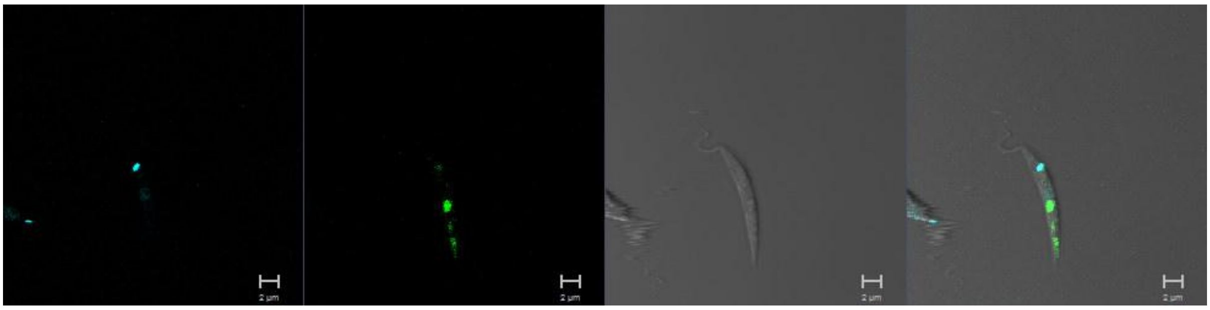

LmxM.28.0520 (AIRK)
N-terminus

Primary localisation: nucleus

Secondary localisation: endomembrane

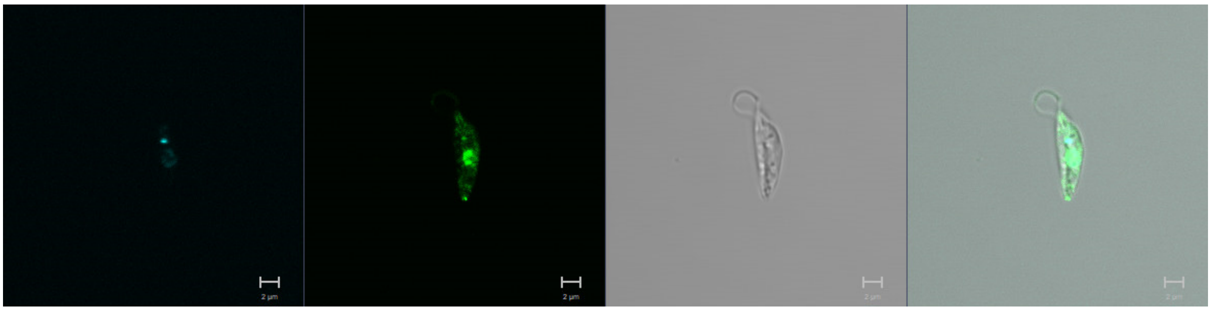

LmxM.08_29.1330 (AUK2)
N-terminus

Primary localisation: cytoplasm

Secondary localisation: endomembrane

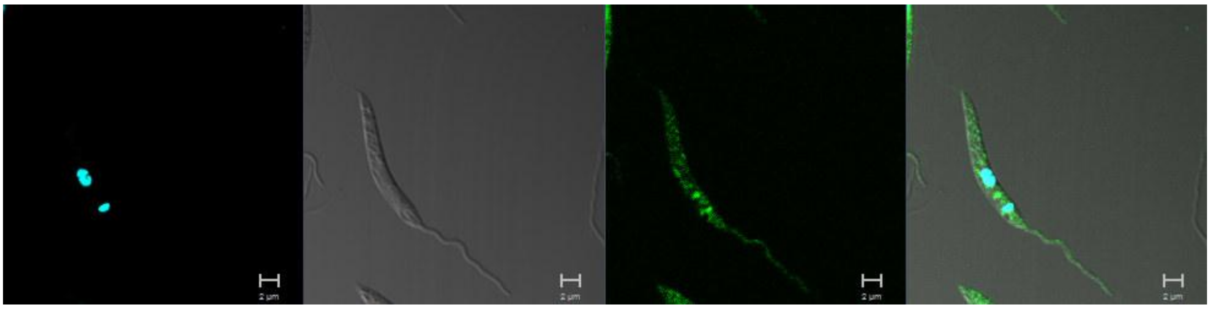

LmxM.19.0590
N-terminus

Primary localisation: cytoplasm

Secondary localisation: endomembrane

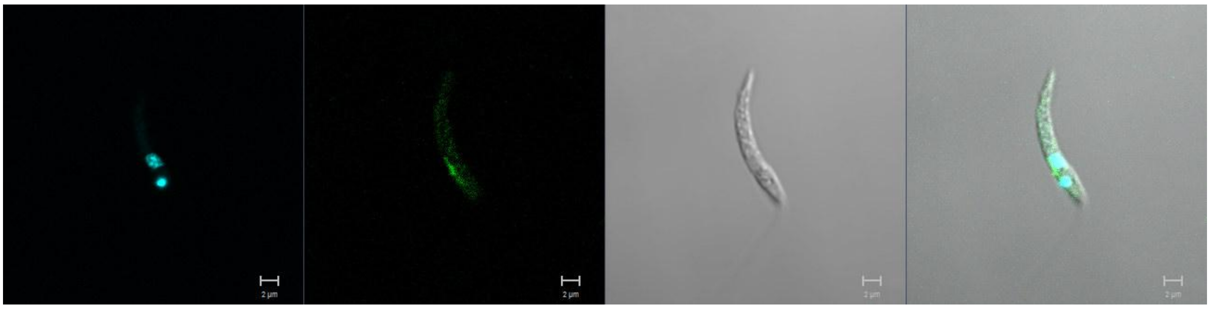

LmxM.11.0250
N-terminus

Primary localisation: cytoplasm

Secondary localisation: lysosome

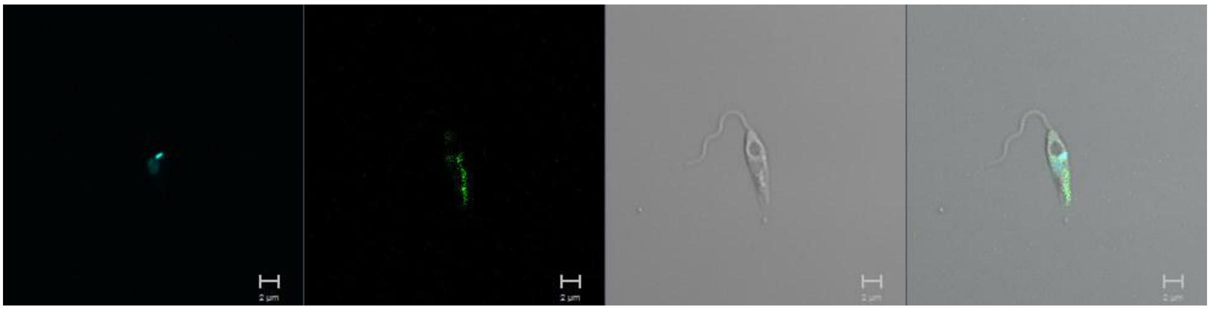

LmxM.24.1730
N-terminus

Primary localisation: cytoplasm

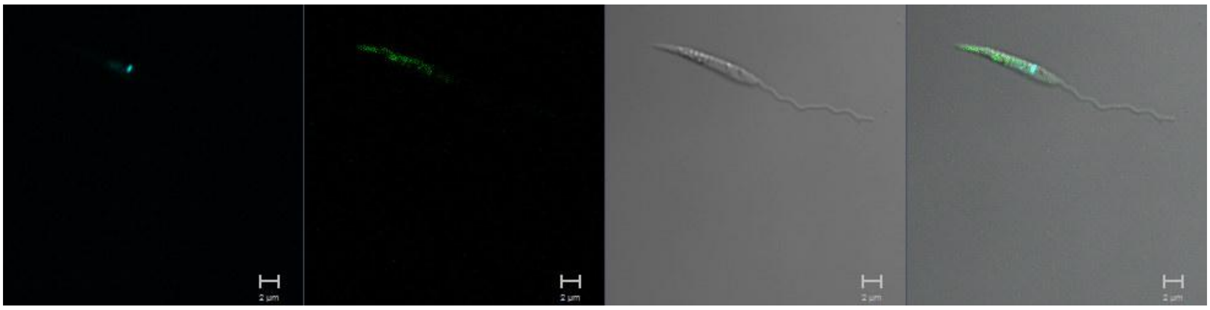

LmxM.34.2320
N-terminus

Primary localisation: flagellum

Secondary localisation: cytoplasm

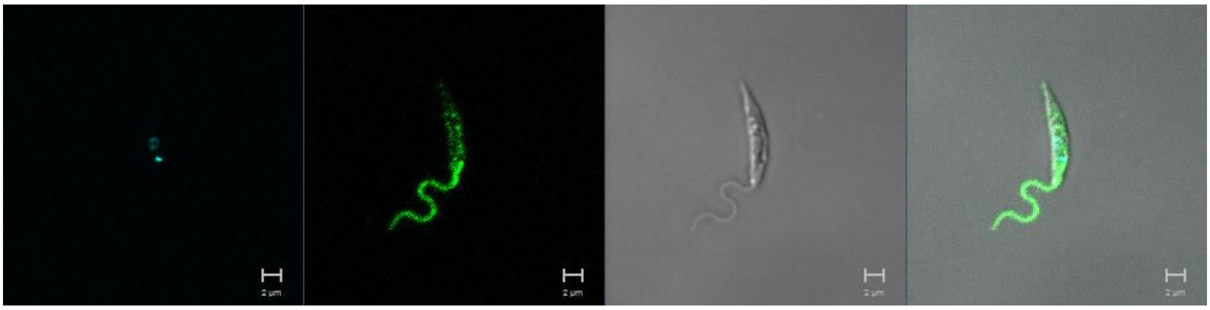

LmxM.34.1730 (CK2A1)
N-terminus

Primary localisation: cytoplasm

Secondary localisation: nucleus, kinetoplast

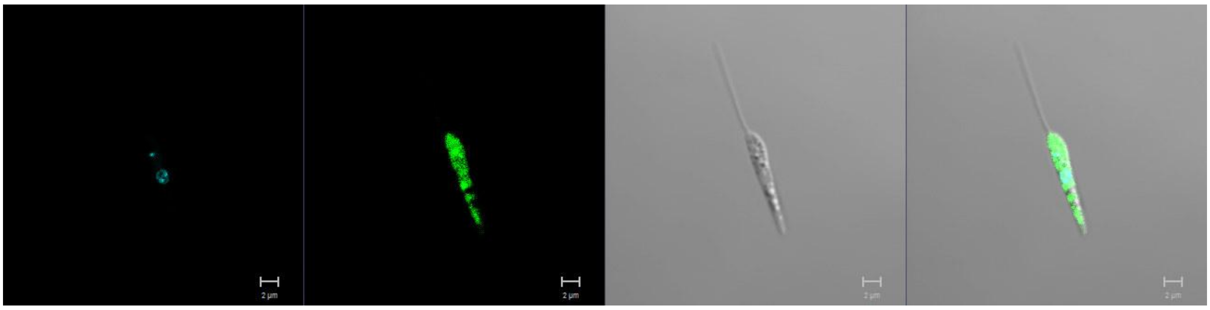

LmxM.02.0360 (CK2A2)
N-terminus

Primary localisation: nucleus

Secondary localisation: lysosome, cytoplasm

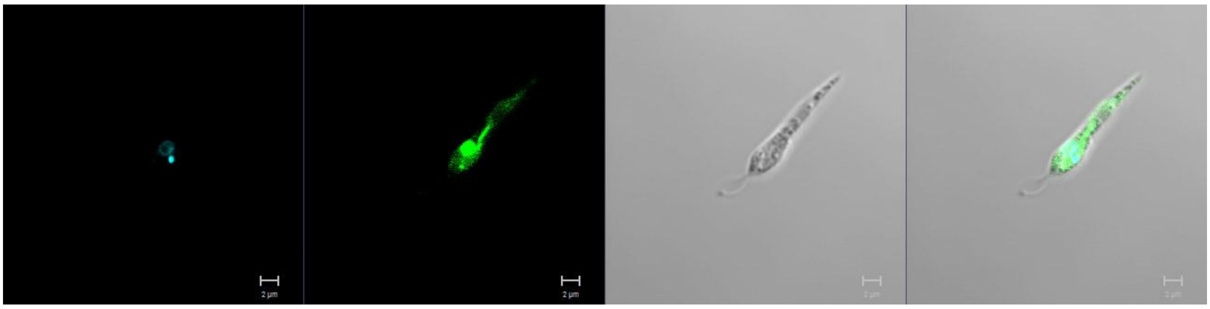

LmxM.15.0770
N-terminus

Primary localisation: cytoplasm

Secondary localisation: flagellum, endomembrane

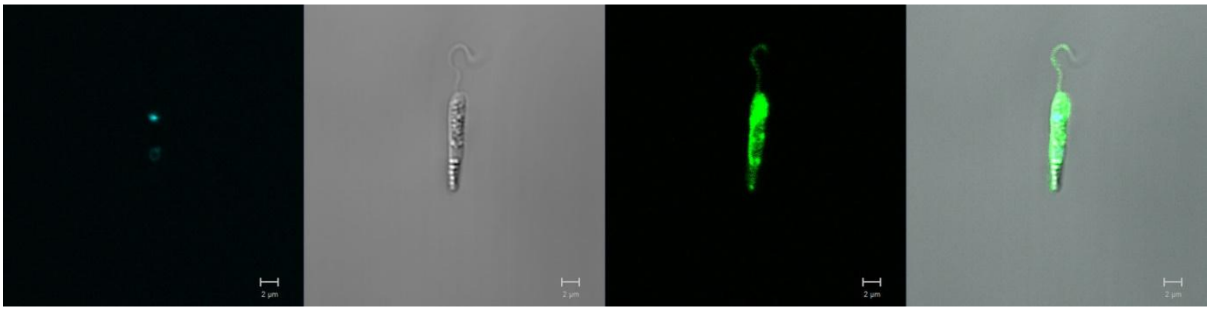

LmxM.33.0030
N-terminus

Primary localisation: cytoplasm

Secondary localisation: flagellum

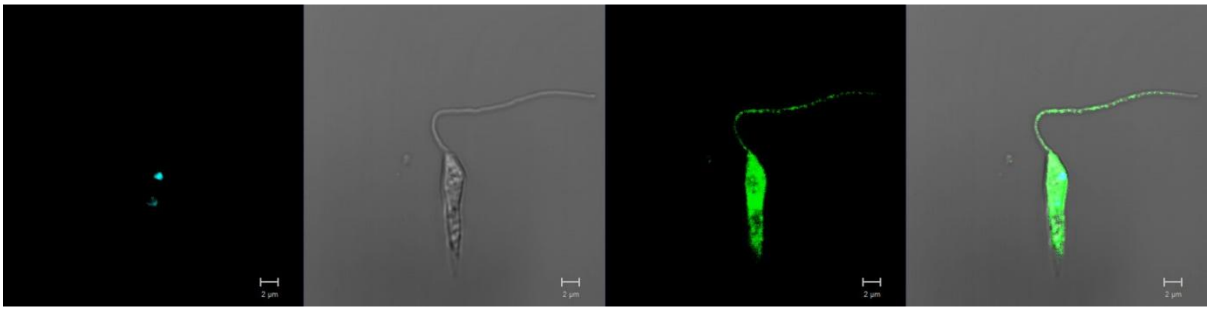

LmxM.11.0060 (EF2A1)
N-terminus

Primary localisation: cytoplasm

Secondary localisation: endoplasmic reticulum

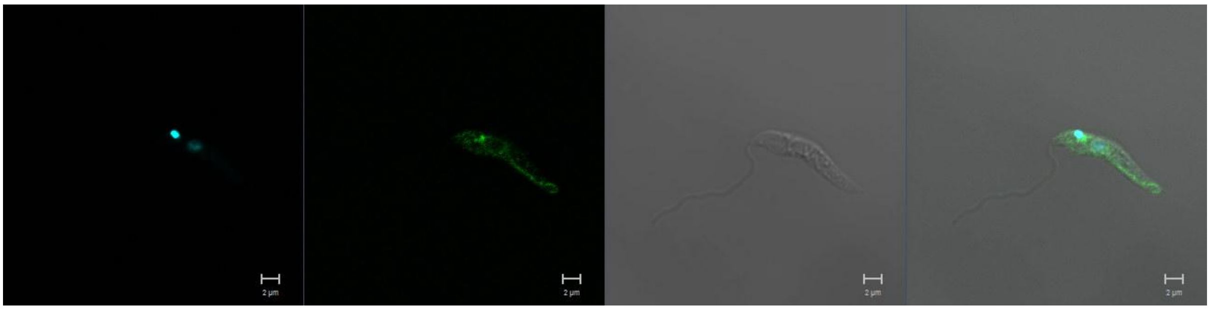

LmxM.29.1560 (EF2A3)
N term - N-terminus

Primary localisation: cytoplasm (low expression)

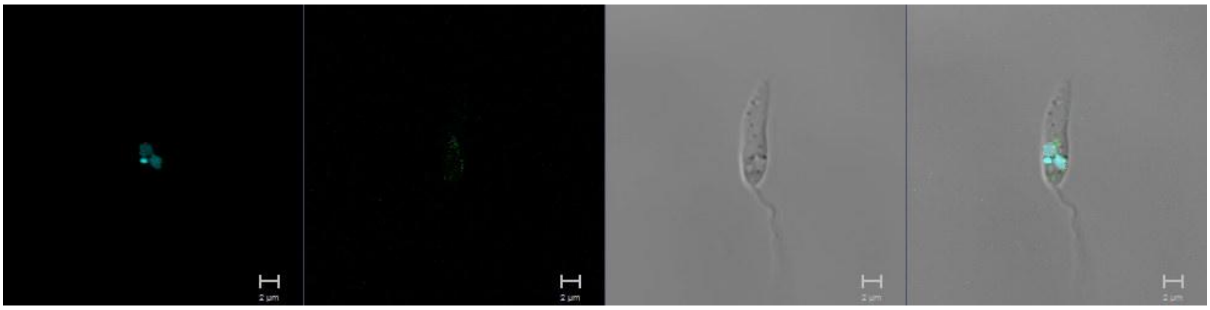

LmxM.33.2150 (EF2A2)
N-terminus

Primary localisation: cytoplasmic organelles

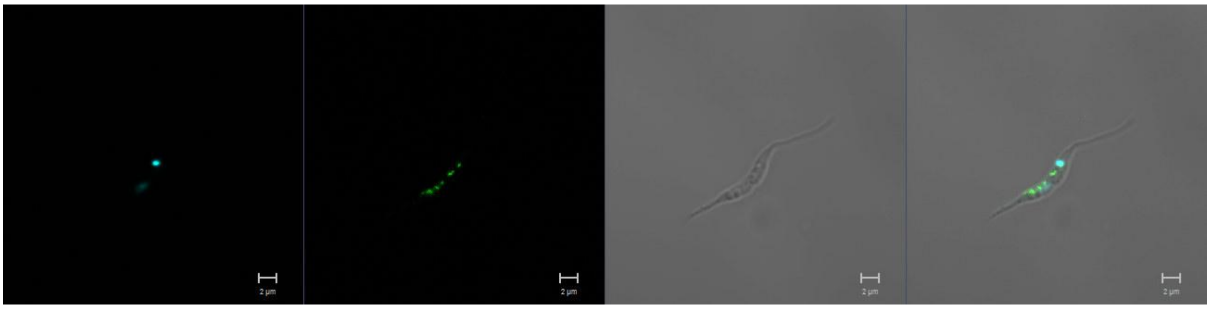

LmxM.17.0790 (PKL)
N-terminus

Primary localisation: cytoplasm (low expression)

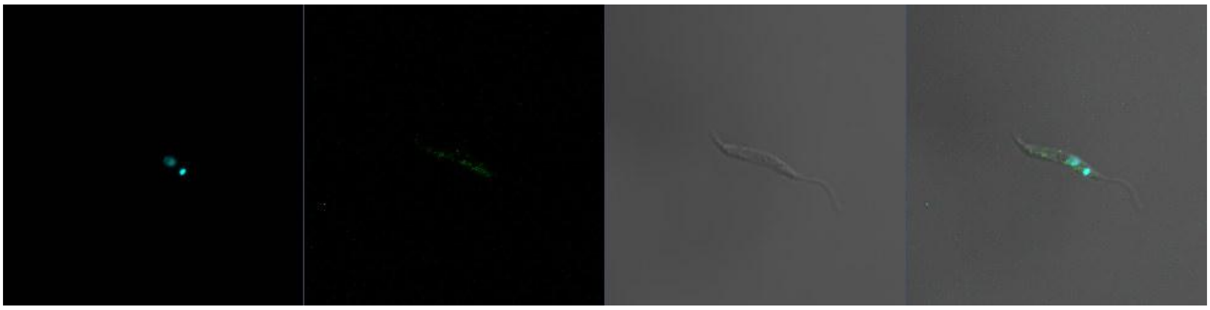

C-terminus

Primary localisation: basal body, endomembrane, cytoplasm (specific to early G1-phase)

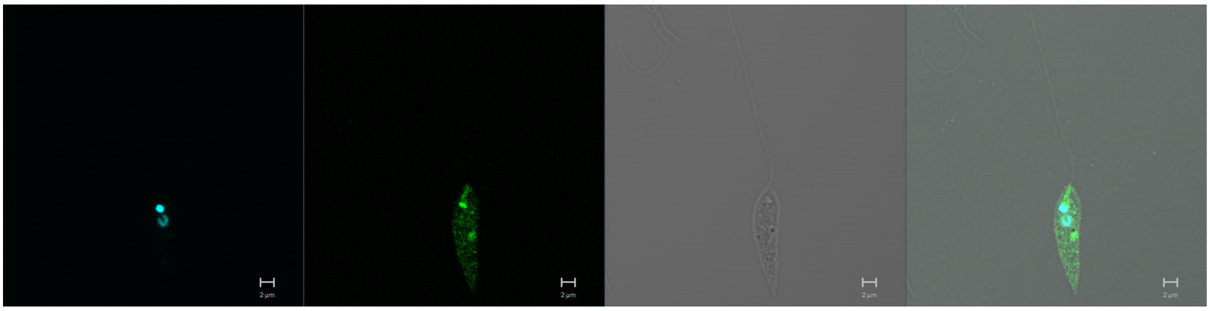

LmxM.30.2860 (TLK)
N-terminus

Primary localisation: cytoplasm (low expression)

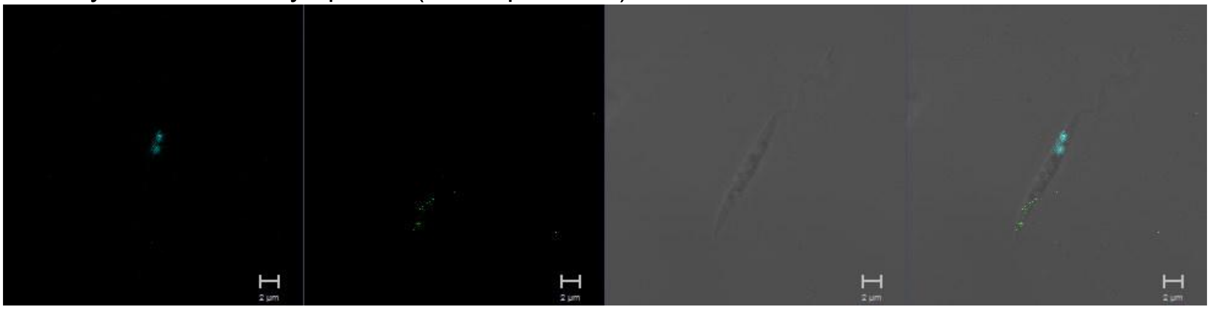

C-terminus

Primary localisation: nucleus

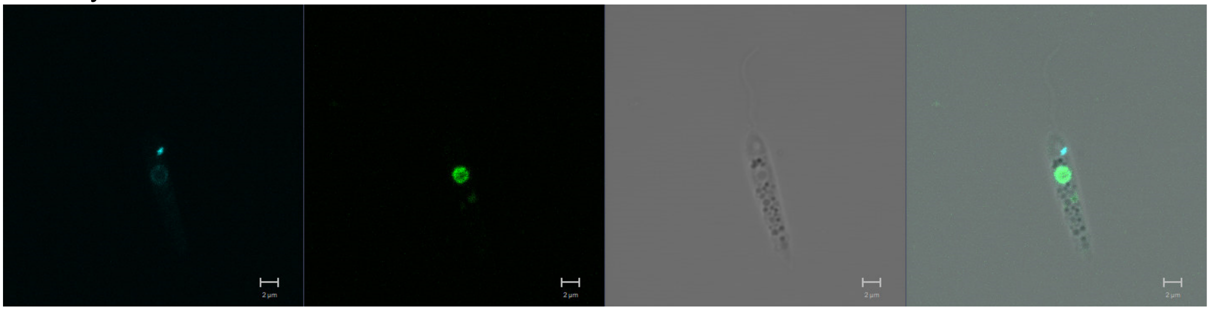

LmxM.28.0620 (ULK4)
N-terminus

Primary localisation: no signal

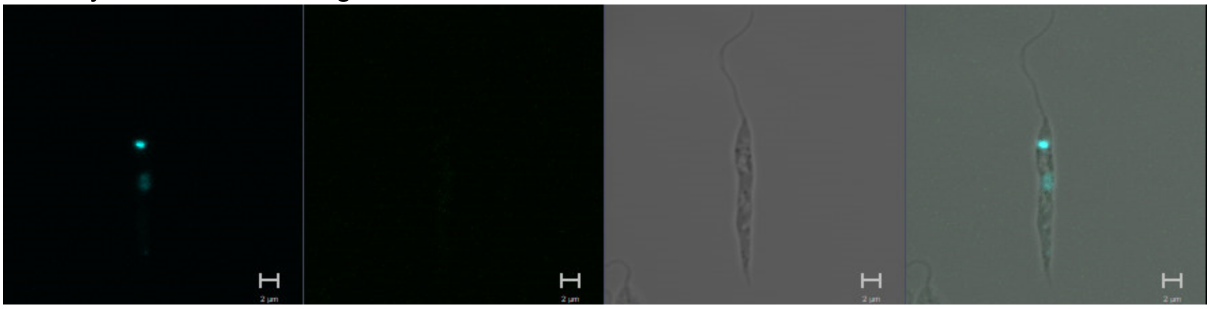

C-terminus

Primary localisation: basal body

Secondary localisation: flagellum tip, cytoplasm, flagellum, nucleus

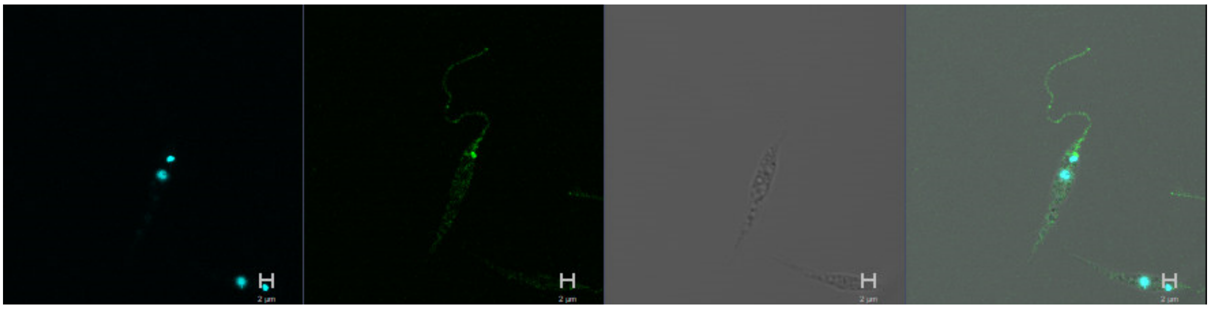

LmxM.13.0440 (STK36)
N-terminus

Primary localisation: no signal

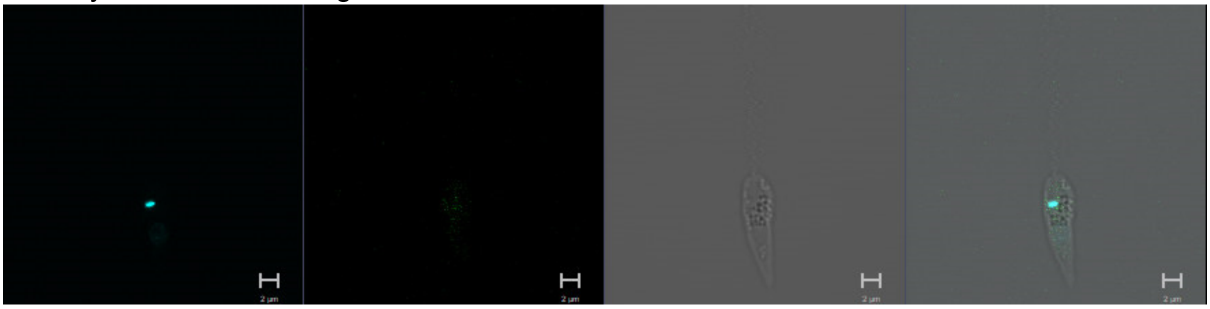

C-terminus

Primary localisation: basal body

Secondary localisation: flagellum tip, cytoplasm, flagellum, nucleus

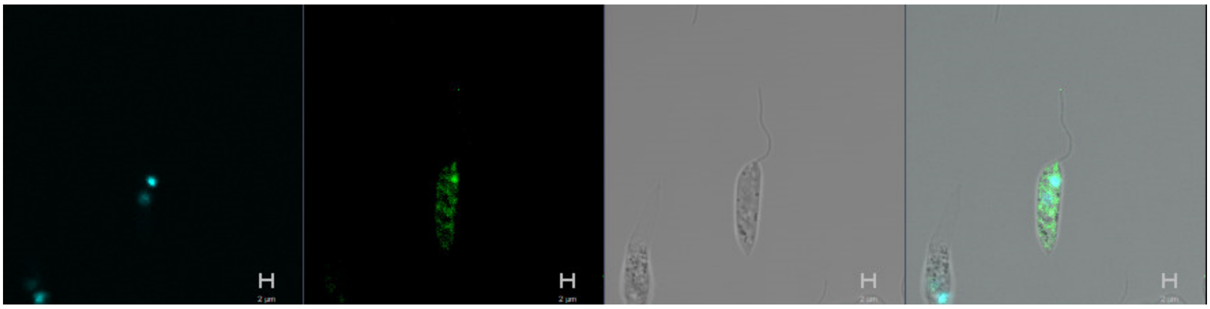

LmxM.28.1760
N-terminus

Primary localisation: failed attempts

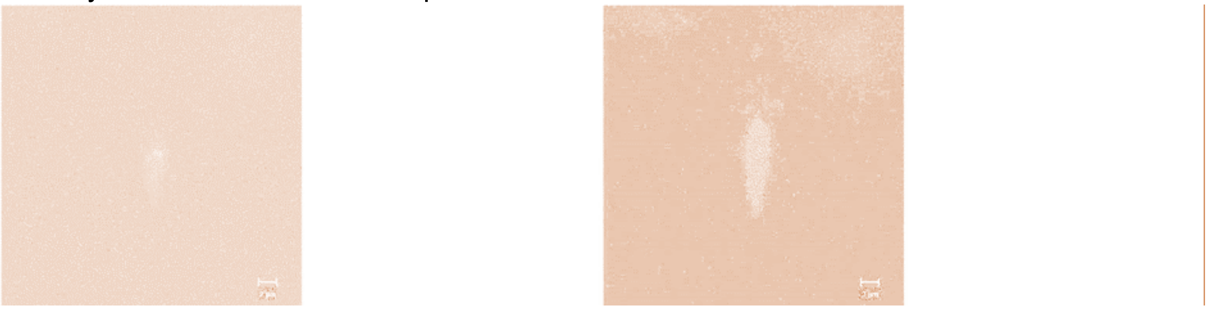

LmxM.08_29.2720
N-terminus

Primary localisation: cytoplasm

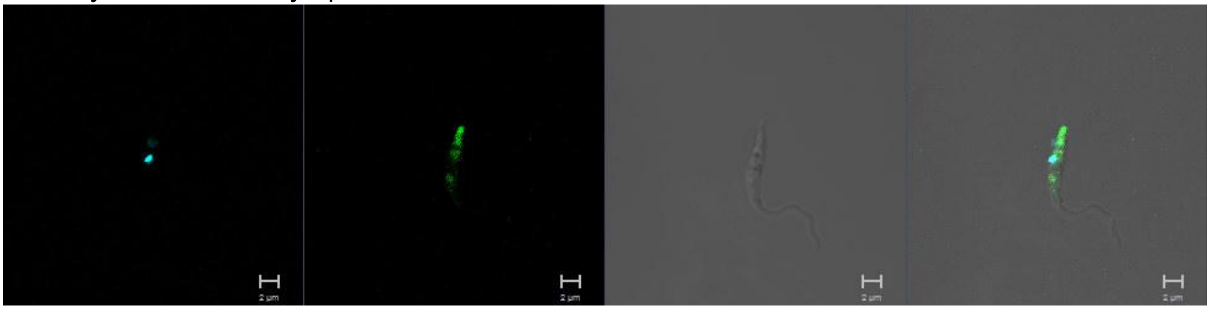

LmxM.33.0940 (WEE1)
N-terminus

Primary localisation: cytoplasm

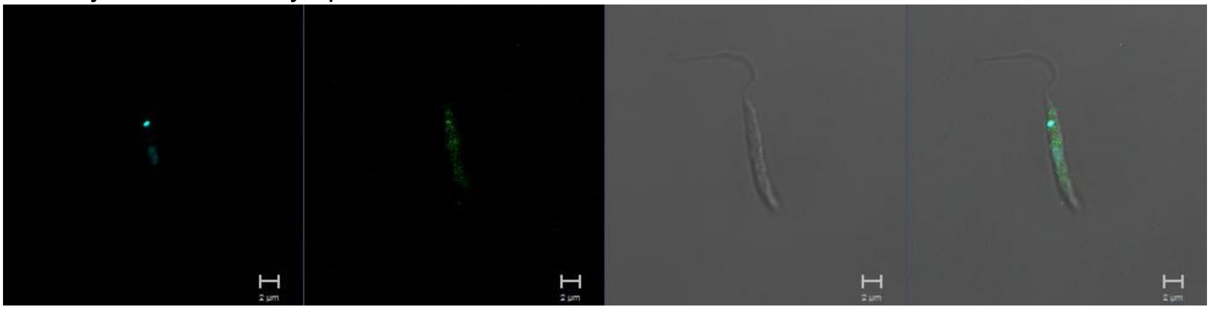

LmxM.08.0530
N-terminus

Primary localisation: endomembrane

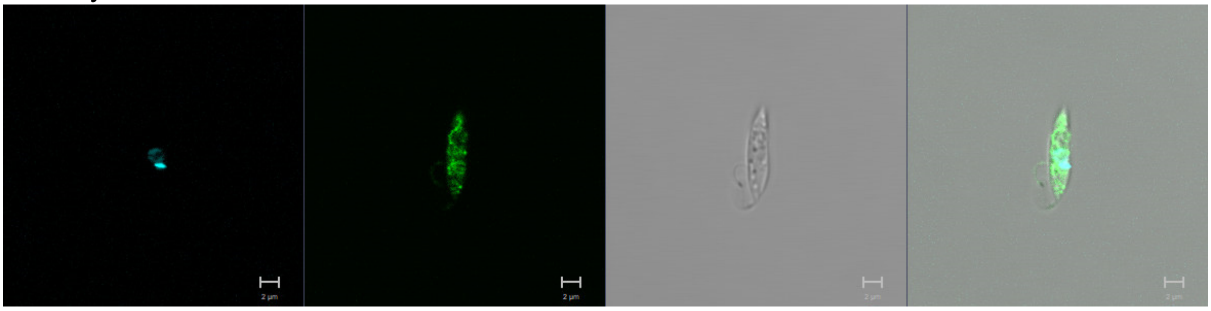

LmxM.20.0960
N-terminus

Primary localisation: cytoplasm (low expression)

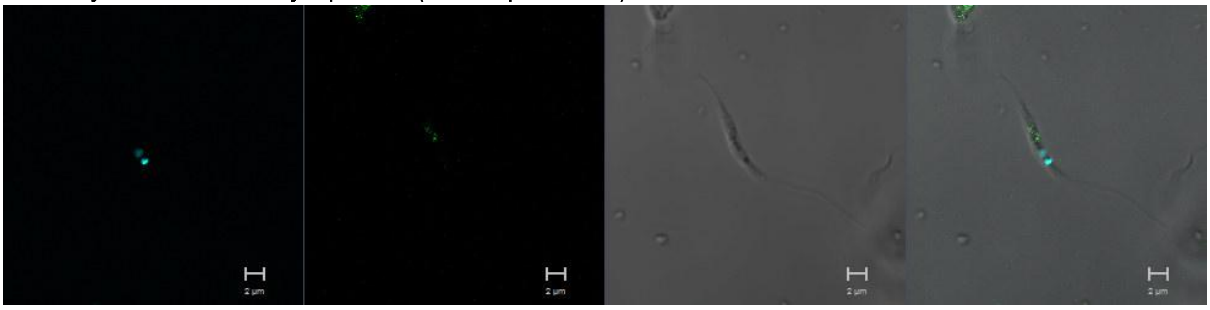

C-terminus

Primary localisation: endomembrane

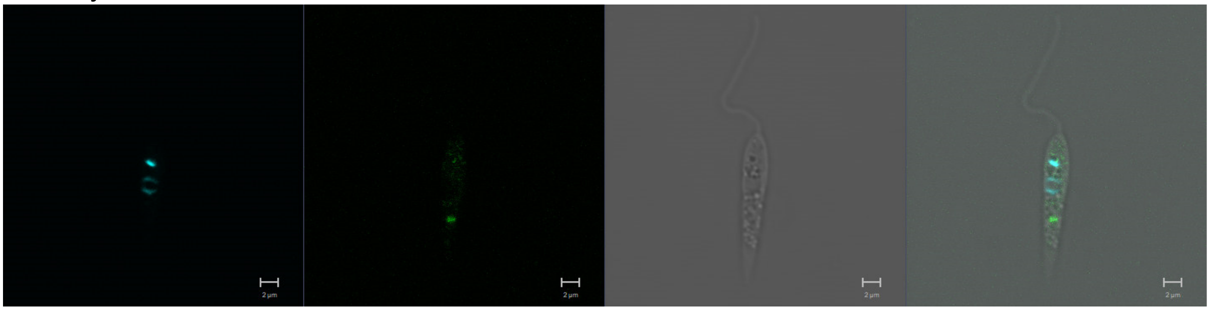

LmxM.21.0823
N-terminus

Primary localisation: lysosome

Secondary localisation: endomembrane

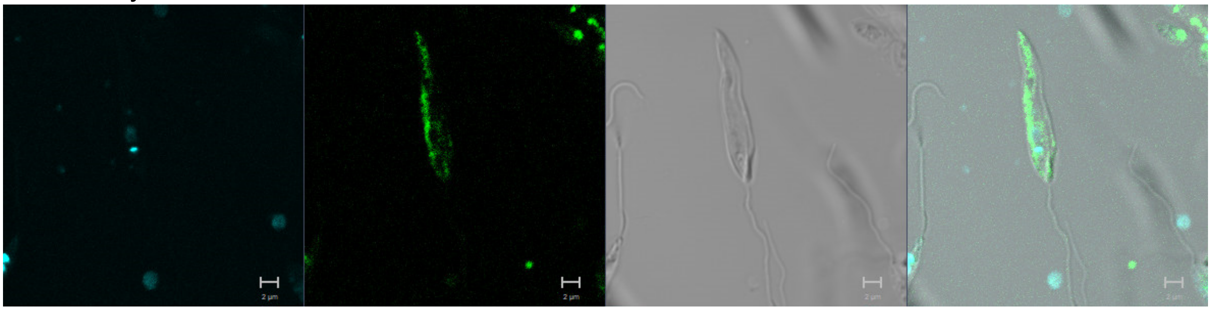

LmxM.36.2630
N-terminus

Primary localisation: endomembrane

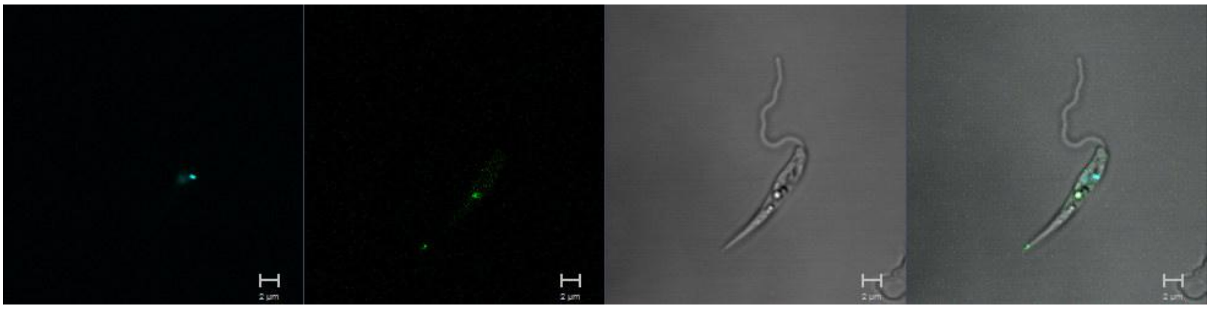

LmxM.22.1150
N-terminus

Primary localisation: cytoplasm

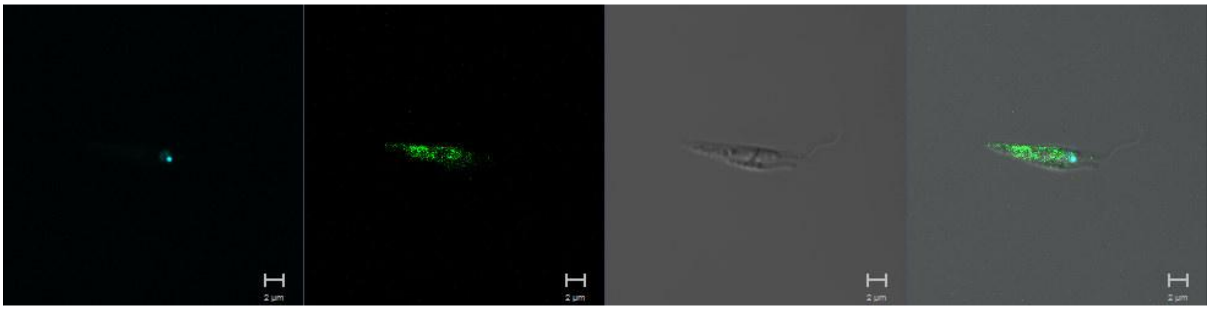

ATYPICAL PROTEIN KINASES

LmxM.08_29.1450 (PIK)
N-terminus

Primary localisation: basal body

Secondary localisation: cytoplasm

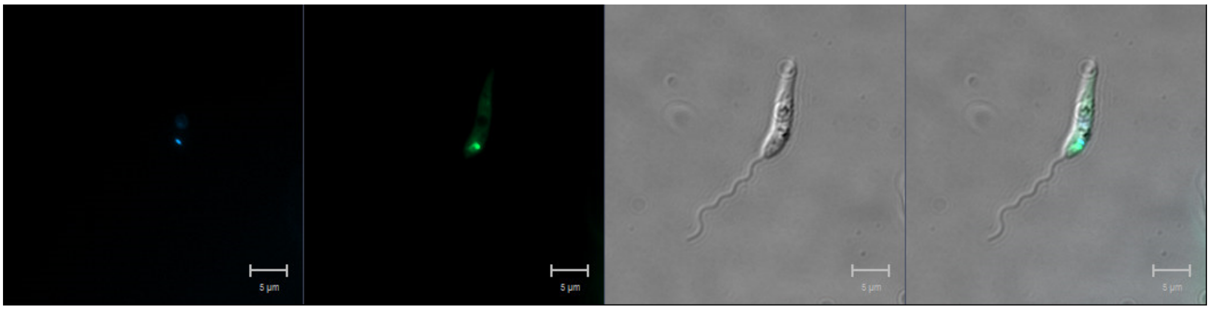

N-terminus

Primary localisation: lysosome

Secondary localisation: endomembrane

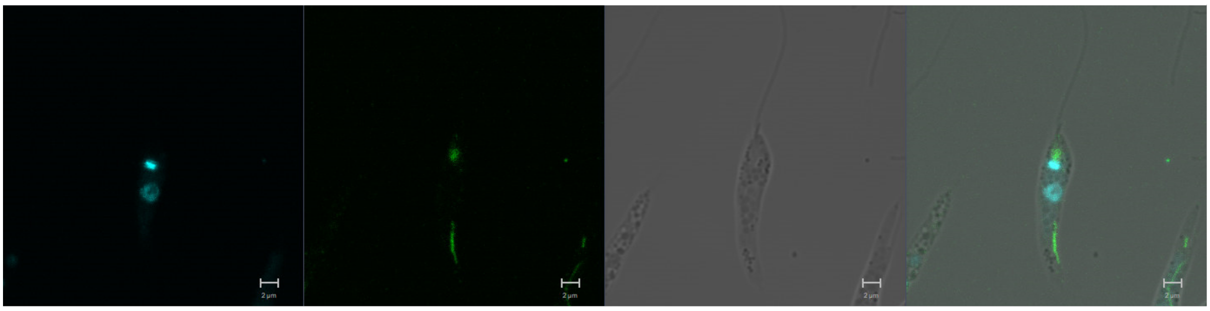

LmxM.24.2010 (PI3K)
N-terminus

Primary localisation: cytoplasm

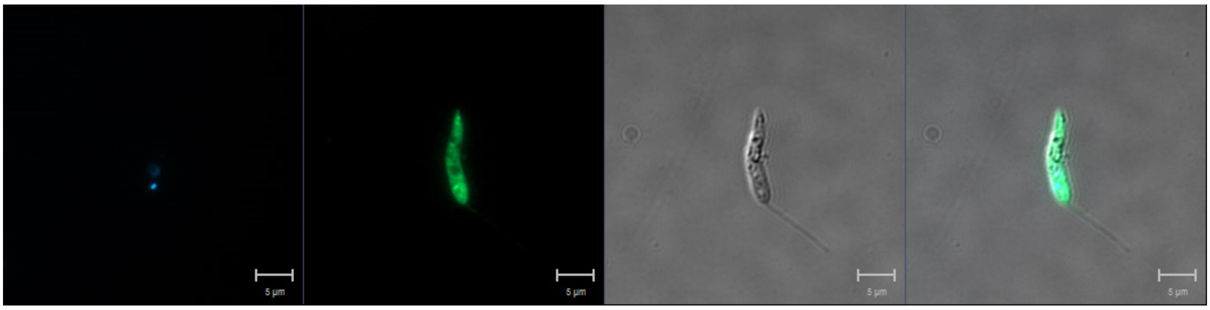

LmxM.33.3590 (PI4K)
N-terminus

Primary localisation: cytoplasm

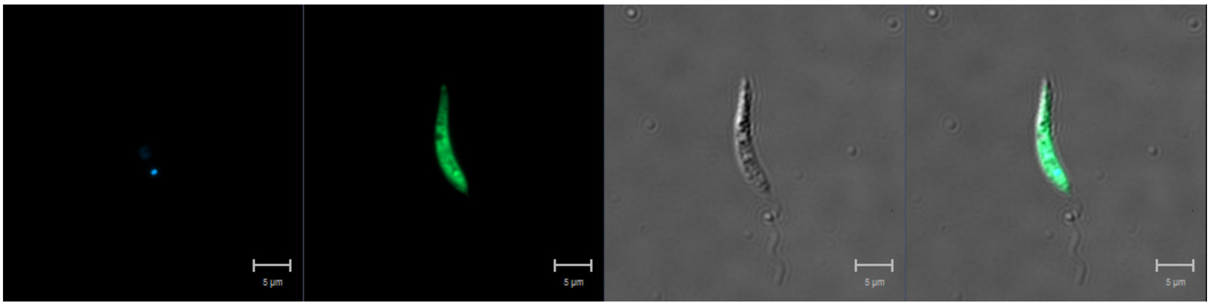

LmxM.27.0890 (PI3,5K)
N-terminus

Primary localisation: cytoplasm

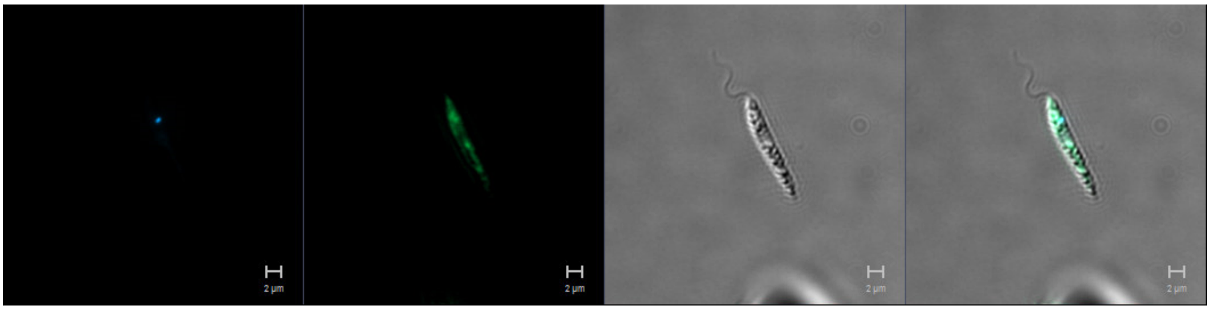

LmxM.33.3090 (PIPKalpha)
C-terminus

Primary localisation: cytoplasm

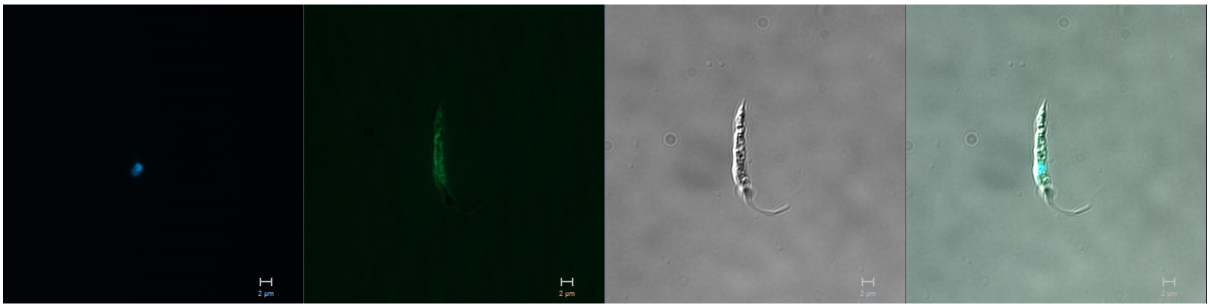

LmxM.34.0560 (PIP5K)
N-terminus

Primary localisation: flagellar pocket

Secondary localisation: cytoplasm

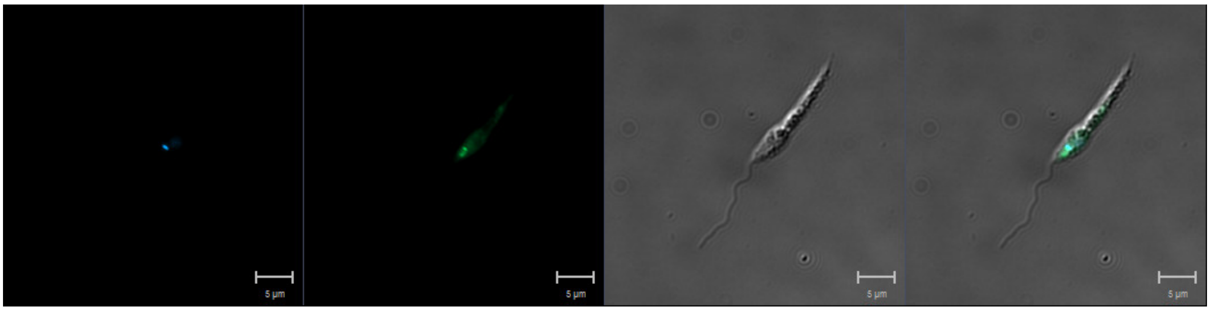

LmxM.36.6320 (TOR1)
N-terminus

Primary localisation: cytoplasm

Secondary localisation: endomembrane

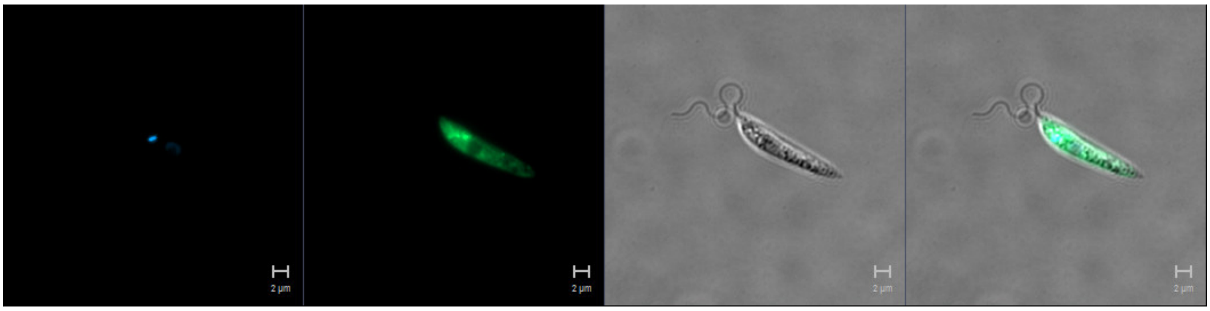

LmxM.33.4530 (TOR2)
N-terminus

Primary localisation: cytoplasmic organelles

Secondary localisation: cytoplasm

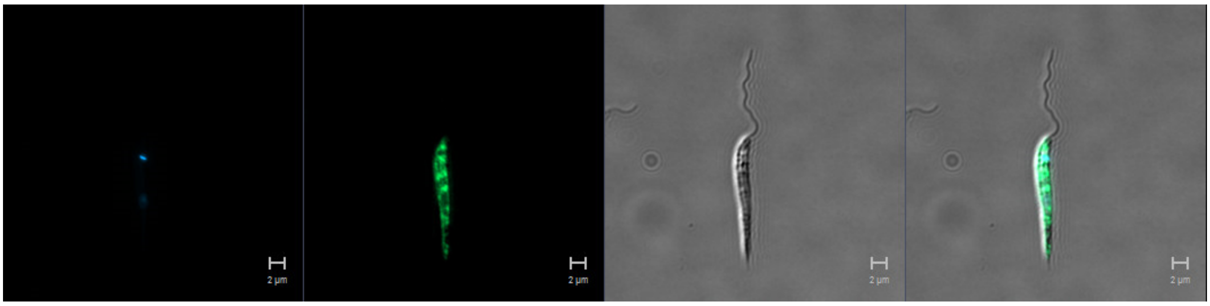

LmxM.33.3940 (TOR3)
N-terminus

Primary localisation: cytoplasm

Secondary localisation: kinetoplast

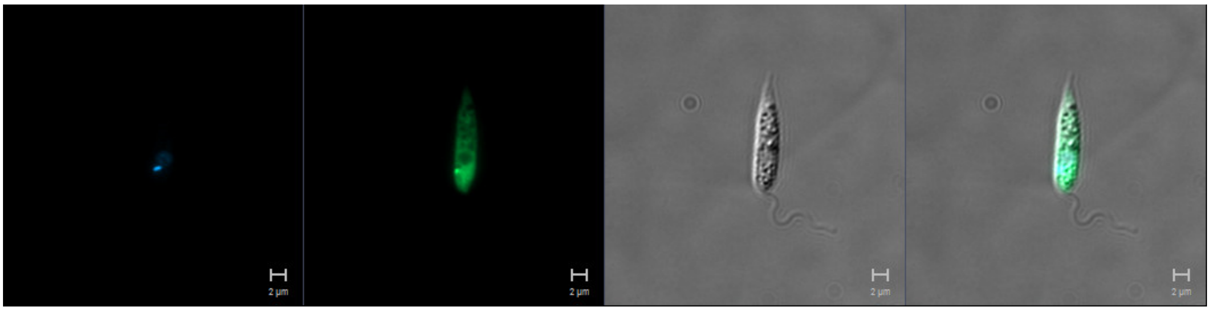

LmxM.02.0120 (ATM)
N-terminus

Primary localisation: nucleus

Secondary localisation: cytoplasm

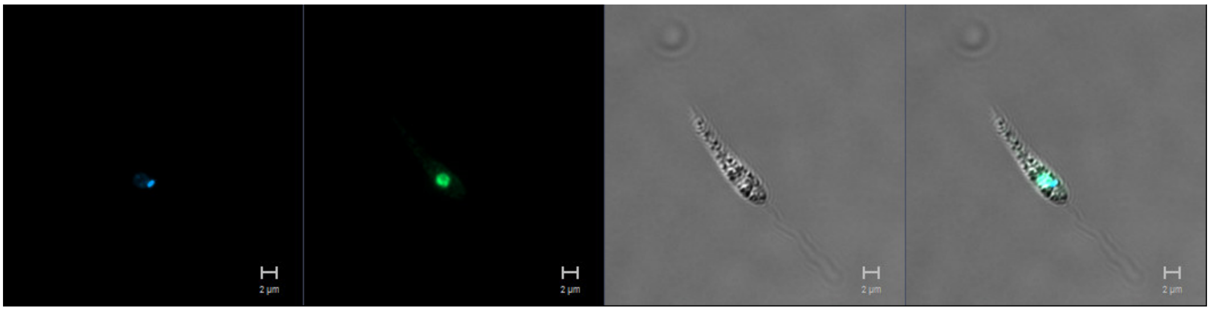

LmxM.31.1460 (ATR)
C-terminus

Primary localisation: cytoplasm

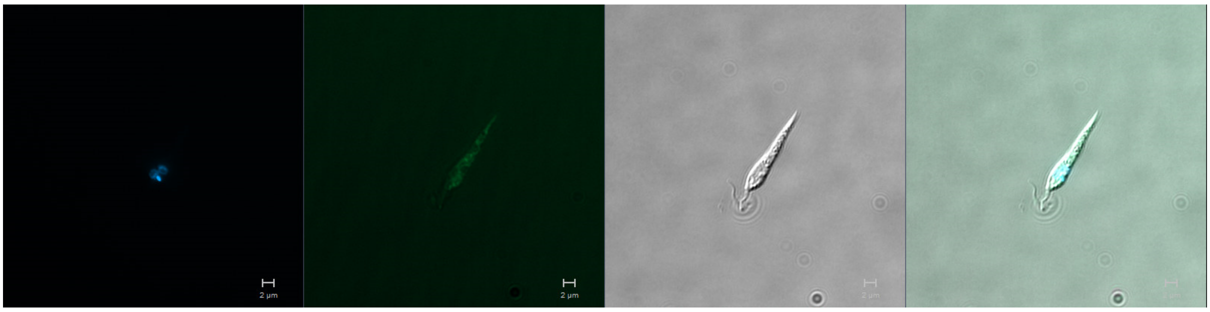

Supplement: Supplementary file 6 — Supplementary Data 3 [file 41467_2021_21360_MOESM6_ESM.pdf]
