# Supplementary material for: Systematic functional analysis of Leishmania protein kinases identifies regulators of differentiation or survival
Source: Nat Commun. 2021 Feb 23;12:1244. doi: 10.1038/s41467-021-21360-8 (PMC7902614; doi:10.1038/s41467-021-21360-8)

Supplementary Data 8. **Heat maps and cluster analysis for amastigotes.**

Supplementary Data 8a

Heat maps for Axenic amastigote data

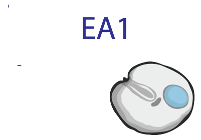

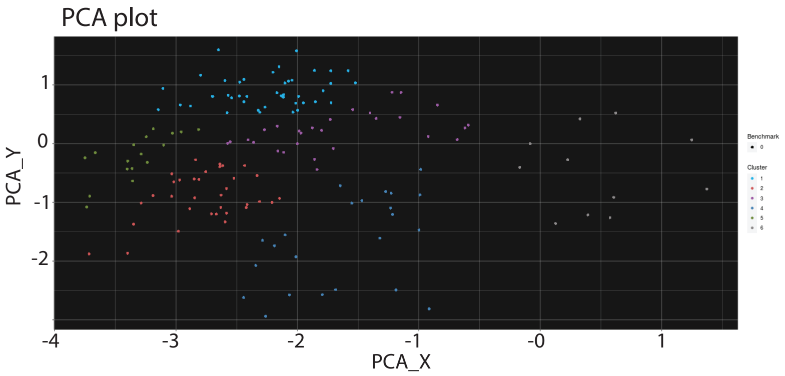

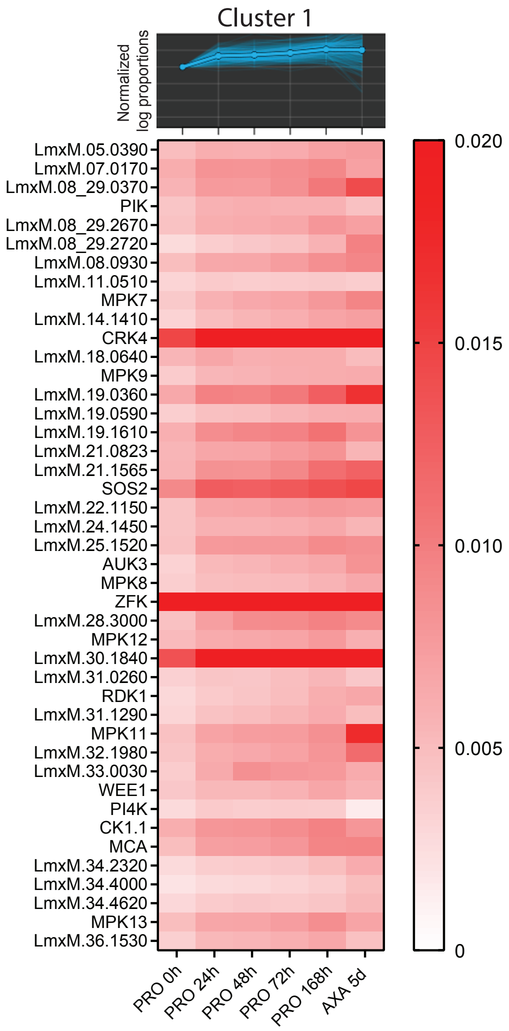

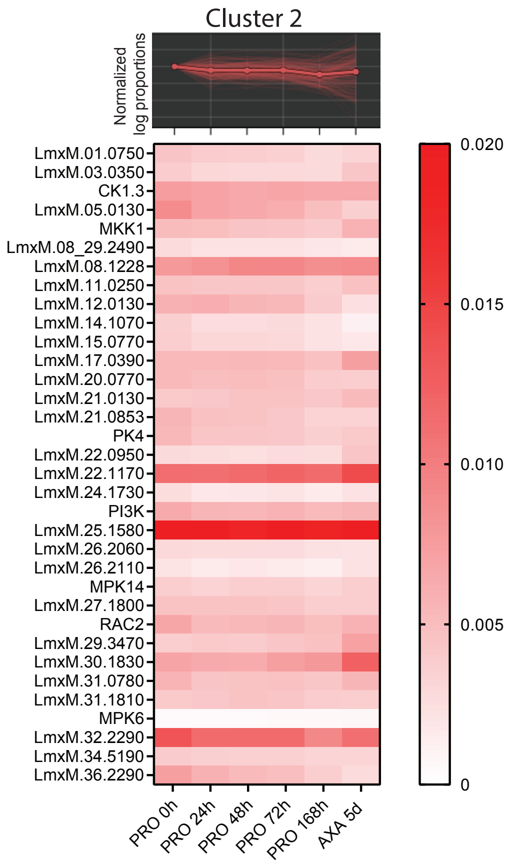

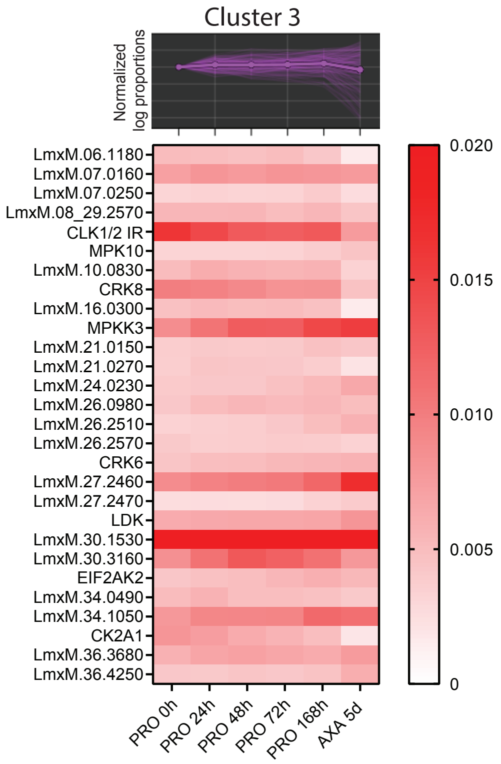

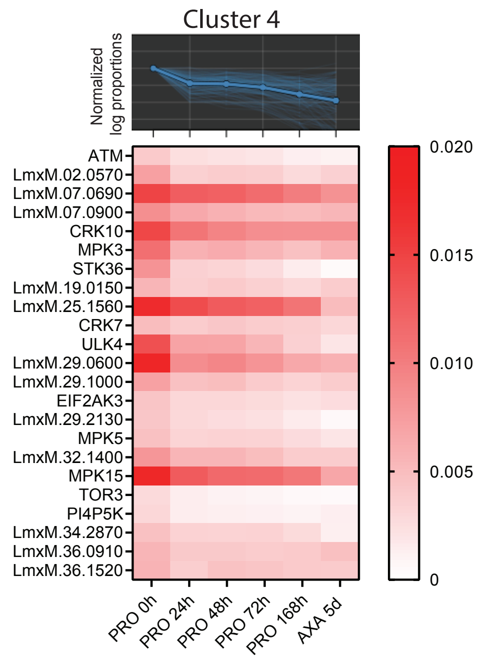

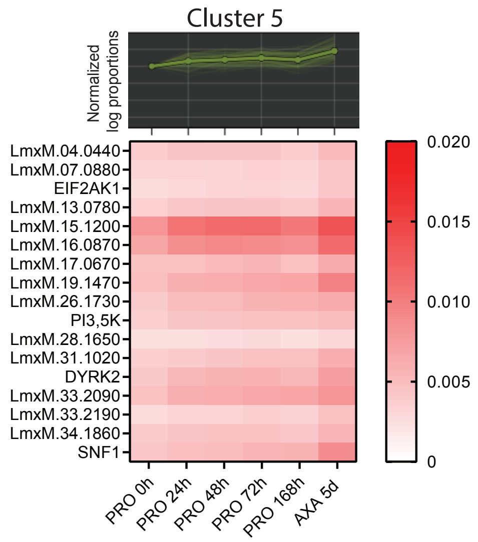

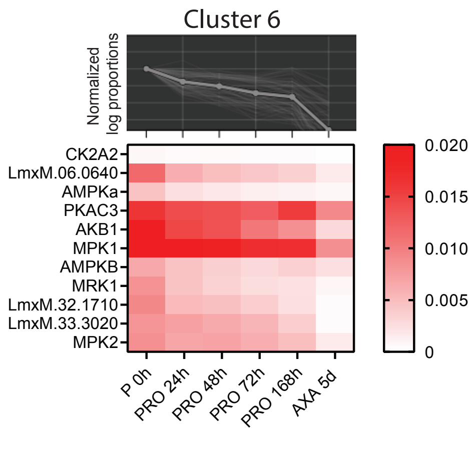

Supplementary Data 8b

Individual plots of Axenic amastigote data

EA1

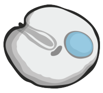

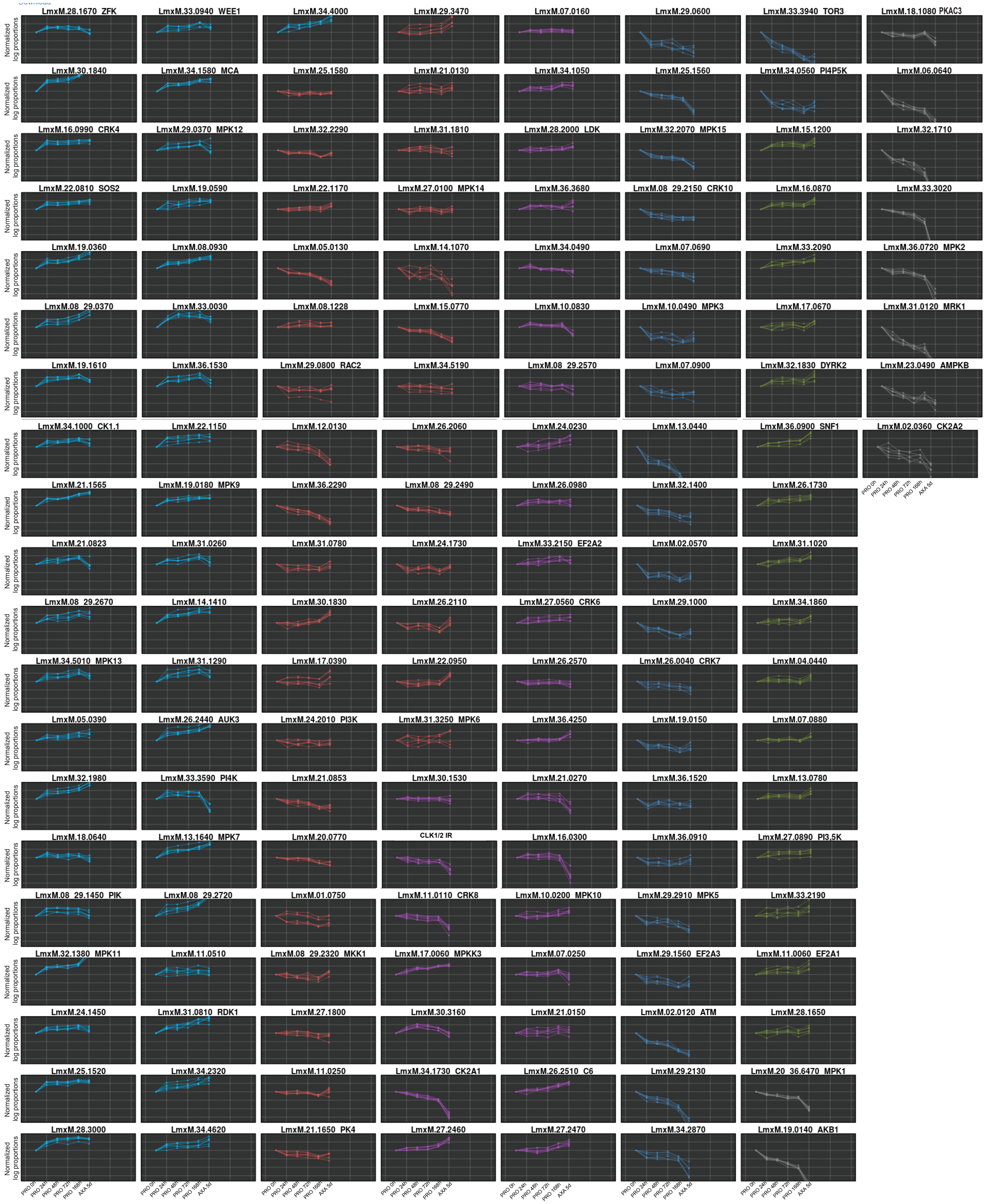

Supplementary Data 8c

Heat maps for macrophage infection data

EA2

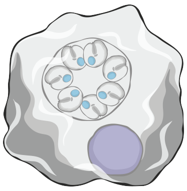

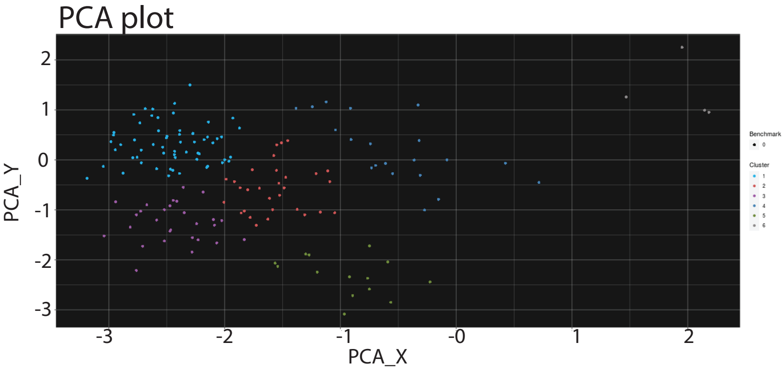

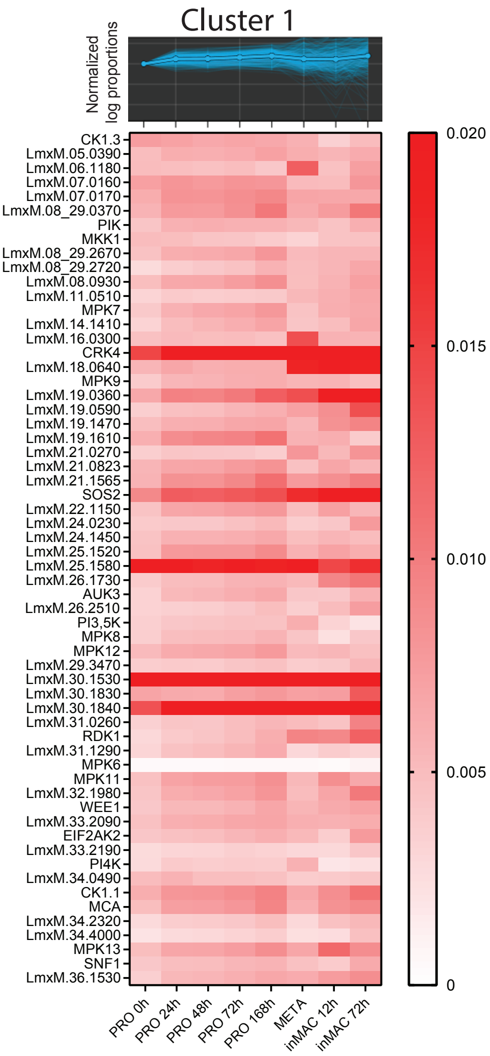

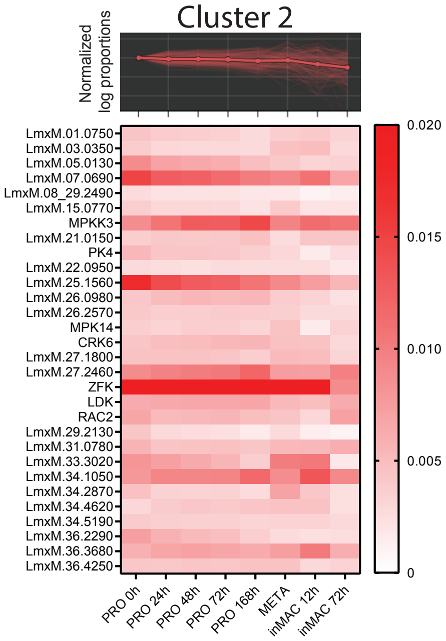

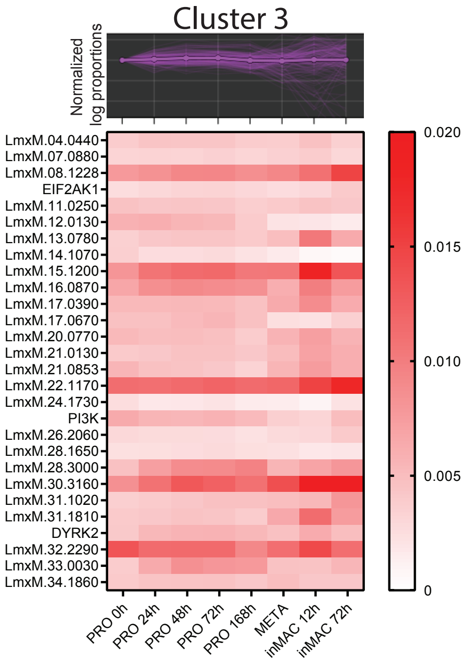

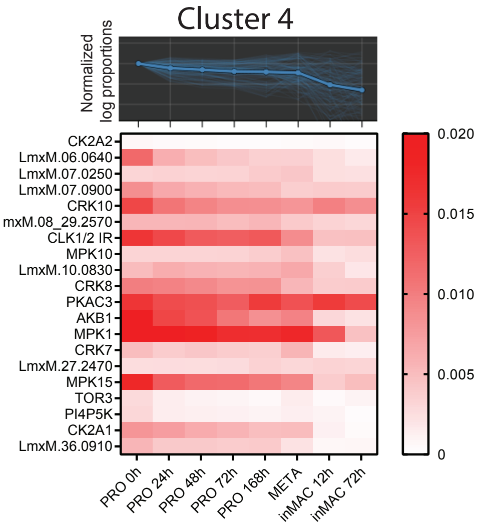

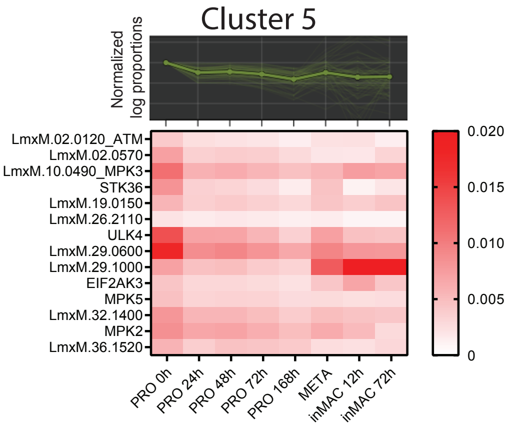

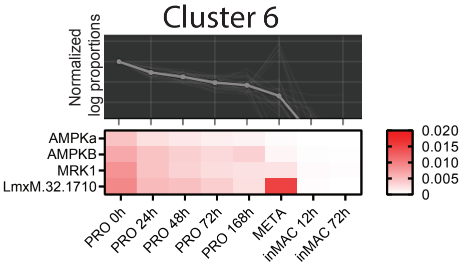

Supplementary Data 8d

Individual plots of macrophage infection data

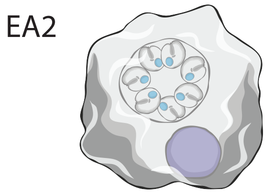

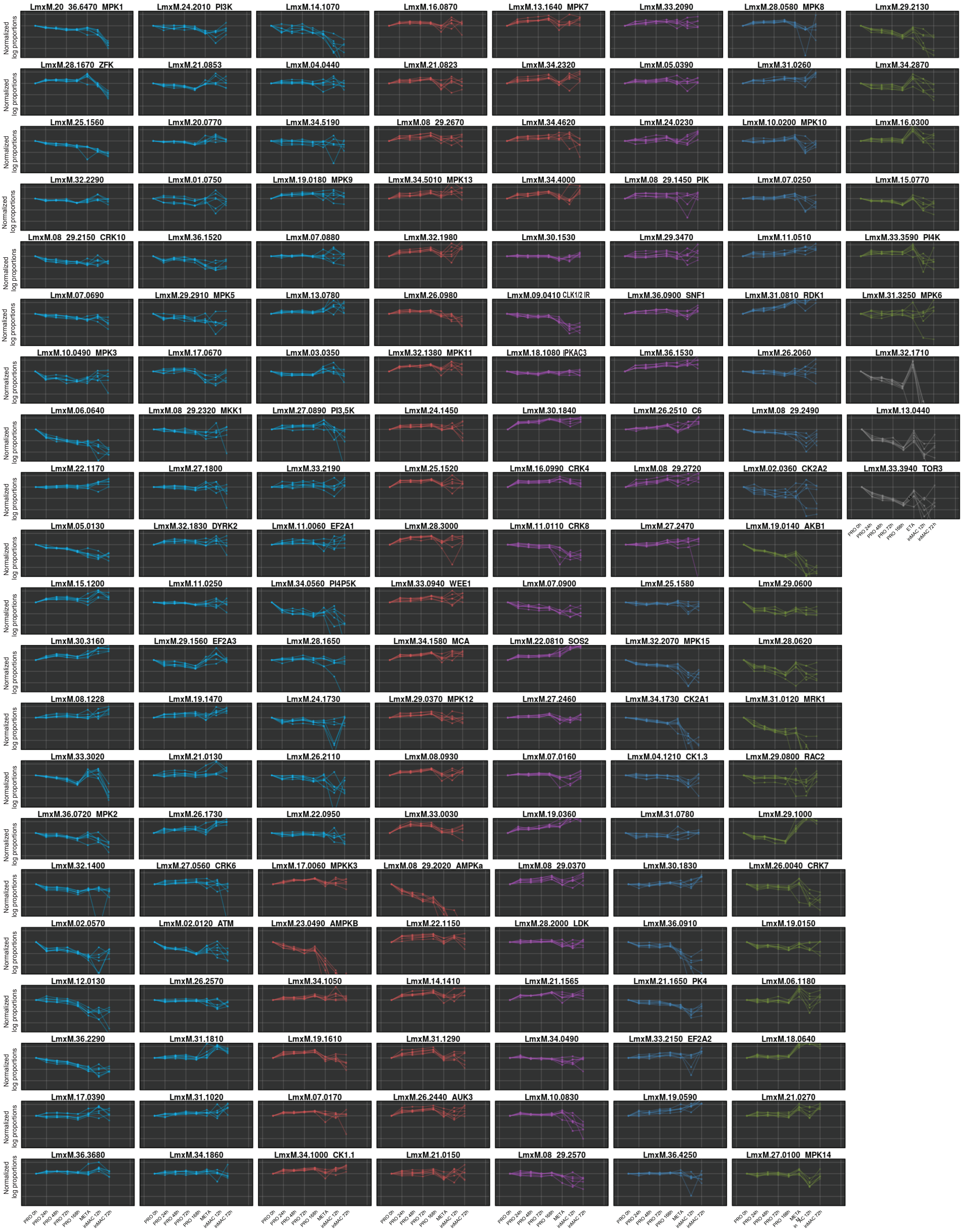

Supplementary Data 8e

Heat maps for mouse footpad infection data

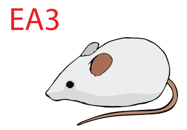

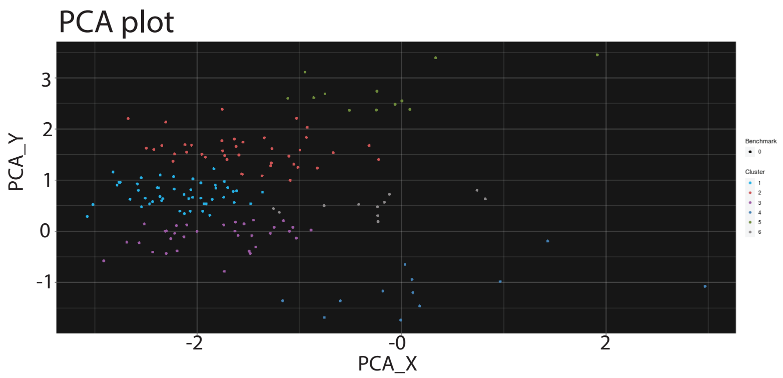

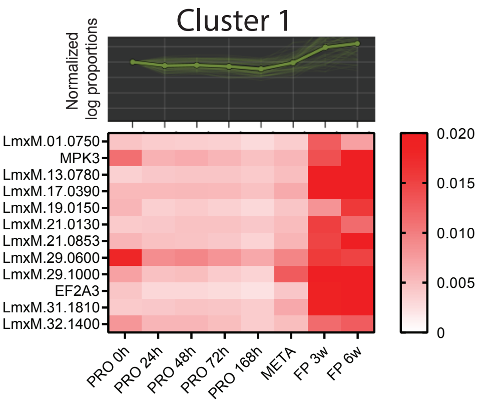

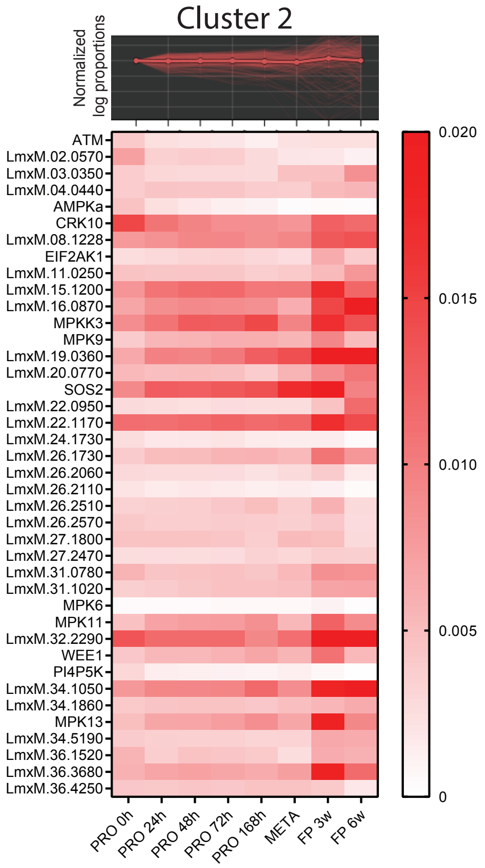

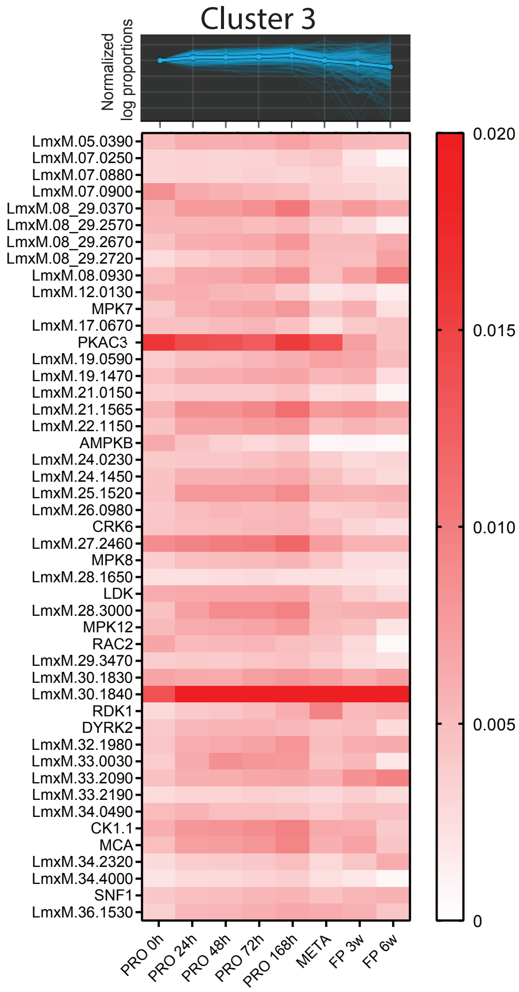

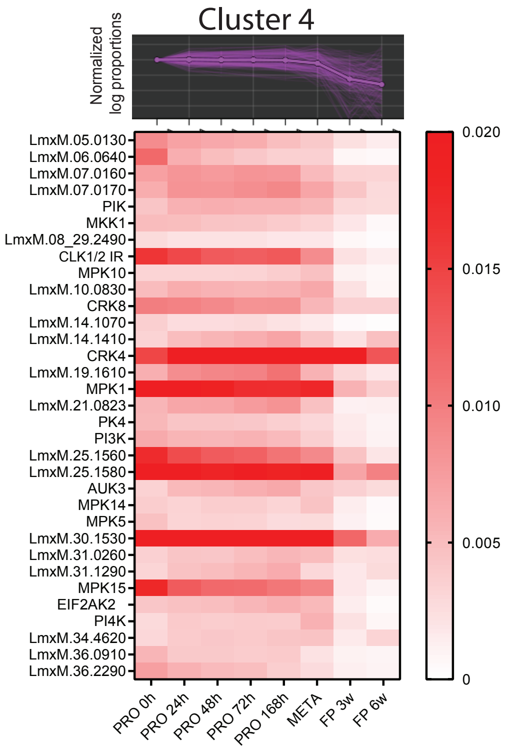

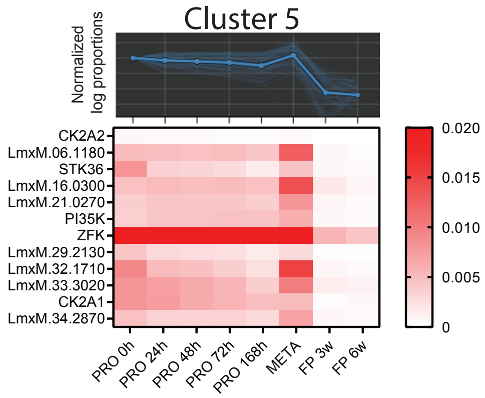

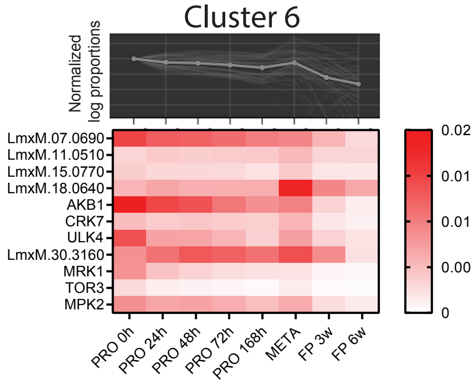

Supplementary Data 8f

Individual plots of mouse footpad infection data

EA3

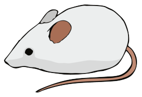

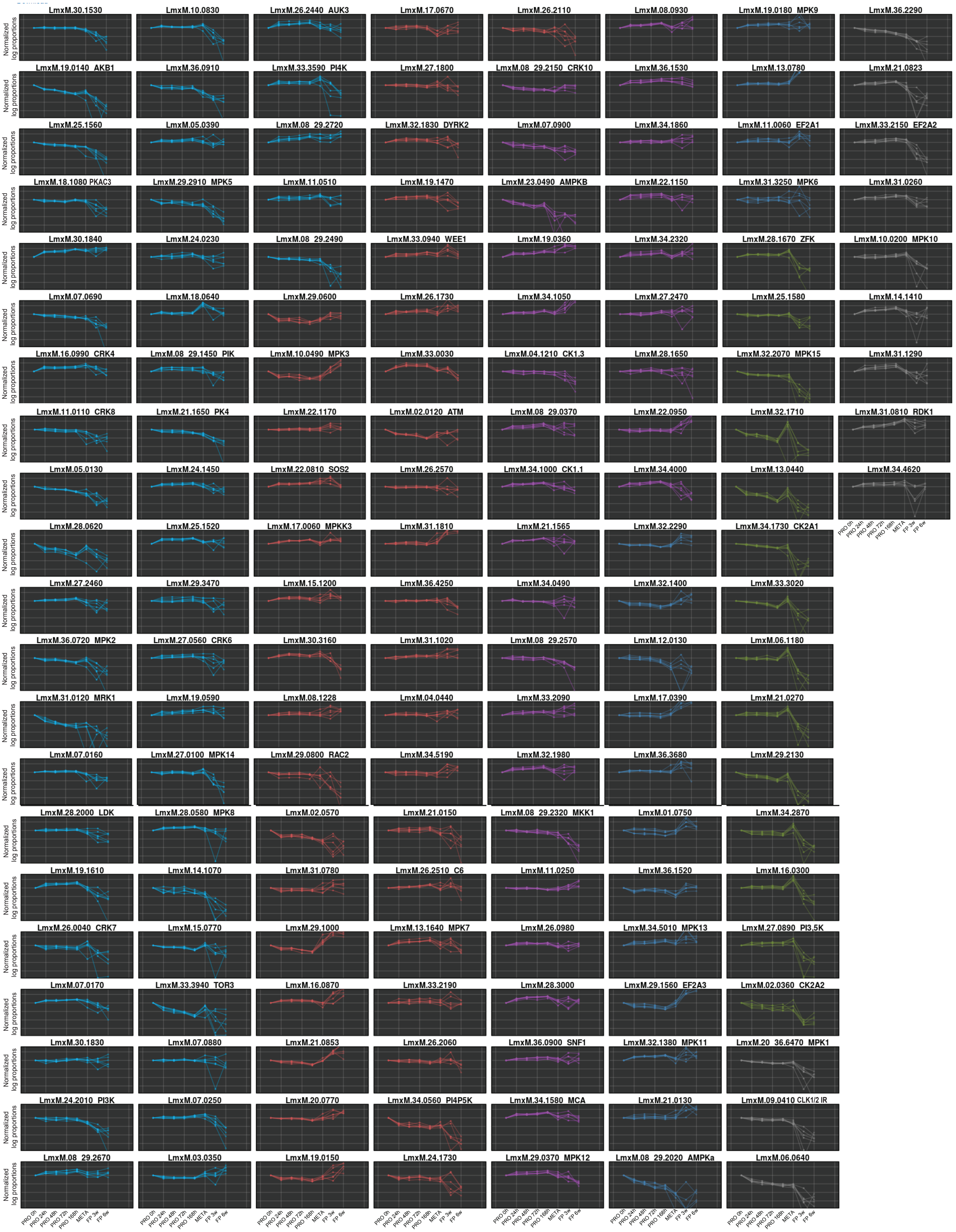

Supplement: Supplementary file 8 — Supplementary Data 5 [file 41467_2021_21360_MOESM8_ESM.pdf]
