# Supplementary material for: Systematic functional analysis of Leishmania protein kinases identifies regulators of differentiation or survival
Source: Nat Commun. 2021 Feb 23;12:1244. doi: 10.1038/s41467-021-21360-8 (PMC7902614; doi:10.1038/s41467-021-21360-8)
Supplement: Supplementary file 13 — Supplementary Software [file 41467_2021_21360_MOESM13_ESM.zip › Leish_clustering_code_v3/index.htm]

# Leish\_cluster\_analysis
