# Supplementary material for: Systematic functional analysis of Leishmania protein kinases identifies regulators of differentiation or survival
Source: Nat Commun. 2021 Feb 23;12:1244. doi: 10.1038/s41467-021-21360-8 (PMC7902614; doi:10.1038/s41467-021-21360-8)
Supplement: Supplementary file 14 — Reporting Summary [file 41467_2021_21360_MOESM14_ESM.pdf]

nature research

Corresponding author(s): J.C. Mottram

Last updated by author(s): 14/12/2020

# Reporting Summary

Nature Research wishes to improve the reproducibility of the work that we publish. This form provides structure for consistency and transparency in reporting. For further information on Nature Research policies, see our Editorial Policies and the Editorial Policy Checklist.

## Statistics

For all statistical analyses, confirm that the following items are present in the figure legend, table legend, main text, or Methods section.

| n/a | Confirmed | |
|---|---|---|
| ☐ | ☒ | The exact sample size (*n*) for each experimental group/condition, given as a discrete number and unit of measurement |
| ☐ | ☒ | A statement on whether measurements were taken from distinct samples or whether the same sample was measured repeatedly |
| ☐ | ☒ | The statistical test(s) used AND whether they are one- or two-sided<br>*Only common tests should be described solely by name; describe more complex techniques in the Methods section.* |
| ☐ | ☒ | A description of all covariates tested |
| ☐ | ☒ | A description of any assumptions or corrections, such as tests of normality and adjustment for multiple comparisons |
| ☐ | ☒ | A full description of the statistical parameters including central tendency (e.g. means) or other basic estimates (e.g. regression coefficient) AND variation (e.g. standard deviation) or associated estimates of uncertainty (e.g. confidence intervals) |
| ☐ | ☒ | For null hypothesis testing, the test statistic (e.g. *F*, *t*, *r*) with confidence intervals, effect sizes, degrees of freedom and *P* value noted<br>*Give P values as exact values whenever suitable.* |
| ☒ | ☐ | For Bayesian analysis, information on the choice of priors and Markov chain Monte Carlo settings |
| ☒ | ☐ | For hierarchical and complex designs, identification of the appropriate level for tests and full reporting of outcomes |
| ☒ | ☐ | Estimates of effect sizes (e.g. Cohen's *d*, Pearson's *r*), indicating how they were calculated |

*Our web collection on statistics for biologists contains articles on many of the points above.*

## Software and code

Policy information about availability of computer code

| | |
|---|---|
| Data collection | No software was used. |
| Data analysis | The custom Bar-seq count script was written in Python 3.7. Custom code for Bar-seq statistical analysis was written in R (v.3.4.4) and are provided. The graphical user interface is enabled by the R Shiny package (v.1.4.0). For non-R users the data can be assessed in https://benpowell.shinyapps.io/Experiment_5_May_2020/ (20 hours/month). Commercial software GraphPad Prism v 8.4 was used for statistical analysis. Analysis of whole genome sequencing data was performed with Cutadapt version 2.5, bwa version 0.7.17 and Mosdepth 0.2.6. Microscopy images were analysed using Zen-2.6 blue edition (Zeiss). |

For manuscripts utilizing custom algorithms or software that are central to the research but not yet described in published literature, software must be made available to editors and reviewers. We strongly encourage code deposition in a community repository (e.g. GitHub). See the Nature Research guidelines for submitting code & software for further information.

## Data

Policy information about availability of data

All manuscripts must include a data availability statement. This statement should provide the following information, where applicable:

- Accession codes, unique identifiers, or web links for publicly available datasets
- A list of figures that have associated raw data
- A description of any restrictions on data availability

The datasets generated and analyzed during the current study are available in the publication and associated supplementary material. Whole genome sequencing Data for Leishmania mutants and all bar-seq data have been deposited at European Nucleotide Archive under study accession PRJEB40373

# Field-specific reporting

Please select the one below that is the best fit for your research. If you are not sure, read the appropriate sections before making your selection.

- [x] Life sciences
- [ ] Behavioural & social sciences
- [ ] Ecological, evolutionary & environmental sciences

For a reference copy of the document with all sections, see nature.com/documents/nr-reporting-summary-flat.pdf

# Life sciences study design

All studies must disclose on these points even when the disclosure is negative.

| | |
|---|---|
| Sample size | For Bar-seq sample size (n=6) was decided in order to achieve >96% power to detect a two-fold change (or more) at a 5% false discovery rate (Robinson DG, Chen W, Storey JD, Gresham D. Design and analysis of Bar-seq experiments. G3 (Bethesda). 2014;4(1):11-18. Published 2014 Jan 10. doi:10.1534/g3.113.008565.<br>For the testing of virulence for the rdk1 gene deletion mutant sample size was determined on the basis of the minimum number of animals required for good data distribution and statistics as described in PMID: PMID: 30850532 |
| Data exclusions | For Bar-seq time-point low-count outliers that likely resulted from sequencing errors were removed as described in Robinson DG, Chen W, Storey JD, Gresham D. Design and analysis of Bar-seq experiments. G3 (Bethesda). 2014;4(1):11-18. Published 2014 Jan 10. doi:10.1534/g3.113.008565. |
| Replication | Each bar-seq screen was carried out with 6 biological replicates. |
| Randomization | Animal studies: Blinding was not possible in the animal models, but animals were selected randomly for each group. |
| Blinding | N/A |

# Reporting for specific materials, systems and methods

We require information from authors about some types of materials, experimental systems and methods used in many studies. Here, indicate whether each material, system or method listed is relevant to your study. If you are not sure if a list item applies to your research, read the appropriate section before selecting a response.

## Materials & experimental systems

| n/a | Involved in the study | |
|---|---|---|
| [ ] | [x] | Antibodies |
| [ ] | [x] | Eukaryotic cell lines |
| [x] | [ ] | Palaeontology and archaeology |
| [ ] | [x] | Animals and other organisms |
| [x] | [ ] | Human research participants |
| [x] | [ ] | Clinical data |
| [x] | [ ] | Dual use research of concern |

## Methods

| n/a | Involved in the study | |
|---|---|---|
| [x] | [ ] | ChIP-seq |
| [x] | [ ] | Flow cytometry |
| [x] | [ ] | MRI-based neuroimaging |

## Antibodies

| | |
|---|---|
| Antibodies used | Anti-myc tag monoclonal clone 4A6 (Millipore) (Cat: Sigma-Aldrich 05-724)<br>Anti-mouse IgG (H+L) conjugated to HRP (Molecular Probes) (Cat: ThermoFisher Scientific 62-6520) |
| Validation | Anti-myc monoclonal clone 4A6 antibody and anti-mouse IgG conjugated to HRP were validated previously PUBMED: 25929859 |

## Eukaryotic cell lines

Policy information about cell lines

| | |
|---|---|
| Cell line source(s) | Leishmania mexicana (MNYC/BZ/62/M379) expressing T7 RNA Pol and SpCas9 was published in Beneke et al (2017) R Soc Open Sci 4:170095 |
| Authentication | The parasite cell line was authenticated via testing for expected phenotypes in in vivo or in vitro infections and by whole genome sequencing. |
| Mycoplasma contamination | The cells were not tested for Mycoplasma |

| | |
|---|---|
| Commonly misidentified lines (See ICLAC register) | Not applicable. |

## Animals and other organisms

Policy information about studies involving animals; ARRIVE guidelines recommended for reporting animal research

| | |
|---|---|
| Laboratory animals | BALB/c mice, female, 4-6 weeks. All mice were housed in IVC caging and ambient temperatures are between 19-23C, humidity levels were 55% plus or minus 10%. There was a 12 hour light/dark cycle, 7.30am to 7.30pm.<br>Female Lutzomyia longipalpis (Jacobina strain( between 3 and 5 days old. The L. longipalpis colony was maintained at 26°C and high humidity on 50% sucrose solution and a 12 h light/12 h dark photoperiod |
| Wild animals | The study does not use animals collected from the wild. |
| Field-collected samples | The study does not use animals collected from the field. |
| Ethics oversight | All experiments were conducted according to the Animals (Scientific Procedures) Act of 1986, United Kingdom, and had approval from the University of York Animal Welfare and Ethical Review Body (AWERB) committee. |

Note that full information on the approval of the study protocol must also be provided in the manuscript.
